# Turning Red without Feeling Embarrassed—Xanthenium-Based Photocages for Red-Light-Activated Phototherapeutics

**DOI:** 10.1021/jacs.2c11499

**Published:** 2023-02-08

**Authors:** Alexandra Egyed, Krisztina Németh, Tibor Á. Molnár, Mihály Kállay, Péter Kele, Márton Bojtár

**Affiliations:** † Chemical Biology Research Group, Institute of Organic Chemistry, Research Centre for Natural Sciences, Magyar tudósok krt. 2., H-1117 Budapest, Hungary; ‡ Hevesy György PhD School of Chemistry, Eötvös Loránd University, Pázmány Péter sétány 1/a., H-1117 Budapest, Hungary; § Department of Physical Chemistry and Materials Science, Faculty of Chemical Technology and Biotechnology, Budapest University of Technology and Economics, Műegyetem rkp. 3., H-1111 Budapest, Hungary; ∥ ELKH-BME Quantum Chemistry Research Group, Műegyetem rkp. 3., H-1111 Budapest, Hungary; ⊥ MTA-BME Lendület Quantum Chemistry Research Group, Műegyetem rkp. 3., H-1111 Budapest, Hungary

## Abstract

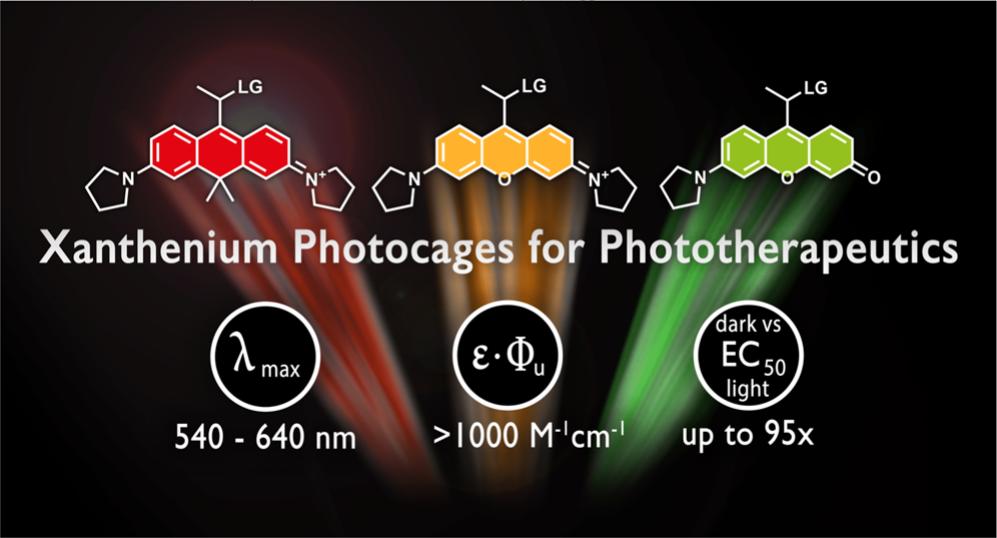

Herein, we present high-yielding, concise access to a set of xanthenium-derived, water-soluble, low-molecular-weight photocages allowing light-controlled cargo release in the green to red region. Very importantly, these new photocages allow installation of various payloads through ester, carbamate, or carbonate linkages even at the last stage of the synthesis. Payloads were uncaged with high efficiency upon green, orange, or red light irradiation, leading to the release of carboxylic acids, phenols, and amines. The near-ideal properties of a carboxanthenium derivative were further evaluated in the context of light-controlled drug release using a camptothecin-derived chemotherapeutic drug, SN38. Notably, the caged drug showed orders of magnitude lower efficiency *in cellulo*, which was reinstated after red light irradiation. The presented photocages offer properties that facilitate the translation of photoactivated chemotherapy toward clinical applications.

## Introduction

**Figure 1:**
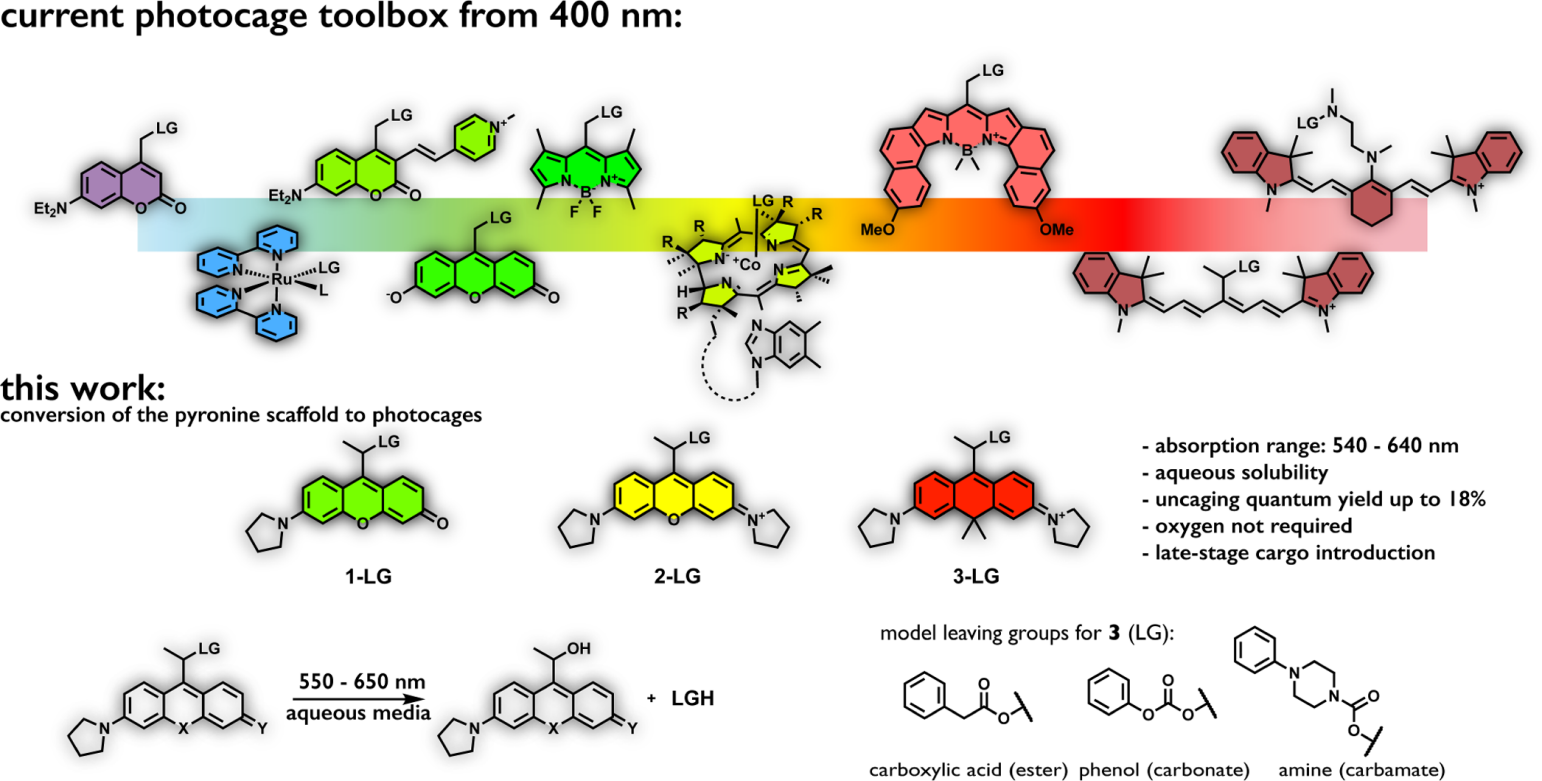
Examples of state-of-the-art photocages (top) and photocages of this work (bottom).

Recent developments in the field of photoresponsive materials have promoted light-related techniques to a precision tool allowing unparalleled spatiotemporal control over biological processes.^1,2^ Exemplified by the success of photodynamic therapy (PDT), where light is combined with exogenously delivered sensitizers to trigger spatiotemporally controlled generation of reactive oxygen species, further phototherapeutic approaches are foreseen to have profound implications on targeted therapies.^3^ Among such emerging approaches, photoactivated chemotherapy (PACT) has received increasing attention.^4−12^ PACT relies on the use of photolabile protecting groups (PPGs or photocages) that transiently disable the biological activity of cytotoxic drugs (payload, cargo) through a specific covalent linkage.^13−18^ Light irradiation of such photoresponsive prodrugs triggers the release of the reactivated drug *via* bond cleavage (photo-uncaging). PACT may represent an alternative to PDT, and more importantly, it has the promise of complementary action, where PDT fails (i.e., in hypoxic tumors).^9^ Despite its advantages and the availability of the directly transferable advanced technology of light delivery from PDT,^19^ clinical translation of PACT is still hindered by the lack of PPGs suitable for *in vivo* applications. To be suitable for clinical use, photocage platforms featuring strong one-photon absorption above 600 nm with efficient uncaging cross sections are needed.^20,21^ Besides, PPG-payload conjugates need to possess acceptable aqueous solubility and biological inertness to render such constructs biocompatible. Further important aspects include easy synthetic access allowing late-stage loading of precious cargos. Moreover, the covalent linkage between the photocages and their payloads is expected to be stable under biological conditions in the dark to prevent uncontrolled, premature release of cytotoxic payloads.^8^ Last but not least, oxygen independency of the photorelease process should also be considered especially when the hypoxic environment of solid tumors is the target area.^9^ Some of these challenges were addressed recently with the development of novel photocages such as π-extended coumarin,^22−25^ BODIPY,^26−31^ and cyanine frames^5,19,32,33^ as well as metal-complex-based PPGs (see Figure 1 for examples).^12,34−37^ Unfortunately, advancement into one direction often results in the loss of other important features. In practice, this usually translates to PPGs with absorption bands >600 nm with improved aqueous solubilities but considerably decreased photocaging quantum yields.^38^ A remarkable achievement was reported by Stacko et al. recently. Their cyanine-based photocages possessed a considerably high uncaging efficiency upon NIR light irradiation.^33^ However, its dependency on molecular oxygen and limited payload tolerance due to the need for an early-stage synthetic installation of the cargo pose serious limitations toward their applications in hypoxic settings.

Another very recent work by Klán and Weinstain has highlighted the efficiency of porphyrin-based PPGs both in their all-organic or metal-complexed forms.^34^ Their nonclassical metal-containing constructs enabled efficient release of caged pyridines or carboxylic acids independent of the presence of oxygen. However, the uncaging cross sections of their photocages were limited above 600 nm due to the low absorptivity of the PPGs in this wavelength range.

Development of novel PPGs is often inspired by the vast knowledge gained on the field of fluorescent dyes. Accordingly, the collection of visible-light-sensitive photocages includes a wide variety of coumarins, BODIPYs, porphyrins, or cyanines.^18^ The palette, however, lacks one of the most popular scaffolds used exhaustively in fluorescent imaging schemes, i.e., xanthenium dyes, such as rhodamines.^39^ Considering the excellent photophysical properties and ready availability of xanthenium dyes together with their spectral tunability, their absence from the palette is surprising, to put it mildly.

In a pioneering work in 2013, Klán and Wirz have recognized the potential of fluorescein-derived PPGs and studied the photocaging ability of a 9-methylxanthenium derivative.^40^ Indeed, the DDQ complex of their fluorescein-derived scaffold was found to be photolabile when irradiating with >500 nm light, while maintaining considerable dark stability. In their continuing study, they have explored the yellow-light-triggered C–C bond cleavage reactions of a pyronine derivative,^41^ with a recent report describing its complex photochemistry.^42^ Intriguingly, however, no further work with related scaffolds was pursued and the photocaging ability of xanthenes and pyronines remained unexplored.

We strongly believe that conversion of xanthene-derived frames to photocages could have a substantial impact on the phototherapeutic landscape. With this in mind, we set forth a systematic study aiming to explore the possibility of transforming a set of xanthenium scaffolds into photocages. We demonstrate here how these water-soluble, low-molecular-weight photocages with absorption maxima in the 530–640 nm range can be accessed through high-yielding, concise synthetic routes even in larger scale. A very important aspect is that these new photocages allow late-stage payload installation through various linkages (e.g., ester, carbamate, carbonate). Payloads caged through various functions were efficiently released upon green, orange, or red light (>650 nm) irradiation, leading to the oxygen-independent release of carboxylic acid, phenol, or amine cargos. A carboxanthenium-derived photocage with close-to-ideal features was further explored in the phototriggered release of a camptothecin payload (SN38). Notably, the photocaged drug possessed 2 orders of magnitude lower potency *in cellulo* that could be fully reinstated following a short pulse of red light irradiation.

## Results and Discussion

Our primary focus was set on dialkyl-substituted xanthenium-derived scaffolds as photolabile protecting groups. Following several attempts to convert the respective xanthones to the 9-hydroxymethyl derivatives, however, it became evident that the oxidized 9-hydroxymethyl scaffolds are unstable. Following payload attachment via esterification and subsequent oxidation with chloranil, we could only isolate a mixture of products where the respective rhodol and xanthenium analogues (notably, not in their chloranil complexed form) were contaminated with a colorless species having an *exo* double bond (see Supporting Information, SI Section S2.2 for some of our initial attempts). A similar *exo* form was reported by Johnsson in 2019 with a silicon rhodamine derivative.^43^ This colorless, undesired side product is considered as a tautomer in the case of rhodols and as the result of deprotonation for xanthenium derivatives.

**Scheme 1:**
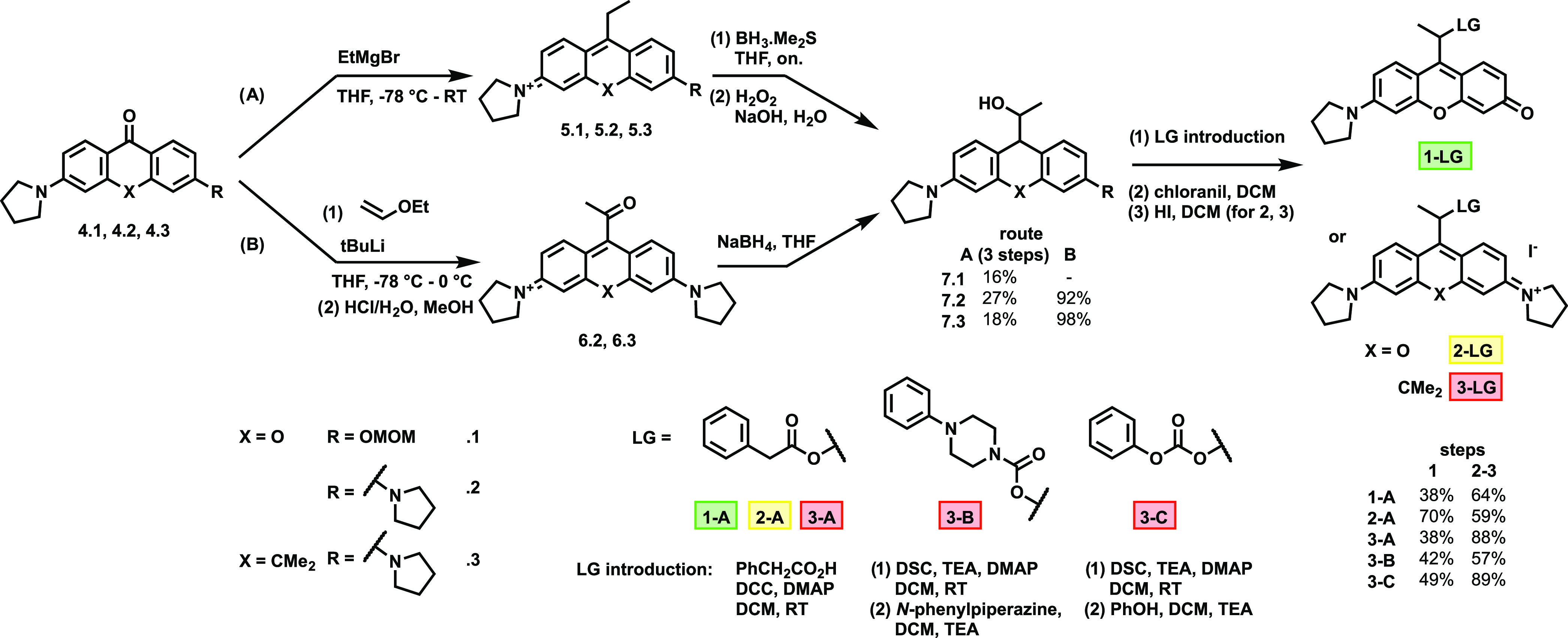
Synthesis and Structure of the Model Photocages

Recognizing these limitations, we assumed that the formation of the unwanted *exo* form could possibly be suppressed upon introduction of an additional substituent onto the carbon atom harboring the photolabile C–O bond. To this end, we devised structures where the key hydroxymethyl moiety in the 9-position was replaced by a 2-hydroxyethyl motif. Satisfyingly, this simple modification was sufficient to prevent the formation of the *exo* double bond and also provided synthetic solutions for the millimolar-scale access to key intermediates. Figure 1 (also Scheme 1) depicts structures of the devised rhodol (**1**), xanthenium (**2**), and carboxanthenium (**3**) photocages. Pyrrolidine was used as alkylamino substiuent(s), to improve photochemical quantum yields.^44,45^ The synthetic routes to access 2-hydroxyethyl derivatives proceed through the respective reduced (xanthene) forms (Scheme 1). These key intermediates can be synthesized from known xanthones (see Figure S1 in the SI for their synthesis) either via a Grignard reaction/hydroboration-oxidation sequence (A) or by an umpolung strategy (B) employing ethyl vinyl ether in the presence of tBuLi as an acyl anion synthon.^46^ This latter approach—that is not yet applicable for the rhodol derivative—also involves a hydrolysis step and subsequent reduction of both the chromophore and the appending carbonyl group. Remarkably, all steps of Route B turned out to be quantitative and did not require any subsequent chromatography.

With the photocage precursors (**7.1**–**7.3**) in hand, we have chosen a set of model payloads harboring a variety of cageable functions. Accordingly, phenylacetic acid (A), *N*-phenylpiperazine (B), and phenol (C) were selected and loaded onto **7**.**3** through ester, carbamate, and carbonate moieties, respectively. In the case of **7.1** and **7**.**2**, only the esters were prepared to assess the uncaging features of the photolabile scaffolds. These conjugates were accessed in high yields using standard coupling protocols. Following derivatization of **7.1**, **7.2**, and **7.3** with the payloads, rearomatization of the caging scaffolds was necessary, which was effected by chloranil as a mild oxidant. Subsequent treatment of the photocaged model compounds with hydrogen iodide resulted the target compounds as iodide salts to render the constructs sufficiently stable in physiological media.

**Figure 2:**
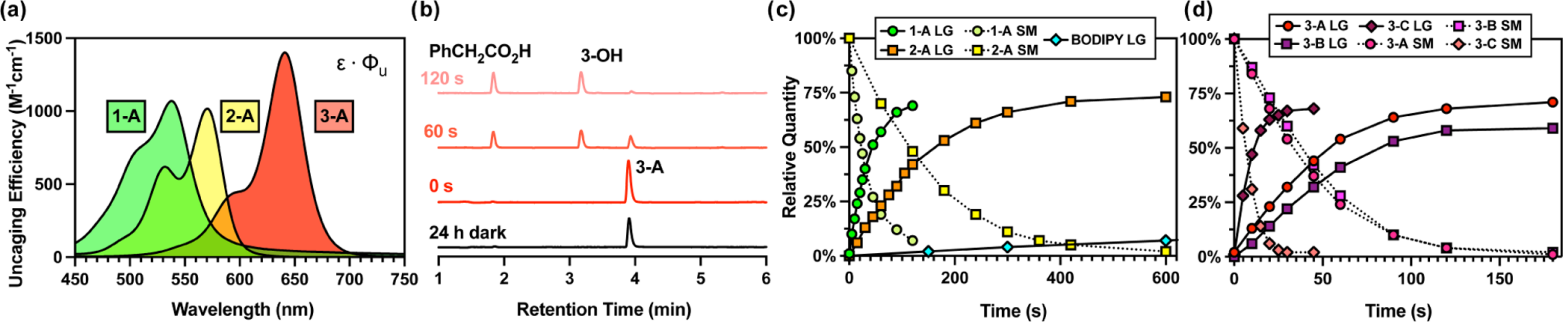
Uncaging of the model conjugates. (a) Wavelength-dependent uncaging efficiencies (ε·Φ) of **1-A**, **2-A**, and **3-A**; (b) exemplary chromatograms for the uncaging of **3-A** with red light; (c) uncaging of the payloads and degradation curves with green light, LG = leaving group (release) SM = starting material (degradation); and (d) uncaging of the payloads and degradation curves with red light (note that only 50% power was applied in case of **3-C**).

The spectroscopic characteristics of the model photocaged conjugates were then assessed. The absorption maxima of the photocages were found to be significantly red-shifted compared to the respective parent dyes, with high molar absorption coefficients in phosphate-buffered saline (PBS) solutions without any sign of aggregation up to 25 μM (Table 1 and Figures S1–S3 in the SI). Most notably, λ_max_ of the carboxanthenium derivatives was around 640 nm, a vital feature for phototherapeutic applications. Next, the photo-uncaging features of the model conjugates were evaluated by high performance liquid chromatography-mass spectrometry (HPLC-MS) using green (λ_max_ = 549 nm, output power: 72 mW for **1-A**), orange (λ_max_ = 605 nm for **2-A**, output power: 140 mW), and red (λ_max_ = 658 nm for **3**-**A**, output power: 210 mW) light-emitting diodes (LEDs) for irradiation (see Section S4.1 in the SI for details). The uncaging experiments were performed in 90% water/MeCN mixtures as well as in 90% PBS/MeCN (0.1 mM concentration). To our delight, irradiation of all photocaged conjugates led to the release of the respective payloads (Figures S6–S12 in the SI). The release efficiencies showed substantial differences with respect to the photocage, the linkage, and very importantly, the water content of the medium. Uncaging quantum yields of the release and degradation were calculated using a benchmark BODIPY (Figure S15 in the SI) in methanol^28^ and green light irradiation (see SI Section S4.3 for more details) and resulted in remarkably high efficiencies (Table 1). Of the ester-loaded photocages, **1-A** was the most efficient (Φ_u_ = 4.7%), while the quantum yields of **2-A** and **3-A** were lower (1.5 and 1.7%, respectively). Due to the high absorption coefficients, the uncaging efficiencies (Figure 2a) were found to be excellent, above 1000 M^–1^ cm^–1^. Photocage **2-A** was found to efficiently respond to orange light as well (Figure S7) but most importantly, red light irradiation of carboxanthenium **3-A** triggered ∼73% release of phenylacetic acid (see Figure S18 in the SI for the chemical yields). Comparison of the different linkages of photocage **3** with different payloads suggests that carbonate **3-C** was the most photolabile with a quantum yield of 18%, while carbamate **3-B** was uncaged with the lowest efficiency. Dark stabilities of the conjugates suggest that the ester and carbamate derivatives are stable in water or in PBS. Limited stability was observed, however, for carbonate **3-C**. Exemplary chromatograms can be seen in Figure 2b, and the release and degradation curves are shown in Figure 2c,d. Decreasing the water percentage of the uncaging medium resulted in a remarkable decrease in the uncaging efficiencies.

**Table 1:** Spectroscopic Properties and Photochemical Quantum Yields of Model Caged Conjugates in Water/MeCN (pH 7.4)

compound	λ_max_ (nm)	λ_em_ (nm)	ε × 10^4^ (M^–1^ cm^–1^)	Φ_u_ (%)^a^	Φ_deg_ (%)^b^	ε × Φ_u_ (M^–1^ cm^–1^)^c^
**1-A**	538	564	2.27	4.7	6.2	1 070
**2-A**	570	592	7.00	1.5	1.9	1020
**3-A**	641	674	8.60	1.7	2.2	1400
**3-B**	637	673	7.51	0.92	1.7	690
**3-C**	643	676	7.33	18	25	13,200

a Uncaging quantum yield, calculated from payload release.

b Degradation quantum yield, calculated from the disappearance of the conjugates; average of three trials, for the standard deviations, see Table S1 in the SI.

c Uncaging quantum efficiency at λ_max_.

Irradiation of **3-A** in water/MeCN mixtures with 10, 30, 50, 70, and 90% water content gave Φ_u_ values of 0.018, 0.037, 0.072, 0.28, and 1.7%, respectively (Table S2 and Figure S19 in the SI). Further studies revealed that oxygen is not required for the photolysis, and careful deoxygenation of the uncaging medium resulted in an increased uncaging quantum yield of **3-A** of 2.3% (Figure S20 in the SI).

Monitoring the uncaging process by HPLC-MS enabled us to follow the appearance of the liberated payloads. At the same time, the products of photolysis could also be identified. Studies suggest that in aqueous media, the main product, derived from the photocage upon photolysis, was the 2-hydroxyethyl derivative in each case. Unproductive recombination and photobleaching might lead to nonquantitative uncaging; however, we were not able to detect these photoproducts.

To unravel the mechanism of uncaging, we have synthesized bromo derivative **1′-Br** by halohydrin formation from the corresponding ethyl-rhodol derivative (see Scheme S8 in the SI for the structure and synthesis). *In situ* elimination and deprotection steps yielded **1′-Br**. Although the instability of **1′-Br** did not allow us to completely purify the product, we performed the irradiation experiments. Short pulses of green light in water/acetonitrile mixture or in methanol resulted in the appearance of the corresponding 2-hydroxyethyl or 2-methoxyethyl derivatives, respectively, suggesting an S_N_1-type photolysis mechanism involving a charge-separated C–X bond in the excited state with subsequent attack of the nucleophilic solvent (Figure S22 in the SI). The increased photolysis rates in media with higher water content are also in accordance with this mechanism.

**Figure 3:**
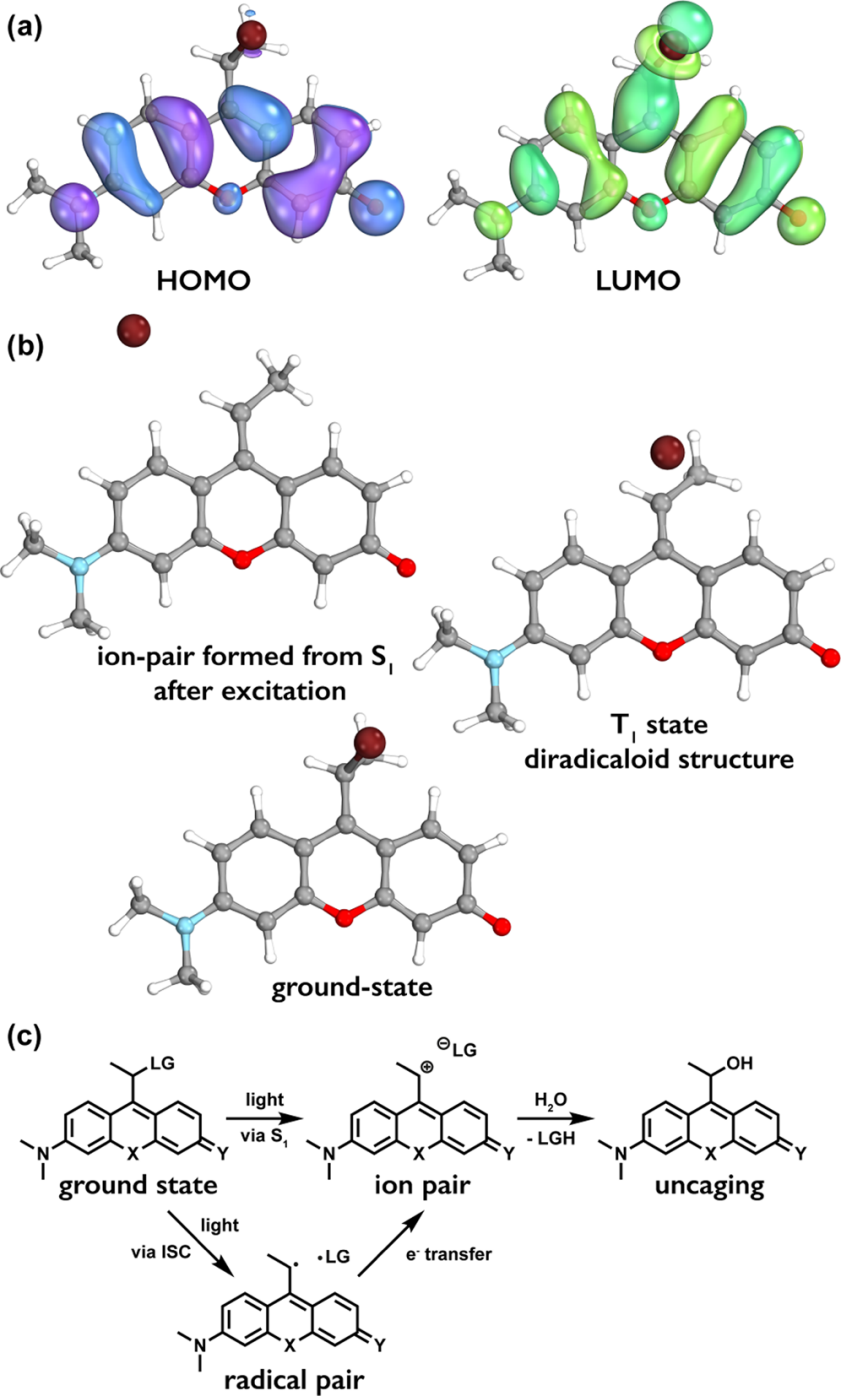
(a) Frontier molecular orbitals of **1′-Br**. (b) Ground-state geometry, the geometry of ion-pair formed after the excitation to the S_1_ state, and the geometry of the T_1_ state. (c) Suggested mechanism of uncaging.

We also wished to provide theoretical evidence for these experimental results. To this end, calculations were performed for the bromoethyl derivatives of dimethylamino substituted cores (**1′**, **2′**, and **3′**, Figure S24 in the SI) since these are the compounds with the simplest substituents. The ground-state geometries were optimized at the density-functional theory (DFT) level using the 6-311+G** basis set, and time-dependent density-functional theory (TD-DFT) geometry optimization was carried out with the same basis set for the first singlet excited state starting from the optimized ground-state structure. For **1′-Br** (rhodol core), the geometry optimization converged to a structure where the bromine atom is located 3.9 Å from the carbon atom to which it was originally connected, and about 2.8 Å from two hydrogen atoms of the molecule. The optimized structure is visualized in Figure 3b, where the ground-state geometry is also displayed for comparison. This suggests the formation of an ion-pair complex upon excitation and supports the S_N_1 mechanism of the reaction. The virtually zero activation energy leads to extremely fast uncaging as supported by experimental results. For **2′-Br** (xanthenium) and **3′-Br** (carboxanthenium), the excited-state geometry optimization resulted in structures that are close to the ground-state ones. Nevertheless, we suspected that ion-pair complexes similar to that for **1′-Br** can also form in the first excited state of those compounds. To prove this assumption, we carried out constrained potential energy surface scans for the S_1_ state. The Br–C bond was gradually increased by increments of 0.2 Å starting from the optimized bond length, and all of the other geometrical parameters were optimized. In other words, the minimum energy reaction path was determined for the dissociation of the Br–C bond. We found that the Br–C bond can dissociate through a low barrier, 17 and 14 kJ mol^–1^ for **2′** and **3′**, respectively, and ion-pairs similar to that for **1′** form. Not only does this suggest that the S_N_1 substitution reaction can also occur for the latter molecules, but it also explains the efficiency of the photolysis for the three compounds.

To further understand the mechanism of photolysis, it is instructive to inspect the electronic structure of the molecules. The lowest singlet excitation is dominated by the HOMO→LUMO transition for the rhodol derivatives. These frontier orbitals for **1′-Br** are depicted in Figure 3a (Figures S25–S27 in the SI for all compounds). Obviously, the excitation results in a significant increase in the electron density on the C-9 carbon atom of the rhodol scaffold, the connecting carbon atom, and the bromine substituent. This accumulation of negative charge is what facilitates the fission of the Br–C bond.

Since the homolytic fission of the Br–C bond is also feasible,^47,48^ further calculations were performed to check this possibility as well. Geometry optimizations were carried out for the T_1_ states of the three model compounds. The geometry optimizations for **1′** and **3′** converged to diradicaloid structures where the Br–C distance is significantly longer than its equilibrium value. These results suggest that a homolytic dissociation pathway is also possible, where, after the excitation to the S_1_ state, a fast intersystem crossing occurs to the T_1_ state, followed by the dissociation of the bromine atom.

As the theoretical results suggested a radical pair formation, we conducted preliminary experiments to reveal more details of the mechanism of uncaging. Uncaging of **3-A** was performed in the presence of singlet oxygen quencher (1 mM sodium azide), triplet state quencher (1 mM NiCl_2_),^49^ and TEMPO as radical scavenger. Photolysis in the presence of singlet oxygen or triplet quenchers (Table S2 in the SI) proceeded with a similar efficiency; however, when TEMPO was present, we observed an increase in uncaging efficiency (to 3.5%) and the formation of a large amount of 3-TEMPO adduct suggesting the involvement of a radical pair in the presence of the scavenger (see Figure S21 in the SI for scheme and the chromatograms). Based on the calculations and these experiments, we suggest that both pathways (i.e., via direct ion-pair formation or through a radical pair, with subsequent electron transfer leading to an ion pair) are productive and lead to the release of the payloads (Figure 3c). However, according to preliminary experiments, none of the photocaging scaffolds produce substantial amount of singlet oxygen (<1% in the case of **1-A**, **2-A**, and **3-A**) indicating that either the triplet state lifetime is too short to react with molecular oxygen or the intersystem crossing quantum yield is negligible. We are currently conducting further experiments to explore the fine details of the mechanism.

We also note that the existence of a third type of uncaging pathway cannot be excluded. Conical intersections may also exist between the S_0_ and S_1_ states, which can lead to internal conversion and dissociation on the S_0_ surface (see, e.g., ref (31) and references therein). Even if this is the case, it is unlikely to be the dominant photorelease mechanism because of the practically spontaneous dissociation in the S_1_ state.

**Scheme 2:**
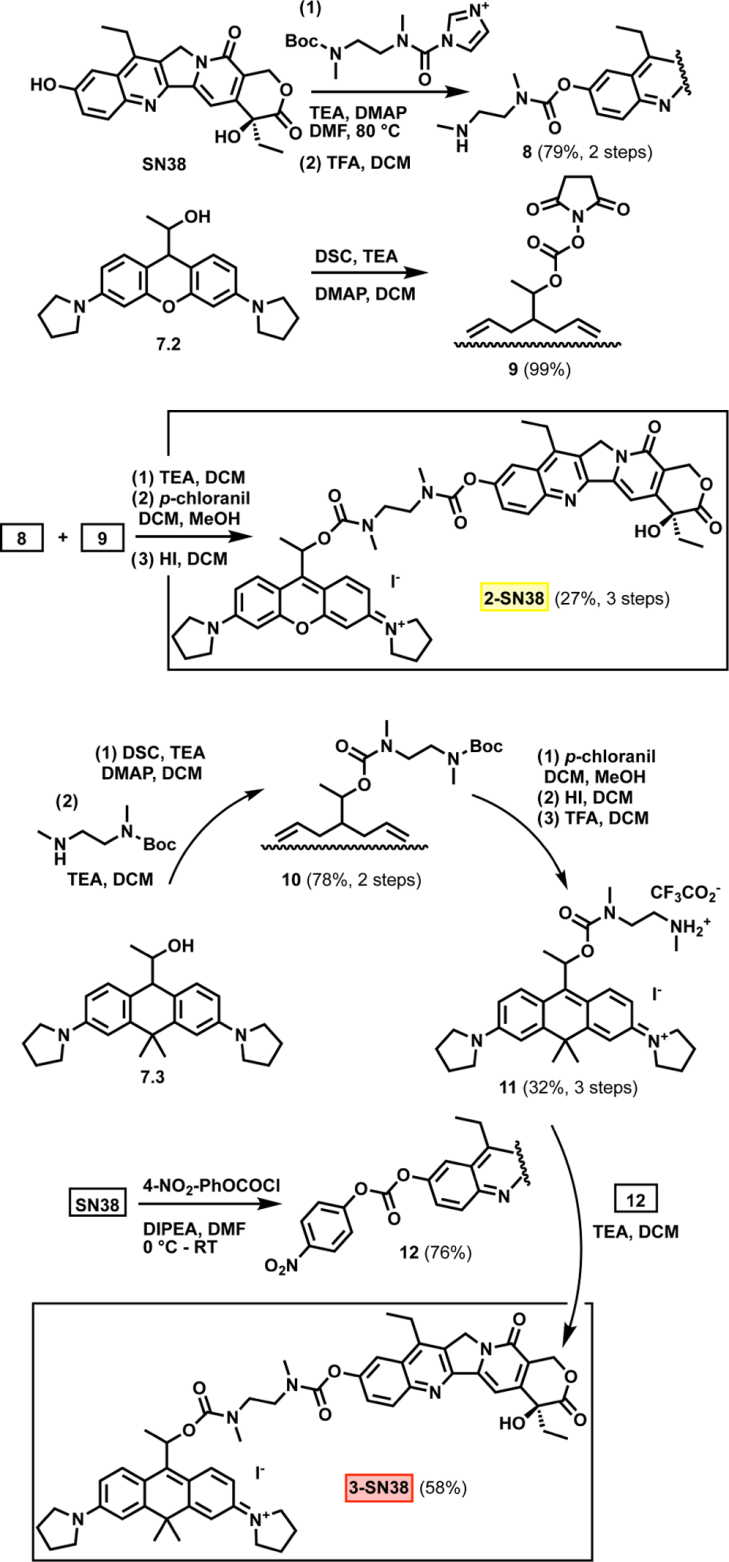
Synthesis of **2-SN38** and **3-SN38**

The good aqueous solubility and high photolysis quantum yields upon red light activation, combined with ready synthetic access need a biological demonstration. We were eager to try our orange- and red-light-sensitive photocages, **2** and **3**, in the controlled release of a selected potent cytotoxic agent, SN38 (7-ethyl-10-hydroxycamptothecin), an efficient inhibitor of topoisomerase I.^50^ The above synthetic route that allows easy derivatization of simple model payloads such as phenylacetic acid, might not be suitable to cage payloads that are potentially sensitive to oxidation. Although SN38 itself is not particularly sensitive to oxidants such as chloranil and indeed, we managed to synthesize **2-SN38** performing the oxidation in the final step (Scheme 2). We developed a synthetic strategy that enables cargo loading in the very last step through an *N*,*N*′-dimethylethylenediamine-based self-immolative linker. The use of a self-immolative linker also enables physiologically stable bridging of the photocage and the payload through carbamate linkages in both cases.

The synthetic scheme for the installation of SN38 onto **7.3** is also outlined in Scheme 2. In brief, a protected self-immolative linker was connected to the active carbonate of **7.3**. Subsequent rearomatization resulting in the xanthenium core was followed by removal of the Boc protecting group to yield **11**. The 4-nitrophenylcarbonate-activated SN38 (**12**) was then allowed to react with **11** resulting in **3-SN38** in high yield.

**2-SN38** and **3-SN38** maintained the spectroscopic properties of the parent photocages with water solubility that allows high concentration during uncaging experiments. Irradiation of the aqueous solution (0.1 mM in water with 10% MeCN) of **3-SN38** with red light led to the release of the linker-SN38 conjugate (less than 10% remaining **3-SN38** after 600 s of irradiation, Φ_dec_ = 0.18%) with subsequent self-immolation of the linker giving rise to the liberation of free SN38. The uncaging of **2-SN38** proceeded at a slightly slower rate using orange light but still within the range suitable for PACT applications.

**Figure 4:**
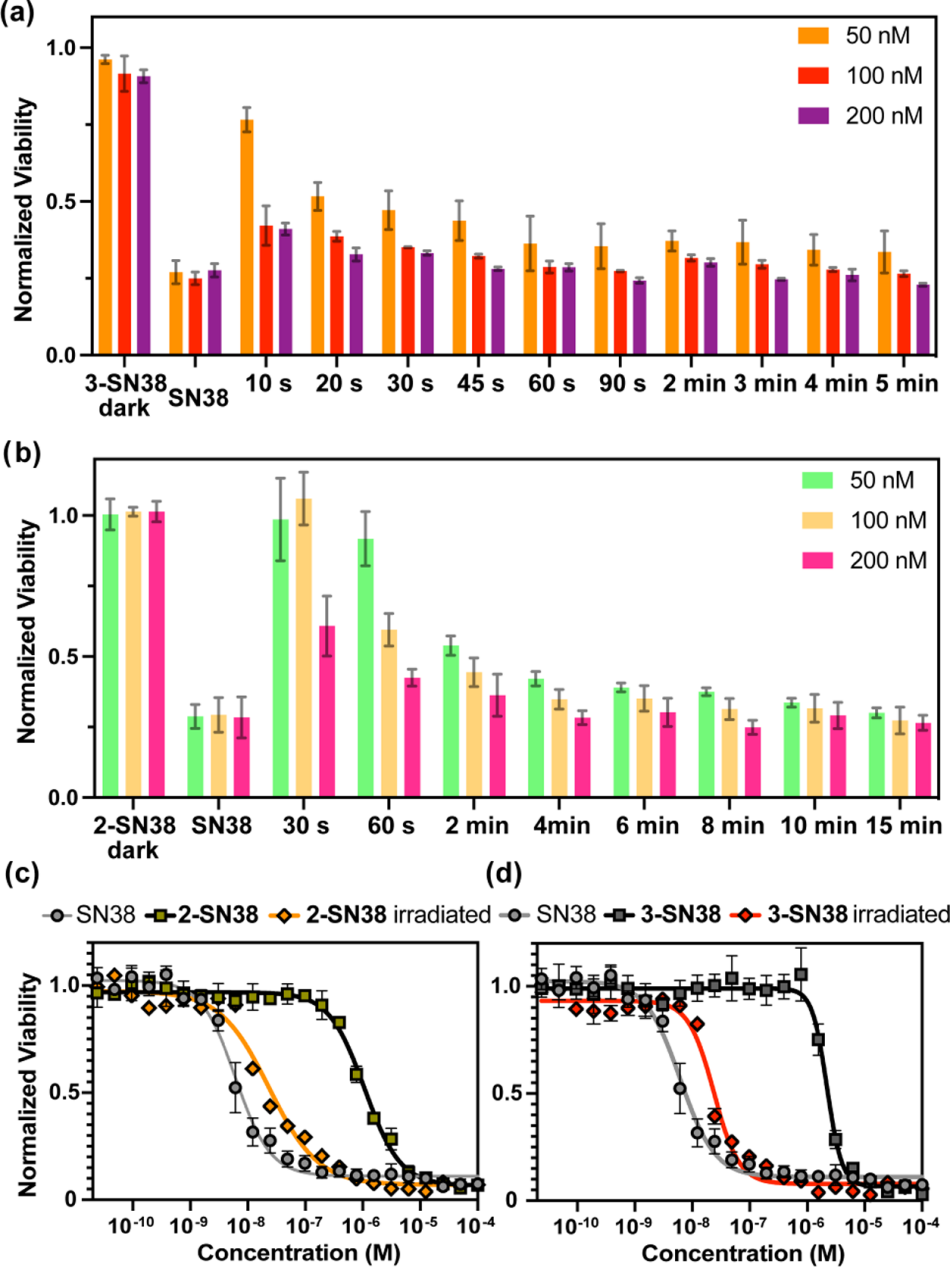
Cellular toxicities as determined by MTT assays of **2-SN38** and **3-SN38** in the dark and upon irradiation on SK-OV-3 cells. (a) Orange light irradiation time-dependent cellular toxicities of **2-SN38**; (b) red light irradiation time-dependent cellular toxicities of **3-SN38**; (c) concentration-dependent cellular toxicities of **2-SN38** after orange light irradiation for 5 min; (d) concentration-dependent cellular toxicities of **3-SN38** after red light irradiation for 60 s. For the statistical analysis of (a) and (b), refer to Tables S3 and S4 in the SI.

Unlike its caged derivative, free SN38 features an intensive green/yellow fluorescence allowing us to determine the first-order rate constant of the self-immolation step in PBS (containing 10% DMSO, pH 7.4). Rapid degradation of the linker-SN38 species was confirmed with a half-life of 1.6 min (Figure S23 in the SI). Taking advantage of the inherent fluorescence of the photocage cores, the cell permeability of **2-SN38** and **3-SN38** was tested in live SK-OV-3 (human ovarian carcinoma) cells using confocal microscopy. **2-SN38** was found to localize in the mitochondria (*R*_p_ = 0.832); however, unlike most rhodamine derivatives that commonly localize in the mitochondria, fluorescence of **3-SN38** co-localized with the emission of fluorescently stained lysosomes (*R*_p_ = 0.788, Figures S28 and S29 in the SI). Although these latter findings are currently without explanation, these experiments revealed that **SN38** conjugates of **2** and **3** are in fact cell permeable and suitable for intracellular applications. Next, we determined the photolysis-dependent cytotoxicities by standard 3-(4,5-dimethylthiazol-2-yl)-2,5-diphenyltetrazolium bromide (MTT) assays on the SK-OV-3 ovarian carcinoma cell line (Figure 4).

To irradiate multiple specimens, a custom-made LED panel was used. The panel having 24 LEDs is suitable to mount a 48-well plate. The board was specifically designed for commercial well plates and offers water cooling to minimize heat shock to cells. Each well can be irradiated with an individual LED (Figure S5 in the SI). First, we assessed the cytotoxicity of the free drug on SK-OV-3 cells. The measured 6.2 nM EC50 value is in agreement with reported values^48^ and confirms the high potency of SN38. Treatment of cells with **3-SN38** for 72 h in the dark (without washing) revealed that caged SN38 has almost 400-fold higher effective concentration (2.2 μM). While this experiment confirmed that the activity of SN38 can be lowered by caging the 10-hydroxy group, it also implicated that the conjugates have considerable dark stability even in the challenging environment of the lysosomes. This remarkable decrease in toxicity possibly originates from the lower ability of the conjugates to form the ternary complex with Top1 and DNA, especially if **3-SN38** is trapped inside the lysosomes although the EC50 of compound **2-SN38** was also significantly higher (1.1 μM) than the free drug. Next, we studied the toxic effects of the conjugates upon light activation. Satisfyingly, brief irradiation of the cells treated with **3-SN38** with red light (i.e., 60 s) resulted in the restoration of the activity of SN38 (EC50 for **3-SN38** after irradiation: 24 nM), providing an excellent photoindex of 95. Importantly, in this concentration range/optical length, the ratio of the caged/uncaged forms of the drug is only subject to the photon count and does not depend on the concentration due to the ultralow absorbance of the sample. Therefore, reducing the irradiation time down to 10 s still produced significant toxicity at higher **3-SN38** concentrations (see Figure 4a,b for time-dependent viabilities) even assuming incomplete uncaging owing to the considerable dark stability of the caged drug. This effect is particularly important for further *in vivo* applications where lower photon counts can create significantly decreased uncaging yields. Irradiation of **2-SN38** with orange light (300 s) resulted in a similar EC50 value (23 nM) and a photoindex of 45. To support the PACT mechanism and exclude any potential phototoxic effects (e.g., ^1^O_2_) of the carborhodamine scaffold, control experiments were performed with **11**, a derivative of **3** without SN38. Although moderate toxicity (4.3 μM) was in fact observed in the dark, only a slight decrease in viability (1.1 μM) was detected upon irradiation (Figure S31 in the SI).

The moderate dark toxicity of the carboxanthenium scaffold after 72 h might be attributed to the chromophore itself, and experiments to reduce this toxicity are currently underway in our laboratory. Nevertheless, these values also justify the choice of a drug with nanomolar potency such as SN38.

## Conclusions

Within this study, we have explored the possibility of turning xanthenium and rhodol chromophores into photolabile protecting groups. Initial evaluation of possible synthetic routes revealed that an additional methyl substituent on the photolabile carbon is required to extend the current palette with these chromophores. Photophysical studies with model PPG-payload conjugates of our newly synthesized rhodol-, xanthenium-, and carboxanthenium-derived water-soluble photocages resulted in highly efficient, light-controlled release of carboxylates, amines, or phenols in the green to red region without the requirement of oxygen. Further studies revealed that the conjugates are sufficiently stable in the dark under physiological conditions. Remarkably, the photocages allow installation of payloads, such as drugs at the very last stage, an aspect of profound importance in the case of sensitive or hardly accessible drugs.

Possessing near-ideal properties as a caging group, carboxanthenium derivative **3** was further studied and was conjugated to a topoisomerase inhibitor, SN38. The photoactivatable prodrug was applied to cells to reveal a photoindex around 100 and nanomolar activity upon red light irradiation.

Further work will focus on elucidating the mechanism of the uncaging and developing a general approach to convert further xanthene dyes such as Si-rhodamines or P-rhodamines into photocages. Nonetheless, we strongly believe that the presented photocages could truly facilitate the translation of photoactivated chemotherapy toward clinical applications as well as offer scientists tools for light-assisted manipulation of living systems.

## Supporting Information Available

Experimental details, synthetic procedures, spectroscopic characterization, physical data determination, details on the imaging experiments, further images, and viability assessment (PDF)
