# Supplementary material for: Turning Red without Feeling Embarrassed—Xanthenium-Based Photocages for Red-Light-Activated Phototherapeutics
Source: J Am Chem Soc. 2023 Feb 8;145(7):4026–34. doi: 10.1021/jacs.2c11499 (PMC9951246; doi:10.1021/jacs.2c11499)
Supplement: Supplementary file 1 — ja2c11499_si_001.pdf [file ja2c11499_si_001.pdf]

Supporting Information (SI)

Turning Red Without Feeling Embarrassed – Xanthenium-Based Photocages for Red-Light Activated Phototherapeutics

Alexandra Egyed,[a,b] Krisztina Németh,[a] Tibor Á. Molnár,[a] Mihály Kállay,[c, d] Péter Kele[a,]* and Márton Bojtár[a,]*

[a] *Chemical Biology Research Group, Institute of Organic Chemistry, Research Centre for Natural Sciences. Magyar tudósok krt. 2. H-1117, Budapest, Hungary. e-mail: kele.peter@ttk.hu; bojtar.marton@ttk.hu*

[b] *Hevesy György PhD School of Chemistry, Eötvös Loránd University, Pázmány Péter sétány 1/a. H-1117, Budapest, Hungary*

[c] *Department of Physical Chemistry and Materials Science, Faculty of Chemical Technology and Biotechnology, Budapest University of Technology and Economics, Műegyetem rkp. 3., H-1111 Budapest, Hungary*

[d] *ELKH-BME Quantum Chemistry Research Group, Műegyetem rkp. 3., H-1111 Budapest, Hungary*

[e] *MTA-BME Lendület Quantum Chemistry Research Group, Műegyetem rkp. 3., H-1111 Budapest, Hungary*

Contents

1. MATERIALS AND METHODS 3

2. SYNTHESIS OF THE COMPOUNDS 4

2.1. Synthetic Overview 4
2.2. Initial attempts to access 9-hydroxymethyl derivatives 7
2.3. Synthesis of the starting material xanthones 9
2.4. Synthesis of the photocage precursors – route A 10
2.5. Synthesis of the photocage precursors – route B 13
2.6. Synthesis of caged model compounds 15
2.7. Synthesis of photocaged SN38 derivatives 21
2.8. Synthesis of **1’-Br** 24

3. SPECTROSCOPIC PROPERTIES OF THE COMPOUNDS 25

4. UNCAGING STUDIES 28

4.1. Experimental details 28
4.2. HPLC chromatograms of the uncaging experiments 30
4.3. Photochemical quantum yield determination 38
4.4. Chemical yields of the uncaging 42
4.5. Uncaging of **3-A** in different conditions 42
4.6. Photolysis studies of **1’-Br** 45
4.7. Self-immolative kinetics of the SN38-linker 46

5. COMPUTATIONAL DETAILS 46

6. FLUORESCENCE MICROSCOPY 59

6.1. Cell sample preparation for fluorescence imaging experiments 59
6.2. Live cell fluorescence imaging 59
6.3. Colocalization Images 60

7. CYTOTOXICITY DETERMINATION 62

7.1. Experimental details 62
7.2. Irradiation time-dependent viabilities 63

8. NMR SPECTRA 66

9. REFERENCES 102

1. Materials and Methods

All starting materials were obtained from commercial suppliers (Sigma-Aldrich, Merck, Alfa Aesar, Reanal, Molar Chemicals, Fluorochem) and used without further purification. Analytical thin-layer chromatography (TLC) was performed on silica gel 60 F254 precoated aluminum TLC plates from Merck. Flash column chromatography was performed on Teledyne Isco CombiFlash® Rf+ automated flash chromatographer with silica gel (25-40 μm) from Zeochem. NMR spectra were recorded on a Varian Inova 500 MHz spectrometer. Chemical shifts (δ) are given in parts per million (ppm) using solvent signals or TMS as the reference. Coupling constants (J) are reported in Hertz (Hz). Analytical RP-HPLC-UV/Vis-MS measurements were employed using a Shimadzu LCMS-2020 instrument applying a Gemini C18 column (100 x 2.00 mm I.D.) in which the stationary phase is 5 μm silica with a pore size of 110 Å. The chromatograms were detected by a UV-Vis diode array (190-800 nm) and an ESI-MS detector. The following linear gradient elution profile was applied, 0 min 0 % B; 6.5 min 100 % B; 7 min 0 % B; 8 min. 0 % B) with eluent A (2 % HCOOH, 5 % MeCN and 93 % water) and B (2 % HCOOH, 80 % MeCN and 18 % water) at a flow rate of 1.0- or 0.8-mL min^{-1} at 40°C. The samples were dissolved in MeCN – water mixtures. Quantitative RP-HPLC-UV/VIS-MS measurements were performed on a similar setup, specifically with a 10-minute-long method with the low rate of 0.8 mL min^{-1}. The following linear gradient elution profile was applied: 0 min 0% B; 7.50 min 100% B; 8.13 min 100% B; 8.75 min 0% B; 10 min 0% B with the eluents A (2 % HCOOH, 5 % MeCN and 93 % water) and B (2 % HCOOH, 80 % MeCN and 18 % water) at 40 °C.

Semipreparative HPLC was performed on a Wufeng Chrom LC100 HPLC system using a Gemini C18 column (150 × 21 mm I.D.) with 5 μm silica (110 pore size) as a stationary phase. Spectroscopic measurements were performed on a Jasco FP 8300 spectrofluorometer. Quartz cuvettes with path length of 1 cm were used. The exact masses were determined with an Agilent 6230 time-of-flight mass spectrometer.

2. Synthesis of the Compounds

2.1. Synthetic Overview

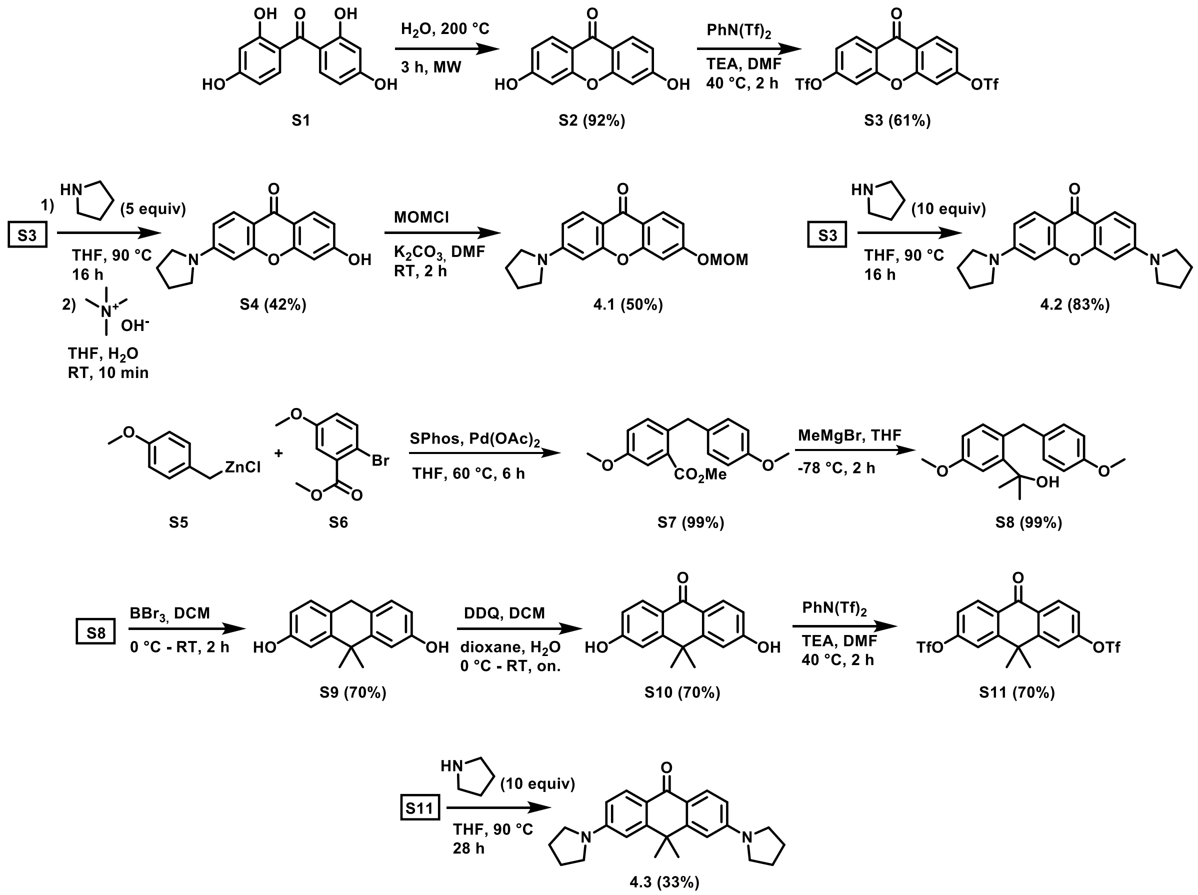

*Scheme S1 Synthesis of xanthones **4***

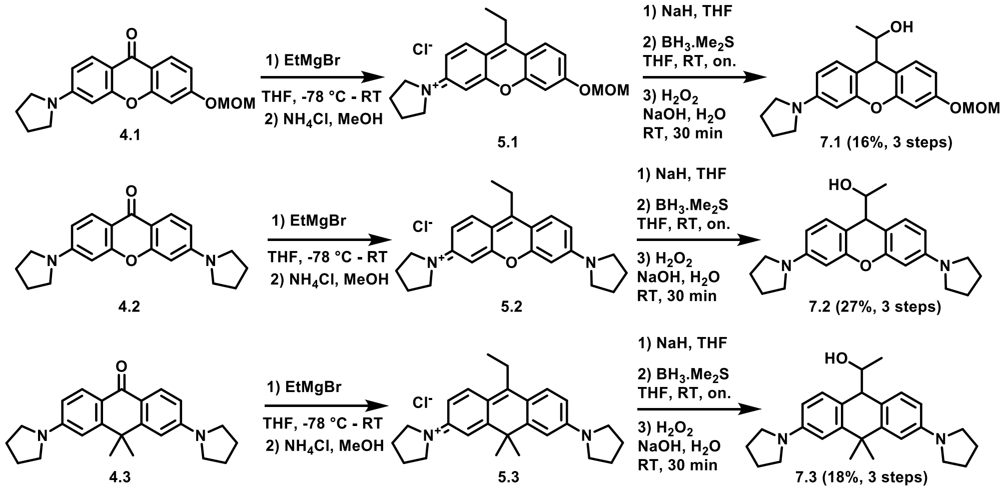

*Scheme S2 Synthesis of photocage precursors **7** – Route A (Grignard)*

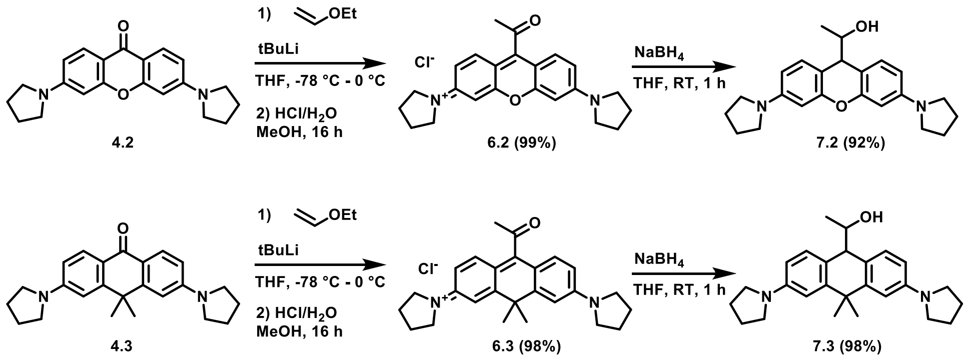

*Scheme S3 Synthesis of photocage precursors **7.2** and **7.3** – Route B (Umpolung)*

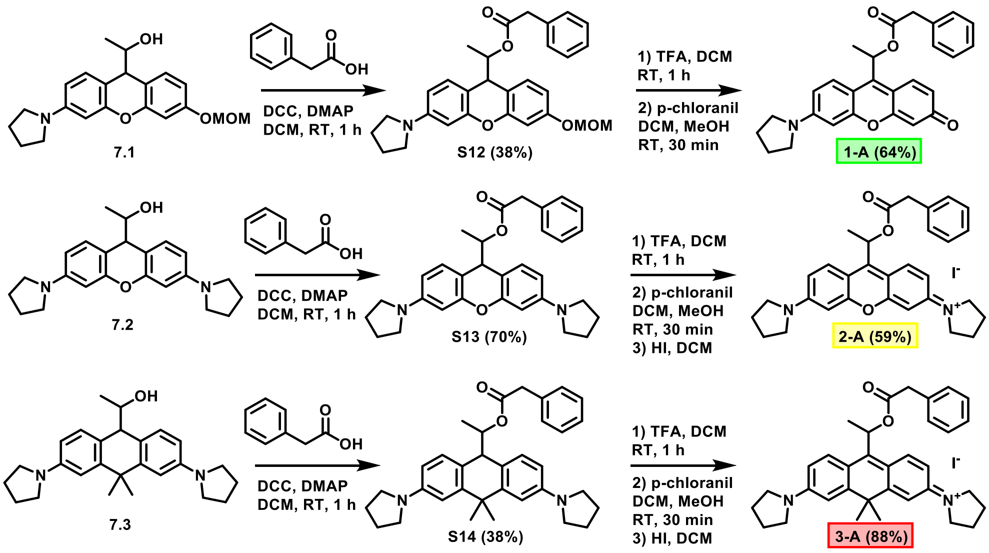

Scheme S4 Synthesis of photocaged model compounds – esters

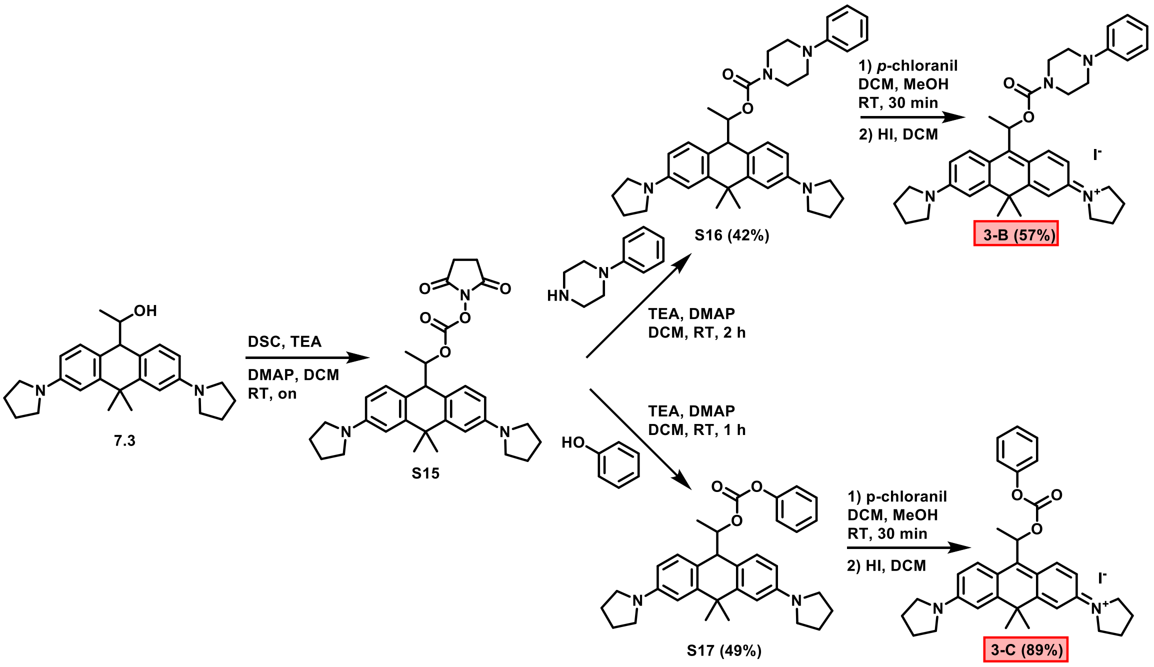

Scheme S5 Synthesis of photocaged model compounds – carbamate and carbonate

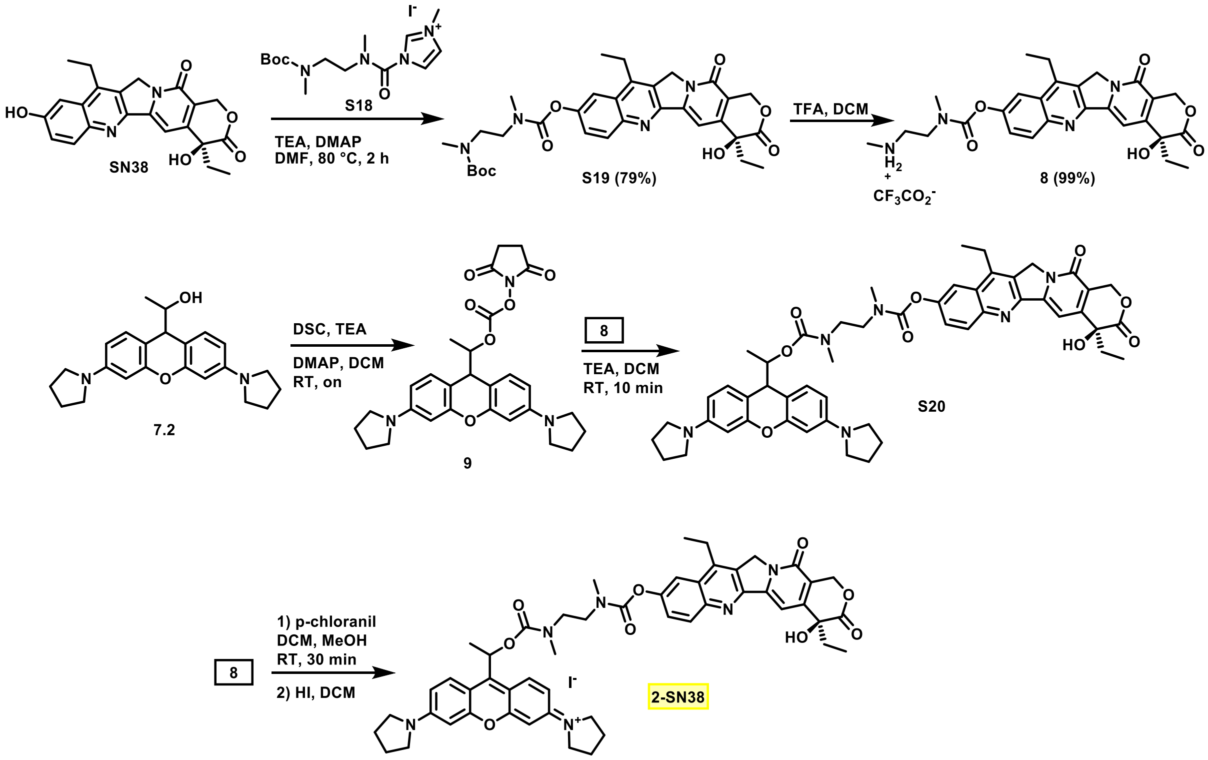

Scheme S6 Synthesis of ***2-SN38***

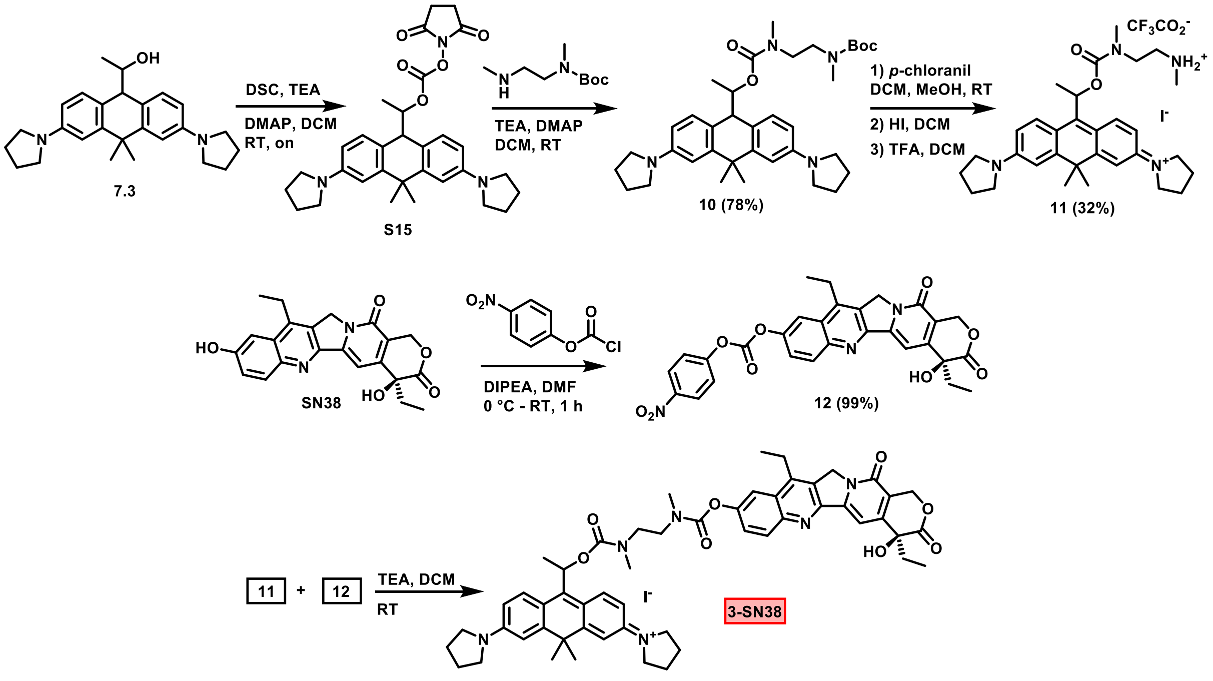

Scheme S7 Synthesis of ***3-SN38***

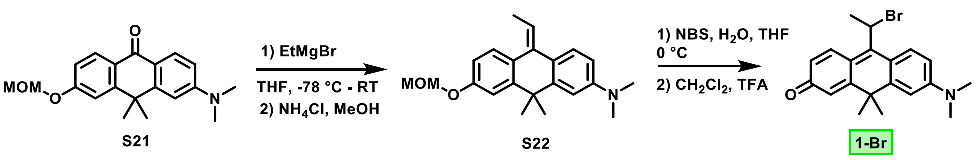

Scheme S8 Synthesis of ***1'-Br***

2.2. Initial Attempts to Access 9-Hydroxymethyl Derivatives

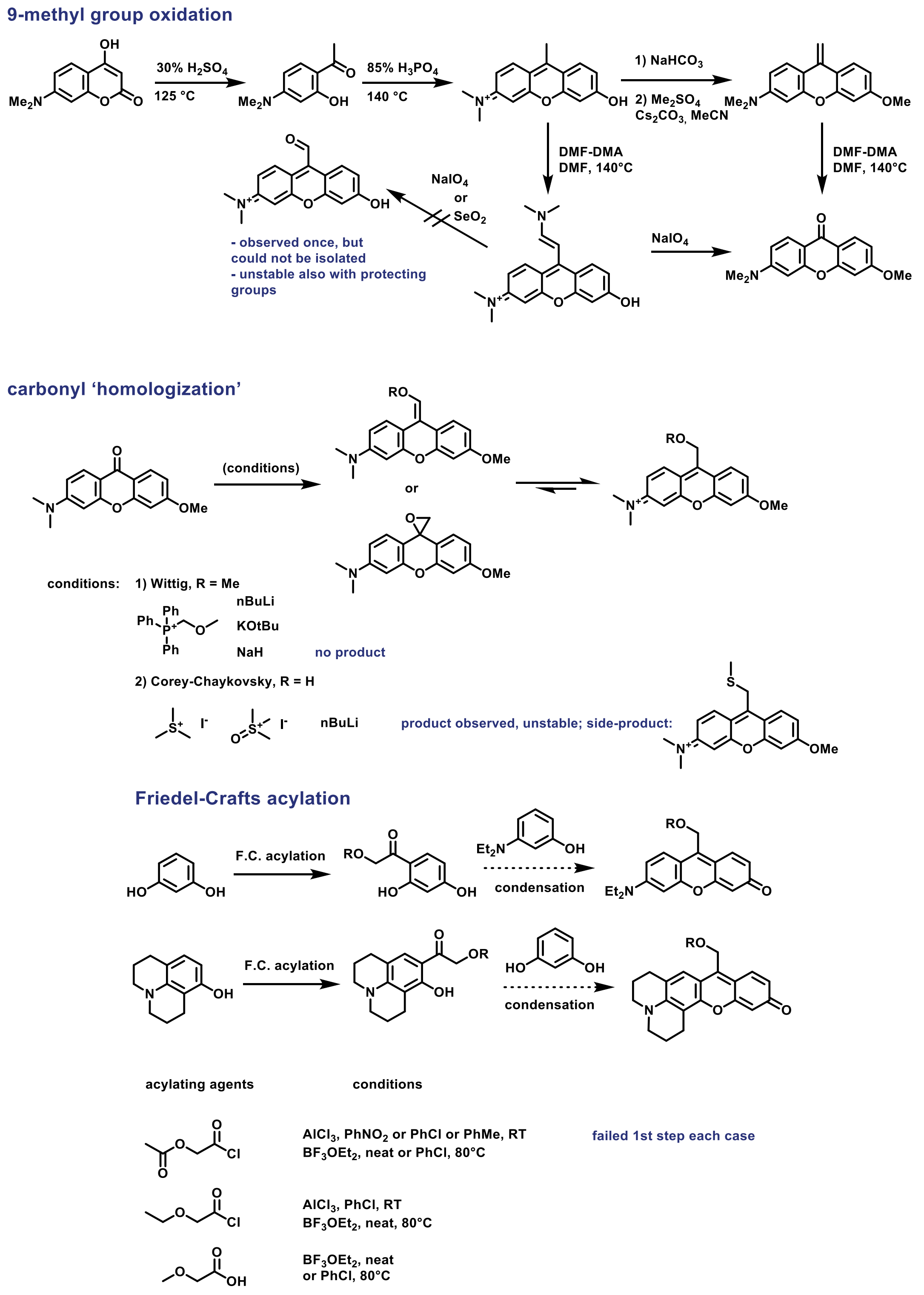

Scheme S9 Attempts for the synthesis of 9-hydroxymethyl rhodol pt. 1

bromoacetophenone path

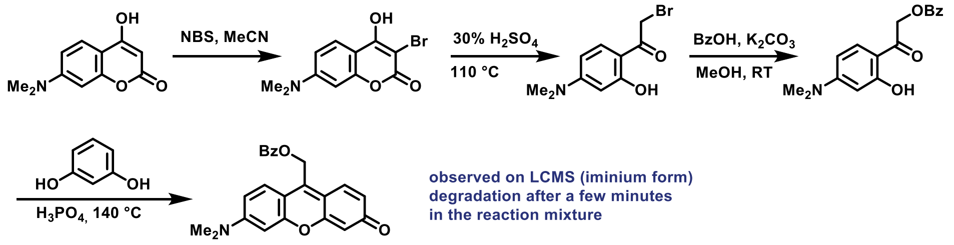

organometallic + hydroboration-oxidation

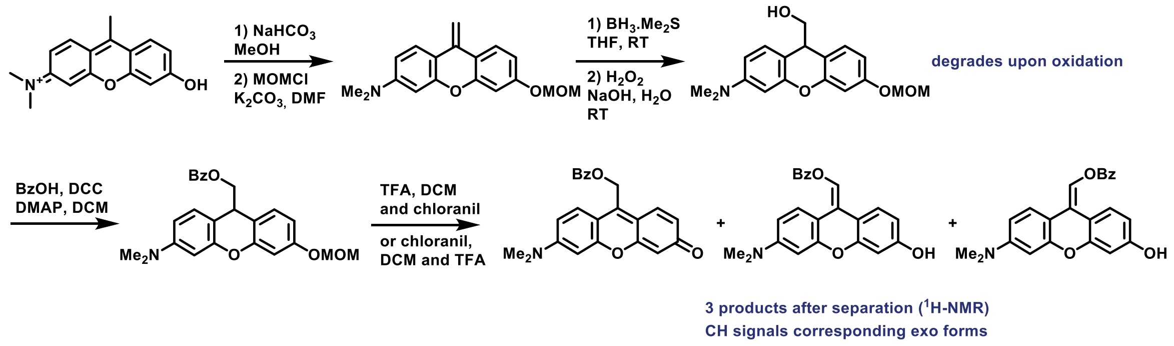

Scheme S10 Attempts for the synthesis of 9-hydroxymethyl rhodol pt. 2

2.3. Synthesis of the Starting Material Xanthones

Compound **S3** [1], **S11** [2,3] and pyrrolidine-substituted xanthones [4] were synthesized according to reported procedures.

*2.3.1. 3-Hydroxy-6-(pyrrolidin-1-yl)-9H-xanthen-9-one (**S4**) [4]*

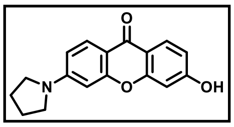

In a sealed tube 9-oxo-9H-xanthene-3,6-diyl-bis(trifluoromethanesulfonate) (**S3**, 2 g, 4.06 mmol, 1 equiv.) was dissolved in dry THF and freshly distilled pyrrolidine (1.66 mL, 20.3 mmol, 5 equiv.) was added under N_2 atmosphere. The reaction was stirred for 16 hours at 90 °C. After cooling to room temperature $N^+(CH_3)_4\ ^-OH$ (10 % in water, 1.85 g, 20.3 mmol, 5 equiv.) was added and stirred for 10 minutes. Then, the solvents were evaporated and the crude product was purified by flash chromatography (SiO_2, DCM : MeOH 0 % to 20 %).

Yield for the 2 steps: 484 mg, 1.72 mmol, 42 %.

^{1}H NMR (500 MHz, DMSO-*d*$_6$) δ 10.70 (s, 1H), 7.94 (d, *J* = 8.6 Hz, 1H), 7.89 (d, *J* = 8.8 Hz, 1H), 6.81 (dd, *J* = 8.7, 2.3 Hz, 1H), 6.77 (d, *J* = 2.2 Hz, 1H), 6.63 (dd, *J* = 8.9, 2.3 Hz, 1H), 6.40 (d, *J* = 2.3 Hz, 1H), 3.36 (d, *J* = 5.8 Hz, 4H), 2.02 – 1.95 (m, 4H).

^{13}C NMR (126 MHz, DMSO-*d*$_6$) δ 173.3, 162.9, 157.6, 157.3, 151.8, 127.4, 127.0, 114.2, 113.1, 110.2, 109.9, 101.9, 96.4, 47.44, 24.9.

HRMS: $[M+H]^+$: calcd for $[C_{17}H_{16}NO_3]^+$: 282.1052, found: 282.1120.

*2.3.2. 3-(Methoxymethoxy)-6-(pyrrolidin-1-yl)-9H-xanthen-9-one (**4.1**)*

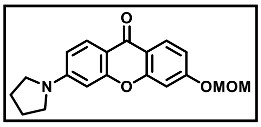

In a round-bottom flask 3-hydroxy-6-(pyrrolidin-1-yl)-9*H*-xanthen-9-one (**S4**, 286 mg, 1.016 mmol, 1 equiv.) was dissolved in dry DMF, K_2CO_3 (702 mg, 5.08 mmol, 5 equiv.) and MOMCl (386 µL, 5. 08 mmol, 5 equiv.) were added under N_2 atmosphere. The reaction was stirred at room temperature for 2 hours. Then, water was added, and the mixture was extracted with EtOAc (3 x 50 mL). The organic phase was dried over $MgSO_4$, filtered and evaporated. The crude product was purified by flash chromatography (Eluent: Hexane : EtOAc 0 % to 40 %).

Yield: 166 mg, 0.509 mmol, 50%.

^{1}H NMR (300 MHz, Chloroform-*d*) δ 8.22 (d, *J* = 8.8 Hz, 1H), 8.13 (d, *J* = 8.9 Hz, 1H), 7.03 (d, *J* = 2.3 Hz, 1H), 6.97 (dd, *J* = 8.8, 2.3 Hz, 1H), 6.58 (dd, *J* = 9.0, 2.3 Hz, 1H), 6.35 (d, *J* = 2.3 Hz, 1H), 5.27 (s, 2H), 3.51 (s, 3H), 3.46 – 3.36 (m, 4H), 2.15 – 2.02 (m, 4H).

^{13}C NMR (75 MHz, Chloroform-*d*) δ 175.4, 161.7, 158.6, 157.7, 152.3, 128.1, 128.1, 117.1, 113.3, 111.5, 110.0, 103.1, 96.7, 94.5, 56.5, 47.9, 25.6.

HRMS: $[M+H]^+$: calcd for $[C_{19}H_{20}NO_4]^+$: 326.1313, found: 326.1382.

*2.3.3. 3,6-Di(pyrrolidin-1-yl)-9H-xanthen-9-one (**4.2**) [4]*

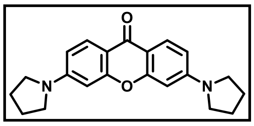

In a sealed tube 9-oxo-9H-xanthene-3,6-diyl bis(trifluoromethanesulfonate) (**S4**, 300 mg, 0.609 mmol, 1 equiv.) was dissolved in dry THF and freshly distilled pyrrolidine (500 µL, 6.09 mmol 10 equiv.) was added under N_2 atmosphere. The reaction was stirred for

16 hours at 90 °C. After cooling to room temperature, the solvent was evaporated, and the crude product was purified by flash chromatography (SiO_2, Eluent: Hexane: EtOAc 0 % to 100 %).
Yield: 170 mg, 0.508 mmol, 83 %.
^{1}H NMR (300 MHz, Chloroform-*d*) δ 8.08 (d, *J* = 8.8 Hz, 2H), 6.50 (d, *J* = 9.0 Hz, 2H), 6.29 (s, 2H), 3.42 – 3.27 (m, 8H), 2.10 – 1.95 (m, 8H).
^{13}C NMR (75 MHz, Chloroform-*d*) δ 175.3, 158.2, 151.8, 127.7, 111.7, 109.3, 96.7, 47.8, 25.5.
HRMS: $[M+H]^+$: calcd for $[C_{21}H_{23}N_2O_2]^+$: 335.1681, found: 335.1750.

*2.3.4. 10,10-Dimethyl-3,6-di(pyrrolidin-1-yl)anthracen-9(10H)-one (**4.3**) [4]*

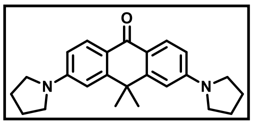

In a dried sealed tube 9,9-dimethyl-10-oxo-9,10-dihydroanthracene-2,7-diyl bis(trifluoromethanesulfonate) (**S11**, 1 g, 1.93 mmol, 1 equiv.) was dissolved in dry THF (10 mL) and freshly distilled pyrrolidine (1.59 mL, 19.3 mmol, 10 equiv.) was added under N_2 atmosphere. The reaction was stirred for 28 hours at 90 °C. After cooling to room temperature, the solvent was evaporated, and the crude product was purified by flash chromatography (SiO_2, Eluent: Hexane: EtOAc 0 % to 100 %)
Yield: 230 mg, 0.629 mmol, 33 %.
^{1}H NMR (300 MHz, Chloroform-*d*) δ 8.27 (d, *J* = 9.3 Hz, 2H), 6.68 – 6.56 (m, 4H), 3.48 – 3.38 (m, 8H), 2.10 – 2.01 (m, 8H), 1.71 (s, 6H).
^{13}C NMR (75 MHz, Chloroform-*d*) δ 152.6, 150.7, 129.5, 119.8, 111.0, 110.1, 107.7, 47.8, 38.2, 34.0, 25.7.
HRMS: $[M+H]^+$: calcd for $[C_{24}H_{29}N_2O]^+$: 361.2202, found: 361.2271.

2.4. Synthesis of the Photocage Precursors – Route A

*2.4.1. 1-(3-(Methoxymethoxy)-6-(pyrrolidin-1-yl)-9H-xanthen-9-yl)ethan-1-ol (**7.1**):*

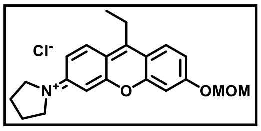

Grignard reaction (step 1): in a dried round-bottom flask 3-(methoxymethoxy)-6-(pyrrolidin-1-yl)-9*H*-xanthen-9-one (**4.1**) (150 mg, 0.46 mmol, 1 equiv.) in anhydrous THF (5 mL) was cooled to -78 °C under N_2 atmosphere and EtMgBr (1 M in THF, 4.6 mL, 4.6 mmol, 10 equiv.) was added dropwise. After stirring for 30 min, the cooling bath was removed, and the reaction was stirred for one more hour at room temperature. Then, saturated NH_4Cl (5 mL) was added dropwise, and the mixture was extracted with EtOAc 3 times (3 x 50 mL). The collected organic phases were washed with brine and dried over Mg_2SO_4. After the evaporation, the dark orange solid (compound **5.1**) was used immediately without purification.
The NMR of the crude product was taken in acetonitrile-d_3, in which the exo-form is present in a higher ratio (~90:10 exo-form : dye-form). The exo-form shows geometric isomerism, E and Z isomers are present.
Crude NMR, mixture of the E/Z isomers of the exo-form:
^{1}H NMR (500 MHz, Acetonitrile-d_3) δ 7.60 (d, *J* = 8.7 Hz, 1H), 7.57 (d, *J* = 8.7 Hz, 1H), 6.87 (dd, *J* = 8.6, 2.5 Hz, 1H), 6.84 (d, *J* = 2.5 Hz, 1H), 6.47 (dd, *J* = 8.7, 2.5 Hz, 1H), 6.34 (d, *J* = 2.5 Hz, 1H), 5.92 (q, *J* = 7.4 Hz, 1H), 5.29 (s, 2H), 3.56 (s, 3H), 3.38 – 3.30 (m, 4H), 2.16 (d, *J* = 7.5 Hz, 3H), 2.10 – 2.04 (m, 4H).

^{1}H NMR (500 MHz, Acetonitrile-d_3) δ 7.71 (d, *J* = 8.6 Hz, 1H), 7.44 (d, *J* = 8.7 Hz, 1H), 6.92 (dd, *J* = 8.6, 2.6 Hz, 1H), 6.90 (d, *J* = 2.5 Hz, 1H), 6.44 (dd, *J* = 8.7, 2.5 Hz, 1H), 6.28 (d, *J* = 2.5 Hz, 1H), 5.92 (q, *J* = 7.4 Hz, 1H), 5.32 (s, 2H), 3.57 (s, 3H), 3.38 – 3.30 (m, 4H), 2.16 (d, *J* = 7.5 Hz, 3H), 2.10 – 2.04 (m, 4H).
^{13}C NMR (126 MHz, Acetonitrile-d_3) δ 158.14, 158.13, 154.6, 154.5, 152.8, 152.7, 149.41, 149.37, 129.8, 129.7, 127.1, 127.0, 126.4, 125.2, 124.9, 120.7, 114.4, 113.7, 113.1, 111.8, 110.9, 109.6, 108.2, 104.8, 104.6, 99.3, 99.0, 95.4, 95.3, 56.5, 56.4, 48.4, 48.4, 30.7, 26.09, 26.07, 16.2, 16.1.
HRMS: $[M]^+$: calcd for $[C_{21}H_{24}NO_3]^+$: 338.1750, found: 338.1753.

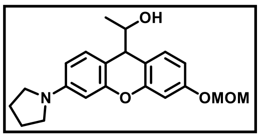

Hydroboration-oxidation (steps 2 & 3): the resulting ethyl-pyronine (compound **5.1**) was dissolved in anhydrous THF (5 mL) and NaH (74 mg, 1.84 mmol, 4 equiv.) was added to the solution under N_2 atmosphere. After 15 min, the yellow solution was filtered through a syringe filter into a deoxygenated round-bottom flask. The solution was cooled to 0 °C and borane-dimethyl sulfide comlex (426 µL, 4.6 mmol, 10 equiv.) was added dropwise. The reaction mixture was stirred overnight at room temperature. Then, the mixture was poured into methanol (15 mL) and 3 M NaOH (3 mL) and 30% H_2O_2 (3 mL) were added dropwise. After stirring for 30 min, the mixture was extracted with EtOAc 3 times (3 x 50 mL) and the combined organic phases were washed with $Na_2S_2O_3$ and brine, dried over Mg_2SO_4 and the solvents were evaporated. The crude product was purified by RP flash chromatography (eluent: water: MeCN 5% to 100%) to afford **7.1** as a colorless solid.
Yield for the 3 steps: 26 mg, 0.073 mmol, 16%.
Crude NMR, mixture of two diastereomers:
^{1}H NMR (500 MHz, Acetonitrile-d_3) δ 7.25 – 7.14 (m, 1H), 7.13 – 7.03 (m, 1H), 6.78 – 6.69 (m, 2H), 6.37 – 6.32 (m, 1H), 6.23 (d, *J* = 2.4 Hz, 1H), 5.17 (t, *J* = 2.4 Hz, 2H), 3.85 – 3.73 (m, 2H), 3.45 – 3.40 (m, 3H), 3.27 – 3.21 (m, 4H), 2.01 – 1.96 (m, 4H), 0.84 – 0.77 (m, 3H).
^{13}C NMR (126 MHz, Acetonitrile-d_3) δ 157.9, 157.8, 154.6, 154.4, 154.4, 154.1, 149.3, 149.3, 131.8, 131.4, 131.3, 130.9, 117.9, 117.2, 112.0, 111.9, 110.1, 109.7, 108.4, 104.8, 104.5, 99.5, 99.2, 95.5, 74.1, 74.1, 56.4, 48.5, 45.9, 45.7, 26.1, 18.9, 18.7.
HRMS: $[M+H]^+$: calcd for $[C_{21}H_{26}NO_4]^+$: 356.1856, found: 356.1850.

*2.4.2. 1-(3,6-Di(pyrrolidin-1-yl)-9H-xanthen-9-yl)ethan-1-ol (**7.2**):*

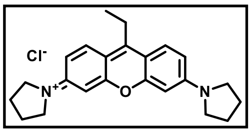

Grignard reaction (step 1): in a dried round-bottom flask 3,6-di(pyrrolidin-1-yl)-9*H*-xanthen-9-one (**4.2**) (300 mg, 0.897 mmol, 1 equiv.) in anhydrous THF (5 mL) was cooled to -78 °C under N_2 atmosphere and EtMgBr (1 M in THF, 8.97 mL, 8.97 mmol, 10 equiv.) was added dropwise. After stirring for 30 min, the cooling bath was removed, and the reaction was stirred for one more hour at room temperature. Then, saturated NH_4Cl (5 mL) was added dropwise and the mixture was extracted with EtOAc 3 times (3 x 50 mL). The collected organic phases were washed with brine and dried over Mg_2SO_4. After the evaporation, the dark pink solid (compound **5.2**) was used immediately without purification.
Crude NMR; dye-form:
^{1}H NMR (500 MHz, Acetonitrile-d_3; *d*-TFA) δ 7.90 (d, *J* = 9.4 Hz, 2H), 6.88 (dd, *J* = 9.4, 2.4 Hz, 2H), 6.44 (d, *J* = 2.3 Hz, 2H), 3.53 – 3.49 (m, 8H), 3.25 (q, *J* = 7.7 Hz, 2H), 2.10 – 2.06 (m, 8H), 1.32 (t, *J* = 7.7 Hz, 3H).

^{13}C NMR (126 MHz, Acetonitrile-d_3; *d*-TFA) δ 163.6, 158.2, 155.5, 130.2, 115.9, 113.4, 97.3, 49.8, 25.9, 22.2, 16.1.

HRMS: $[M]^+$: calcd for $[C_{23}H_{27}N_2O]^+$: 347.2117, found: 347.2112.

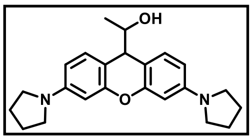

Hydroboration-oxidation (steps 2 & 3): the resulting ethyl-pyronine (compound **5.2**) was dissolved in anhydrous THF (5 mL) and NaH (86 mg, 3.58 mmol, 4 equiv.) was added to the solution under N_2 atmosphere. After 15 min, the yellow solution was filtered through a syringe filter into a deoxygenated round-bottom flask. The solution was cooled to 0 °C and borane-dimethyl sulfide comlex (850 µL, 8.97 mmol, 10 equiv.) was added dropwise. The reaction mixture was stirred overnight at room temperature. Then, the mixture was poured into methanol (20 mL) and 3 M NaOH (5 mL) and 30% H_2O_2 (5 mL) were added dropwise. After stirring for 30 min, the mixture was extracted with EtOAc 3 times (3 x 50 mL) and the combined organic phases were washed with $Na_2S_2O_3$ and brine, dried over Mg_2SO_4 and the solvents were evaporated to afford **7.2** as a colorless solid.

Yield for the 3 steps: 87 mg, 0.239 mmol, 27%.

Crude NMR:

^1H NMR (500 MHz, Chloroform-*d*) δ 7.15 – 7.02 (m, 2H), 6.36 – 6.28 (m, 4H), 3.84 – 3.73 (m, 2H), 3.27 – 3.21 (m, 8H), 2.01 – 1.95 (m, 8H), 0.97 (d, *J* = 5.9 Hz, 3H).

^{13}C NMR (126 MHz, Chloroform-*d*) δ 154.1, 153.8, 148.3, 148.2, 129.9, 129.7, 110.1, 109.2, 107.2, 107.1, 99.4, 99.2, 73.8, 47.9, 45.5, 25.6, 18.8. In the ^{13}C NMR NMR spectrum, all diastereotopic aromatic signals are doubled.

HRMS: $[M+H]^+$: calcd for $[C_{23}H_{29}N_2O_2]^+$: 365.2223, found: 365.2217.

2.4.3. 1-(10,10-Dimethyl-3,6-di(pyrrolidin-1-yl)-9,10-dihydroanthracen-9-yl)ethan-1-ol (7.3):

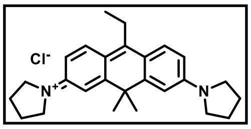

Grignard reaction (step 1): in a dried round-bottom flask 10,10-dimethyl-3,6-di(pyrrolidin-1-yl)anthracen-9(10*H*)-one (**4.3**) (50 mg, 0.139 mmol, 1 equiv.) in anhydrous THF (5 mL) was cooled to -78 °C under N_2 atmosphere and EtMgBr (1 M in THF, 1.39 mL, 1.390 mmol, 10 equiv.) was added dropwise. After stirring for 30 min, the cooling bath was removed, and the reaction was stirred for one more hour at room temperature. Then, saturated NH_4Cl (5 mL) was added dropwise and the mixture was extracted with EtOAc 3 times (3 x 50 mL). The collected organic phases were washed with brine and dried over Mg_2SO_4. After the evaporation, the dark blue solid (compound **5.3**) was used immediately without purification.

Crude NMR, two form present in 1:1 ratio (dye- and exo-form):

Dye-form:

^1H NMR (500 MHz, Acetonitrile-d_3; *d*-TFA) δ 8.06 (d, *J* = 9.4 Hz, 2H), 6.95 (d, *J* = 2.5 Hz, 2H), 6.80 (dd, *J* = 9.4, 2.5 Hz, 2H), 3.64 – 3.60 (m, 8H), 3.30 (q, *J* = 7.7 Hz, 2H), 2.10 – 2.05 (m, 8H), 1.58 (s, 6H), 1.35 (t, *J* = 7.7 Hz, 3H).

Exo-form:

^{1}H NMR (500 MHz, Acetonitrile-d_3; *d*-TFA) δ 7.79 (s, 1H), 7.76 (s, 1H), 7.66 (d, *J* = 8.2 Hz, 1H), 7.62 (d, *J* = 8.3 Hz, 1H), 7.51 (s, 1H), 7.50 (s, 1H), 6.37 (q, *J* = 7.3 Hz, 1H), 3.77 – 3.71 (m, 8H), 2.29 – 2.22 (m, 8H), 2.10 (d, *J* = 7.4 Hz, 3H), 1.66 (s, 6H).

^{13}C NMR (126 MHz, Acetonitrile-d_3; *d*-TFA) δ 170.2, 158.0, 154.7, 135.4, 130.4, 126.8, 126.4, 120.1, 114.8, 112.4, 68.4, 59.2, 49.8, 42.2, 34.6, 30.7, 28.1, 26.3, 25.9, 24.8, 24.7, 23.7, 17.1, 16.2. In the ^{13}C NMR spectrum, some signals of the two species overlap.

HRMS: $[M]^+$: calcd for $[C_{26}H_{33}N_2]^+$: 373.2638, found: 373.2621.

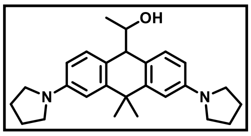

Hydroboration-oxidation (steps 2 & 3): the resulting ethyl-carbopyronine (compound **5.3**) was dissolved in anhydrous THF (5 mL) and NaH (16 mg, 0.417 mmol, 4 equiv.) was added to the solution under N_2 atmosphere. After 15 min, the yellow solution was filtered through a syringe filter into a deoxygenated round-bottom flask. The solution was cooled to 0 °C and borane-dimethyl sulfide comlex (132 µL, 1.39 mmol, 10 equiv.) was added dropwise. The reaction mixture was stirred overnight at room temperature. Then, the mixture was poured into methanol (15 mL) and 3 M NaOH (3 mL) and 30% H_2O_2 (3 mL) were added dropwise. After stirring for 30 min, the mixture was extracted with EtOAc 3 times (3 x 50 mL) and the combined organic phases were washed with $Na_2S_2O_3$ and brine, dried over Mg_2SO_4 and the solvents were evaporated. The crude product was purified by flash chromatography (eluent: hexane: EtOAc: 0 % to 30%) to afford **7.3** as a colorless solid.

Yield for the 3 steps: 10 mg, 0.026 mmol, 18%.

Crude NMR:

^{1}H NMR (500 MHz, Acetonitrile-d_3) δ 7.23 (d, *J* = 8.4 Hz, 1H), 7.14 (d, *J* = 8.3 Hz, 1H), 6.77 (dd, *J* = 5.4, 2.5 Hz, 2H), 6.55 – 6.50 (m, 2H), 3.85 – 3.77 (m, 2H), 3.35 – 3.32 (m, 8H), 2.19 (s, 1H), 2.06 – 2.02 (m, 8H), 1.71 (d, *J* = 7.2 Hz, 6H), 0.84 (d, *J* = 6.1 Hz, 3H).

^{13}C NMR (126 MHz, Acetonitrile-d_3) δ 148.19, 148.18, 146.5, 146.3, 131.0, 130.8, 123.1, 122.8, 111.03, 110.99, 110.5, 110.2, 75.2, 50.7, 48.6, 48.5, 39.7, 35.4, 33.8, 26.1, 20.3. In the ^{13}C NMR NMR spectrum, some diastereotopic signals are doubled and the double signals are underlined.

HRMS: $[M+H]^+$: calcd for $[C_{26}H_{35}N_2O]^+$: 391.2743, found: 391.2744.

2.5. Synthesis of the Photocage Precursors – Route B

2.5.1. *1-(3,6-di(pyrrolidin-1-yl)-9H-xanthen-9-yl)ethan-1-ol (**7.2**):*

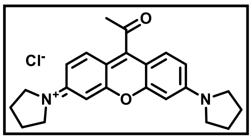

Umpolung acylation (step 1): in a previously dried round-bottom flask anhydrous THF and ethyl-vinyl ether (846 µL, 8.97 mmol, 15 equiv.) was cooled to -78 °C under N_2 atmosphere and tert-butyllithium (1.7 M in pentane, 2.46 mL, 4.186 mmol, 7.5 equiv.) was added dropwise. The resulting yellow solution was stirred for 20 min at -78 °C, then warmed up to 0 °C in an ice-water bath and kept at this temperature for 5 min. Next, the reaction mixture was cooled back down to -78 °C, then the suspension of 3,6-di(pyrrolidin-1-yl)-9*H*-xanthen-9-one (**4.2**) (200 mg, 0.598 mmol, 1 equiv.) in dry THF was added dropwise. The orange solution was stirred for 20 min at -78 °C, then the cooling bath was removed, and the reaction mixture was allowed to warm to room temperature and stirred for one more hour. After completion, the reaction was quenched with saturated NH_4Cl (5 mL), then poured into 50

mL methanol. With the dropwise addition of cc. HCl (2.5 mL) the orange solution turned dark pink and was stirred for 16 hours at room temperature (hydrolysis step). The reaction was monitored using LC-MS and after the completion of the hydrolysis, water was added and the mixture was extracted with CH_2Cl_2 5 times (5 x 50 mL), the combined organic layers were washed with brine and dried over Mg_2SO_4. After the evaporation of the volatiles, the dark pink solid (compound **6.2**) was used without further purification. Yield: 236 mg, 0.595 mmol, 99%.

Crude NMR:

^{1}H NMR (500 MHz, Acetonitrile-*d*$_3$; *d*-TFA) δ 7.47 (d, *J* = 9.3 Hz, 2H), 6.95 (dd, *J* = 9.3, 2.3 Hz, 2H), 6.67 (d, *J* = 2.3 Hz, 2H), 3.61 – 3.58 (m, 8H), 2.71 (s, 3H), 2.11 – 2.08 (m, 8H).

^{13}C NMR (126 MHz, Acetonitrile-*d*$_3$; *d*-TFA) δ 203.0, 158.5, 158.2, 156.0, 130.5, 116.9, 114.7, 110.0, 98.2, 50.2, 25.8.

HRMS: [M]$^+$: calcd for $[C_{23}H_{25}N_2O_2]^+$: 361.1910, found: 361.1906.

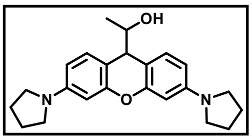

Double reduction (steps 2 & 3): the crude acetyl-pyronine compound (**6.2**) (236 mg, 0.595 mmol, 1 equiv.) was dissolved in ethanol (30 mL) and $NaBH_4$ (225 mg, 5.95 mmol, 10 equiv.) was added to the solution. The reaction was stirred for one hour at room temperature until the LC-MS indicated complete conversion. 20 mL water was added to the mixture at 0 °C and it was subsequently extracted with CH_2Cl_2 3 times (3 x 50 mL). The combined organic layers were washed with water, brine and dried over Mg_2SO_4. After filtration and evaporation, the light pink solid (compound **7.2**) was used without any further purification. Yield: 200 mg, 0.549 mmol, 92%.

Crude NMR:

^{1}H NMR (500 MHz, Chloroform-*d*) δ 7.15 – 7.02 (m, 2H), 6.36 – 6.28 (m, 4H), 3.84 – 3.73 (m, 2H), 3.27 – 3.21 (m, 8H), 2.01 – 1.95 (m, 8H), 0.97 (d, *J* = 5.9 Hz, 3H).

^{13}C NMR (126 MHz, Chloroform-*d*) δ 154.1, 153.8, 148.3, 148.2, 129.9, 129.7, 110.1, 109.2, 107.2, 107.1, 99.4, 99.2, 73.8, 47.9, 45.5, 25.6, 18.8. In the ^{13}C NMR NMR spectrum, some diastereotopic signals are doubled.

HRMS: [M+H]$^+$: calcd for $[C_{23}H_{29}N_2O_2]^+$: 365.2223, found: 365.2217.

*2.5.2. 1-(10,10-Dimethyl-3,6-di(pyrrolidin-1-yl)-9,10-dihydroanthracen-9-yl)ethan-1-ol (**7.3**):*

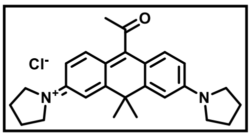

Umpolung acylation (step 1): in a previously dried round-bottom flask anhydrous THF and ethyl-vinyl ether (785 µL, 8.32 mmol, 15 equiv.) was cooled to -78 °C under N_2 atmosphere and tert-butyllithium (1.7 M in pentane, 2.45 mL, 4.16 mmol, 7.5 equiv.) was added dropwise. The resulting yellow solution was stirred for 20 min at -78 °C, then warmed up to 0 °C in an ice-water bath and kept at this temperature for 5 min. Next, the reaction mixture was cooled back down to -78 °C, the suspension of 10,10-dimethyl-3,6-di(pyrrolidin-1-yl)anthracen-9(10*H*)-one (**4.3**) (200 mg, 0.55 mmol, 1 equiv.) in dry THF was added dropwise. The orange solution was stirred for 20 min at -78 °C, then the cooling bath was removed, and the reaction mixture was allowed to warm to room temperature and stirred for one more hour. After completion, the reaction was quenched with saturated NH_4Cl (5 mL), then poured into 50 mL methanol. With the dropwise addition of cc. HCl (2.5 mL) the orange solution turned dark blue and was stirred for 16 hours at room temperature (hydrolysis step). The reaction was monitored using LC-MS and

after the completion of the hydrolysis, water was added and the mixture was extracted with CH_2Cl_2 5 times (5 x 100 mL), the combined organic layers were washed with brine and dried over Mg_2SO_4. After the evaporation of the volatiles, the dark blue solid (**6.3**) was used without further purification. Yield: 228 mg, 0.539 mmol, 98%.

Crude NMR:

^{1}H NMR (500 MHz, Acetonitrile-*d*$_3$; *d*-TFA) δ 7.38 (d, *J* = 9.2 Hz, 2H), 7.03 (d, *J* = 2.4 Hz, 2H), 6.78 (dd, *J* = 9.2, 2.4 Hz, 2H), 3.68 – 3.63 (m, 8H), 2.67 (s, 3H), 2.11 – 2.07 (m, 8H), 1.70 (s, 6H).

^{13}C NMR (126 MHz, Acetonitrile-*d*$_3$; *d*-TFA) δ 206.3, 161.6, 158.7, 158.1, 155.0, 135.7, 116.0, 115.5, 113.9, 50.2, 42.7, 33.6, 25.8.

HRMS: $[M]^+$: calcd for $[C_{26}H_{31}N_2O]^+$: 387.2430, found: 387.2421.

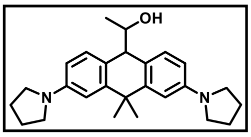

Hydroboration-oxidation (steps 2 & 3): the crude acetyl-pyronine compound (**6.3**) (228 mg, 0.539 mmol, 1 equiv.) was dissolved in ethanol (10 mL) and $NaBH_4$ (204 mg, 5.539 mmol, 10 equiv.) was added to the solution. The reaction was stirred for one hour at room temperature until the LC-MS indicated complete conversion. 10 mL water was added to the mixture at 0 °C and it was subsequently extracted with CH_2Cl_2 5 times (5 x 50 mL). The combined organic layers were washed with water, brine and dried over Mg_2SO_4. After filtration and evaporation, the blue solid (compound **7.3**) was used without any further purification.

Yield: 211 mg, 0.541 mmol, 98%.

Crude NMR:

^{1}H NMR (500 MHz, Acetonitrile-*d*$_3$) δ 7.23 (d, *J* = 8.4 Hz, 1H), 7.14 (d, *J* = 8.3 Hz, 1H), 6.77 (dd, *J* = 5.4, 2.5 Hz, 2H), 6.55 – 6.50 (m, 2H), 3.85 – 3.77 (m, 2H), 3.35 – 3.32 (m, 8H), 2.19 (s, 1H), 2.06 – 2.02 (m, 8H), 1.71 (d, *J* = 7.2 Hz, 6H), 0.84 (d, *J* = 6.1 Hz, 3H).

^{13}C NMR (126 MHz, Acetonitrile-*d*$_3$) δ 148.19, 148.18, 146.5, 146.3, 131.0, 130.8, 123.1, 122.8, 111.03, 110.99, 110.5, 110.2, 75.2, 50.7, 48.6, 48.5, 39.7, 35.4, 33.8, 26.1, 20.3. In the ^{13}C NMR NMR spectrum, some diastereotopic signals are doubled,

HRMS: $[M+H]^+$: calcd for $[C_{26}H_{35}N_2O]^+$: 391.2743, found: 391.2744.

2.6. Synthesis of Caged Model Compounds

2.6.1. 1-(3-(Methoxymethoxy)-6-(pyrrolidin-1-yl)-9H-xanthen-9-yl)ethyl 2-phenylacetate (*S12*)

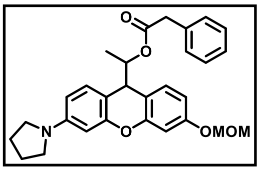

Hydroxyethyl derivative **7.1** (26 mg, 0.073 mmol, 1 equiv.) was dissolved in 1 ml CH_2Cl_2 then phenylacetic acid (15 mg, 0.11 mmol, 1.5 equiv.), DCC (18 mg, 0.087 mmol, 1.2 equiv.) and DMAP (catalytic amount) were added to the solution and the resulting mixture was stirred for 30 min. After completion, the reaction mixture was concentrated onto celite and purified by flash chromatography on silica (eluent: hexane–EtOAc, 0% to 10%) affording **S12** as a colorless oil. Yield: 12 mg, 0.027 mmol, 38%.

Mixture of two diastereomers (double integrals):

^{1}H NMR (500 MHz, Chloroform-*d*) δ 7.37 – 7.33 (m, 4H), 7.31 – 7.28 (m, 6H), 7.16 (d, J = 8.5 Hz, 1H), 7.10 (d, J = 8.4 Hz, 1H), 6.78 – 6.75 (m, 2H), 6.75 – 6.70 (m, 3H), 6.63 – 6.59 (m, 1H), 6.31 (dd, *J* = 8.4, 2.4 Hz,

1H), 6.27 – 6.20 (m, 3H), 5.18 – 5.15 (m, 4H), 5.07 – 4.99 (m, 2H), 4.05 (d, J = 3.9 Hz, 2H), 3.66 – 3.62 (m, 4H), 3.50 (s, 3H), 3.49 (s, 3H), 3.31 – 3.26 (m, 8H), 2.03 – 1.98 (m, 8H), 0.90 – 0.86 (m, 6H).

^{13}C NMR (126 MHz, Chloroform-*d*) δ 171.0, 157.0, 153.7, 153.5, 153.5, 153.4, 153.0, 152.2, 148.2, 137.1, 134.1, 134.1, 130.6, 130.3, 129.9, 129.7, 129.4, 128.6, 128.6, 127.1, 127.0, 115.7, 114.1, 111.2, 111.0, 108.0, 107.5, 107.4, 107.3, 106.7, 104.1, 103.8, 98.8, 98.6, 94.6, 76.3, 56.0, 47.7, 47.7, 42.0, 41.9, 41.9, 41.8, 36.6, 25.5, 25.5, 24.7, 23.3, 14.7, 14.4.

HRMS: $[M+H]^+$: calcd for $[C_{29}H_{32}NO_5]^+$: 474.2274, found: 474.2267.

*2.6.2. 1-(3-Oxo-6-(pyrrolidin-1-yl)-3H-xanthen-9-yl)ethyl 2-phenylacetate (**1-A**)*

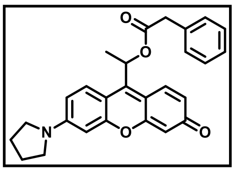

Deprotection (step 1): compound **S12** (35 mg, 0.074 mmol, 1 equiv.) was dissolved in CH_2Cl_2 (2 mL), and TFA (500 µL) was added and stirred at room temperature for 1 hour. After the completion of the deprotection, the volatiles were evaporated and the solid was thoroughly dried.

Oxidation (step 2): the resulting solid was dissolved in 1 mL CH_2Cl_2 – MeOH 1 : 1 and *p*-chloranil (36 mg, 0.148 mmol, 2 equiv.) was added to the solution in the dark. The mixture was stirred until the LC-MS indicated full conversion (30 min). Then, the reaction mixture was evaporated and applied directly to a silica gel column and purified by flash chromatography on silica (eluent: CH_2Cl_2 – MeOH 0% to 20%) affording **35** as a red crystalline solid.

Yield for the two steps*:* 20 mg, 0.048 mmol, 64%.

^{1}H NMR (500 MHz, Acetonitrile-*d$_3$*; *d*-TFA) δ 8.23 (d, J = 9.9 Hz, 1H), 8.18 (d, J = 9.9 Hz, 1H), 7.30 – 7.25 (m, 3H), 7.20 – 7.16 (m, 2H), 7.07 – 6.98 (m, 3H), 6.73 – 6.67 (m, 2H), 3.78 – 3.72 (m, 2H), 3.71 (s, 2H), 3.67 – 3.60 (m, 2H), 2.15 – 2.10 (m, 4H), 1.80 (d, J = 7.0 Hz, 3H).

^{13}C NMR (126 MHz, Acetonitrile-*d$_3$*; *d*-TFA) δ 171.6, 166.8, 159.6, 159.2, 157.9, 157.2, 134.6, 131.1, 130.9, 130.4, 129.6, 128.2, 119.4, 117.6, 115.8, 113.5, 103.7, 97.9, 69.5, 51.0, 50.8, 41.5, 25.9, 25.6, 21.4. In the ^{13}C NMR NMR spectrum, some diastereotopic signals are doubled and underlined.

HRMS: $[M+H]^+$: calcd for $[C_{27}H_{26}NO_4]^+$: 427.1784, found: 427.2747.

*2.6.3. 1-(3,6-Di(pyrrolidin-1-yl)-9H-xanthen-9-yl)ethyl 2-phenylacetate (**S13**)*

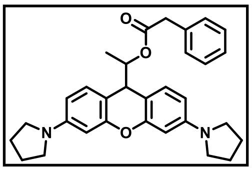

Hydroxyethyl derivative **7.2** (50 mg, 0.137 mmol, 1 equiv.) was dissolved in 2 mL CH_2Cl_2 then phenylacetic acid (28 mg, 0.205 mmol, 1.5 equiv.), DCC (34 mg, 0.165 mmol, 1.2 equiv.) and DMAP (catalytic amount) were added to the solution and the resulting mixture was stirred for 30 min. After completion, the reaction mixture was concentrated onto celite and purified by flash chromatography on silica (eluent: hexane–EtOAc, 0% to 10%) affording **S13** as a colorless oil.

Yield: 46 mg, 0.095 mmol, 70%.

^{1}H NMR (500 MHz, Chloroform-*d*) δ 7.38 – 7.28 (m, 5H), 7.12 (d, *J* = 8.3 Hz, 1H), 6.76 (d, *J* = 8.3 Hz, 1H), 6.30 (dd, *J* = 8.4, 2.4 Hz, 1H), 6.27 (d, *J* = 2.3 Hz, 2H), 6.23 (dd, *J* = 8.4, 2.5 Hz, 1H), 5.08 – 5.02 (m, 1H), 4.04 (d, *J* = 3.9 Hz, 1H), 3.66 (d, *J* = 3.0 Hz, 2H), 3.32 – 3.27 (m, 8H), 2.05 – 1.97 (m, 8H), 0.89 (d, *J* = 6.4 Hz, 3H).

^{13}C NMR (126 MHz, Chloroform-*d*) δ 171.1, 153.9, 153.5, 148.2, 134.4, 130.5, 129.8, 129.6, 129.3,128.7, 127.1, 109.0, 107.5, 107.3, 107.1, 98.9, 98.8, 76.8, 47.8, 42.1, 41.7, 25.6, 14.6. In the ^{13}C NMR spectrum, some diastereotopic signals are doubled.

HRMS: $[M+H]^+$: calcd for $[C_{31}H_{35}N_2O_3]^+$: 483.2642, found: 483.2624.

2.6.4. 1-(9-(1-(2-Phenylacetoxy)ethyl)-6-(pyrrolidin-1-yl)-3H-xanthen-3-ylidene)pyrrolidin-1-ium iodide (**2-A**)

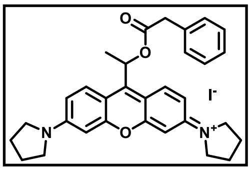

Compound **S13** (83 mg, 0.172 mmol, 1 equiv.) was dissolved in 2 mL CH_2Cl_2 – MeOH 1 : 1 and *p*-chloranil (170 mg, 0.688 mmol, 4 equiv.) was added to the solution. The mixture was stirred until the LC-MS indicated full conversion (30 min). Then, the reaction mixture was evaporated and applied directly to a silica gel column and purified by flash chromatography on silica (eluent: CH_2Cl_2 – MeOH 0% to 20%). The product was redissolved in CH_2Cl_2, one drop of HI (57% in water) was added, stirred for 5 min and another flash chromatography (on silica) was carried out (eluent: CH_2Cl_2 – MeOH 0% to 20 %) affording **2-A** as purple crystals.

Yield: 62 mg, 0.102 mmol, 59%.

^1H NMR (500 MHz, Acetonitrile-d_3) δ 8.08 (d, J = 9.6 Hz, 2H), 7.31 – 7.23 (m, 3H), 7.21 – 7.17 (m, 2H), 6.85 (dd, J = 9.5, 2.4 Hz, 2H), 6.64 (q, *J* = 7.0 Hz, 1H), 6.59 (d, J = 2.4 Hz, 2H), 3.70 (s, 2H), 3.65 – 3.50 (m, 8H), 2.11 – 2.07 (m, 8H), 1.78 (d, J = 7.0 Hz, 3H).

^{13}C NMR (126 MHz, Acetonitrile-d_3) δ 170.5, 157.6, 156.4, 154.4, 133.7, 129.4, 129.2, 128.6, 127.1, 115.2, 111.0, 96.6, 68.4, 48.9, 40.6, 24.9, 20.5.

HRMS: $[M]^+$: calcd for $[C_{31}H_{33}N_2O_3]^+$: 481.2486, found: 481.2493.

2.6.5. 1-(10,10-dimethyl-3,6-di(pyrrolidin-1-yl)-9,10-dihydroanthracen-9-yl)ethyl 2-phenylacetate (**S14**)

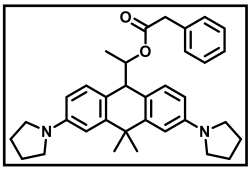

Hydroxyethyl derivative **7.3** (162 mg, 0.416 mmol, 1 equiv.) was dissolved in 5 mL CH_2Cl_2 then phenylacetic acid (85 mg, 0.624 mmol, 1.5 equiv.), DCC (103 mg, 0.499 mmol, 1.2 equiv.) and DMAP (catalytic amount) were added to the solution and the resulting mixture was stirred for 30 min. After completion, the reaction mixture was concentrated onto celite and purified by flash chromatography on silica (eluent: Hexane–EtOAc, 0% to 10%) affording **S14** as a colorless oil.

Yield: 60 mg, 0.118 mmol, 28%.

^1H NMR (500 MHz, Chloroform-*d*) δ 7.38 – 7.27 (m, 5H), 7.23 (d, J = 8.4 Hz, 1H), 6.94 (d, J = 8.4 Hz, 1H), 6.68 (d, J = 2.5 Hz, 1H), 6.65 (d, J = 2.5 Hz, 1H), 6.48 (dd, J = 8.4, 2.4 Hz, 1H), 6.41 (dd, J = 8.4, 2.5 Hz, 1H), 5.17 – 5.08 (m, 1H), 4.12 (d, J = 4.7 Hz, 1H), 3.67 (d, J = 4.3 Hz, 2H), 3.38 – 3.32 (m, 8H), 2.06 – 1.98 (m, 8H), 1.69 (d, J = 3.4 Hz, 7H), 0.80 (d, J = 6.4 Hz, 3H).

^{13}C NMR (126 MHz, Chloroform-*d*) δ 171.2, 147.0, 146.96, 145.9, 145.1, 134.5, 130.3, 130.0, 129.6, 128.7, 127.1, 121.5, 119.9, 110.6, 110.1, 109.4, 109.4, 77.9, 47.9, 46.4, 42.2, 35.1, 34.0, 25.6, 15.9.

HRMS: $[M+H]^+$: calcd for $[C_{34}H_{41}N_2O_2]^+$: 509.3162, found: 509.3154.

2.6.6. *1-(9,9-Dimethyl-10-(1-(2-phenylacetoxy)ethyl)-7-(pyrrolidin-1-yl)anthracen-2(9H)-ylidene)pyrrolidin-1-ium iodide (**3-A**)*

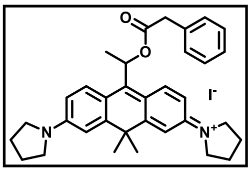

Compound **S14** (26 mg, 0.043 mmol, 1 equiv.) was dissolved in 1 ml CH_2Cl_2 – MeOH 1 : 1 and *p*-chloranil (21 mg, 0.086 mmol, 2 equiv.) was added to the solution. The mixture was stirred until the LC-MS indicated full conversion (30 min). Then, the reaction mixture was evaporated and applied directly to a silica gel column and purified by flash chromatography on silica (eluent: CH_2Cl_2 – MeOH 0% to 20%). The product was redissolved in CH_2Cl_2, one drop of HI (57% in water) was added, stirred for 5 min and another flash chromatography (on silica, eluent: CH_2Cl_2 – MeOH 0% to 20%) was carried out, obtaining the final compound as blue crystals with I^- counterion.

Yield: 24 mg, 0.038 mmol, 88%.

^{1}H NMR (500 MHz, Acetonitrile-*d*$_3$) δ 8.07 (d, J = 9.5 Hz, 2H), 7.26 – 7.20 (m, 3H), 7.13 (dd, J = 7.4, 2.1 Hz, 2H), 6.93 (d, J = 2.5 Hz, 2H), 6.63 (dd, J = 9.5, 2.5 Hz, 2H), 6.58 (q, *J* = 7.1 Hz, 1H), 3.67 – 3.61 (m, 8H), 2.11 – 2.07 (m, 8H), 1.82 (d, J = 7.1 Hz, 3H), 1.61 (s, 6H).

^{13}C NMR (126 MHz, Acetonitrile-*d*$_3$) δ 170.4, 162.1, 156.9, 153.2, 134.44, 134.43, 133.7, 129.2, 128.5, 127.0, 118.0, 113.5, 111.5, 69.9, 48.9, 41.7, 40.8, 24.8, 21.2.

HRMS: $[M]^+$: calcd for $[C_{34}H_{39}N_2O_2]^+$: 507.3006, found: 507.3006.

2.6.7. *1-(10,10-Dimethyl-3,6-di(pyrrolidin-1-yl)-9,10-dihydroanthracen-9-yl)ethyl (2,5-dioxopyrrolidin-1-yl) carbonate (**S15**):*

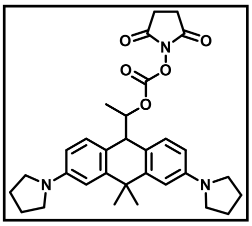

1-(10,10-Dimethyl-3,6-di(pyrrolidin-1-yl)-9,10-dihydroanthracen-9-yl)ethan-1-ol (**7.3**) (150 mg, 0.385 mmol, 1 equiv.) was dissolved in acetonitrile (7 mL), *N*,*N'*-disuccinimidyl carbonate (DSC, 493 mg, 1.92 mmol, 5 equiv.), TEA (536 µL, 3.85 mmol, 10 equiv.) and DMAP (cat.) were added to the solution and stirred for overnight at room temperature. After the LC-MS indicated complete conversion, the reaction was extracted 5 times with saturated $NaHCO_3$, the organic phase was dried over Mg_2SO_4, and the solvent was evaporated. The crude product was used immediately without further purification.

Yield: 192 mg, 0.362 mmol, 94%.

^{1}H NMR (500 MHz, Acetonitrile-*d*$_3$) δ 7.17 – 7.10 (m, 2H), 6.72 (dd, *J* = 9.7, 2.5 Hz, 2H), 6.54 – 6.49 (m, 2H), 4.99 – 4.93 (m, 1H), 4.22 (d, *J* = 4.7 Hz, 1H), 3.34 – 3.25 (m, 8H), 3.11 – 3.09 (m, 2H), 3.05 – 3.02 (m, 2H), 2.03 – 1.98 (m, 8H), 1.67 (d, *J* = 8.1 Hz, 6H), 0.93 (d, *J* = 6.4 Hz, 3H).

^{13}C NMR (126 MHz, Acetonitrile-*d*$_3$) δ 169.8, 167.3, 165.3, 154.4, 151.1, 147.6, 147.5, 146.0, 145.3, 130.0, 129.5, 119.6, 118.5, 110.7, 110.3, 109.5, 109.4, 85.5, 67.8, 47.5, 45.8, 38.6, 34.0, 33.2, 25.3, 25.3, 25.2, 19.8, 15.5, 13.3.

MS: $[M+H]^+$: calcd for $[C_{31}H_{38}N_3O_5]^+$: 532, found: 532.

2.6.8. *1-(10,10-Dimethyl-3,6-di(pyrrolidin-1-yl)-9,10-dihydroanthracen-9-yl)ethyl 4-phenylpiperazine-1-carboxylate (**S16**)*

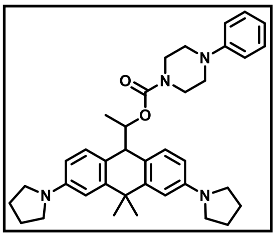

Compound **S15** (177 mg, 0.128 mmol, 1 equiv.) was dissolved in 5 mL CH_2Cl_2 (stabilized with amylene) then *N*-phenylpiperazine (30 µL, 0.192 mmol, 1.5 equiv.), TEA (53 µL, 0.384 mmol, 3 equiv.) and DMAP (15 mg, 0.128 mmol, 1 equiv.) were added and the was stirred for 1 hour at room temperature. After completion, the mixture was concentrated onto celite *in vacuo* and purified by flash chromatography on silica (eluent: hexane–EtOAc 0% to 10%) to afford **S16** as a colorless oil.

Yield: 31 mg, 0.054 mmol, 42%.

^{1}H NMR (500 MHz, Acetonitrile-d_3) δ 7.27 (dd, *J* = 8.8, 7.2 Hz, 2H), 7.19 (d, *J* = 8.4 Hz, 1H), 7.11 (d, *J* = 8.3 Hz, 1H), 7.00 – 6.95 (m, 2H), 6.89 – 6.83 (m, 1H), 6.73 (d, *J* = 2.5 Hz, 1H), 6.70 (d, *J* = 2.5 Hz, 1H), 6.52 – 6.46 (m, 2H), 4.93 – 4.83 (m, 1H), 4.09 (d, *J* = 5.6 Hz, 1H), 3.70 – 3.45 (m, 4H), 3.31 – 3.26 (m, 8H), 3.18 – 3.07 (m, 4H), 2.03 – 1.97 (m, 8H), 1.66 (d, *J* = 1.6 Hz, 6H), 0.79 (d, *J* = 6.5 Hz, 3H).

^{13}C NMR (126 MHz, Chloroform-*d*) δ 155.2, 151.5, 147.0, 147.0, 146.9, 146.0, 145.0, 130.4, 129.9, 129.4, 121.8, 120.5, 116.9, 116.9, 110.7, 110.2, 109.9, 109.5, 109.4, 78.4, 61.6, 49.7, 49.6, 47.9, 46.2, 38.8, 34.8, 34.5, 25.6, 25.6, 15.9, 14.8.

HRMS: $[M+H]^+$: calcd for $[C_{37}H_{47}N_4O_2]^+$: 579.3693, found: 579.3686.

2.6.9. *1-(9,9-Dimethyl-10-(1-((4-phenylpiperazine-1-carbonyl)oxy)ethyl)-7-(pyrrolidin-1-yl)anthracen-2(9H)-ylidene)pyrrolidin-1-ium iodide (**3-B**)*

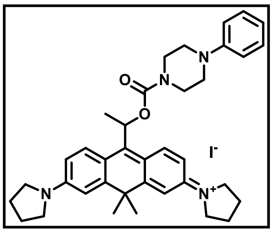

Compound **S16** (10 mg, 0.017 mmol, 1 equiv.) was dissolved in 1 mL CH_2Cl_2 – MeOH 1 : 1 in the dark and *p*-chloranil (17 mg, 0.068 mmol, 4 equiv.) was added to the solution. The mixture was stirred until the LC-MS indicated full conversion (30 min). The reaction mixture was evaporated and applied directly to a silica gel column and purified by flash chromatography on silica (eluent: CH_2Cl_2 – MeOH 0% to 20%). The product was redissolved in CH_2Cl_2 (1 mL), one drop of HI (57% in water) was added, the mixture was stirred for 5 min and another flash chromatography (on silica, eluent: CH_2Cl_2 – MeOH 0% to 20%) was carried out, obtaining the final compound as blue crystals with I^- counterion.

Yield: 7 mg, 0.010 mmol, 57%.

^{1}H NMR (500 MHz, Acetonitrile-d_3) δ 8.28 (d, *J* = 9.5 Hz, 2H), 7.27 – 7.23 (m, 2H), 6.97 (d, *J* = 2.5 Hz, 2H), 6.94 (d, *J* = 8.2 Hz, 2H), 6.86 (t, *J* = 7.3 Hz, 1H), 6.80 (dd, *J* = 9.6, 2.5 Hz, 2H), 6.56 (q, *J* = 7.1 Hz, 1H), 3.80 – 2.85 (m, 8 H and 4H), 2.10 – 2.07 (m, 8H), 1.87 (d, *J* = 7.1 Hz, 3H), 1.65 (s, 6H).

^{13}C NMR (126 MHz, Acetonitrile-d_3) δ 158.1, 154.8, 154.3, 135.7, 135.6, 130.6, 119.2, 114.7, 112.6, 71.5, 49.9, 42.8, 34.1, 30.4, 25.8, 22.5.

HRMS: $[M]^+$: calcd for $[C_{37}H_{45}N_4O_2]^+$: 577.3537, found: 577.3538.

*2.6.10. 1-(10,10-Dimethyl-3,6-di(pyrrolidin-1-yl)-9,10-dihydroanthracen-9-yl)ethyl phenyl carbonate (**S17**)*

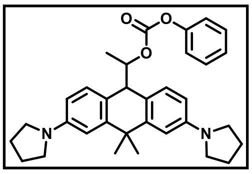

Crude compound **S15** (50 mg, 0.128 mmol, 1 equiv.) was dissolved in CH_2Cl_2 (3 mL) then phenol (18 mg, 0.192 mmol, 1.5 equiv.), triethylamine (53 uL, 0.384 mmol, 3 equiv.) and DMAP (16 mg, 0.128 mmol, 1 equiv.) were added and the reaction was stirred for 1 hour at room temperature. After completion, the reaction was concentrated onto celite and purified by flash chromatography on silica (eluent: hexane–EtOAc 0% to 10%) to afford **S17** as a colorless oil.

Yield: 32 mg, 0.063 mmol, 49%.

^{1}H NMR (500 MHz, Acetonitrile-d_3) δ 7.39 (dd, J = 8.5, 7.4 Hz, 2H), 7.28 – 7.24 (m, 1H), 7.18 (d, J = 8.3 Hz, 1H), 7.14 (d, J = 8.4 Hz, 1H), 7.11 – 7.07 (m, 2H), 6.73 (dd, J = 9.0, 2.5 Hz, 2H), 6.55 – 6.47 (m, 2H), 4.90 (q, J = 6.3 Hz, 1H), 4.11 (d, J = 5.7 Hz, 1H), 3.32 – 3.27 (m, 8H), 2.03 – 1.98 (m, 8H), 1.68 (s, 6H), 1.03 (d, J = 6.4 Hz, 3H).

^{13}C NMR (126 MHz, Acetonitrile-d_3) δ 154.0, 152.3, 148.5, 148.4, 147.0, 146.4, 131.1, 130.8, 130.4, 127.0, 122.4, 121.6, 120.7, 111.2, 111.1, 110.4, 110.3, 82.5, 48.0, 39.7, 35.2, 33.5, 26.2, 17.4.

HRMS: $[M+H]^+$: calcd for $[C_{33}H_{39}N_2O_3]^+$: 511.2955, found: 511.2951.

*2.6.11. 1-(9,9-Dimethyl-10-(1-((phenoxycarbonyl)oxy)ethyl)-7-(pyrrolidin-1-yl)anthracen-2(9H)-ylidene)pyrrolidin-1-ium iodide (**3-C**)*

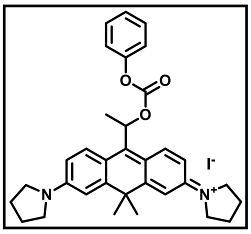

Compound **S17** (32 mg, 0.063 mmol, 1 equiv.) was dissolved in 1 mL CH_2Cl_2 – MeOH 1 : 1 in the dark and *p*-chloranil (64 mg, 0.251 mmol, 4 equiv.) was added to the solution. The mixture was stirred until the LC-MS indicated full conversion (30 min). The reaction mixture was evaporated and applied directly to a silica gel column and purified by flash chromatography on silica (eluent: CH_2Cl_2 – MeOH 0% to 20%).

The product was redissolved in CH_2Cl_2, one drop of HI (57% in water) was added, the mixture was stirred for 5 min and another flash chromatography (on silica, eluent: CH_2Cl_2 – MeOH 0% to 20%) was carried out, obtaining the final compound as blue crystals with I^- counterion.

Yield: 34 mg, 0.0534 mmol, 89%.

^{1}H NMR (500 MHz, Acetonitrile-d_3) δ 8.21 (d, J = 9.5 Hz, 2H), 7.38 (t, J = 7.9 Hz, 2H), 7.27 (t, J = 7.4 Hz, 1H), 7.12 – 7.05 (m, 2H), 7.00 (d, J = 2.5 Hz, 2H), 6.82 (dd, J = 9.5, 2.5 Hz, 2H), 6.64 (q, J = 7.0 Hz, 1H), 3.72 – 3.62 (m, 8H), 2.11 – 2.06 (m, 8H), 1.95 (d, J = 1.8 Hz, 3H), 1.66 (s, 6H).

^{13}C NMR (126 MHz, Acetonitrile-d_3) δ 160.3, 157.1, 153.4, 152.6, 151.0, 134.5, 129.7, 126.4, 121.2, 120.9, 118.1, 113.9, 111.9, 73.7, 49.0, 41.9, 24.8, 21.4.

HRMS: $[M]^+$: calcd for $[C_{33}H_{37}N_2O_3]^+$: 509.2799, found: 509.2802.

2.7. Synthesis of Photocaged SN38 Derivatives

2.7.1. *Boc-protected linker-SN38 (**S19**) [5]*

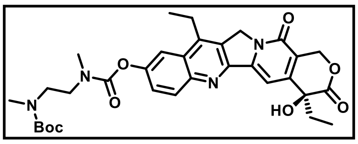

SN-38 (177 mg, 0.45 mmol, 1 equiv.) was dissolved in dry DMF, then compound **46** (230 mg, 0.54 mmol, 1.2 equiv.)[36], triethylamine (136 µL, 1.35 mmol, 3 equiv.) and DMAP (2 mg, 0.035 mmol, 0.03 equiv.) was added under N_2 atmosphere. The reaction was stirred at 80 °C for 2 hours. The solvent was evaporated, concentrated onto silica gel and purified by flash chromatography on silica (eluent: CH_2Cl_2 – MeOH 0% to 20%).

Yield: 400 mg, 0.356 mmol, 79%.

^{1}H NMR (500 MHz, DMSO-*d*$_6$) δ 8.19 (d, *J* = 9.1 Hz, 1H), 7.93 (m, 1H), 7.63 (d, *J* = 9.3 Hz, 1H), 7.34 (s, 1H), 6.50 (s, 1H), 5.44 (s, 2H), 5.35 (s, 2H), 3.63 (t, *J* = 5.1 Hz, 1H), 3.52 – 3.44 (m, 3H), 3.23 – 3.14 (m, 4H), 3.00 (s, 2H), 2.85 (d, *J* = 10.4 Hz, 2H), 1.93 – 1.82 (m, 2H), 1.44 (s, 3H), 1.39 (s, 6H), 1.33 – 1.29 (m, 3H), 0.89 (t, *J* = 7.3 Hz, 3H).

MS: $[M+H]^+$: calcd for $[C_{28}H_{32}N_3O_6]^+$: 607, found: 607.

2.7.2. *1-(3,6-Di(pyrrolidin-1-yl)-9H-xanthen-9-yl)ethyl (2,5-dioxopyrrolidin-1-yl) carbonate (**9**)*

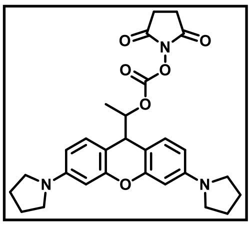

Compound **7.2** 100 mg, 0.274 mmol, 1 equiv.) was dissolved in acetonitrile (5 mL), DSC (352 mg, 1.37 mmol, 5 equiv.), TEA (382 µL, 2.74 mmol, 10 equiv.) and DMAP (cat.) were added to the solution and stirred for overnight at room temperature. After the LC-MS indicated complete conversion, the reaction was extracted 5 times with saturated $NaHCO_3$, the organic phase was dried over Mg_2SO_4, and the solvent was evaporated. The crude product was used immediately without further purification and characterization as the long-term stability of the succinimidyl carbonates are poor.

Yield: 136 mg, 0.270 mmol, 99%.

MS: $[M+H]^+$: calcd for $[C_{28}H_{32}N_3O_6]^+$: 506, found: 506.

2.7.3. *Reduced 2-SN38 conjugate (**S20**)*

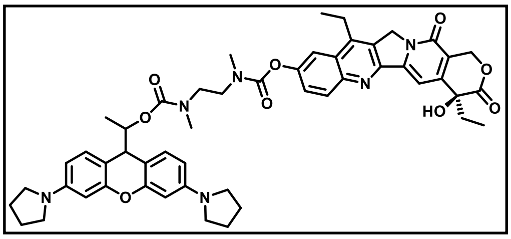

SN38-Boc-protected self-immolative linker (**S19**) (166 mg, 0.274 mmol, 1 equiv.) was dissolved in CH_2Cl_2 and TFA (25% for the mixture) was added and stirred at room temperature for 1 hour. After the completion of the deprotection, the volatiles were evaporated and the solid was thoroughly dried.

The succinimidyl carbonate (**9**) (0.274 mmol, 1 equiv.) was dissolved in CH_2Cl_2, compound **8**, and triethylamine (114 µL, 0.822 mmol, 3 equiv.) was added, and stirred for 1 hour at room temperature. The reaction was concentrated onto silica gel and purified by flash chromatography on silica (Eluent: CH_2Cl_2 – MeOH 0% to 20%). The compound was dissolved in acetonitrile and purified once more by reverse-phase chromatography (eluent: water – acetonitrile 5% to 100%). The compound appeared to be air sensitive resulting in rapid oxidation of the product, therefore was used in the next step without further characterization.

Yield: 127 mg, 0.142 mmol, 52%.

MS: $[M+H]^+$: calcd for $[C_{51}H_{57}N_6O_9]^+$: 897, found: 897.

2.7.4. Conjugate **2-SN38**

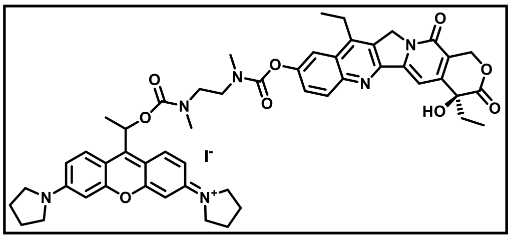

The reduced photocage (**S20**) (30 mg, 0.034 mmol, 1 equiv.) was dissolved in CH_2Cl_2 – MeOH 1-1 mL) and *p*-chloranil (17 mg, 0.067 mmol, 2 equiv.) was added to the solution. The mixture was stirred until the LC-MS indicated full conversion (30 min). The reaction was evaporated and applied directly to a silica gel column and purified by flash chromatography on silica (Eluent: CH_2Cl_2 – MeOH 0% to 20%).

The product was redissolved in CH_2Cl_2, one drop of HI (57% in water) was added, stirred for 5 min and another flash chromatography (on silica) was carried out, obtaining the final compound with I^- counterion (eluent: CH_2Cl_2 – MeOH 0% to 20%).

Yield: 18 mg, 0.018 mmol, 52%.

^{1}H NMR (500 MHz, Acetonitrile-*d*$_3$) δ 8.43 – 8.02 (m, 5H), 7.57 – 7.49 (m, 1H), 7.01 – 6.90 (m, 2H), 6.72 – 6.51 (m, 1H), 6.13 (s, 1H), 5.72 – 5.64 (m, 1H), 5.51 – 5.30 (m, 3H), 3.65 – 3.46 (m, 11H), 3.33 – 3.14 (m, 4H), 3.08 (d, *J* = 2.7 Hz, 2H), 3.02 (s, 2H), 2.96 – 2.89 (m, 2H), 2.17 – 2.07 (m, 11H), 1.90 – 1.81 (m, 4H), 1.42 – 1.29 (m, 4H), 1.24 – 1.17 (m, 2H), 1.09 – 1.00 (m, 4H).

HRMS: $[M]^+$: calcd for $[C_{51}H_{55}N_6O_9]^+$: 895.4025, found: 895.4028.

2.7.5. Tert-butyl (1-(10,10-dimethyl-3,6-di(pyrrolidin-1-yl)-9,10-dihydroanthracen-9-yl)ethyl) ethane-1,2-diylbis(methylcarbamate) (**10**)

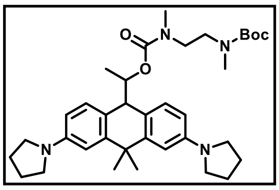

Compound **S15** (100 mg, 0,256 mmol, 1 equiv.) was dissolved in CH_2Cl_2 (3 mL) then *tert*-butyl methyl(2-(methylamino)ethyl)carbamate (145 mg, 0.768 mmol, 3 equiv.), triethylamine (53 µL, 0.384 mmol, 1.5 equiv.) were added and the reaction was stirred for 1 hour at room temperature. After completion, the reaction was concentrated onto celite and purified by flash chromatography on silica (eluent: hexane –EtOAc 0% to 10%) to afford **10** as a colorless oil.

Yield: 120 mg, 0.199 mmol, 78%.

^{1}H NMR (500 MHz, Chloroform-*d*) δ 7.32 (t, *J* = 9.4 Hz, 1H), 7.18 – 7.09 (m, 1H), 6.67 (dd, *J* = 22.7, 2.5 Hz, 2H), 6.56 – 6.45 (m, 2H), 5.06 (s, 1H), 4.34 – 4.23 (m, 1H), 3.32 (dd, *J* = 8.4, 5.1 Hz, 8H), 3.06 – 2.96 (m, 4H), 2.96 – 2.81 (m, 6H), 2.02 (m, 8H), 1.70 (d, *J* = 11.2 Hz, 6H), 1.46 (s, 9H), 0.72 (d, *J* = 6.4 Hz, 3H).

^{13}C NMR (126 MHz, Chloroform-*d*) δ 146.8, 146.7, 144.7, 129.78, 119.6, 110.6, 109.8, 109.2, 60.4, 47.7, 45.8, 38.5, 34.5, 28.4, 28.4, 25.5, 21.0, 15.5, 14.2.

HRMS: $[M+H]^+$: calcd for $[C_{36}H_{53}N_4O_4]^+$: 605.4061, found: 605.4052.

2.7.6. Compound **11** (oxidation and deprotection)

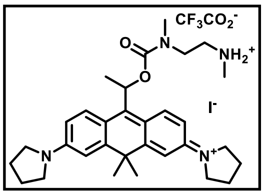

Step 1 (oxidation): Boc-protected compound **10** (120 mg, 0.198 mmol, 1 equiv.) was dissolved in CH_2Cl_2 – MeOH -1 : 1 (3 mL), and chloranil (152 mg,0.596 mmol, 3 equiv.) was added to the solution. After 30 min, the solvent was evaporated, the dark blue solid

was applied directly on silica gel column and purified by flash chromatography (eluent: CH_2Cl_2 – MeOH 0% to 20%).

Step 2 (anion exchange): the purified product was redissolved in CH_2Cl_2 (2 mL), one drop of HI (57% in water) was added, stirred for 5 min and another flash chromatography (on silica) was carried out (eluent: CH_2Cl_2 – MeOH 0% to 20%).

Step 3 (deprotection): Boc-protected iodide salt was dissolved in CH_2Cl_2 (1 mL), then trifluoroacetic acid (25% for the mixture) was added and stirred at room temperature for 1 hour. Then volatiles were evaporated and the solid was thoroughly dried. The blue product was used without further purification in the next step.

Yield for the 3 steps: 32%.

^{1}H NMR (500 MHz, Acetonitrile-*d*$_3$) δ 8.24 (d, *J* = 9.5 Hz, 2H), 6.98 (d, *J* = 2.4 Hz, 2H), 6.80 (dd, *J* = 9.5, 2.5 Hz, 2H), 6.59 (d, *J* = 7.5 Hz, 1H), 3.67 – 3.63 (m, 8H), 3.14 (dd, *J* = 10.5, 6.8 Hz, 2H), 2.99 (s, 3H), 2.61 (s, 3H), 2.27 (t, *J* = 7.4 Hz, 2H), 2.12 – 2.08 (m, 8H), 1.88 (d, *J* = 7.0 Hz, 3H), 1.65 (s, 6H).

^{13}C NMR (126 MHz, Acetonitrile-*d*$_3$, *d*-TFA) δ 163.2, 158.1, 154.2, 135.8, 119.58, 119.57, 115.0, 112.99, 112.97, 72.0, 55.3, 50.3, 46.7, 42.8, 34.4, 34.3, 25.9, 22.9.

HRMS: [M]$^+$: calcd for $[C_{31}H_{43}N_4O_2]^+$: 503.3380, found: 503.3377.

2.7.7. Activation of SN38 with 4-nitrophenyl chloroformate (**12**)

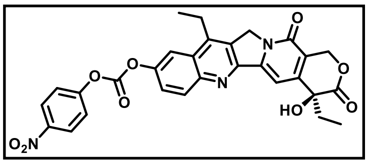

Compound **12** was synthesized according to [5].

2.7.8. Conjugate **3-SN38**

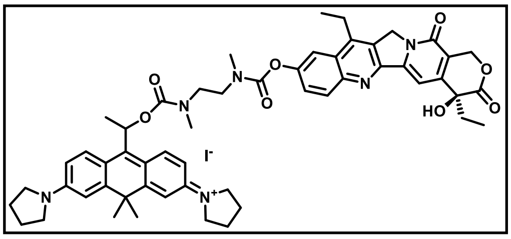

Compound **12** (112 mg, 0.198 mmol, 1 equiv.) was dissolved in dry DMF, then crude compound **11** (120 mg, 0.198 mmol, 1 equiv.), and DIPEA (69 µL, 0.396 mmol, 2 equiv.) were added and the reaction mixture was stirred for 30 min at room temperature. After the LC-MS indicated full conversion, the solvent was evaporated, the reaction was concentrated onto silica gel and purified by flash chromatography on silica gel (Eluent: CH_2Cl_2 – MeOH 0% to 20 %) to obtain compound **3-SN38** as a blue solid.

Yield: 107 mg, 0,116 mmol, 58%.

^{1}H NMR (500 MHz, Acetonitrile-*d*$_3$) δ 8.35 – 7.93 (m, 3H), 7.88 – 7.47 (m, 2H), 6.95 – 6.76 (m, 2H), 6.78 – 6.50 (m, 3H), 5.61 (d, J = 16.6 Hz, 1H), 5.38 (d, J = 16.6 Hz, 1H), 5.37 – 5.29 (m, 2H), 3.64 – 3.48 (m, 11H), 3.30 – 3.06 (m, 5H), 3.03 (s, 1H), 2.97 – 2.76 (m, 3H), 2.10 – 2.01 (m, 9H), 1.88 – 1.77 (m, 3H), 1.66 – 1.51 (m, 4H), 1.46 (d, J = 5.3 Hz, 2H), 1.38 – 1.21 (m, 5H), 1.02 – 0.93 (m, 3H).

HRMS: [M]$^+$: calcd for $[C_{54}H_{61}N_6O_8]^+$: 921.4545, found: 921.4551.

2.8. Synthesis of **1'-Br**

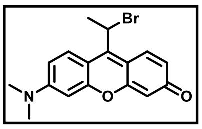

In a dried round-bottom flask 3,6-di(pyrrolidin-1-yl)-9*H*-xanthen-9-one (50 mg, 0.167 mmol, 1 equiv.) in anhydrous THF (5 mL) was cooled to -78 °C under N_2 atmosphere and EtMgBr (1 M in THF, 1.67 mL, 1.67 mmol, 10 equiv.) was added dropwise. After stirring for 30 min, the cooling bath was removed, and the reaction was stirred for one more hour at room temperature. Then, saturated NH_4Cl (5 mL) was added dropwise, and the mixture was extracted with EtOAc 3 times (3 x 50 mL). The collected organic phases were washed with brine and dried over Mg_2SO_4. After the evaporation, the dark pink solid was dissolved in anhydrous THF (3 mL) and KO^{t}Bu (1 M in THF, 35 µL, 0.033 mmol, 0.2 equiv.) was added to the solution. The colour changed to light pink, then distilled water (1 mL) was added, and the reaction was cooled to 0 °C in ice-water bath. Then *N*-bromosuccinimide (33 mg, 0.184 mmol, 1.1 equiv.) was added in dark, for which the reaction turned dark purple. After 15 min stirring at room temperature, the mixture was extracted with CH_2Cl_2 and water. The organic phase was dried over Mg_2SO_4, and the solvent was evaporated.

The crude product was redissolved in CH_2Cl_2 (1 ml) and TFA (200 µL) was added dropwise. After 30 min stirring, the solvents were evaporated. The crude product was purified by flash chromatography (eluent: CH_2Cl_2 – MeOH 0% to 20%) to obtain **23** as dark red crystals. The long-term stability of the bromo derivative was poor.

Yield for 3 steps:18 mg, 0.0517 mmol, 31%.

The NMR of the isolated product is a mixture of protonated (iminium) and deprotonated species (see the spectra below) in a ratio of approx. 3 to 1. Due to the stability issues, only 1H-NMR data and LCMS chromatograms are provided.

Iminium-form:

^{1}H NMR (500 MHz, Acetonitrile-*d*$_3$; *d*-TFA) δ 8.52 (br s, 1H), 8.36 (br s, 1H), 7.36 (dd, *J* = 9.9, 2.6 Hz, 1H), 7.18 (dd, *J* = 9.3, 2.5 Hz, 1H), 7.11 (d, *J* = 2.5 Hz, 1H), 6.89 (d, *J* = 2.6 Hz, 1H), 6.24 (q, *J* = 7.1 Hz, 1H), 3.37 (s, 6H), 2.24 (d, *J* = 7.1 Hz, 3H).

Oxo-form:

^{1}H NMR (500 MHz, Acetonitrile-*d*$_3$; *d*-TFA) δ 7.87 (d, *J* = 8.7 Hz, 1H), 7.64 (d, *J* = 2.3 Hz, 1H), 7.56 – 7.47 (m, 2H), 6.85 (d, *J* = 2.4 Hz, 1H), 6.73 (dd, *J* = 8.7, 2.4 Hz, 1H), 4.46 (q, *J* = 6.8 Hz, 1H), 3.20 (s, 6H), 1.56 (d, *J* = 6.8 Hz, 3H).

3. Spectroscopic Properties of the Compounds

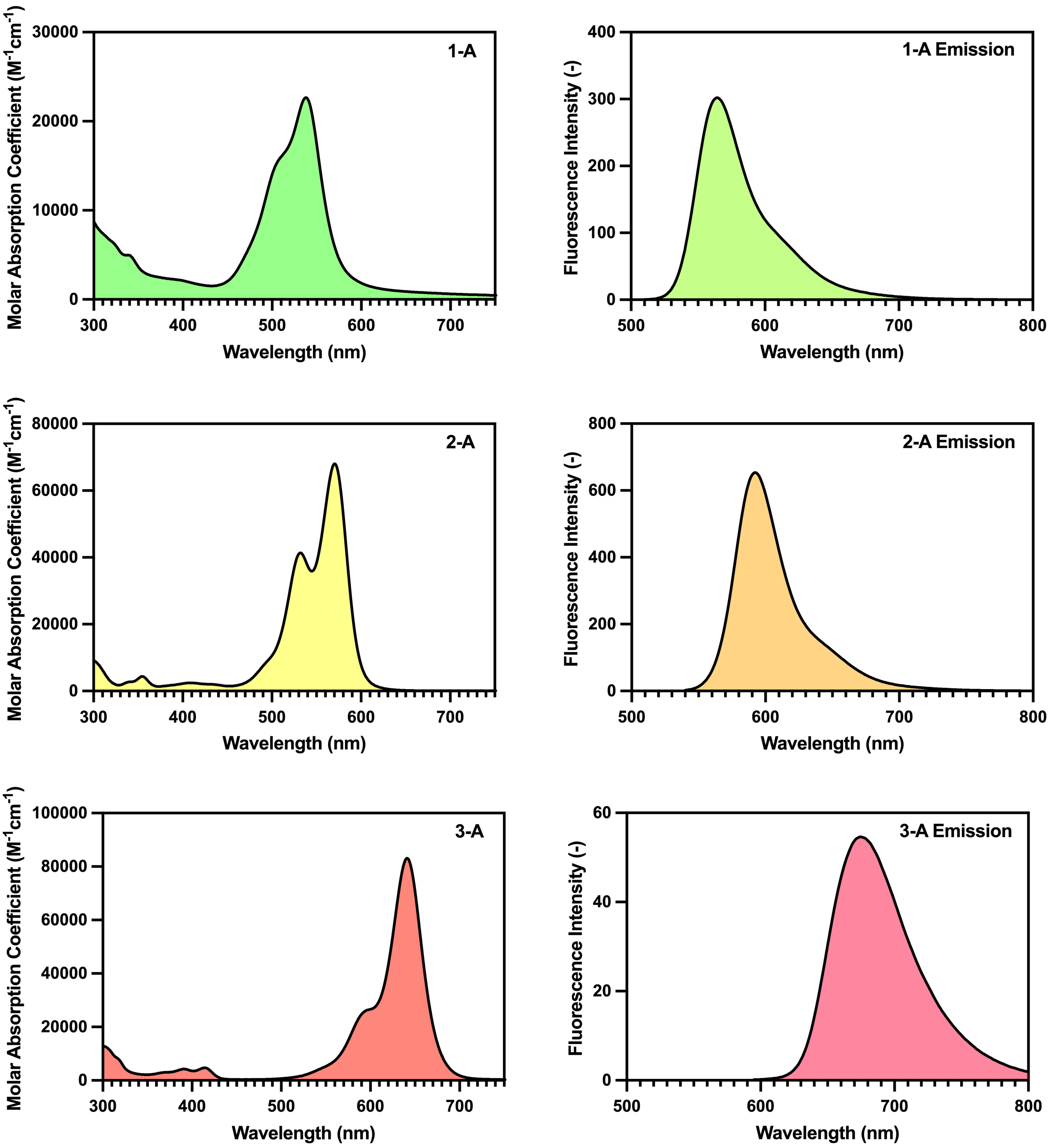

*Figure S1 Absorption and emission spectra of compounds **1-A**, **2-A** and **3-A** in PBS. Excitation wavelength: 500 nm (**1-A),** 530 nm (**2-A**), 585 nm (**3-A).***

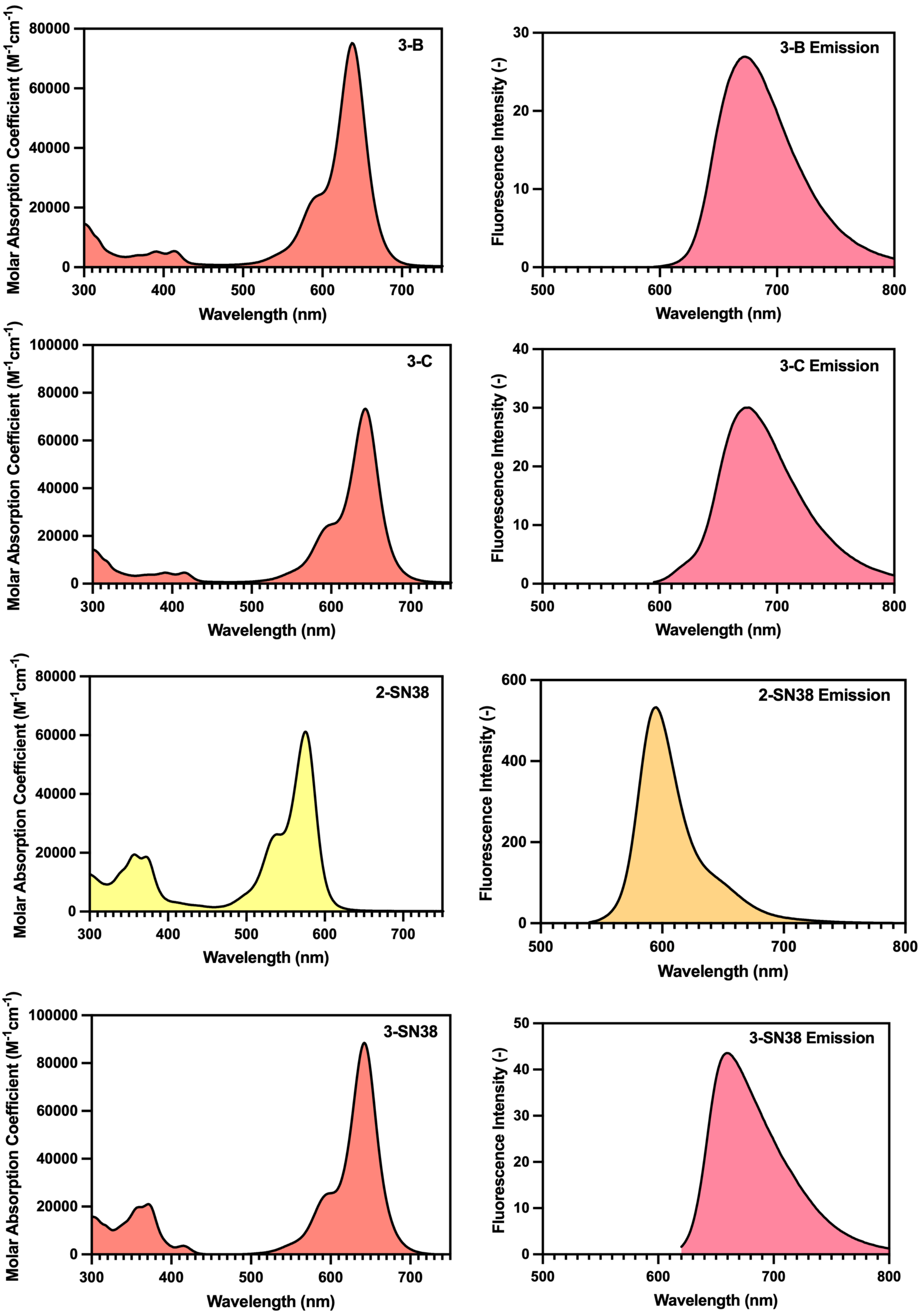

*Figure S2 Absorption and emission spectra of compounds **3-B**, **3-C**, **2-SN38** and **3-SN38** in PBS. Excitation wavelength: 585 nm (**3-B**), 585 nm (**3-C**), 530 nm (**2-SN38**), 610 nm (**3-SN38**).*

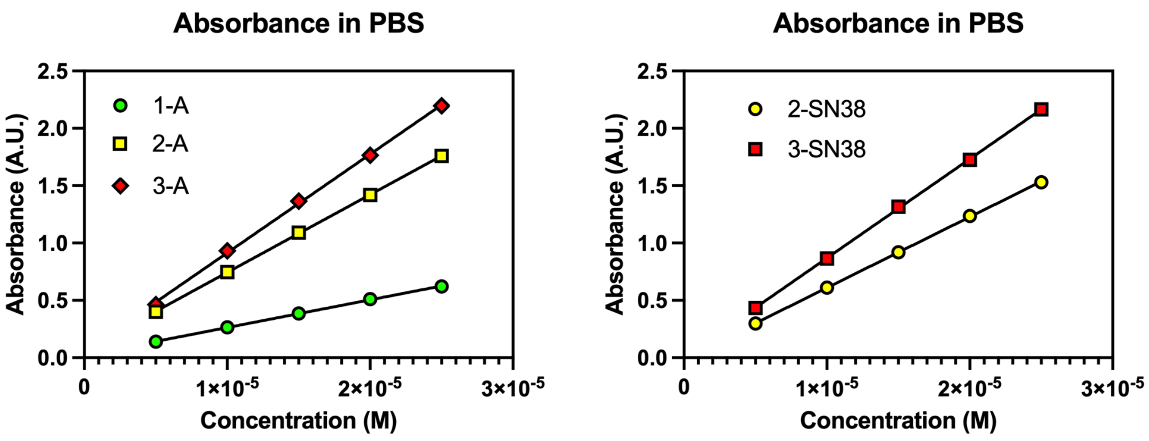

Figure S3 Concentration-dependent absorbance of the compounds in PBS (<2% MeCN)

4. Uncaging Studies

4.1. Experimental details

The uncaging experiments were performed using custom-made LED panels.

The uncaging experiments were performed using custom-built LED panels with commercial light sources with the following specifications per color: green: 4W input power, λ_{max} = 549 nm half-width: 16 nm, output power: 72 mW; orange: 4W input power, λ_{max} = 605 nm half-width: 10 nm, output power: 140 mW and red: 4W input power, λ_{max} = 658 nm half-width: 10 nm, output power: 210 mW. See Fig S5 for the photos and Fig S4 for the spectra. Sample solutions were prepared, each containing 1 ml of solvent (90% v/v water-MeCN or 90% PBS-MeCN or 100% methanol for the photochemical quantum yield determination; optical path length: 14 mm) and 0.1 mM or 0.05 mM concentration of the compounds. The samples were irradiated for a given time using continuous water cooling for the light source and carefully avoiding overheating. Then, the samples were transferred to the HPLC-UV-MS system and the chromatograms were recorded. The traces at 195 nm, 254 nm or 369 nm (dependent on the payload) and at the absorption maxima of the photoremovable protecting groups (530 nm, 570 nm, 640 nm) were compared and the corresponding peaks were integrated and compared to reference calibration sets. The possible photoproducts were identified by their corresponding m/z value and also from the UV/VIS spectra from the DAD-equipped instrument. For the stability determination, the HPLC sample solutions were kept in the dark and chromatograms were recorded multiple times. We note that for compound **3-C**, 50% input power setting was used for the green LED. The linearity of input power vs. intensity (determined using a fluorimeter) is shown on Fig S4. Output power was measured using a Coherent FieldMate Laser Power meter with an 1000:1 attenuator (OP-2 VIS).

For the photochemical quantum yield determination, refer to section 4.3.

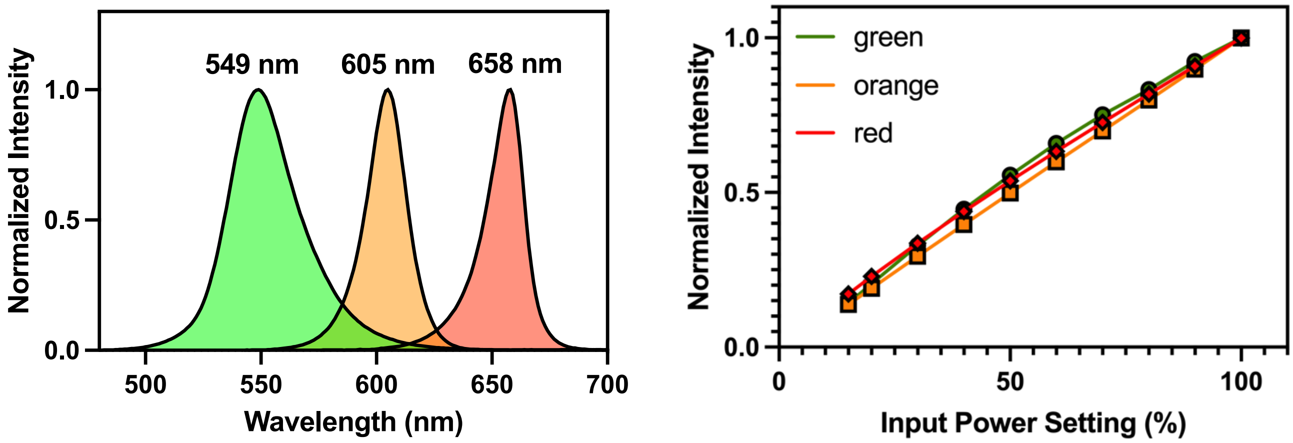

Figure S4 Normalized emission spectra of the LEDs used in this work (left), the corresponding emission maxima are also shown and the normalized intensity output vs. input power setting of the LEDs (right)

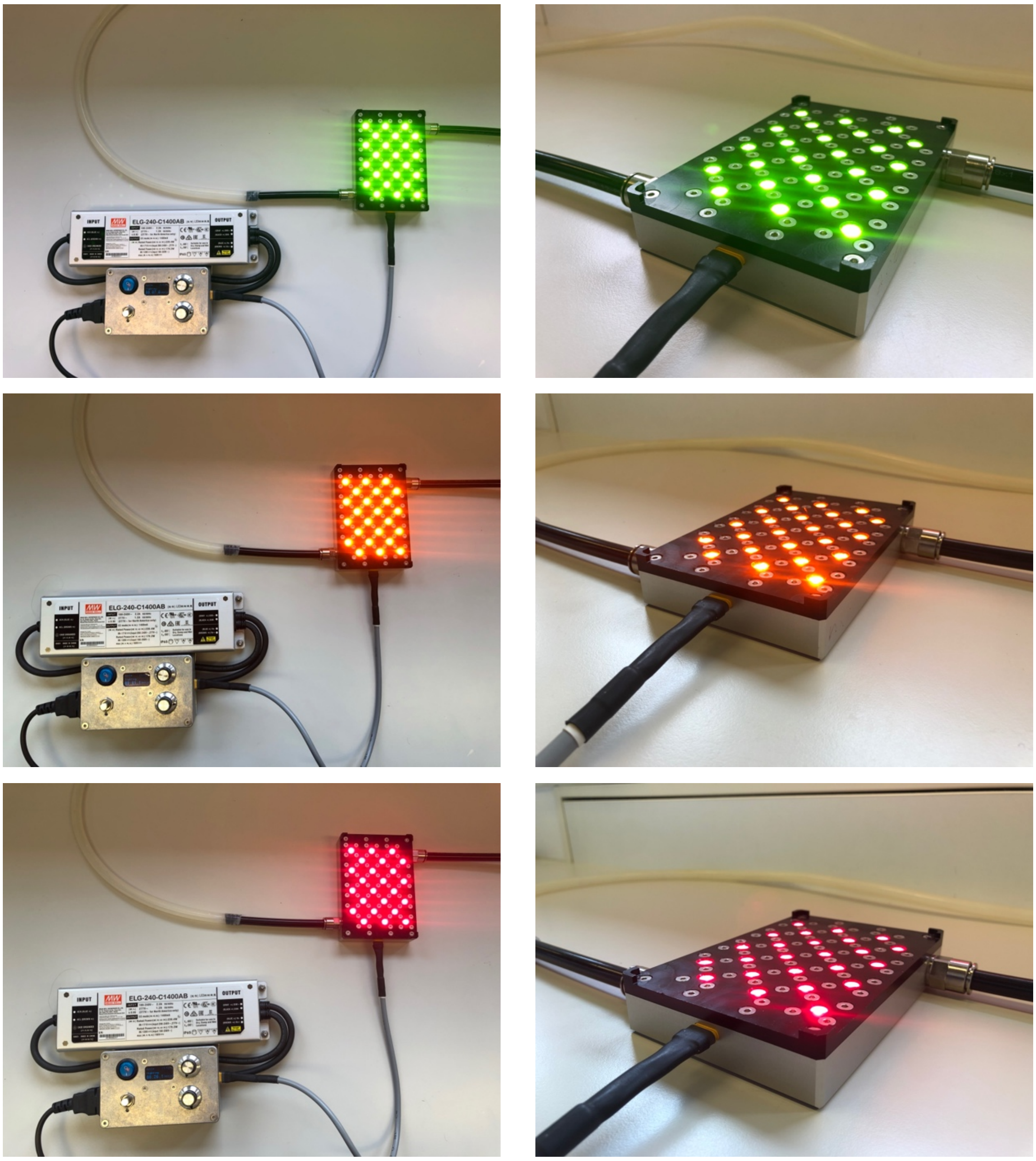

Figure S5 Photographs of the custom-built LED panels

4.2. HPLC Chromatograms of the Uncaging Experiments

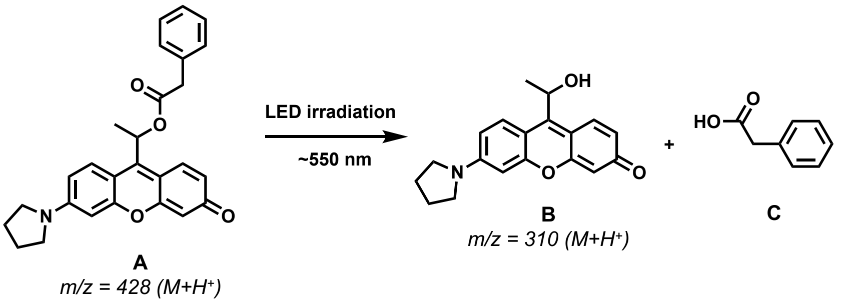

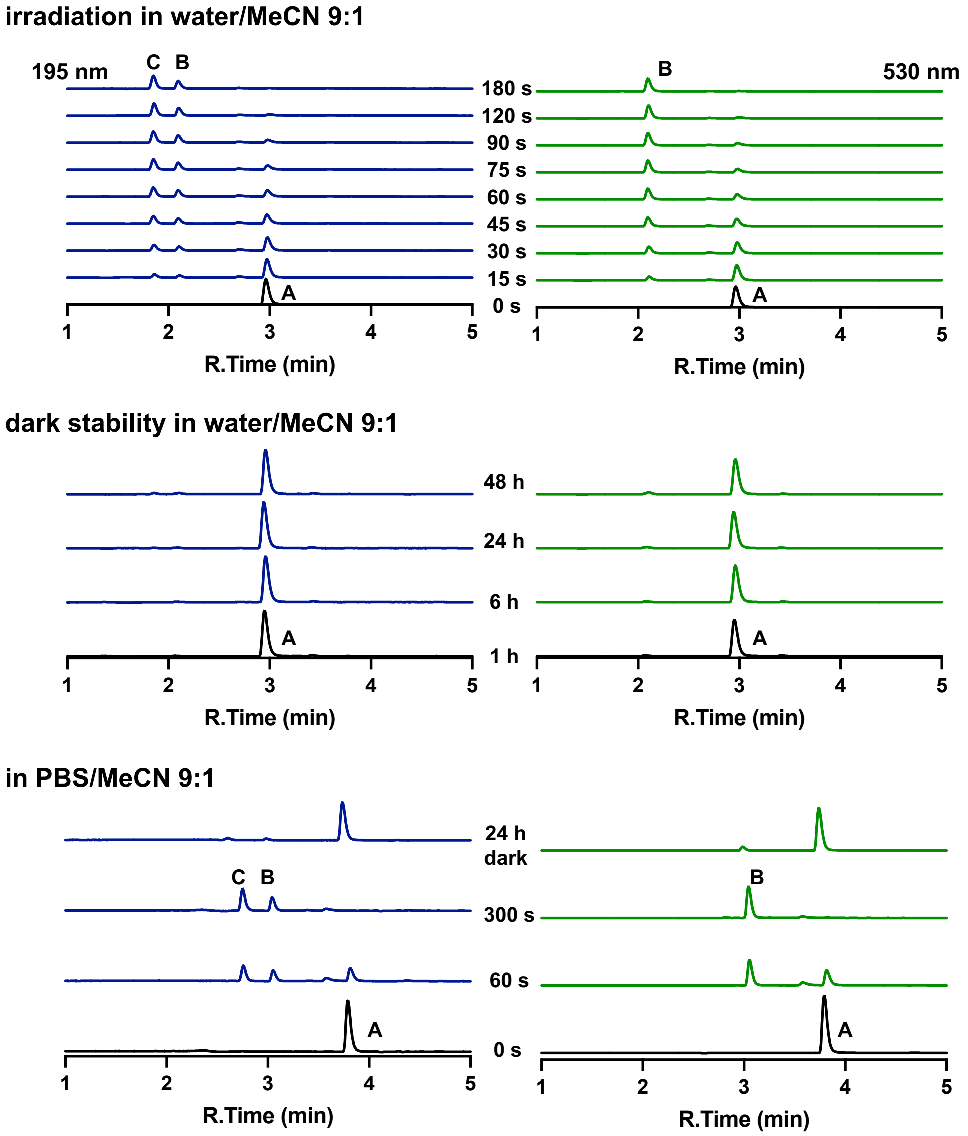

*Figure S6 Partial chromatograms of the green light uncaging and stability experiments of **1-A** as followed by HPLC-UV-MS instrument. The release of phenylacetic acid (C) was monitored at 195 nm, the disappearance of the starting material (A) was monitored at 530 nm.*

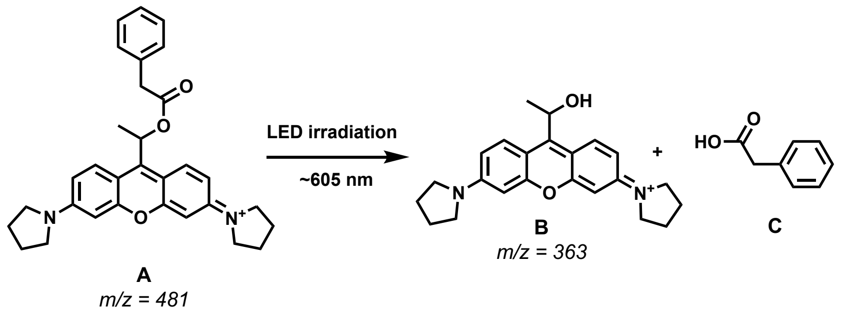

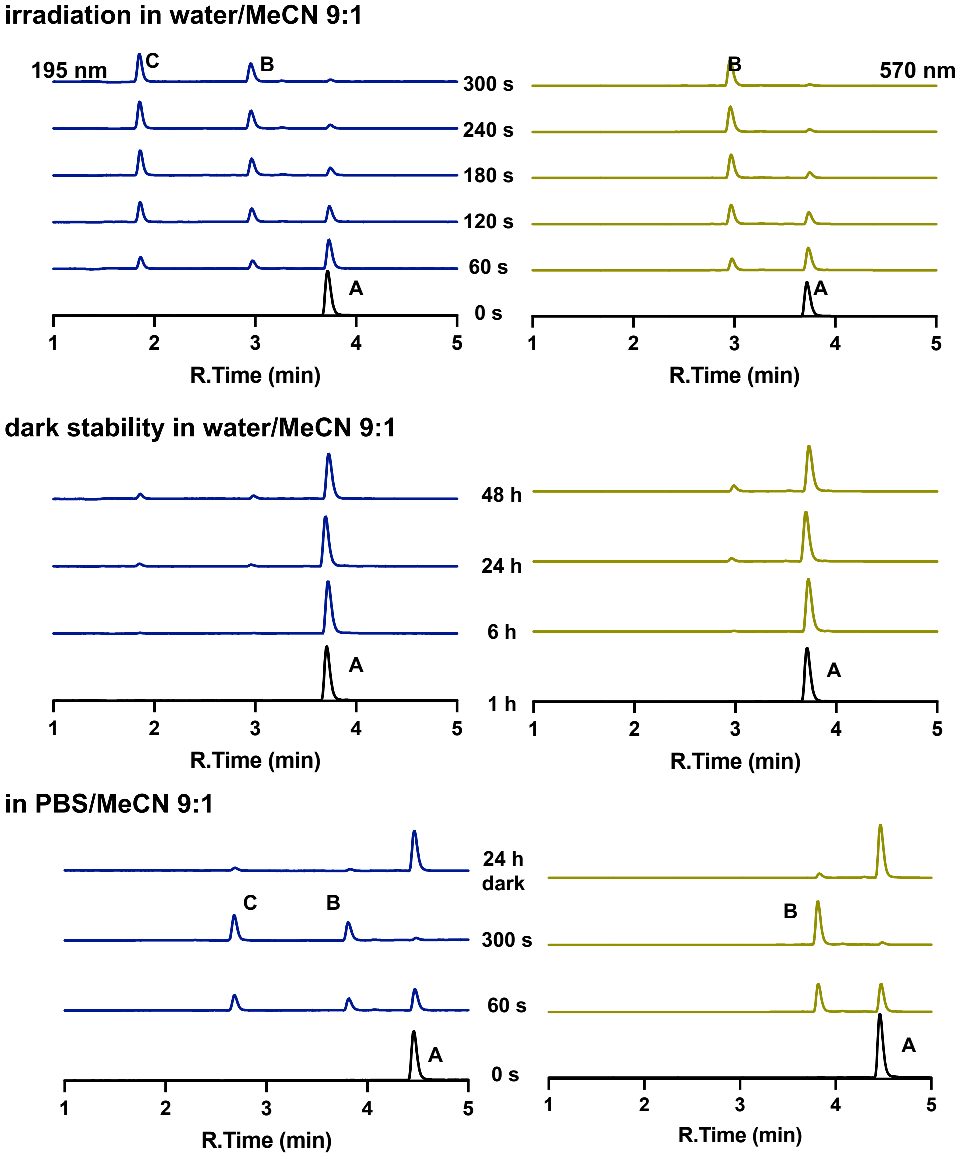

*Figure S7 Partial chromatograms of the orange light uncaging and stability experiments of **2-A** as followed by HPLC-UV-MS instrument. The release of phenylacetic acid (C) was monitored at 195 nm, the disappearance of the starting material (A) was monitored at 570 nm.*

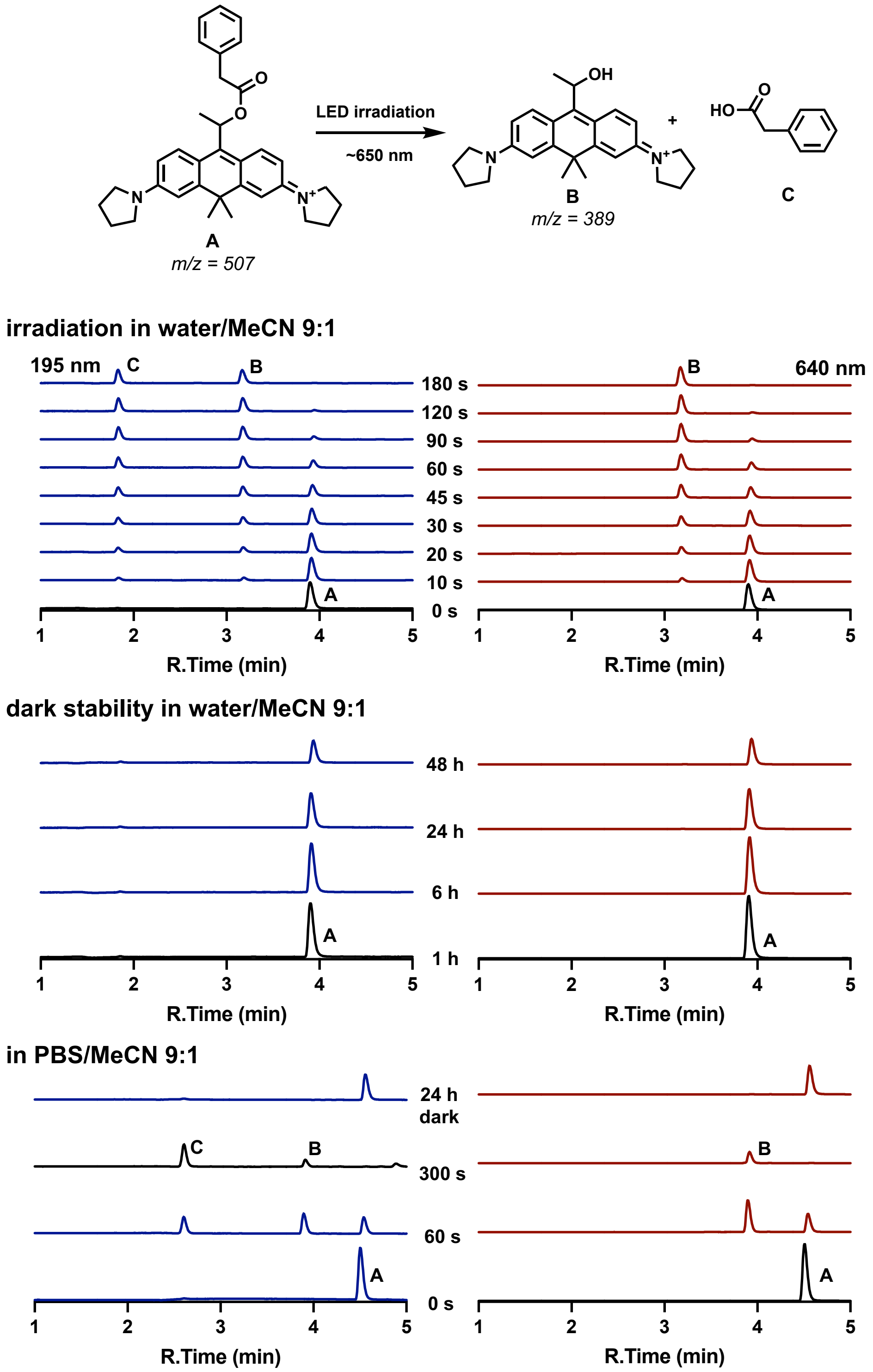

*Figure S8 Partial chromatograms of the red light uncaging and stability experiments of 3-**A** as followed by HPLC-UV-MS instrument. The release of phenylacetic acid (C) was monitored at 195 nm, the disappearance of the starting material (A) was monitored at 640 nm.*

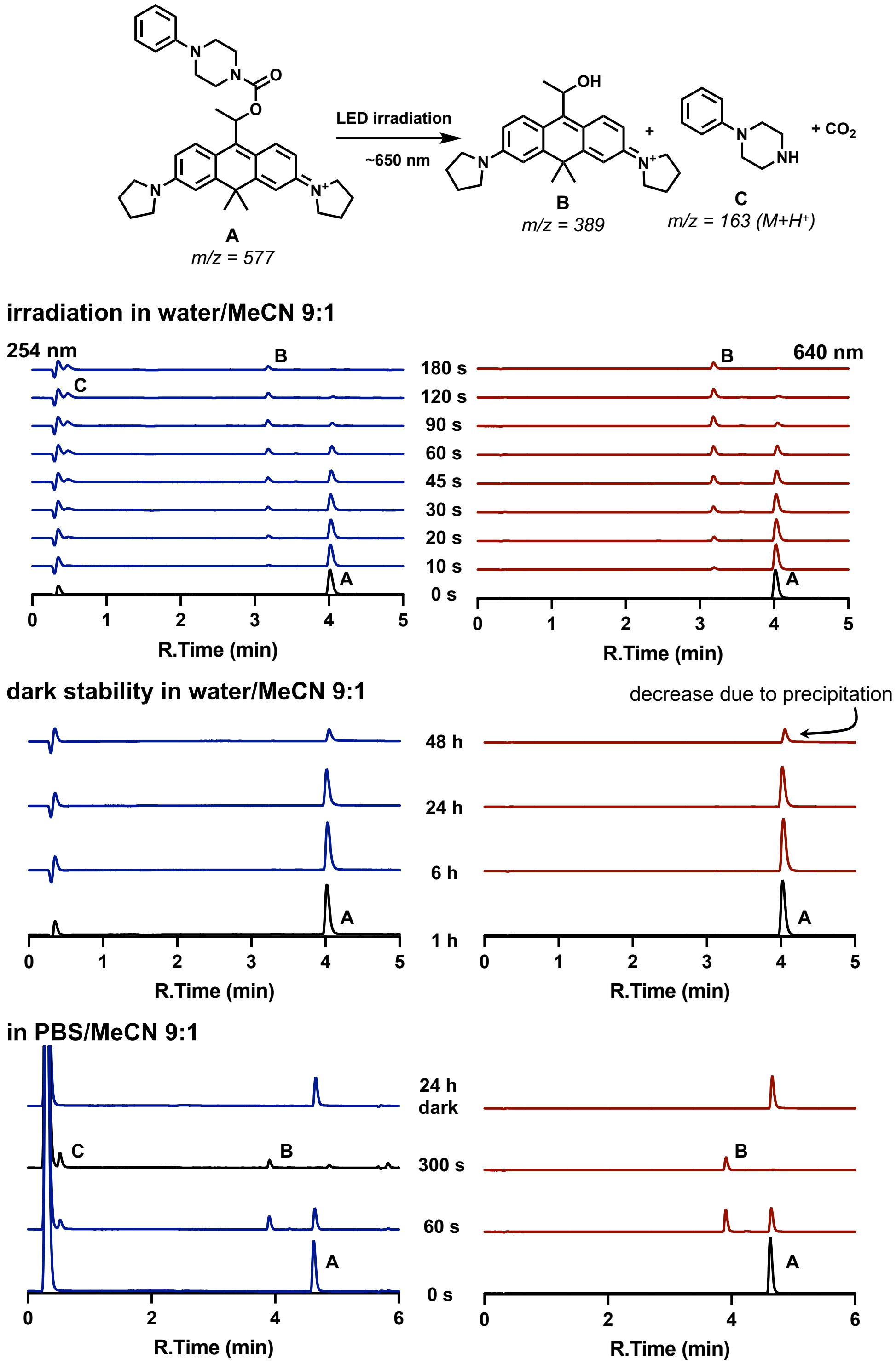

*Figure S9 Partial chromatograms of the uncaging and stability experiments of **3-B** as followed by HPLC-UV-MS instrument. The release of N-phenylpiperazine (C) was monitored at 254 nm, the disappearance of the starting material (A) was monitored at 640 nm. The decrease of **3-B** (peak A) is the result of precipitation due to high concentration (0.1 mM).*

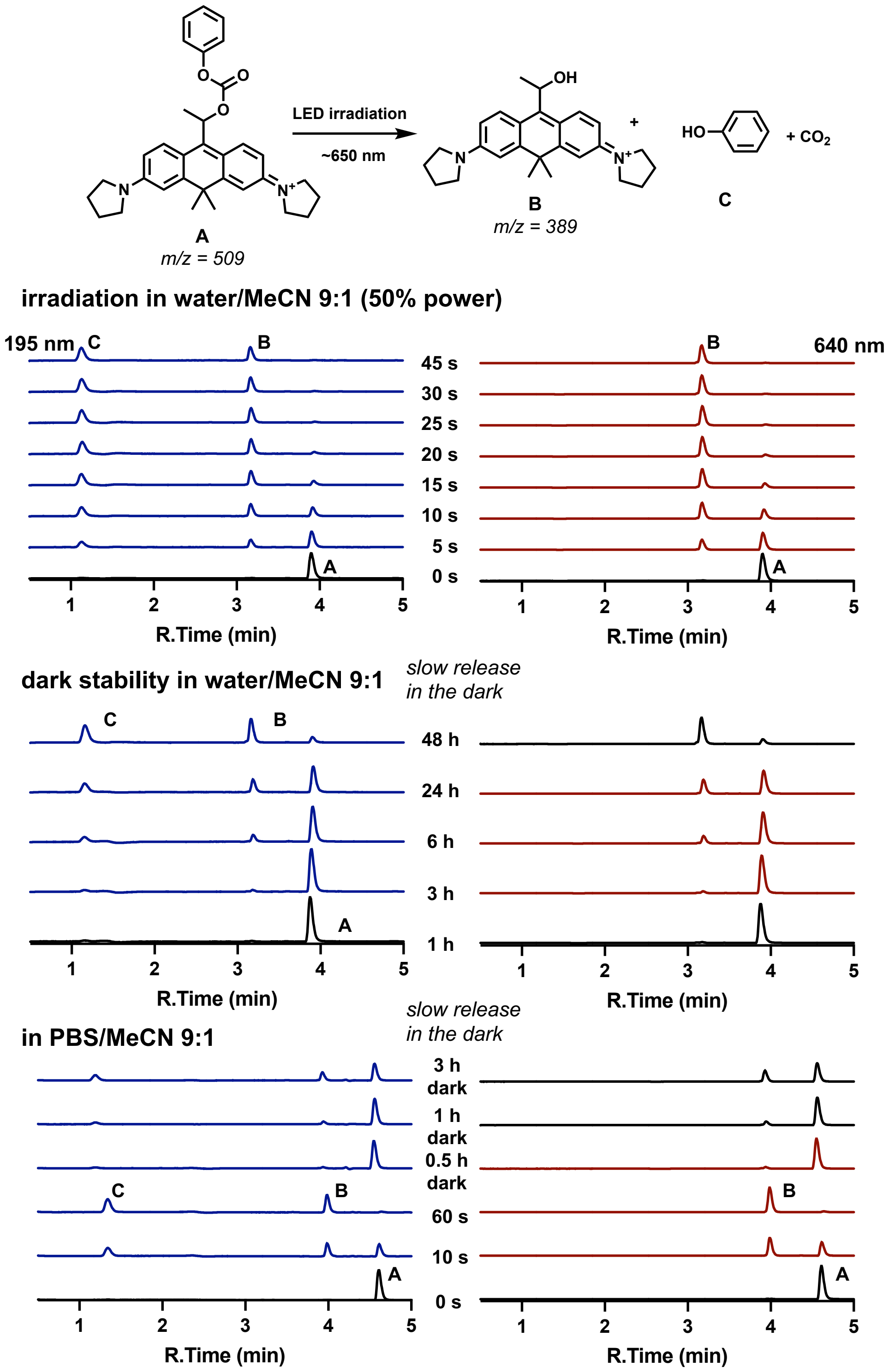

Figure S10 Partial chromatograms of the uncaging and stability experiments of ***3-C*** *as followed by HPLC-UV-MS instrument. The release of phenol (C) was monitored at 195 nm, the disappearance of the starting material (A) was monitored at 640 nm. The low dark aqueous stability of* ***3-C*** *is visible after 3 hours in the dark.*

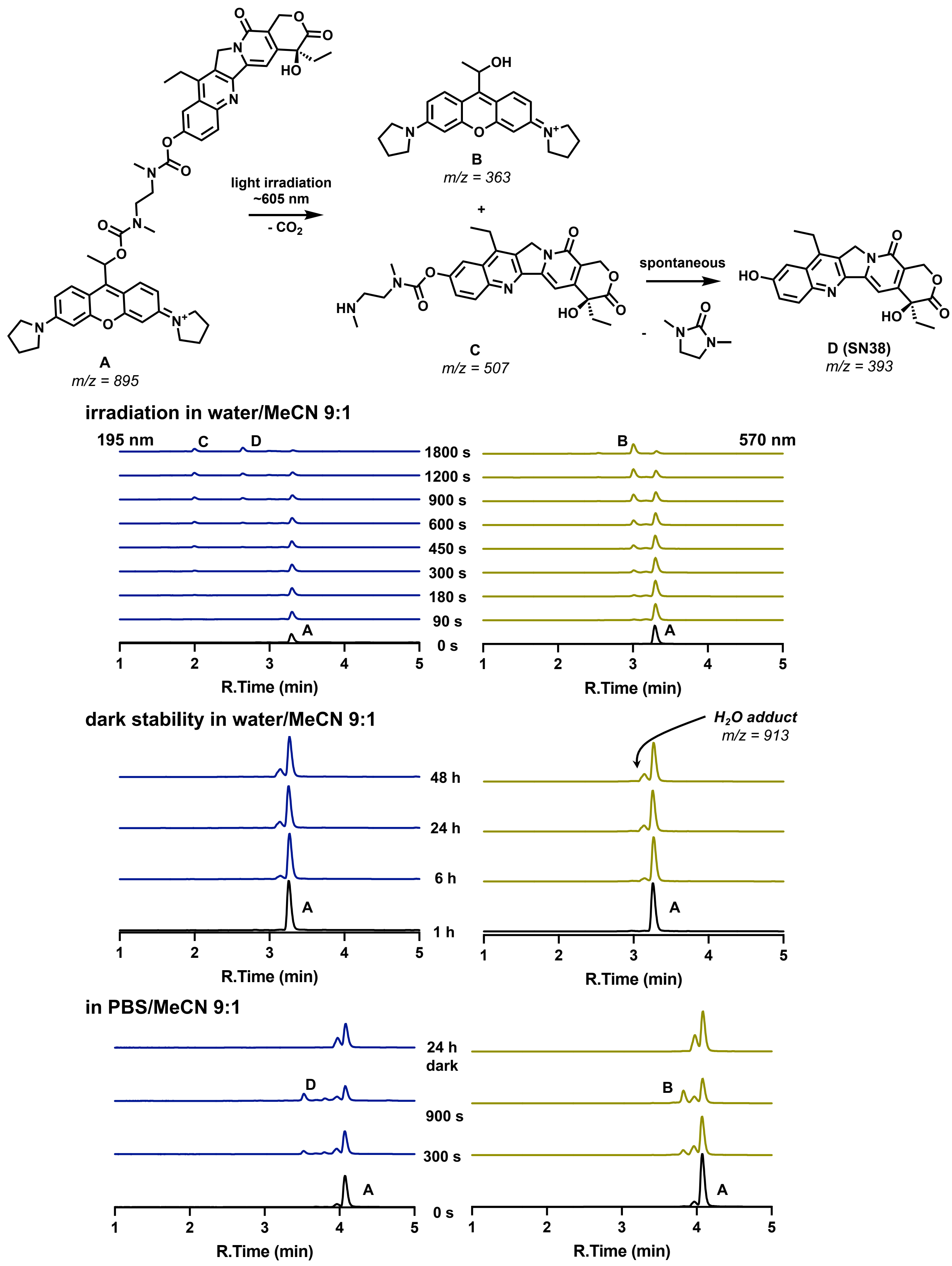

*Figure S11 Partial chromatograms of the uncaging and stability experiments of **2-SN38** as followed by HPLC-UV-MS instrument. The release of the SN38-adduct (C) and SN38 (D) was monitored at 369 nm, the disappearance of the starting material (A) was monitored at 570 nm.*

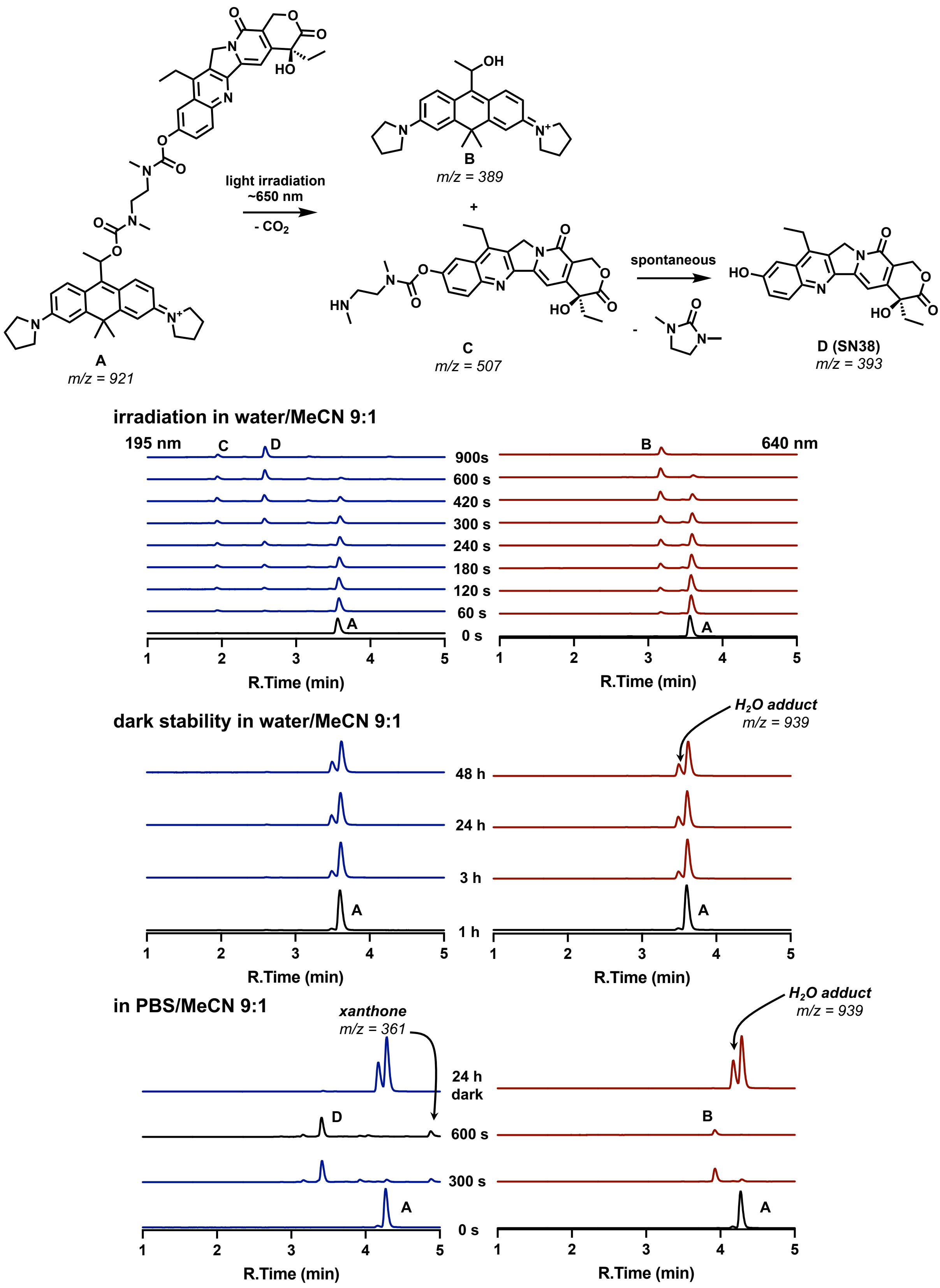

*Figure S12 Partial chromatograms of the uncaging and stability experiments of **3-SN38** as followed by HPLC-UV-MS instrument. The release of the SN38-adduct (C) and SN38 (D) was monitored at 369 nm, the disappearance of the starting material (A) was monitored at 640 nm.*

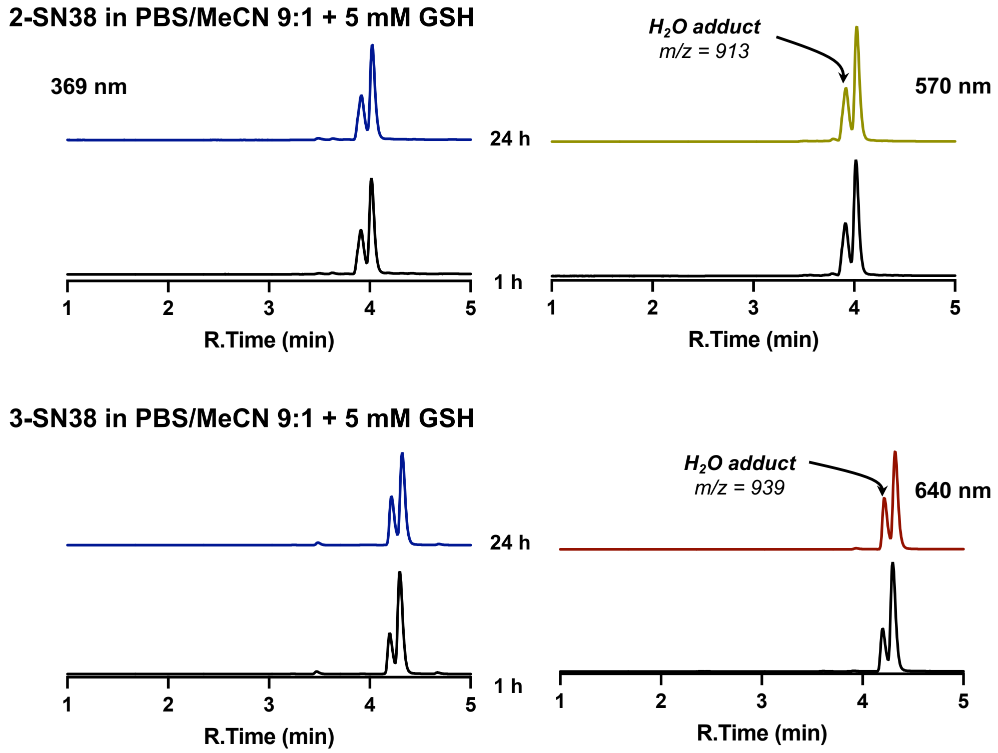

*Figure S13 Partial chromatograms demonstrating the stability of **2-SN38** and **3-SN38** in the presence of GSH (reduced form, 5 mM) in PBS (pH was adjusted to 7.4)*

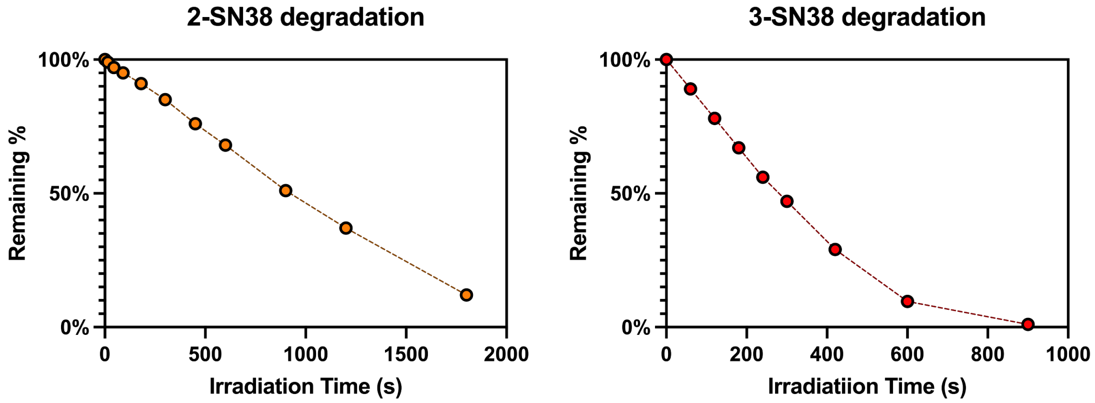

*Figure S14 Uncaging of the SN38 conjugates with orange (**2-SN38**) and red (**3-SN38**) light*

4.3. Photochemical Quantum Yield Determination

The relative photochemical quantum yields of the uncaging (payload/LG release) together with the degradation (decrease in SM concentration) quantum yields were determined from the results of the quantitative HPLC-UV experiments and shown in Table S1. In all cases, green light irradiation was used since all target conjugates absorbed in this range as well as the reference standard BODIPY from [6] (in methanol, as reported). The uncaging in case of the model conjugates were repeated 3 times in total. The photochemical quantum yields were determined using the following equation:

$$\Phi_u = \Phi_{u,ref} \cdot \frac{k \cdot c \cdot V \cdot a_{ref}}{k_{ref} \cdot c_{ref} \cdot V_{ref} \cdot a}$$

where

Φ_u	is the photochemical quantum yield of the sample
$\Phi_{u,ref}$	is the photochemical quantum yield of the reference
k	is the obtained reaction rate constant of the photoreaction of the sample
k_{ref}	is the obtained reaction rate constant of the photoreaction of the reference
c	is the concentration of the sample
c_{ref}	is the concentration of the reference
V	is the volume of the sample
V_{ref}	is the volume of the reference
a	is the absorption correction factor of the sample
a_{ref}	is the absorption correction factor of the reference

The absorption correction factors (a) were determined from the molar absorption coefficients and the green LED emission data obtained by a fluorimeter using the following equation:

$$a = \sum_{\lambda = \lambda_{min}}^{\lambda_{max}} \left[(1 - T(\lambda)) \frac{F(\lambda)}{\sum_{\lambda = \lambda_{min}}^{\lambda_{max}} [F(\lambda)]} \right]$$

where

$T(\lambda)$	is the transmittance at each wavelength (determined from the molar absorption coefficients)
$F(\lambda)$	is the LED emission at each wavelength
$\lambda_{min}, \lambda_{max}$	is the LED emission range

In brief, first we have calculated the transmittance at each wavelength (usual path length: 1.4 cm). Calculating 1-T at each wavelength results in the efficiency of photon absorption which was weighed at each wavelength with the emission of the LED with a normalized area under the curve of 1. In other words, the transmittance at each wavelength together with the emission data of the light source (normalized to the integrated emission) can be used to calculate the percentage of absorbed photons as can be seen in Fig S17.

The rate constants of the photoreactions were determined by considering the reactions monoexponential with an initial linear phase (up to 10-20% degradation). First, the concentration vs. HPLC-UV peak were determined for each leaving group and starting material. Then, in each case, a linear fitting of the time vs. released cargo percentages was performed that resulted in the rate constant k as determined from the fitted linear equation. The rate constants were determined for both the reference (with known Φ_u) and the sample. The determination of the degradation quantum yields was similar, in these cases the linear fitting was performed on the starting material percentages. For **3-C**, since the photon output of the device was reduced by 50%, the final quantum yields were multiplied by 2. For reference, a commonly known benchmark BODIPY derivative was used (in methanol, as reported) with a similar phenylacetic acid-derived cargo unit (Fig. S15, substrate 7 from [6]), Φ_u = 0.15%:

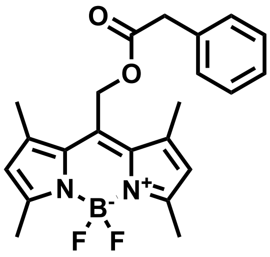

Figure S15 Structure of the reference BODIPY compound

Table S1 Uncaging and Degradation Quantum Yields

compound	uncaging quantum yield	degradation quantum yield
**1-A**	4.7% ±0.3%	6.2% ±0.7%
**2-A**	1.5% ±0.1%	1.9% ±0.2%
**3-A**	1.7% ±0.1%	2.2% ±0.3%
**3-B**	0.92% ±0.02%	1.7% ±0.4%
**3-C**	18% ±0.9%	25% ±2%
**2-SN38**	-	0.15% ±0.02%
**3-SN38**	-	0.18% ±0.03%

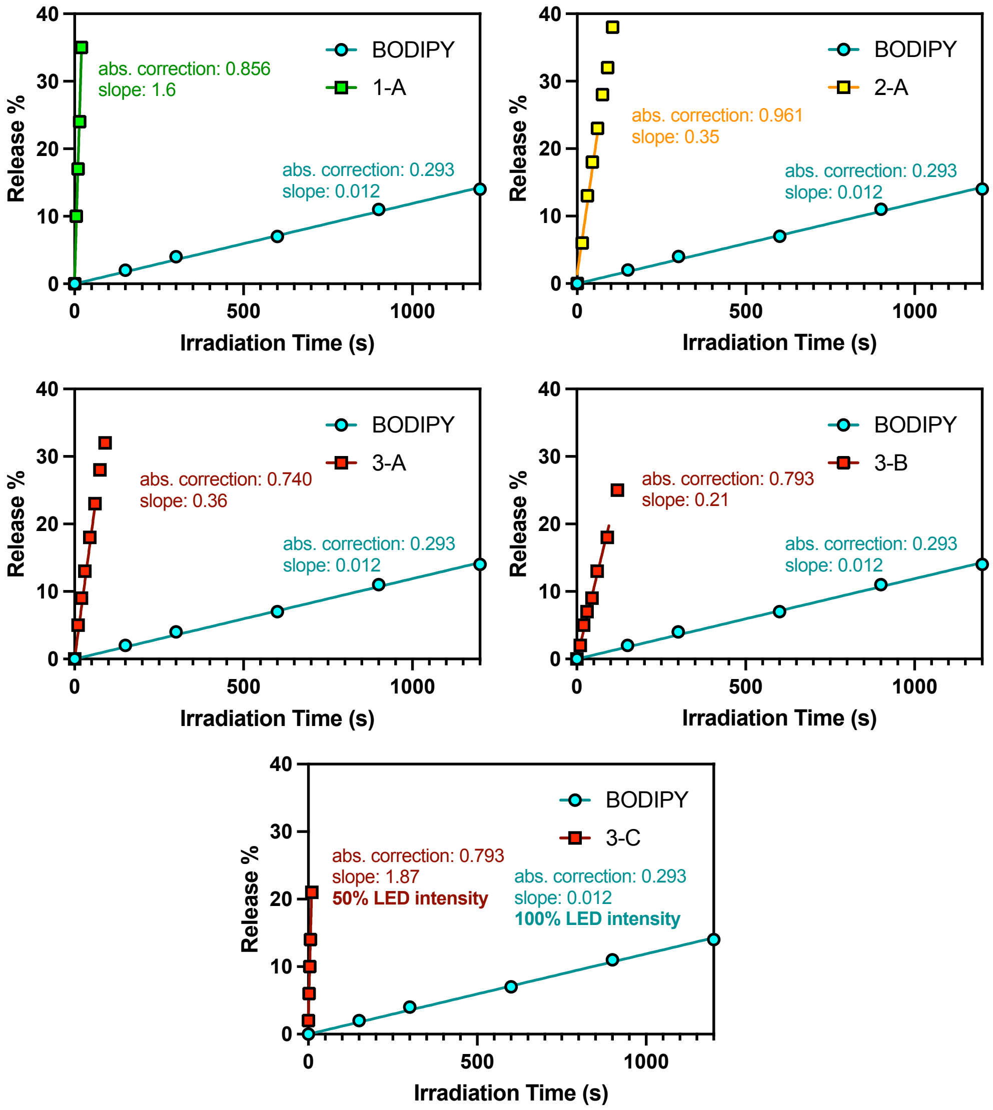

Figure S16 Comparison of photorelease with the BODIPY reference using green light irradiation (first trial shown). The regression for the initial linear phase was used for the quantum yield determination.

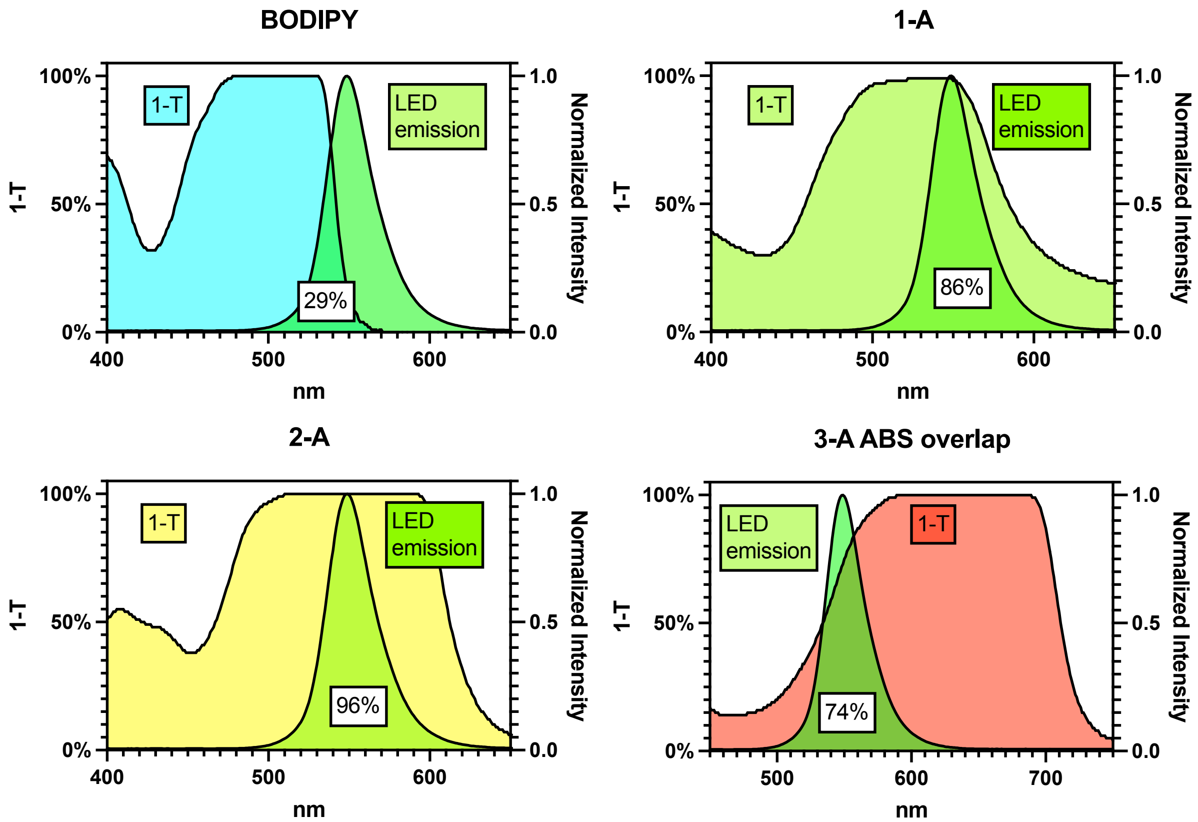

Figure S17 Examples for the calculation of absorption correction factors for the green LED light source (path length: 1.4 cm)

4.4. Chemical Yields of the Uncaging

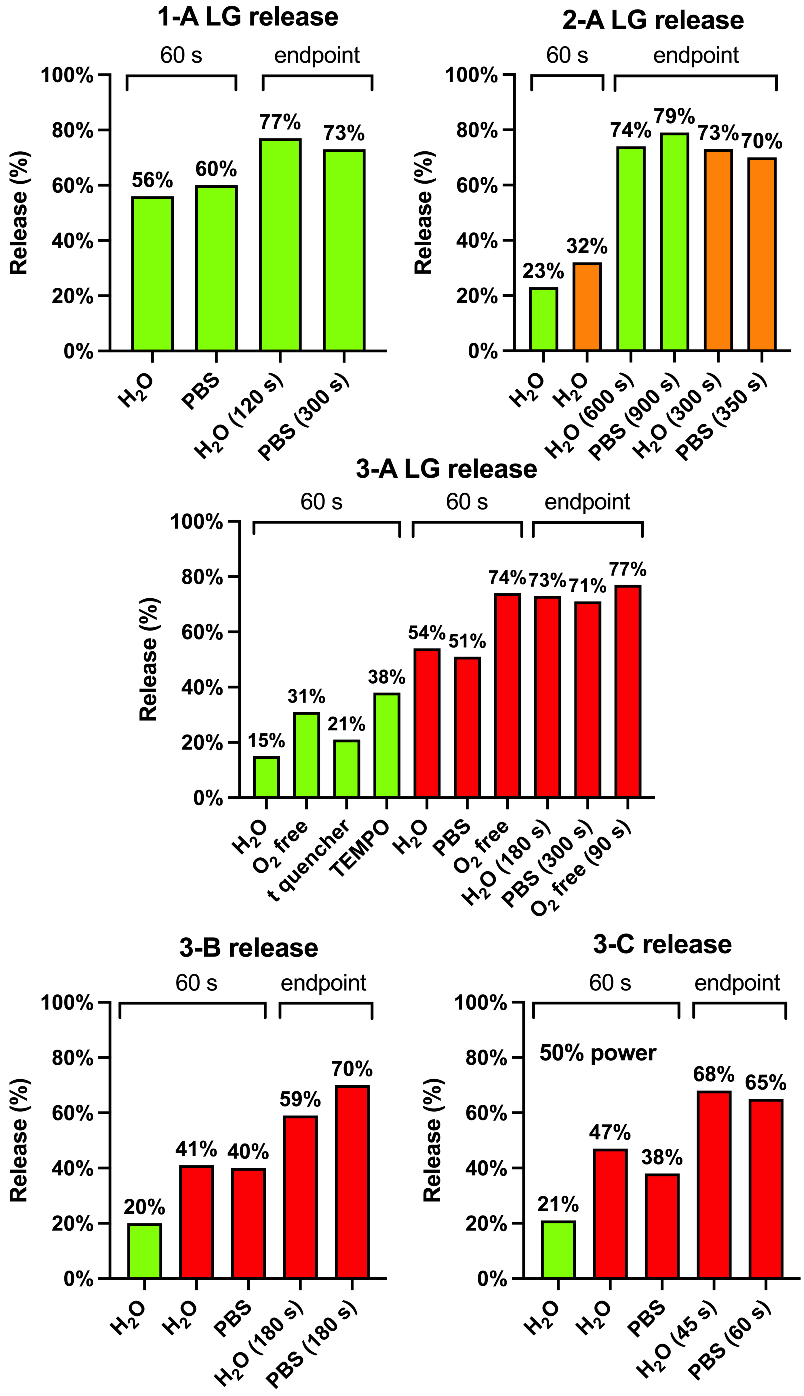

Figure S18 Chemical yields of uncaging of the model compounds. Endpoint refers to irradiation times in brackets where the remaining starting material concentration was below 5% of the original concentration.

4.5. Uncaging of **3-A** in Different Conditions

Water content dependence experiments were carried out in 0.1 mM solution in different water/MeCN compositions (irradiation with the green LED). The oxygen-dependency experiments were carried out in 0.1 mM solution of **3-A** in 90 % H_2O and 10 % CH_3CN (LC-MS grade solvents). The 1 mM stock solution of **3-A** was diluted to 10 mL under N_2 to receive the above-mentioned composition and nominal concentration. The solution was then deoxygenated with the freeze-pump-thaw technique (5 times) and the flask was finally

filled with N_2. The solution was distributed under N_2 to get eight LC-MS samples with the same concentration and volume. The samples were irradiated through different period of times with green light (10 seconds difference) and were measured. Another experiment was also conducted using red light irradiation and the results are shown in Fig S20.

The photochemical quantum yields of **3-A** are compiled in Table S2.

Table S2 Uncaging and Degradation Quantum Yields of 3-A under Different Conditions

conditions	uncaging quantum yield	degradation quantum yield
aerated	1.7% ±0.1%	2.2% ±0.3%
deoxygenated	2.3%	3.6%
singlet oxygen quencher	1.7%	3.3%
triplet quencher	1.8%	2.2%
TEMPO	3.5%	4.7%
water-MeCN 7:3	0.28%	0.40%
water-MeCN 1:1	0.072%	0.19%
water-MeCN 3:7	0.037%	0.087%
water-MeCN 1:9	0.018%	0.073%

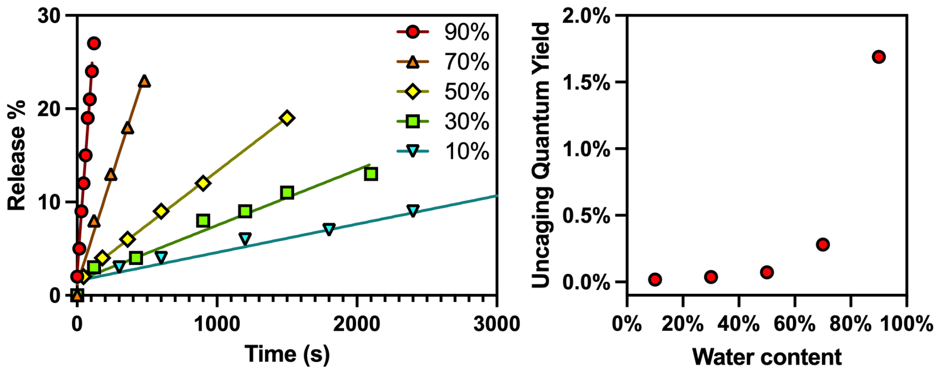

*Figure S19 Comparison of phenylacetic acid photorelease from **3-A** in water/MeCN mixtures (percentage refers to water content) using green light (left) and uncaging quantum yield dependence based on the water content of the mixtures*

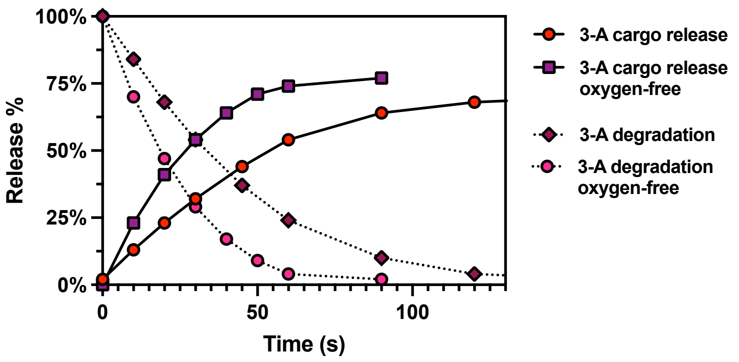

*Figure S20 Comparison of uncaging of **3-A** in the presence and absence of oxygen*

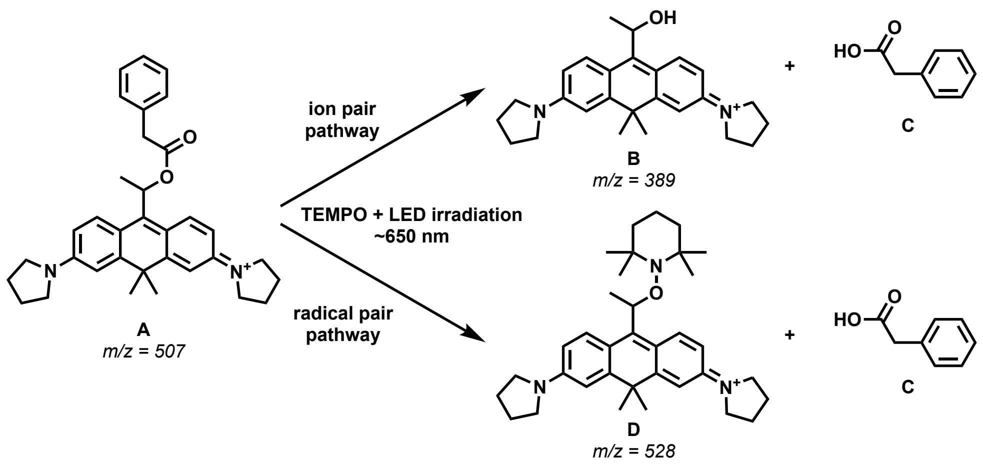

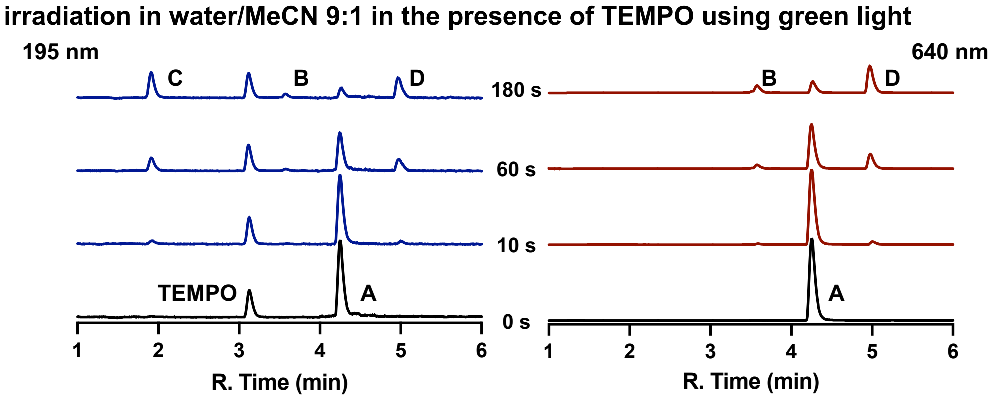

*Figure S21 Photolysis studies of **3-A** (0.1 mM) in the presence of TEMPO (1 mM) together with the assumed pathways and respective products*

4.6. Photolysis Studies of 1’-Br

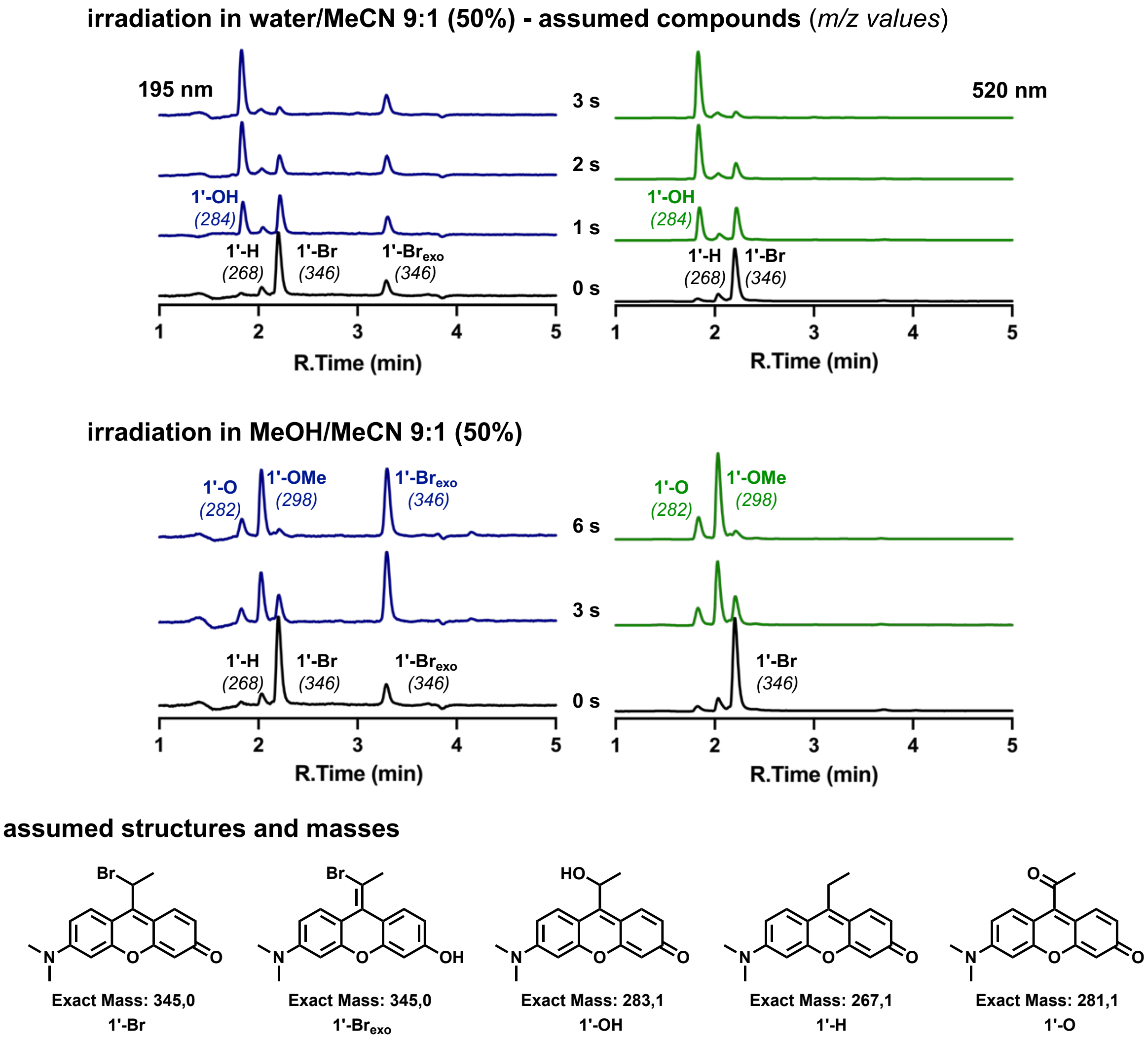

*Figure S22 Photolysis studies of **1’-Br** in various solvents. The photolysis were measured in 0.1 mM concentration, with 50% light intensity of the green LED (see Figure S5). At the zero points of the measurements impurities (1’-Br$_{exo}$ and 1’-H) can be seen as even the short-term stability of the **1’-Br** product is low. The assumed structures are also shown and annotated on the chromatograms. Note that the observed $[M+H]^+$ masses are shown on the chromatograms.*

4.7. Self-immolative Kinetics of the SN38-linker

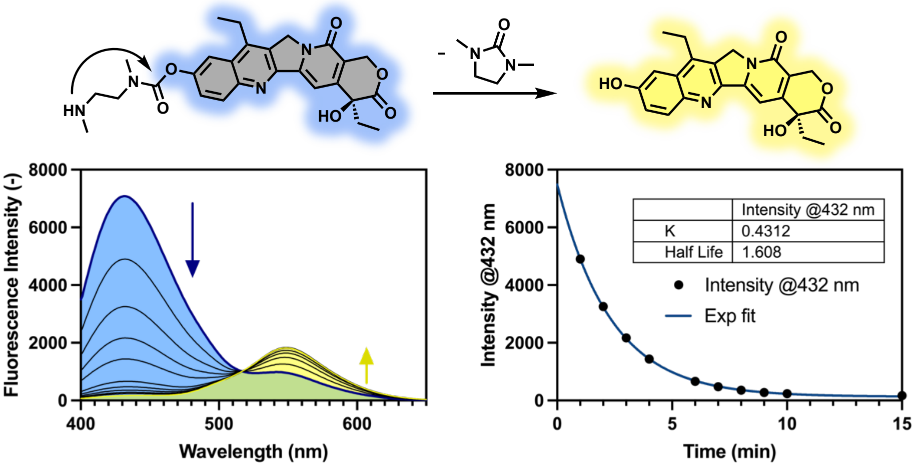

*Figure S23 Self-immolative kinetics of compound **8** (linker-appended SN38). The DMSO solutions of the TFA salt of **8** was directly added to PBS (pH 7.4, containing 10% DMSO) in a concentration of 2.5 μM. Excitation wavelength: 390 nm. The monoexponential fit provided the calculated half-life of the unstable conjugate.*

5. Computational Details

Density functional theory (DFT) calculations were carried out using the CAM-B3LYP functional [7] and the 6-311+G** basis set. For the excited-state computations, the time-dependent (TD) DFT [8] model was employed using the above functional and basis set. The solvent effects were taken into account by the polarized continuum model (PCM) [9] using water as solvent. All calculation were performed by the Gaussian quantum chemistry suite [10]. Molecular structures and orbitals were visualized using the IboView program [11].

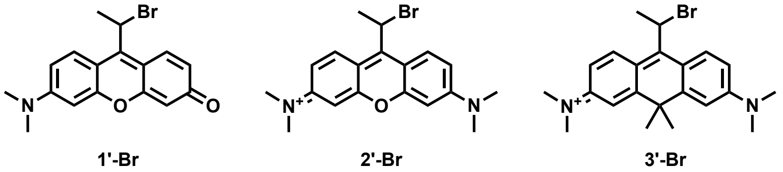

Figure S24 Structures of the model compounds investigated in silico

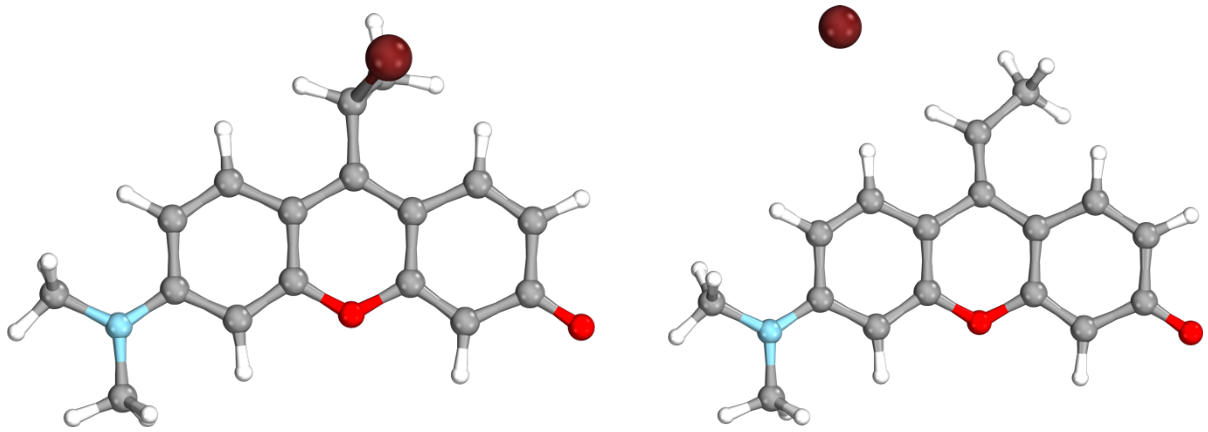

*Figure S25 Geometry of **1’-Br**: ground-state (left), ion-pair formed after the excitation to the S_1 state (right)*

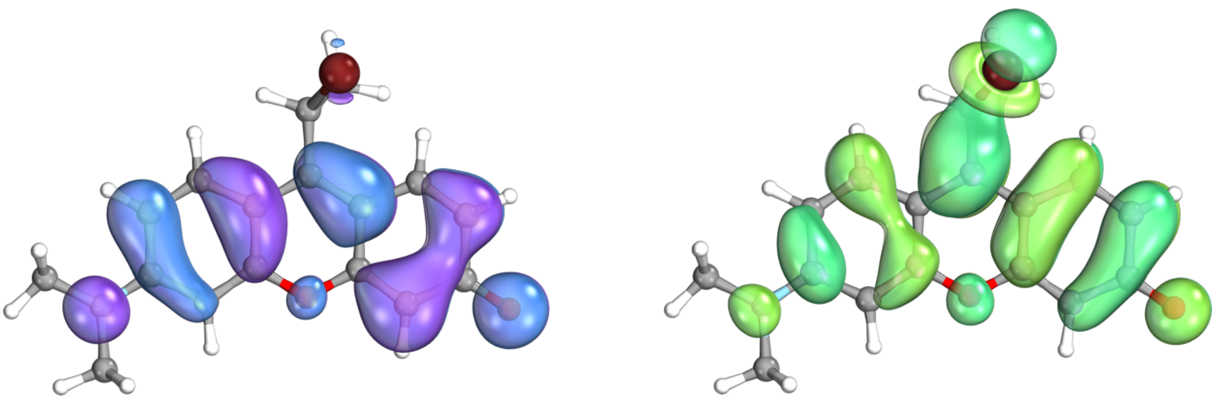

*Figure S26 HOMO (top) and LUMO (bottom) of **1’-Br***

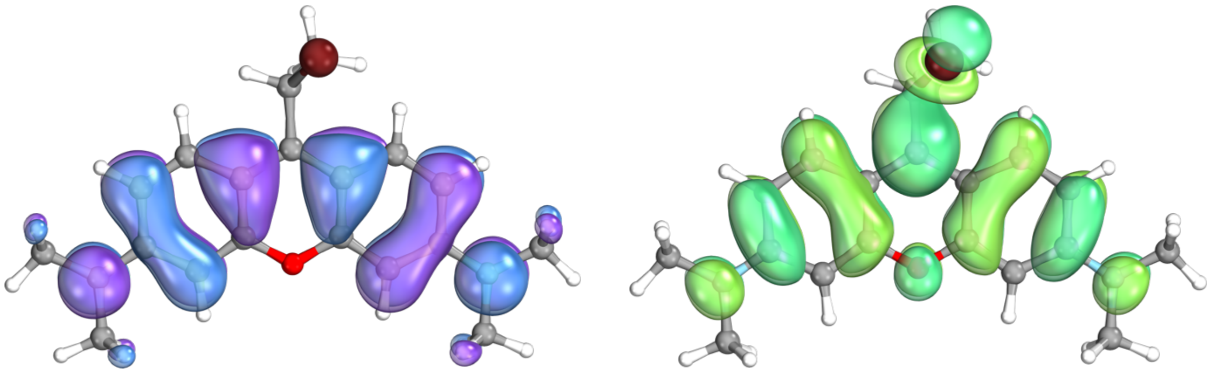

*Figure S27 HOMO (top) and LUMO (bottom) of **2’-Br***

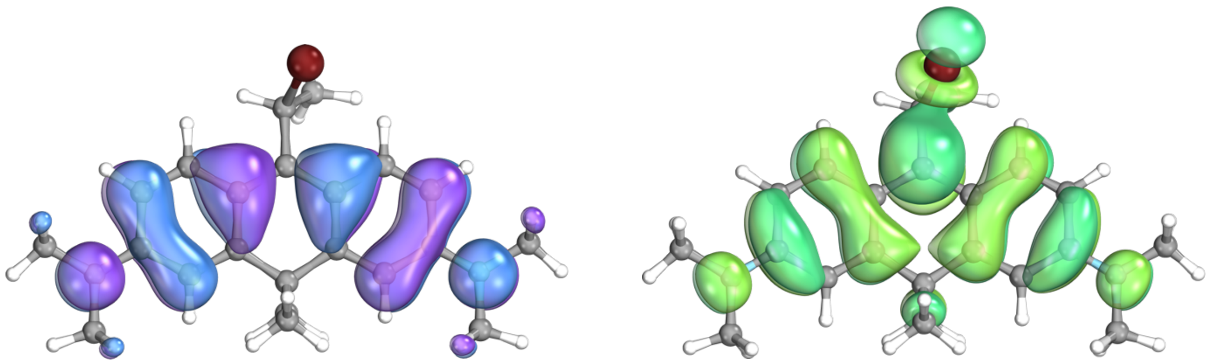

*Figure S28 HOMO (top) and LUMO (bottom) of **3’-Br***

Optimized geometries for the model compounds (Cartesian coordinates in Å)

1'-Br, S_0 state, total energy: -3436.65838 a.u.

C	0.011605	-0.014636	0.003631
C	0.008086	-0.007652	1.444972
C	1.310559	-0.002042	2.057425
C	2.473813	-0.035980	1.354825
C	2.468714	-0.065327	-0.079986
C	1.152442	-0.041534	-0.712013
O	1.419572	0.025763	3.408132
C	0.330360	0.075509	4.210775
C	-0.968970	0.065501	3.665460
C	-1.120790	0.006586	2.243700
C	0.593230	0.129882	5.562212
C	-0.464869	0.183837	6.484557
C	-1.789439	0.178292	5.959982
C	-2.018010	0.119760	4.615587
N	-0.239770	0.240982	7.816747
C	-1.350784	0.302813	8.755734
C	-2.520524	-0.097988	1.683805
C	-2.820080	-1.373153	0.912815
O	3.511931	-0.100672	-0.758855
C	1.122475	0.246546	8.326034
Br	-2.985175	1.521750	0.623743
H	1.127548	-0.038327	-1.795206
H	3.419993	-0.039696	1.880311
H	-0.929607	0.028856	-0.521654
H	1.626406	0.131217	5.873975
H	-2.636383	0.222158	6.627500
H	-3.049002	0.121995	4.293594
H	-3.236209	-0.020976	2.487770
H	-2.219930	-1.488070	0.014202
H	-3.874268	-1.400667	0.638938
H	-2.615634	-2.223765	1.568091
H	1.095416	0.294097	9.410776
H	1.659305	-0.661345	8.037152
H	1.681483	1.112221	7.959752
H	-0.953901	0.348412	9.765746
H	-1.964650	1.192175	8.591621

H	-1.988843	-0.581588	8.681088

2'-Br, S_0 state, total energy: -3495.81771 a.u.

C	0.008622	-0.028557	0.005676
C	0.006890	-0.024967	1.430614
C	1.290763	-0.023762	2.032472
C	2.467144	-0.055001	1.323406
C	2.438693	-0.079323	-0.082894
C	1.155032	-0.054180	-0.719216
O	1.418335	0.001222	3.375918
C	0.343463	0.048949	4.188747
C	-0.970297	0.041964	3.647533
C	-1.135855	-0.011555	2.251335
C	0.615317	0.095630	5.533410
C	-0.439983	0.145704	6.463539
C	-1.776837	0.143561	5.947287
C	-2.018745	0.091700	4.613703
N	-0.210972	0.195592	7.784844
C	-1.315875	0.252324	8.737243
C	-2.532921	-0.114787	1.687834
C	-2.824642	-1.392215	0.916425
N	3.567952	-0.116956	-0.809240
C	4.864513	-0.123999	-0.144364
C	1.155822	0.193245	8.291455
Br	-2.974183	1.504635	0.625446
C	3.522338	-0.135757	-2.267784
H	4.537757	-0.194389	-2.647267
H	1.088907	-0.047330	-1.796293
H	3.392004	-0.058277	1.878896
H	-0.926872	0.015505	-0.528415
H	1.649819	0.094084	5.839425
H	-2.615199	0.184758	6.625333
H	-3.051089	0.096020	4.298101
H	-3.252282	-0.037250	2.488333
H	-2.220572	-1.507555	0.020608
H	-3.877242	-1.418258	0.637127
H	-2.625082	-2.242585	1.573228
H	3.061416	0.771979	-2.663740
H	2.970918	-1.003444	-2.635376
H	1.130901	0.233256	9.375967

H	1.684234	-0.715185	7.992689
H	1.711949	1.060514	7.927593
H	-0.907397	0.301169	9.741843
H	-1.931882	1.139253	8.576188
H	-1.946420	-0.636573	8.667550
H	5.647539	-0.161403	-0.895432
H	5.005174	0.778943	0.454918
H	4.968806	-0.996819	0.504622

3'-Br, S_0 state, total energy: -3538.47952 a.u.

C	0.011361	-0.025306	-0.005223
C	0.000948	0.016137	1.417135
C	1.265895	0.012686	2.073263
C	2.418662	-0.093537	1.333635
C	2.417294	-0.164164	-0.080383
C	1.155781	-0.101468	-0.734084
C	1.380653	0.068615	3.588705
C	0.045413	0.304703	4.278622
C	-1.185727	0.297770	3.555536
C	-1.199571	0.072726	2.159499
C	0.038175	0.479853	5.639763
C	-1.150119	0.699297	6.379125
C	-2.369356	0.749169	5.649202
C	-2.372252	0.543520	4.306018
N	-1.127302	0.862934	7.711130
C	-2.363142	1.097958	8.450935
C	-2.537964	-0.192155	1.500636
C	-2.660335	-1.514915	0.759358
C	1.941454	-1.288920	4.080431
C	2.352083	1.208167	3.974475
N	3.559999	-0.274774	-0.778516
C	4.847819	-0.314275	-0.096019
C	0.132364	0.823729	8.445270
Br	-3.093428	1.348778	0.368445
C	3.535311	-0.347122	-2.235405
H	4.551356	-0.468536	-2.597915
H	1.094005	-0.106977	-1.811586
H	3.366202	-0.127696	1.845993
H	-0.916402	0.050328	-0.548216
H	0.973377	0.448520	6.174584

H	-3.302698	0.952273	6.151334
H	-3.329523	0.614202	3.812755
H	-3.314394	-0.186297	2.247531
H	-2.013303	-1.602176	-0.108216
H	-3.693524	-1.657489	0.444061
H	-2.404563	-2.318246	1.455139
H	3.122482	0.565374	-2.671940
H	2.946109	-1.200412	-2.577681
H	-0.071853	0.968905	9.501509
H	0.630783	-0.140366	8.322278
H	0.807749	1.616109	8.114490
H	-2.129850	1.174511	9.508386
H	-2.842021	2.028156	8.136707
H	-3.066605	0.274097	8.314629
H	5.636318	-0.405446	-0.836759
H	5.020003	0.599951	0.476760
H	4.911366	-1.170976	0.578915
H	3.333650	1.049487	3.531326
H	1.971917	2.171620	3.630852
H	2.486708	1.258409	5.053611
H	2.061351	-1.281758	5.163912
H	1.267728	-2.105655	3.814829
H	2.914099	-1.485320	3.629094

1’-Br, S_1 state, total energy: -3436.57532 a.u.

C	-0.000155	-0.138645	0.107337
C	0.051466	0.126635	1.497419
C	1.340831	0.229638	2.075245
C	2.493562	0.150507	1.345615
C	2.427023	-0.076405	-0.050666
C	1.127564	-0.234505	-0.643688
C	-1.127588	0.312761	2.330951
C	-0.899888	0.152818	3.726437
C	0.435015	0.262827	4.243408
O	1.486164	0.458742	3.403414
C	0.711830	0.229605	5.567305
C	-0.346653	0.009236	6.538211
C	-1.691481	-0.214177	5.998881
C	-1.935766	-0.141500	4.675801
O	-0.125038	-0.015206	7.748801

C	-2.335272	0.642084	1.738680
Br	-3.246638	-0.023312	-2.002086
N	3.532298	-0.157375	-0.794727
C	4.850721	-0.025970	-0.180059
C	3.460252	-0.396097	-2.236142
C	-3.606857	1.049625	2.381701
H	-2.475599	-0.451281	6.706689
H	1.726522	0.357849	5.920531
H	-2.929249	-0.345174	4.308954
H	3.433580	0.261031	1.863094
H	1.036922	-0.438476	-1.699299
H	-0.956567	-0.266490	-0.385340
H	-2.358608	0.657262	0.656063
H	-3.470538	1.527074	3.351109
H	-4.267251	0.184283	2.522866
H	-4.139994	1.733611	1.720356
H	5.609152	-0.115395	-0.950223
H	4.954476	0.947752	0.302006
H	5.009994	-0.811868	0.560688
H	4.464959	-0.367702	-2.643531
H	3.026571	-1.375216	-2.446211
H	2.866231	0.375670	-2.726413

2’-Br, S_1 state, total energy: -3495.72980 a.u.

C	0.014367	-0.108704	0.005770
C	0.013398	0.116574	1.410844
C	1.296354	0.218613	2.012339
C	2.464483	0.158405	1.306099
C	2.440378	-0.034807	-0.098887
C	1.163458	-0.177522	-0.723904
C	-1.157071	0.242439	2.217269
C	-0.962619	0.105444	3.624122
C	0.346836	0.217996	4.164833
O	1.420202	0.437650	3.353033
C	0.614625	0.151684	5.502737
C	-0.435879	-0.069612	6.430936
C	-1.751540	-0.259143	5.907026
C	-1.988803	-0.182410	4.567130
N	-0.196801	-0.118803	7.751922
C	-1.281129	-0.355059	8.698081

C	-2.471264	0.430356	1.588092
Br	-3.482704	-1.380574	1.480810
N	3.577905	-0.092776	-0.812881
C	4.873008	0.017669	-0.153716
C	3.542956	-0.293714	-2.256638
C	-3.410393	1.467222	2.174545
C	1.155765	0.049742	8.268457
H	-0.881155	-0.317770	9.706092
H	-2.572654	-0.480067	6.571824
H	1.639373	0.267401	5.821155
H	-2.989311	-0.372453	4.209875
H	3.391204	0.265308	1.848835
H	1.097055	-0.353099	-1.786729
H	-0.924937	-0.244988	-0.511547
H	-2.353167	0.633822	0.531679
H	-3.695902	1.273849	3.204465
H	-4.315107	1.531186	1.571323
H	-2.907443	2.437428	2.137998
H	-1.732989	-1.337198	8.539588
H	-2.054482	0.410032	8.603059
H	5.656795	-0.057748	-0.900492
H	4.970936	0.979060	0.356347
H	5.009885	-0.784910	0.575367
H	4.556812	-0.253619	-2.641268
H	3.114124	-1.267307	-2.507397
H	2.957261	0.487761	-2.745012
H	1.130363	-0.016902	9.351148
H	1.819248	-0.730481	7.887520
H	1.560212	1.025532	7.989667

3'-Br, S_1 state, total energy: -3538.40671 a.u.

C	0.001195	0.084877	0.071626
C	0.015637	0.340639	1.469910
C	1.283890	0.321405	2.138341
C	2.419273	0.010871	1.438453
C	2.395037	-0.272354	0.044587
C	1.135334	-0.209502	-0.622398
C	-1.188200	0.539174	2.214650
C	-1.139582	0.156350	3.591505
C	0.113915	0.143023	4.289670

C	1.321860	0.761386	3.596789
C	0.178552	-0.343614	5.567898
C	-0.970762	-0.826596	6.253415
C	-2.217274	-0.793027	5.560615
C	-2.284568	-0.323454	4.284411
N	-0.888896	-1.293085	7.510933
C	-2.083354	-1.737031	8.221077
C	1.172649	2.305031	3.641810
C	2.640728	0.404254	4.283698
C	-2.413352	0.976818	1.527960
Br	-3.581598	-0.653855	0.960736
N	3.520375	-0.575158	-0.625920
C	4.797920	-0.674040	0.068585
C	3.491866	-0.805780	-2.065714
C	-3.310438	1.968926	2.241435
C	0.394228	-1.361812	8.199197
H	-1.806476	-2.019175	9.231845
H	-3.114640	-1.163848	6.032691
H	1.122600	-0.342190	6.086084
H	-3.233295	-0.370995	3.771099
H	3.368956	0.007666	1.946685
H	1.066104	-0.417753	-1.679455
H	-0.939687	0.074718	-0.461209
H	-2.169281	1.364667	0.546342
H	-3.701100	1.603813	3.187525
H	-4.148512	2.242881	1.601890
H	-2.725851	2.871009	2.441880
H	-2.533657	-2.603580	7.730430
H	-2.824548	-0.937043	8.276845
H	5.558235	-0.995881	-0.636050
H	5.097080	0.292237	0.483894
H	4.746163	-1.404948	0.878388
H	4.505664	-0.973758	-2.414694
H	2.890917	-1.684714	-2.312306
H	3.084935	0.059792	-2.592843
H	0.248344	-1.822725	9.170838
H	1.107985	-1.965162	7.634214
H	0.815166	-0.363876	8.348134
H	2.645592	0.767747	5.311124
H	2.824510	-0.671578	4.293000

H	3.475235	0.895988	3.784197
H	2.013489	2.772004	3.125450
H	0.249651	2.638332	3.167656
H	1.170135	2.644842	4.679211

1’-Br, T_1 state, total energy: -3436.643972 a.u.

C	0.027638	-0.039038	-0.012016
C	0.034418	0.131851	1.395295
C	1.309893	0.195191	2.008580
C	2.480968	0.145059	1.297745
C	2.452920	-0.018596	-0.104167
C	1.172044	-0.126741	-0.737197
C	-1.147164	0.306512	2.193871
C	-0.954387	0.128458	3.617342
C	0.352494	0.202187	4.153269
O	1.437063	0.382954	3.344085
C	0.598768	0.102142	5.489694
C	-0.481584	-0.163877	6.410944
C	-1.802532	-0.366255	5.834470
C	-2.010992	-0.216667	4.504325
O	-0.283541	-0.252545	7.633756
C	-2.292230	0.771962	1.638315
Br	-3.536184	-1.933270	1.141206
N	3.582360	-0.084576	-0.821821
C	4.881073	0.004341	-0.163905
C	3.541138	-0.260046	-2.271250
C	-3.474946	1.417796	2.251705
H	-2.604328	-0.652130	6.503059
H	1.606815	0.184436	5.873785
H	-2.984269	-0.427923	4.086123
H	3.408168	0.230267	1.842306
H	1.106042	-0.290361	-1.801375
H	-0.917488	-0.150840	-0.523222
H	-2.306763	0.816917	0.561404
H	-3.624774	1.241816	3.309873
H	-4.373434	1.129633	1.709797
H	-3.356771	2.500514	2.112839
H	5.661934	-0.063442	-0.914601
H	4.988191	0.955956	0.360832
H	5.014412	-0.811855	0.549484

H	4.557982	-0.274035	-2.650171
H	3.061779	-1.203348	-2.539638
H	3.007280	0.561772	-2.751566

2’-Br, T_1 state, total energy: -3495.76113 a.u.

C	0.010759	-0.103463	0.018498
C	0.008168	0.173051	1.410429
C	1.297129	0.298152	2.011753
C	2.459455	0.202210	1.318352
C	2.436380	-0.040089	-0.085573
C	1.163367	-0.197387	-0.706114
C	-1.160001	0.324495	2.216348
C	-0.966125	0.163938	3.619259
C	0.347655	0.298279	4.164946
O	1.401875	0.595609	3.344272
C	0.621049	0.201166	5.490489
C	-0.425993	-0.069098	6.418265
C	-1.734775	-0.274472	5.893179
C	-1.979360	-0.172407	4.554582
N	-0.182128	-0.143205	7.736730
C	-1.260979	-0.410044	8.686355
C	-2.470155	0.480414	1.583914
Br	-3.466698	-1.365773	1.407416
N	3.575053	-0.125090	-0.793787
C	4.874328	-0.016893	-0.134264
C	3.546880	-0.344197	-2.238729
C	-3.457560	1.465514	2.176639
C	1.171089	0.037997	8.256858
H	-0.858435	-0.369973	9.692887
H	-2.548432	-0.531858	6.554350
H	1.641352	0.343398	5.812708
H	-2.975982	-0.376941	4.195335
H	3.386331	0.333732	1.855708
H	1.098758	-0.411204	-1.762208
H	-0.928239	-0.256684	-0.493974
H	-2.355430	0.700964	0.530091
H	-3.744309	1.243755	3.200674
H	-4.360019	1.507245	1.568343
H	-2.990314	2.454290	2.162989

H	-1.686645	-1.400803	8.519802
H	-2.047194	0.339848	8.595984
H	5.656626	-0.161937	-0.871682
H	4.996410	0.969664	0.316070
H	4.977607	-0.780467	0.637625
H	4.559633	-0.280153	-2.621998
H	3.145790	-1.332121	-2.472055
H	2.941545	0.416822	-2.730881
H	1.152465	-0.081536	9.334786
H	1.848242	-0.705979	7.835193
H	1.542477	1.036738	8.021953

3'-Br, T_1 state, total energy: -3538.461337 a.u.

C	-0.000041	0.189978	0.090035
C	0.049192	0.448015	1.481517
C	1.307759	0.397689	2.154492
C	2.424314	0.035496	1.460676
C	2.372736	-0.264936	0.063477
C	1.112998	-0.158300	-0.606220
C	-1.164789	0.686916	2.249296
C	-1.111023	0.237694	3.617885
C	0.137105	0.211024	4.308950
C	1.353660	0.826157	3.618732
C	0.197789	-0.295331	5.577024
C	-0.953860	-0.818759	6.233243
C	-2.186278	-0.821646	5.519546
C	-2.248827	-0.307642	4.260728
N	-0.871509	-1.307711	7.479429
C	-2.056630	-1.824442	8.158910
C	1.228209	2.370415	3.672897
C	2.671909	0.447393	4.290379
C	-2.230370	1.317264	1.652527
Br	-3.608632	-0.935255	0.395287
N	3.471839	-0.635052	-0.593674
C	4.750983	-0.792774	0.102431
C	3.438666	-0.911170	-2.032918
C	-3.399159	1.965555	2.295074
C	0.406983	-1.352630	8.182228
H	-1.787236	-2.102505	9.172429
H	-3.073410	-1.254549	5.955547

H	1.137773	-0.296473	6.102900
H	-3.176689	-0.382332	3.712648
H	3.374609	-0.014172	1.966814
H	1.030055	-0.384900	-1.657810
H	-0.954989	0.204152	-0.416878
H	-2.100589	1.592014	0.615286
H	-3.692302	1.547494	3.251921
H	-4.252780	1.971334	1.620600
H	-3.119883	3.013488	2.468542
H	-2.445668	-2.706877	7.646874
H	-2.837952	-1.064982	8.205450
H	5.494014	-1.140890	-0.605905
H	5.077415	0.162168	0.515441
H	4.657127	-1.525279	0.903817
H	4.446855	-1.120739	-2.370652
H	2.809567	-1.778244	-2.237020
H	3.058480	-0.047222	-2.576478
H	0.255305	-1.798631	9.159477
H	1.129986	-1.957394	7.632448
H	0.811263	-0.348045	8.319157
H	2.693358	0.818656	5.315018
H	2.837549	-0.631590	4.307052
H	3.509194	0.920925	3.776889
H	2.077617	2.827806	3.162431
H	0.312741	2.730632	3.204971
H	1.233718	2.700127	4.713216

Results of the constrained potential energy surface scans (increments with respect to the equilibrium Br-C bond lengths in Å and total energies in atomic units)

2’-Br, S_1 state

0.0 -3495.72980
0.2 -3495.72814
0.4 -3495.72667
0.6 -3495.72588
0.8 -3495.72581
1.0 -3495.72502
1.2 -3495.72417
1.4 -3495.72347
1.6 -3495.72421

1.8 -3495.72432

3’-Br, S_1 state

0.0 -3538.40671

0.2 -3538.40512

0.4 -3538.40354

0.6 -3538.40264

0.8 -3538.40207

1.0 -3538.40174

1.2 -3538.40159

6. Fluorescence Microscopy

6.1. Cell sample preparation for fluorescence imaging experiments

SK-OV-3 (ATCC HTB-77) cells were maintained in McCoy's 5A (Modified) Medium, HEPES (Gibco 22330021) media supplemented with 10% FBS (Gibco 10500-064), 1% penicillin-streptomycin (Gibco 15140-122). The cells were cultured at 37°C in a 5% CO_2 atmosphere and passaged - using trypsin (Gibco 25300-054) - every 3–4 days up to 20 passages.

6.2. Live cell fluorescence imaging

SK-OV-3 (4,5000 cell/well) cells were transferred into μ-Slide 8 well plates (Ibidi 80827) and were incubated for 40 h at 37°C in a 5% CO_2 atmosphere. After 30 min treatment with compounds **2-SN38** and **3-SN38** in the concentration of 1 μM and in some cases in combination with 1 nM MitoTracker Deep Red FM (Invitrogen M22426) or 1 nM LysoTracker Deep red (Invitrogen L12492) or 50 nM MitoTracker Red FM (Invitrogen M22425) or 50 nM LysoTracker red DND-99 (Invitrogen L7528) samples were subjected to microscopy analyses.

Confocal images were acquired on a Leica TCS SP8 STED 3X microscope using 552 nm and 638 nm lasers for excitation. The images were taken using a Leica HC PL APO 100x/1.40 oil immersion objective along with Leica PMT and HyD detectors. All images were taken using dual detection (channel 1: PMT detector, channel 2: HyD detector). The channels were selected for minimal bleed-through between the green/yellow and red channels. In case of **2-SN38**, MitoTracker Deep Red and LysoTracker Deep Red were used. The following wavelengths were used: green/yellow channel (for **2-SN38**): 565-624 nm using 552 nm excitation; red channel (for the trackers): 700-800 nm using 638 nm excitation. For **3-SN38**, MitoTracker Red and LysoTracker Red were used with the following wavelengths: green/yellow channel (for the emission of the MitoTracker Red and LysoTracker Red) 564-600 nm using 552 nm excitation; red channel (for **3-SN38**) 715-800 nm using 638 nm excitation. The images were processed using ImageJ software and the pixel values were plotted on each channel. The brightness on the displayed images is enhanced for better visibility. Pearson’s r values (R_p) were calculated using the JACoP plugin in ImageJ

(Bolte S, Cordelieres FP. A guided tour into subcellular colocalization analysis in light microscopy. J Microsc. 2006;224:213-32.)

6.3. Colocalization Images

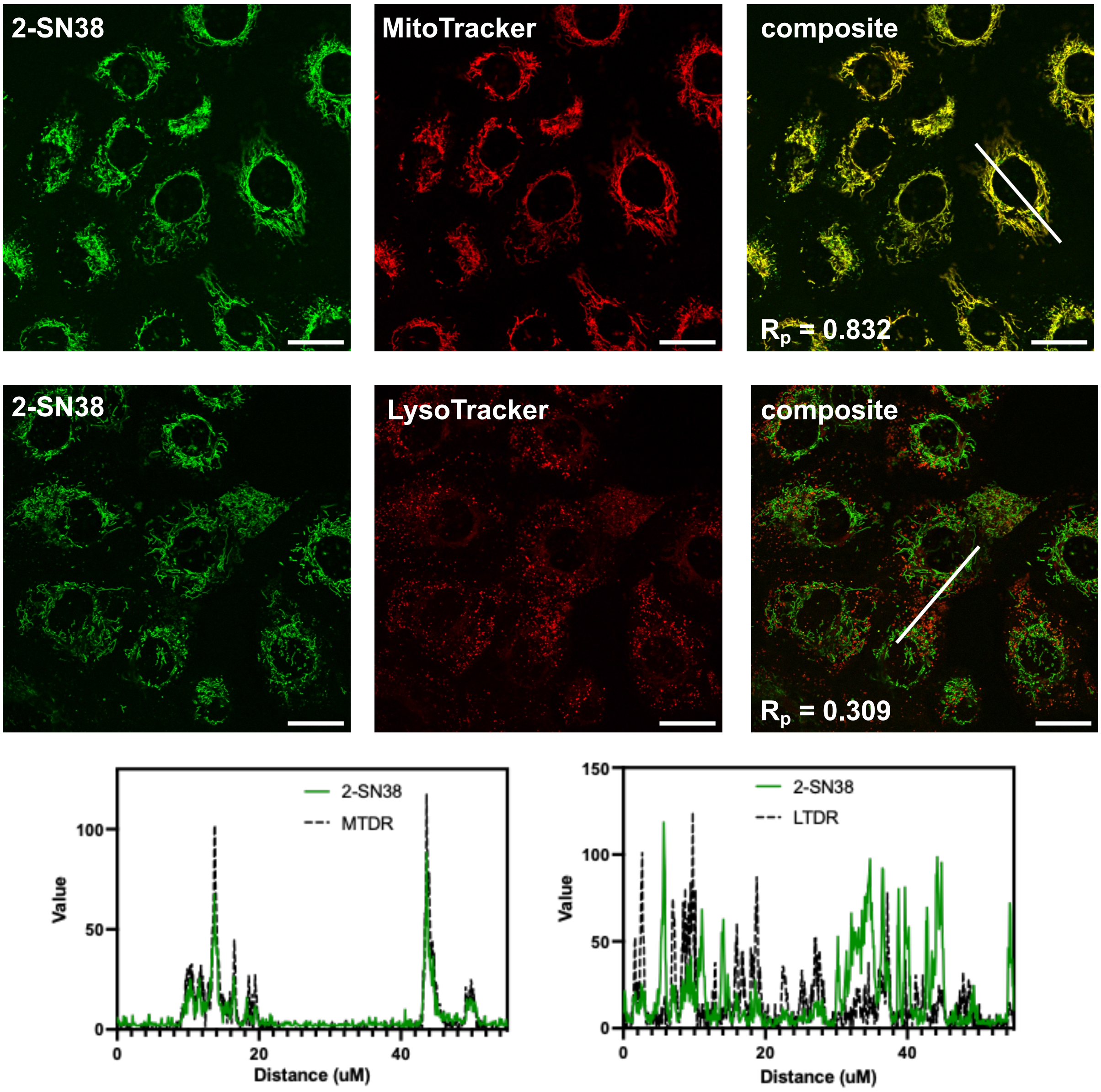

*Figure S29 Confocal images of SK-OV-3 cells treated with 1 μM **2-SN38** and 1 nM MitoTracker Deep Red or LysoTracker Deep Red. The white lines in the composite are plotted on the graphs to show the colocalization of **2-SN38** with the MitoTracker. Scale bar: 25 μm*

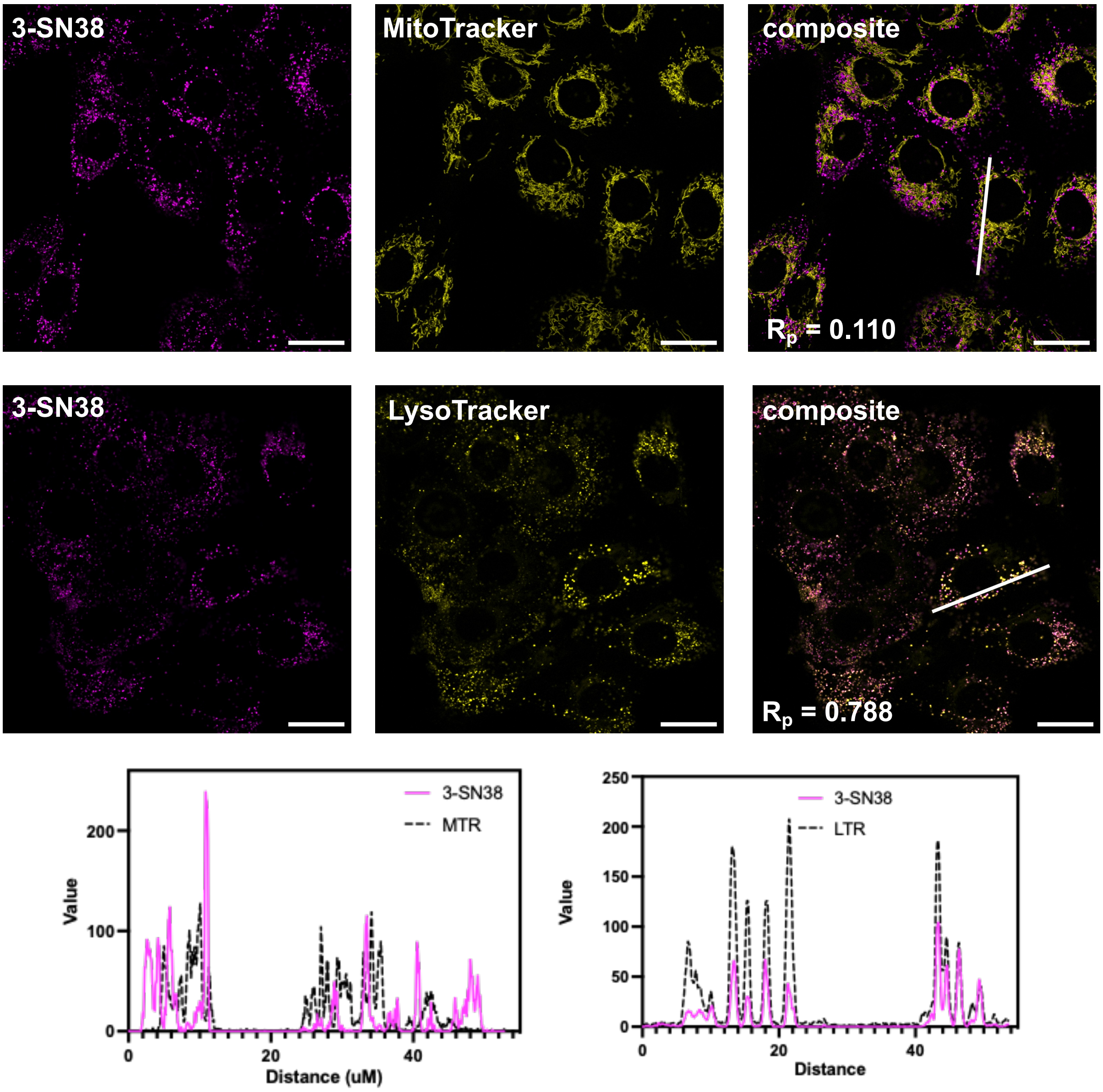

*Figure S30 Confocal images of SK-OV-3 cells treated with 1 μM **3-SN38** and 50 nM MitoTracker Red or LysoTracker Red. The white lines in the composite are plotted on the graphs to show the colocalization of **3-SN38** with the LysoTracker. Scale bar: 25 μm*

7. Cytotoxicity Determination

7.1. Experimental Details

A viability test was carried out to assess the toxicity of SN38 as well as the photocaged SN38 conjugates and compound **11** as the negative control after light irradiation and in the dark on SK-OV-3 cells. Cells were transferred into a 48-well plate (Thermo Fisher Scientific, 130187) (4,500 cells/well) and incubated for 20 – 24 h at 37 °C in a 5% CO_2 atmosphere in McCoy's 5A (Modified) Medium, HEPES (Gibco 22330021) supplemented with 10% FBS (Gibco 10500-064), 1% penicillin-streptomycin (Gibco 15140-122). Cells were treated with compounds in the concentration range of 10^{-11}-10^{-4} M for 90 min followed by light irradiation (using the setup presented at section 4.1) or kept in the dark at 37 °C in 5% CO_2 atmosphere. After treatment and irradiation, cells were kept at 37 °C in 5% CO_2 atmosphere for 72 hours. After the incubation period, supernatants were replaced with 0.5 mg/ml MTT (3-(4,5-dimethylthiazol-2-yl)-2,5-diphenyltetrazolium bromide; Sigma M5655-1G) solution (in complete DMEM: Dulbecco's modified Eagle's medium (DMEM, Gibco 41965-039), supplemented with 10% FBS (Gibco 10500-064), 1% penicillin-streptomycin (Gibco 15140-122), 1% Glutamax (Gibco 35050-061) and 1% sodium pyruvate (Life Technologies, Gibco 11360-070)) and incubated for 120 min at 37 °C in the dark. The insoluble formazan crystals were dissolved in 250 µl DMSO. Absorbance was detected at 540 nm using a Biotek Synergy 2 Cytation 3 imaging plate reader with Gen5 software version 3.08 (Biotek Winooski, VT, USA). Viability was expressed as ratio (n = 3) of the readings of untreated control cells.

7.2. Irradiation Time-Dependent Viabilities

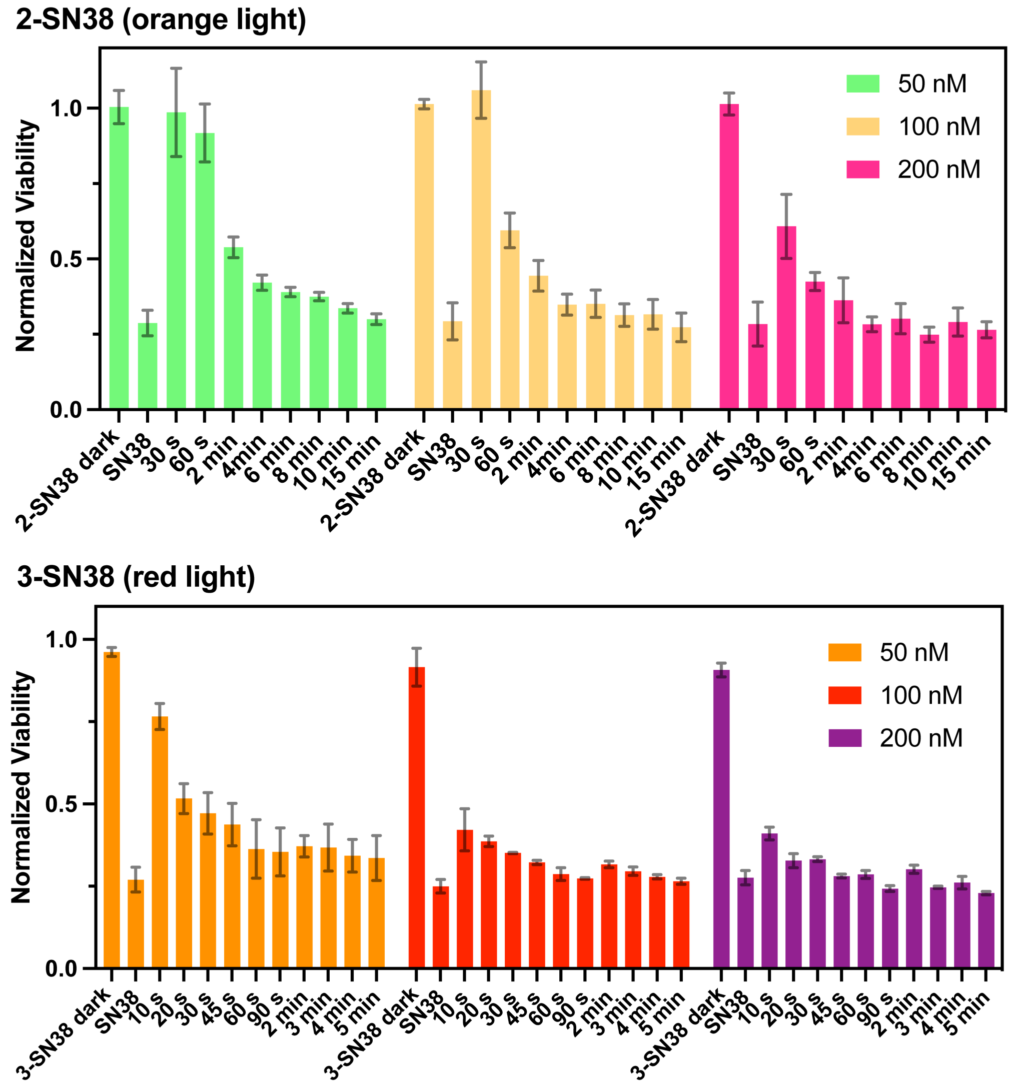

*Figure S31 Irradiation time-dependent viabilities for **2-SN38** and **3-SN38** on SK-OV-3 cells.*

*Table S3 Significance of the irradiation time-dependent normalized viabilitiy values of **2-SN38** compared to control cells, **2-SN38** in the dark and SN38*

significance level
not significant
p<0.05
p<0.01
p<0.001
p<0.0001

Significance against control cells (no treatment)

		Irradiation time								
	dark	0.5 min	1 min	2 min	4 min	6 min	8 min	10 min	15 min	SN38
50 nM	0.5094	0.6838	0.7232	0.0028	0.0023	0.0035	0.0035	0.0028	0.0022	0.0006
100 nM	0.0785	0.2419	0.0025	0.0008	0.001	0.0005	0.0007	0.0004	0.0003	0.0004
200 nM	0.3764	0.015	0.0007	0.0006	0.0006	0.0001	0.0005	0.0001	0.0005	0.0003

Significance against cells treated with **2-SN38** in the dark

	Irradiation time								
	0.5 min	1 min	2 min	4 min	6 min	8 min	10 min	15 min	SN38
50 nM	0.3555	0.0721	0.0004	0.0005	0.0011	0.0012	0.0009	0.0007	<0.0001
100 nM	0.5641	0.0026	0.0009	<0.0001	0.0004	0.0001	0.0005	0.0004	0.0014
200 nM	0.0092	<0.0001	0.0007	<0.0001	<0.0001	<0.0001	<0.0001	<0.0001	0.0007

Significance against cells treated with SN38

		Irradiation time							
	dark	0.5 min	1 min	2 min	4 min	6 min	8 min	10 min	15 min
50 nM	<0.0001	0.013	0.004	0.0068	0.1704	0.5245	0.8967	0.2915	0.085
100 nM	0.0014	0.0009	0.0109	0.2183	0.5316	0.5979	0.2161	0.2552	0.0861
200 nM	0.0007	0.0377	0.3063	0.9385	0.1754	0.2771	0.0938	0.212	0.1227

*Table S4 Significance of the irradiation time-dependent normalized viabilitiy values of **3-SN38** compared to control cells, **3-SN38** in the dark and SN38*

Significance against control cells (no treatment)

		Irradiation time										
	dark	10 s	20 s	30 s	45 s	60 s	90 s	2 min	3 min	4 min	5 min	SN38
50 nM	0.3559	0.0286	0.0054	0.0063	0.0051	0.0022	0.004	0.0036	0.0033	0.0036	0.0033	0.0005
100 nM	0.3736	0.0029	0.0022	0.0037	0.0034	0.0025	0.0027	0.0028	0.0033	0.0017	0.0029	0.0003
200 nM	0.2572	0.0005	0.0007	0.0004	0.0007	0.0003	0.001	0.0003	0.0004	0.0002	0.0003	0.0003

Significance against cells treated with **3-SN38** in the dark

	Irradiation time										
	10 s	20 s	30 s	45 s	60 s	90 s	2 min	3 min	4 min	5 min	SN38
50 nM	0.0517	0.0013	0.003	0.0021	0.0003	0.0018	0.0011	0.0009	0.0014	0.0011	<0.0001
100 nM	<0.0001	<0.0001	0.0004	0.0005	0.0001	0.0002	0.0001	0.0006	<0.0001	0.0004	0.0002
200 nM	0.0003	<0.0001	<0.0001	<0.0001	<0.0001	<0.0001	<0.0001	<0.0001	0.0002	<0.0001	0.0008

Significance against cells treated with SN38

		Irradiation time									
	dark	10 s	20 s	30 s	60 s	90 s	1.5 min	2 min	3 min	4 min	5 min
50 nM	<0.0001	0.0012	0.0038	0.0146	0.0198	0.0708	0.1153	0.0642	0.0683	0.1641	0.2087
100 nM	0.0002	0.0213	0.0404	0.1129	0.2475	0.8007	0.2972	0.873	0.5978	0.9877	0.6603
200 nM	0.0008	0.0909	0.5657	0.5255	0.6933	0.7868	0.2799	0.9691	0.3155	0.4866	0.2089

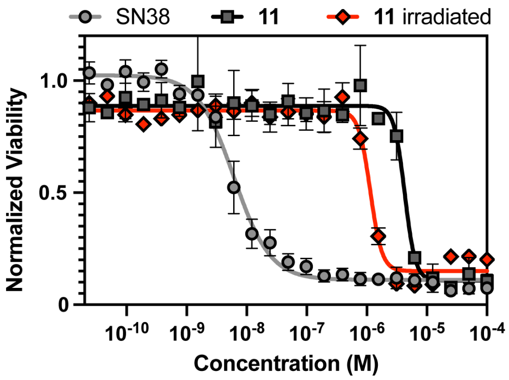

*Figure S32 Concentration-dependent effects of **11** (photocage **3**-linker without SN38) on normalized viabilities together with their EC50 curves. Irradiation time: 60 s red light*

8. NMR Spectra

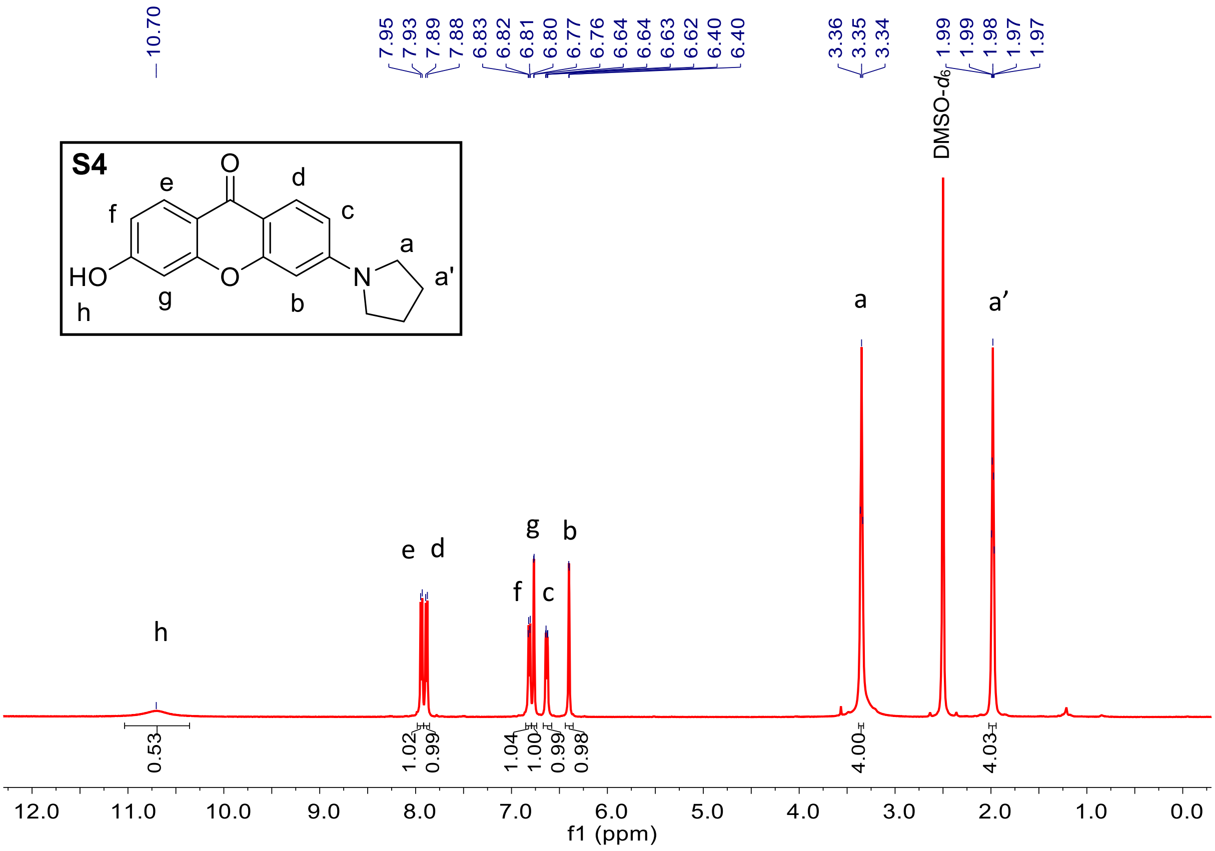

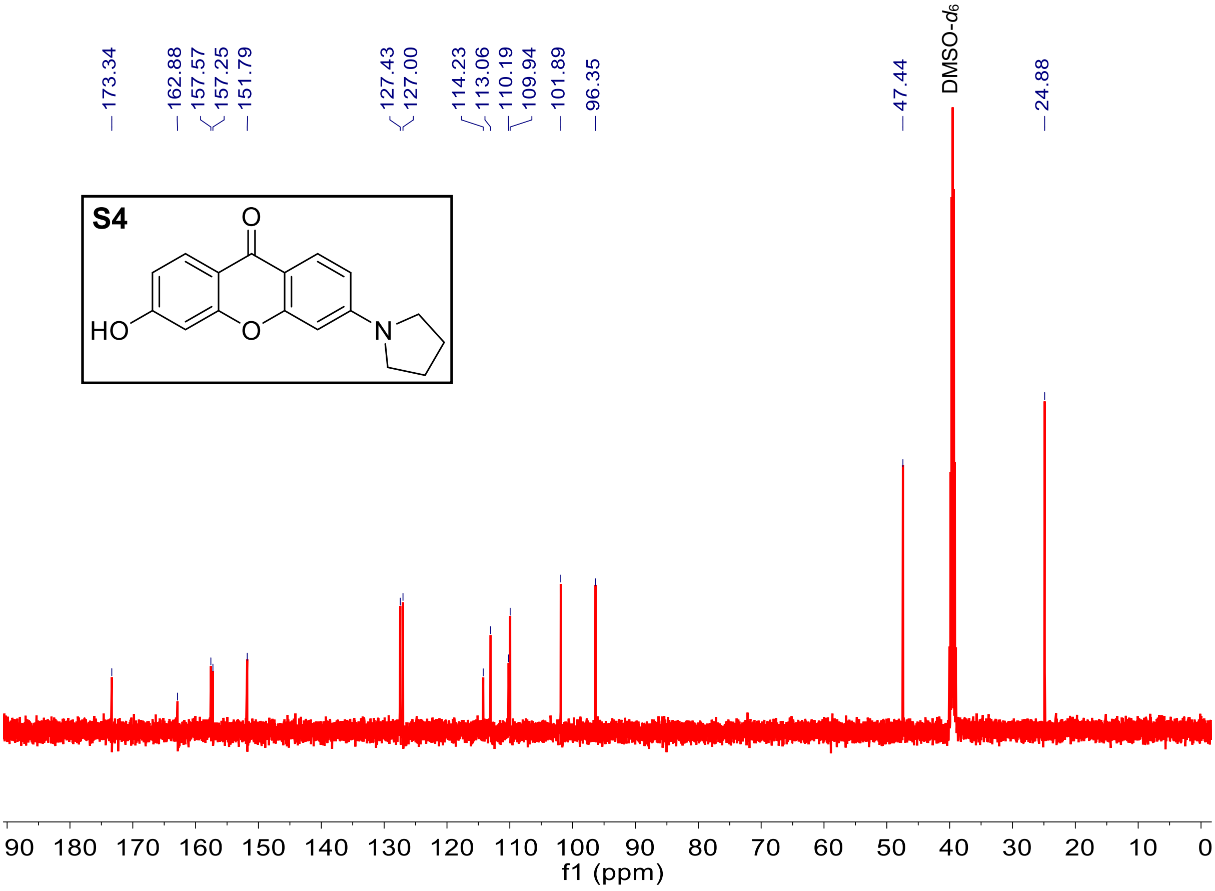

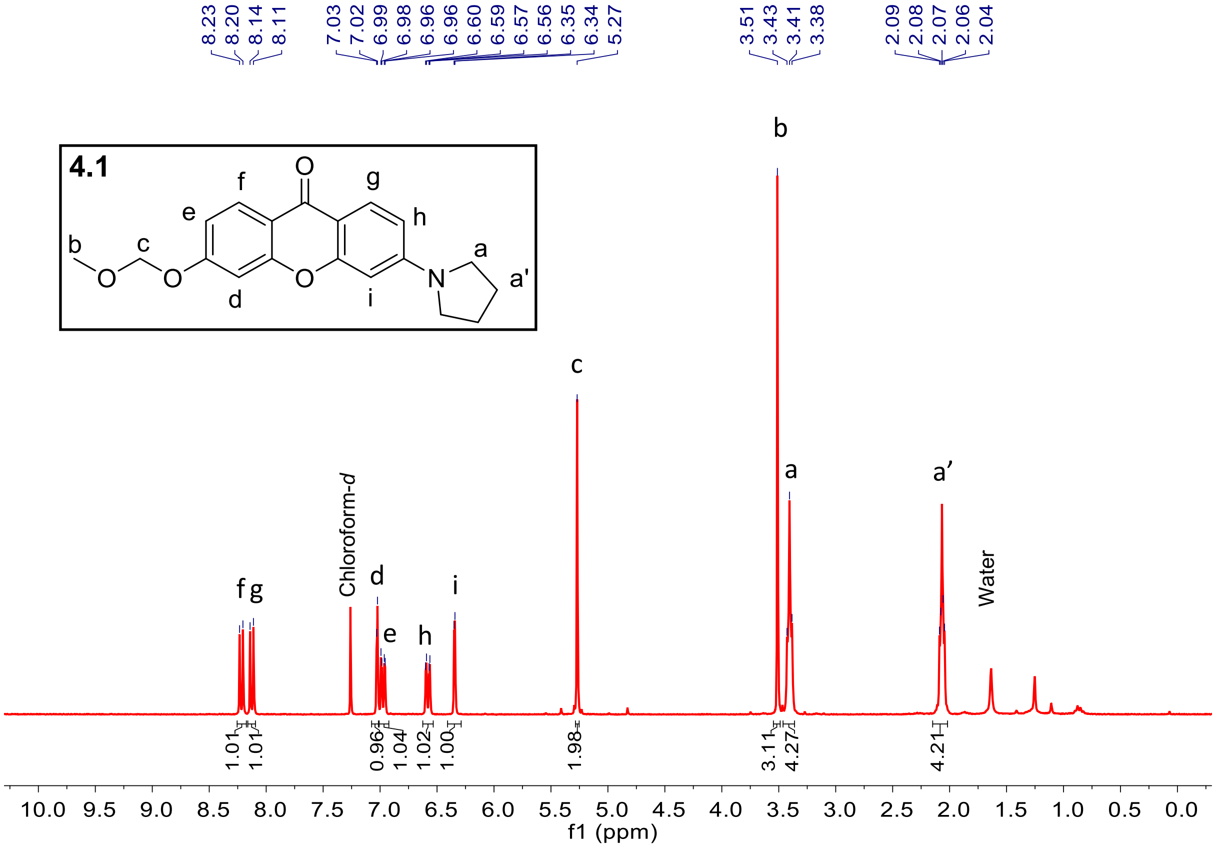
8.23
8.20
8.14
8.11
7.03
7.02
6.99
6.98
6.96
6.96
6.60
6.59
6.57
6.56
6.35
6.34
5.27
3.51
3.43
3.41
3.38
2.09
2.08
2.07
2.06
2.04
4.1
b
c
a
a'
d
e
f
g
h
i
Chloroform-*d*
Water
1.01
1.01
0.96
1.04
1.02
1.00
1.98
3.11
4.27
4.21
10.0 9.5 9.0 8.5 8.0 7.5 7.0 6.5 6.0 5.5 5.0 4.5 4.0 3.5 3.0 2.5 2.0 1.5 1.0 0.5 0.0
f1 (ppm)

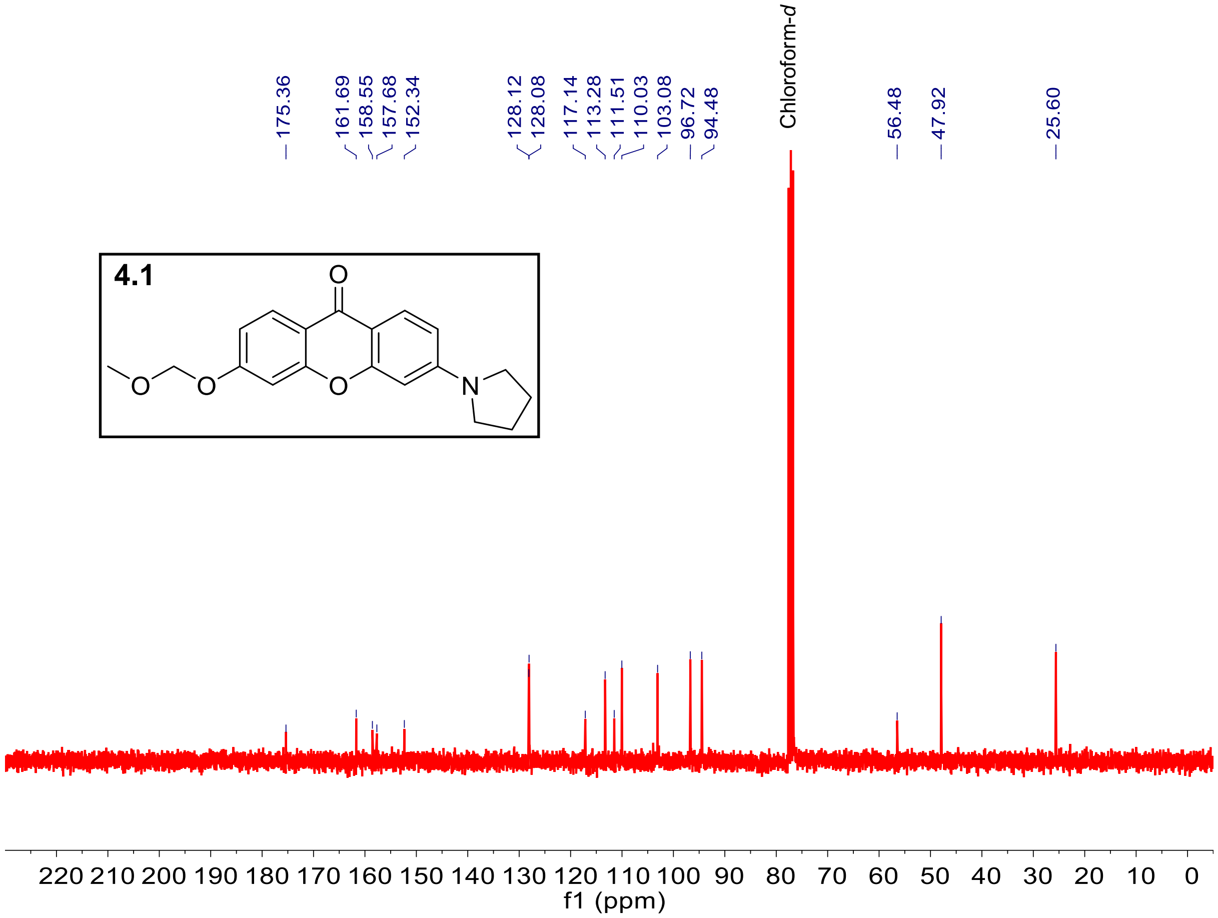
175.36
161.69
158.55
157.68
152.34
128.12
128.08
117.14
113.28
111.51
110.03
103.08
96.72
94.48
Chloroform-*d*
56.48
47.92
25.60
4.1
220 210 200 190 180 170 160 150 140 130 120 110 100 90 80 70 60 50 40 30 20 10 0
f1 (ppm)

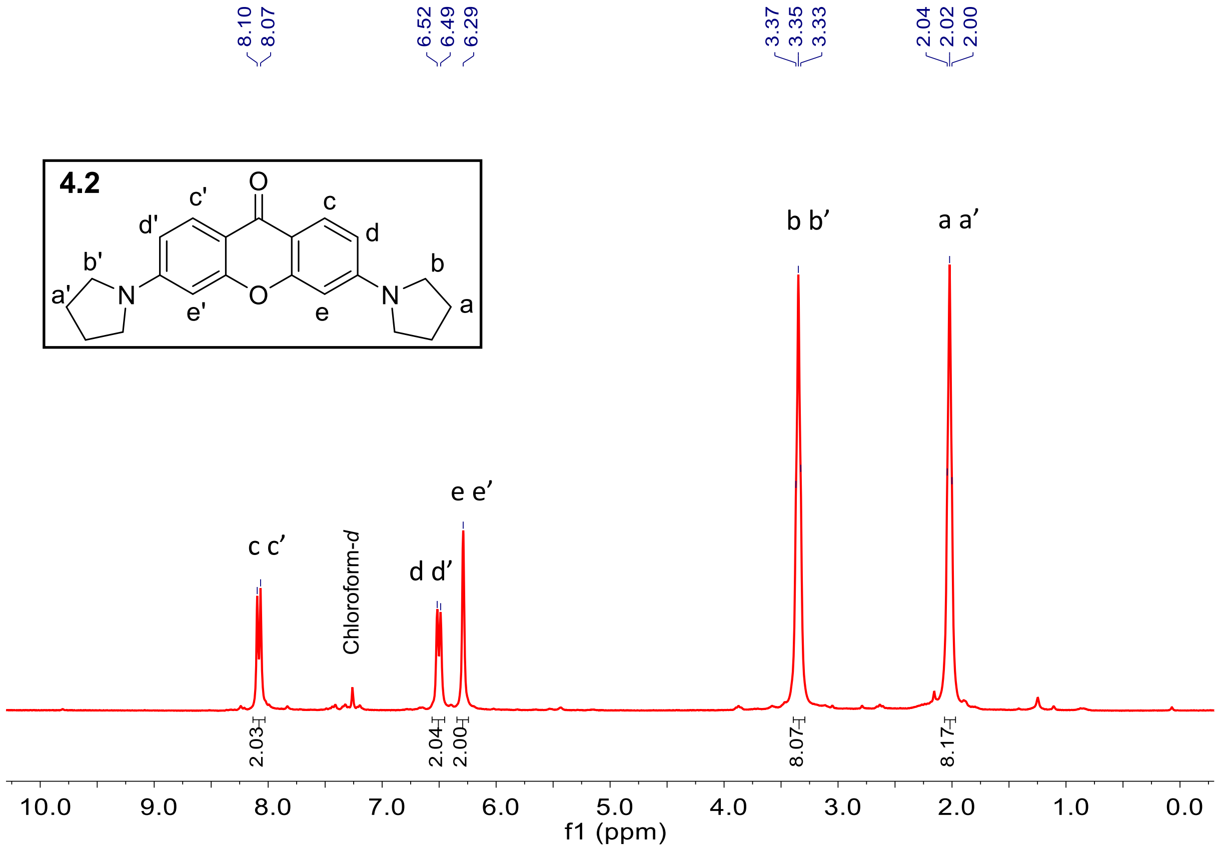
8.10
8.07
6.52
6.49
6.29
3.37
3.35
3.33
2.04
2.02
2.00
4.2
c'
O
c
d'
d
b'
b
a'
N
N
a
e'
O
e
b b’
a a’
e e’
c c’
Chloroform-d
d d’
2.03
2.04
2.00
8.07
8.17
10.0 9.0 8.0 7.0 6.0 5.0 4.0 3.0 2.0 1.0 0.0
f1 (ppm)

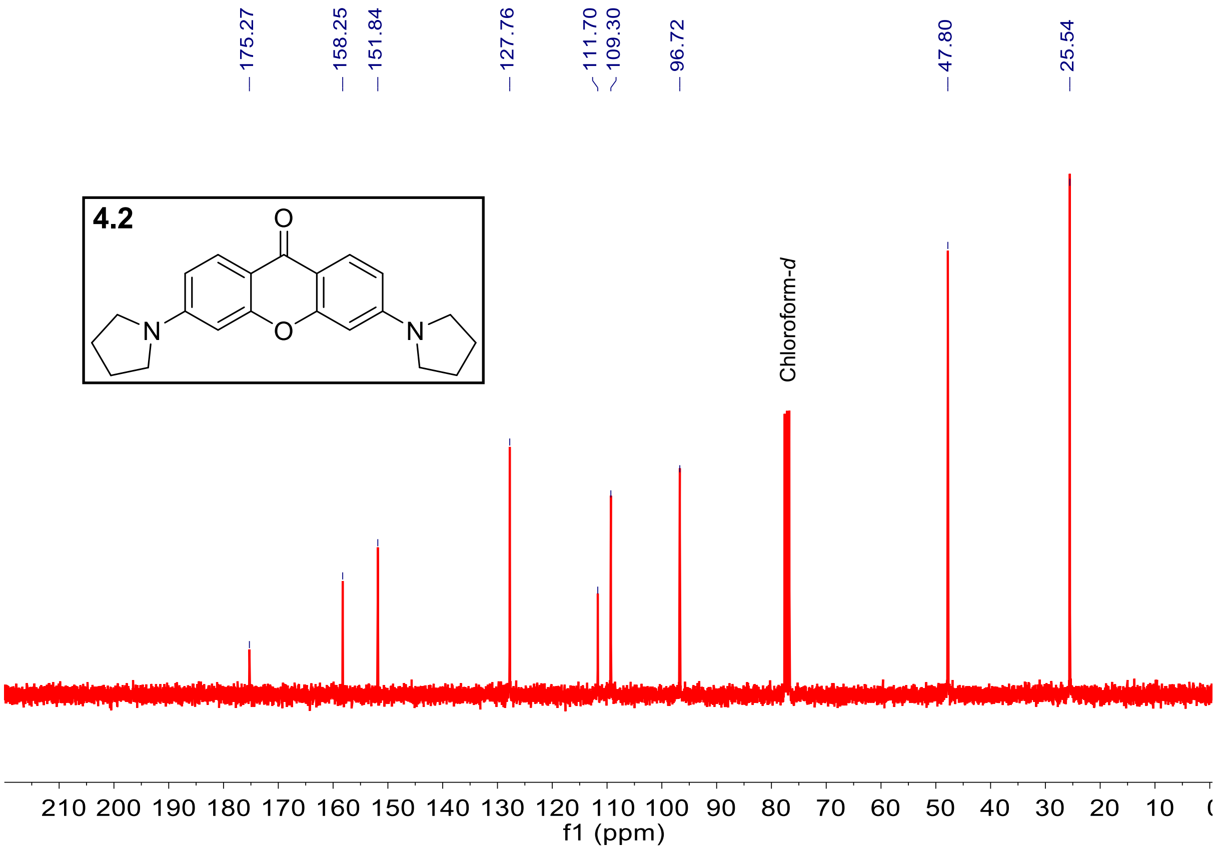
175.27
158.25
151.84
127.76
111.70
109.30
96.72
47.80
25.54
4.2
O
N
O
N
Chloroform-d
210 200 190 180 170 160 150 140 130 120 110 100 90 80 70 60 50 40 30 20 10
f1 (ppm)

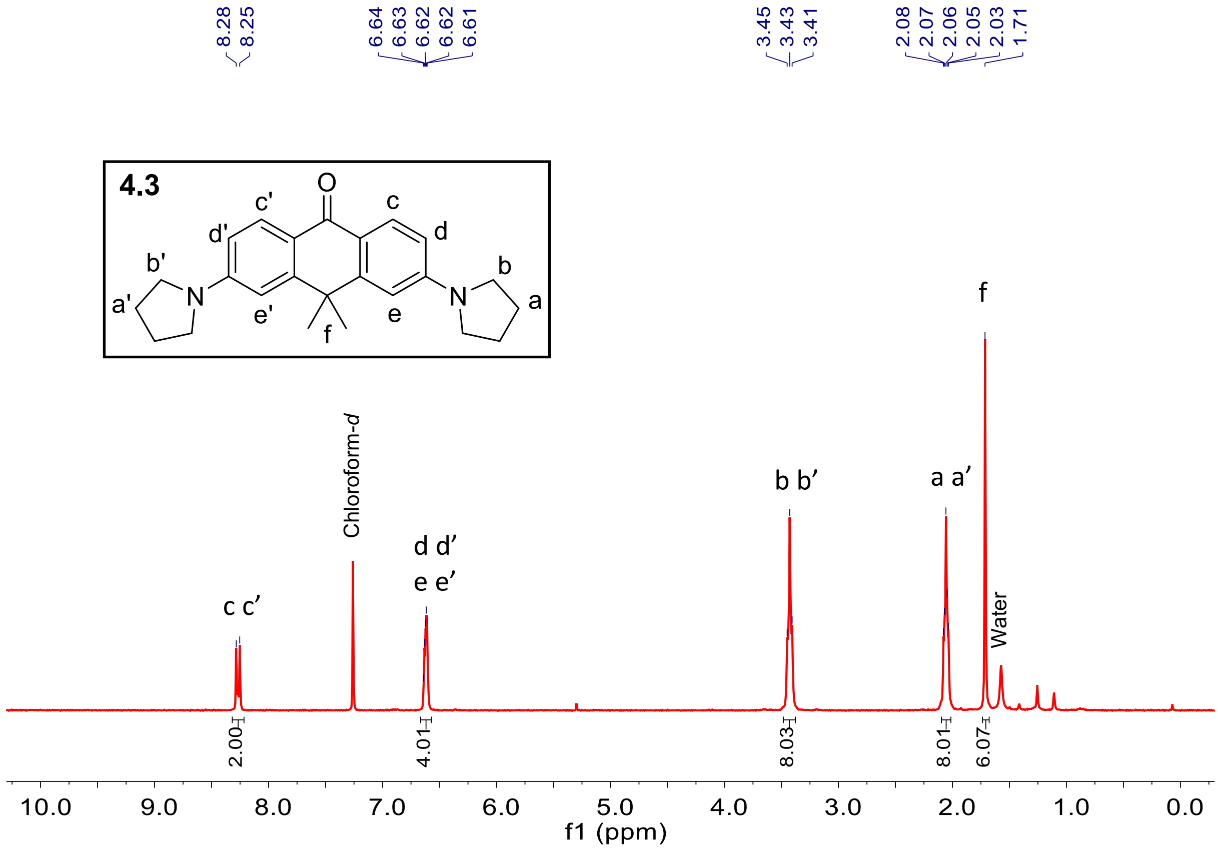
8.28
8.25
6.64
6.63
6.62
6.62
6.61
3.45
3.43
3.41
2.08
2.07
2.06
2.05
2.03
1.71
4.3
O
c'
c
d'
d
b'
b
a'
a
N
e'
e
f
Chloroform-d
c c'
d d'
e e'
b b'
a a'
Water
2.00
4.01
8.03
8.01
6.07
10.0 9.0 8.0 7.0 6.0 5.0 4.0 3.0 2.0 1.0 0.0
f1 (ppm)

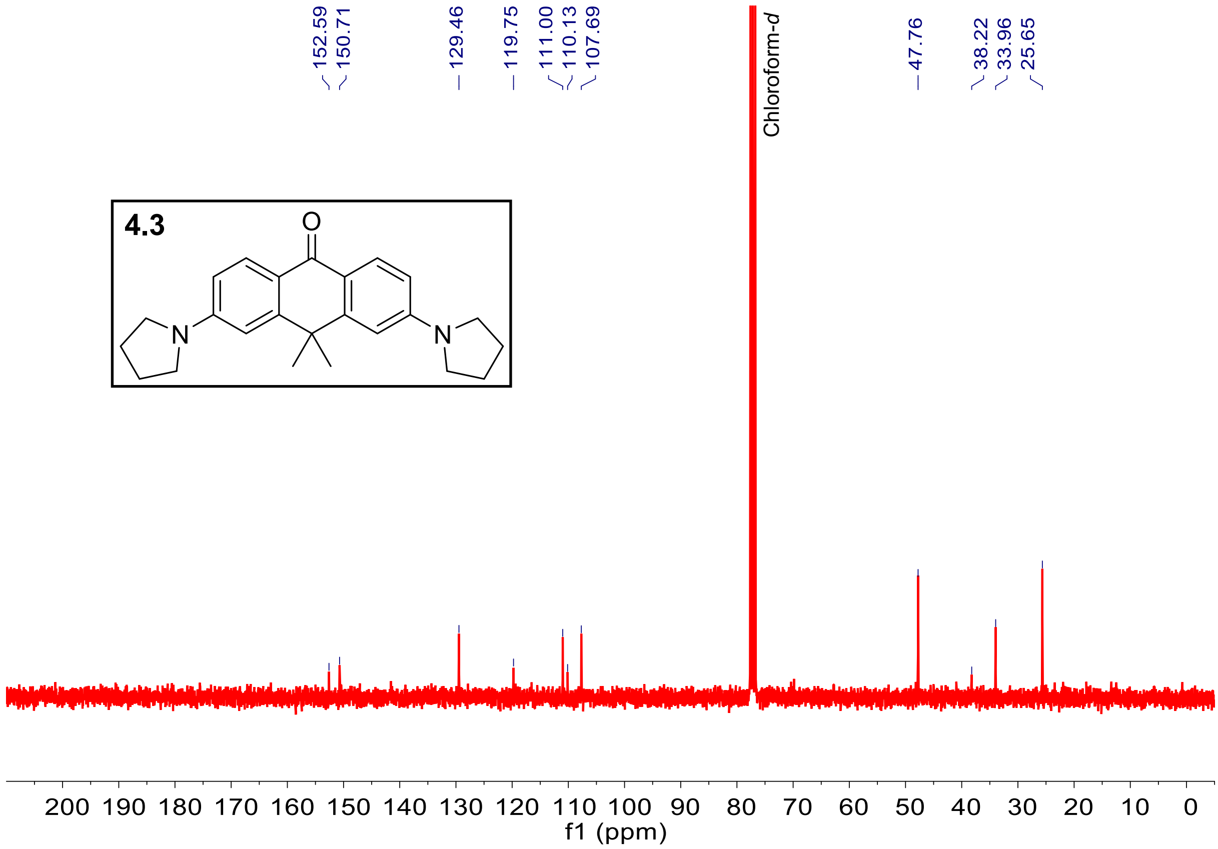
152.59
150.71
129.46
119.75
111.00
110.13
107.69
Chloroform-d
47.76
38.22
33.96
25.65
4.3
O
N
200 190 180 170 160 150 140 130 120 110 100 90 80 70 60 50 40 30 20 10 0
f1 (ppm)

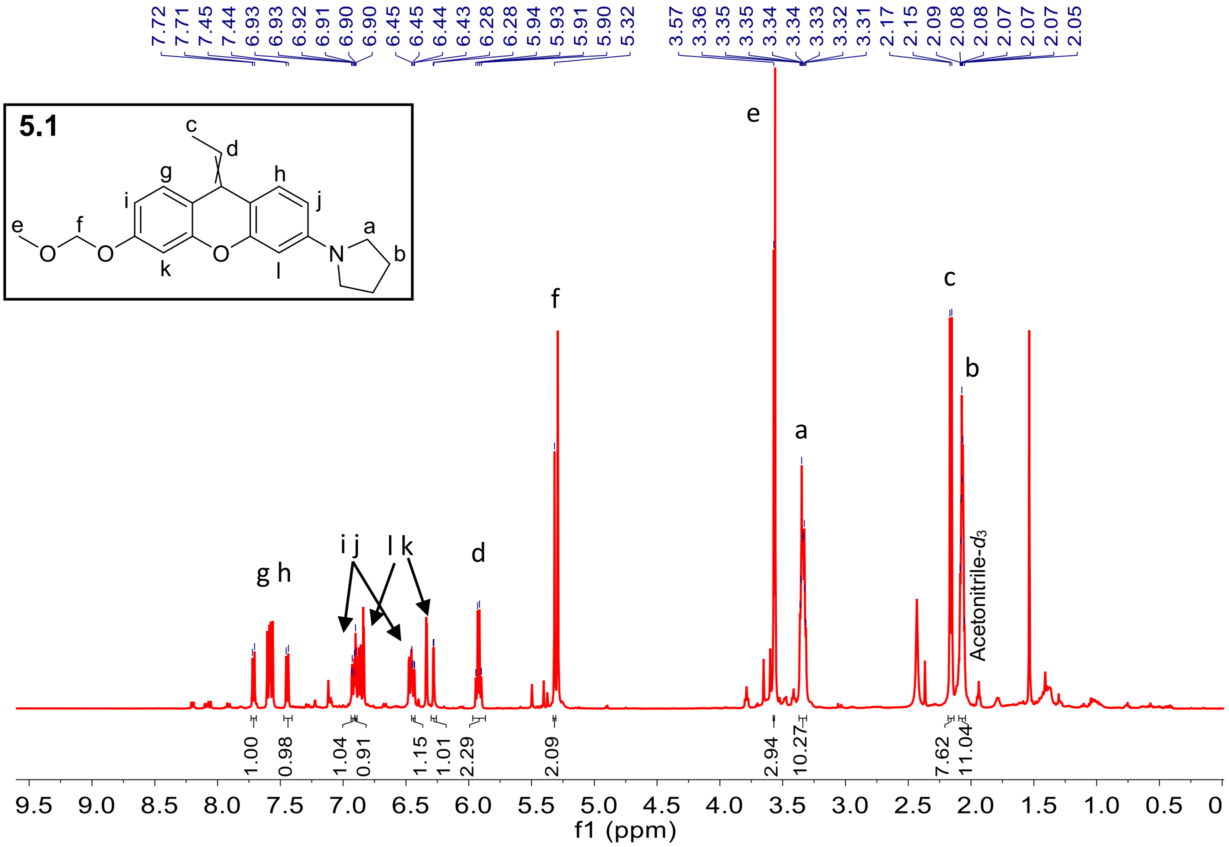
5.1
Acetonitrile-d_3
f1 (ppm)

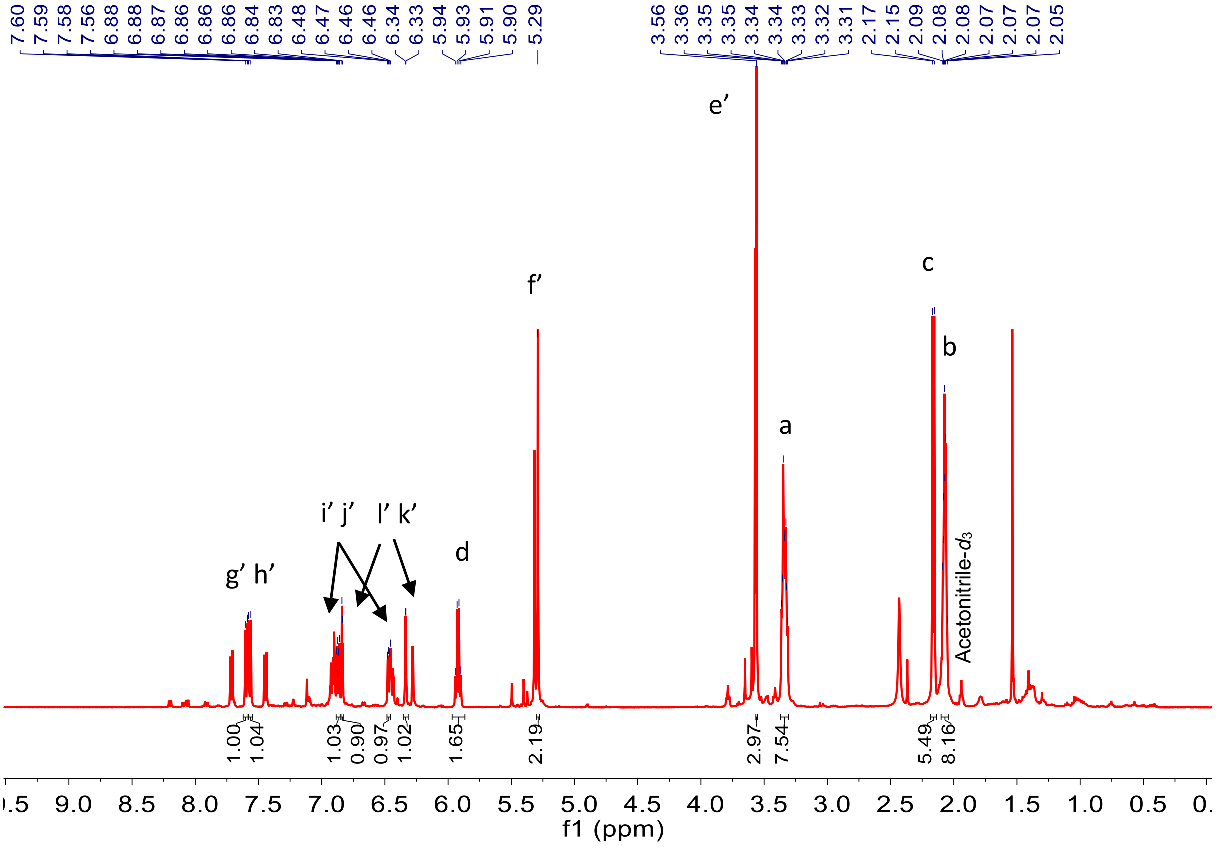
Acetonitrile-d_3
f1 (ppm)

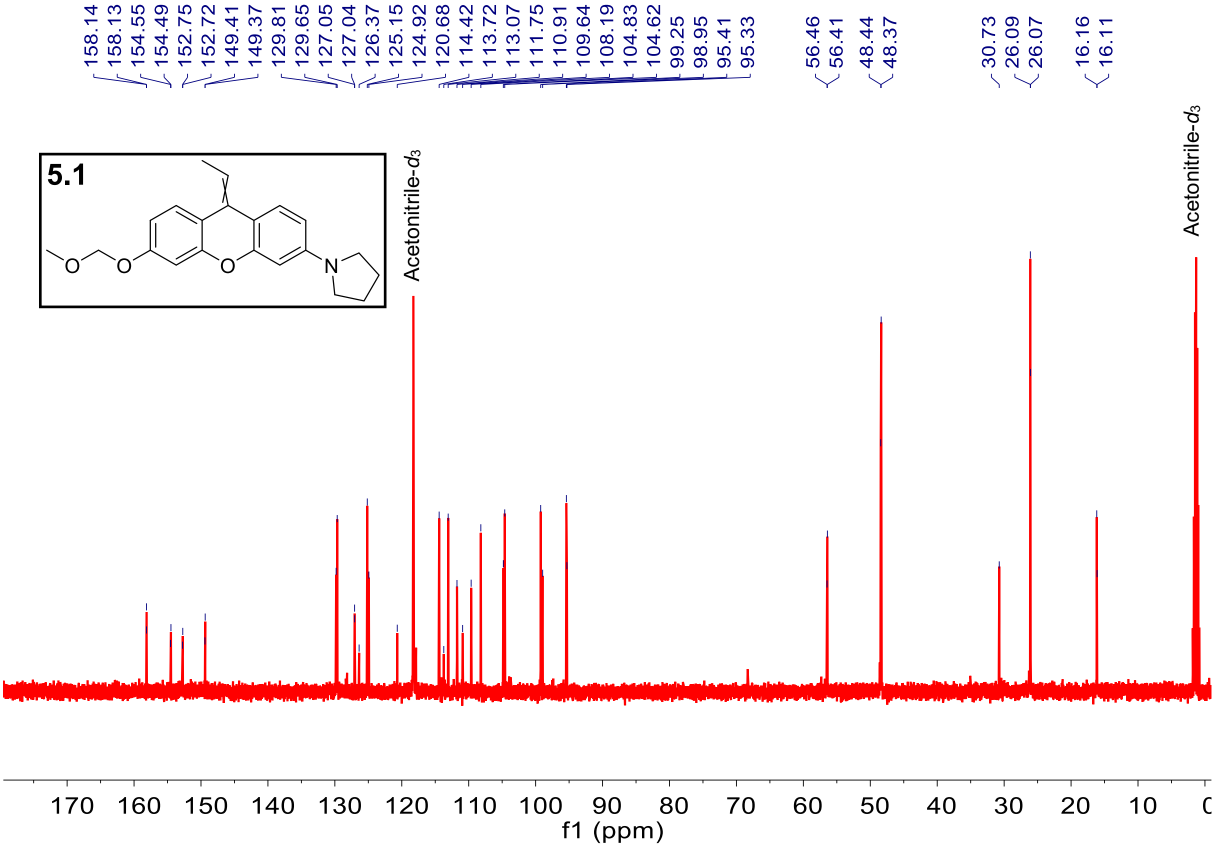
158.14
158.13
154.55
154.49
152.75
152.72
149.41
149.37
129.81
129.65
127.05
127.04
126.37
125.15
124.92
120.68
114.42
113.72
113.07
111.75
110.91
109.64
108.19
104.83
104.62
99.25
98.95
95.41
95.33
56.46
56.41
48.44
48.37
30.73
26.09
26.07
16.16
16.11
5.1
Acetonitrile-d3
Acetonitrile-d3
170 160 150 140 130 120 110 100 90 80 70 60 50 40 30 20 10 0
f1 (ppm)

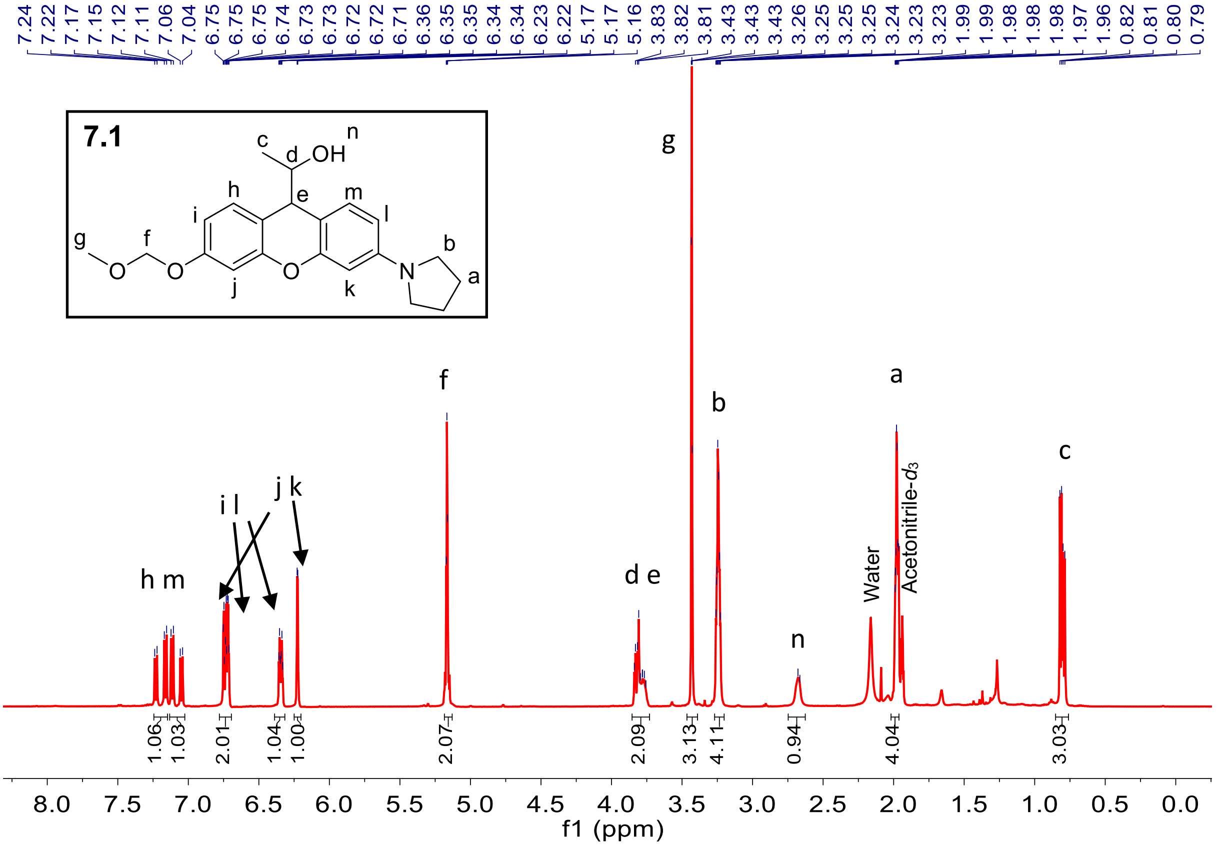
7.1
f1 (ppm)
Water
Acetonitrile-d_3

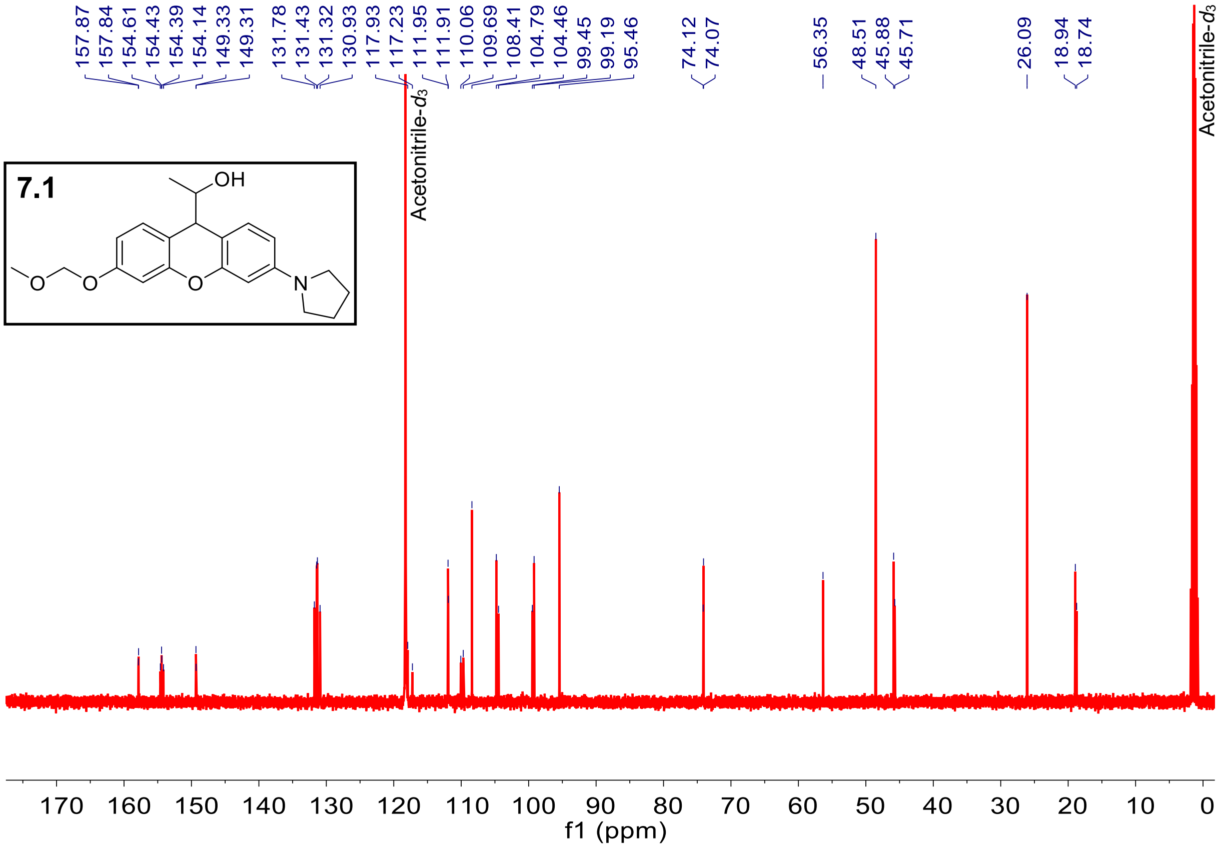
7.1
f1 (ppm)
Acetonitrile-d_3

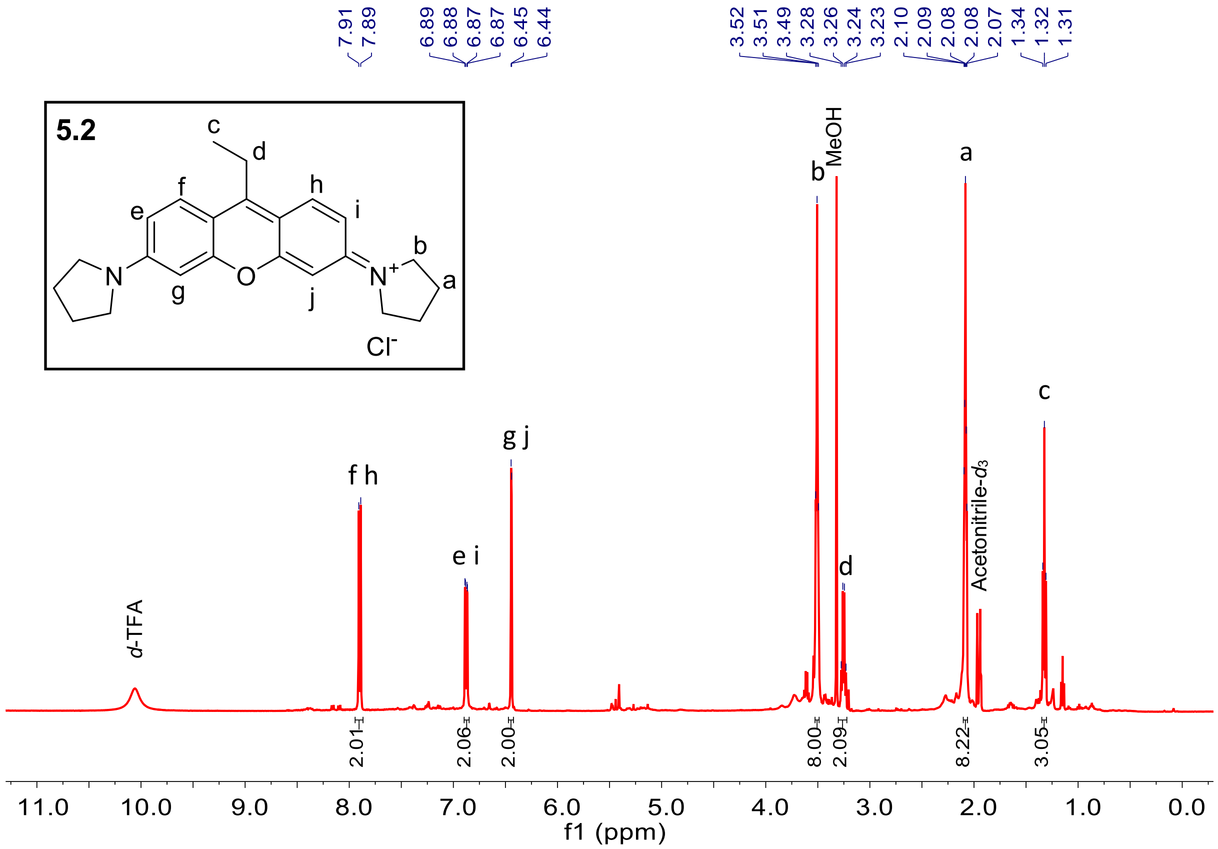
7.91
7.89
6.89
6.88
6.87
6.87
6.45
6.44
3.52
3.51
3.49
3.28
3.26
3.24
3.23
2.10
2.09
2.08
2.08
2.07
1.34
1.32
1.31
5.2
c
d
e
f
h
i
b
N
N+
O
g
j
a
Cl-
b
MeOH
a
c
g j
f h
e i
d
Acetonitrile-d3
d-TFA
2.01
2.06
2.00
8.00
2.09
8.22
3.05
11.0
10.0
9.0
8.0
7.0
6.0
5.0
4.0
3.0
2.0
1.0
0.0
f1 (ppm)

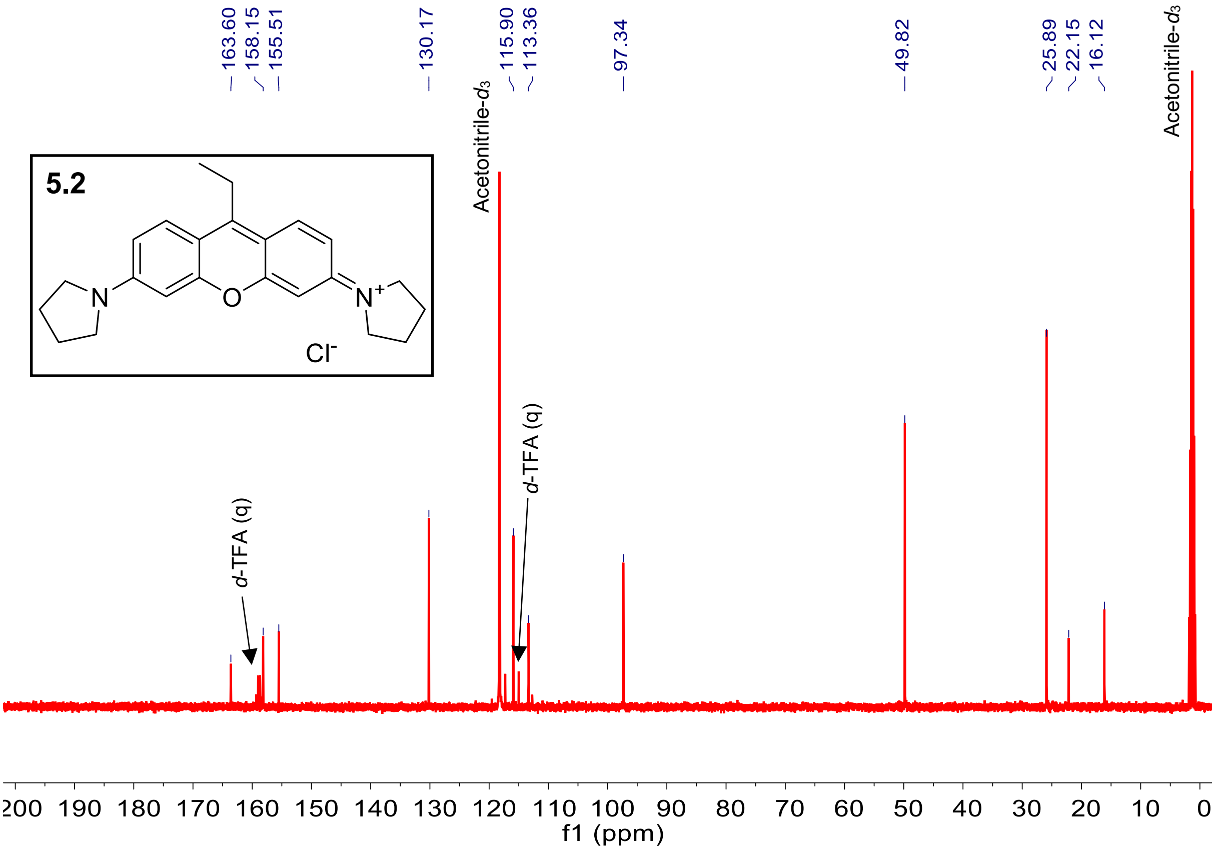
163.60
158.15
155.51
130.17
115.90
113.36
97.34
49.82
25.89
22.15
16.12
Acetonitrile-d3
Acetonitrile-d3
5.2
N
N+
O
Cl-
d-TFA (q)
d-TFA (q)
200
190
180
170
160
150
140
130
120
110
100
90
80
70
60
50
40
30
20
10
0
f1 (ppm)

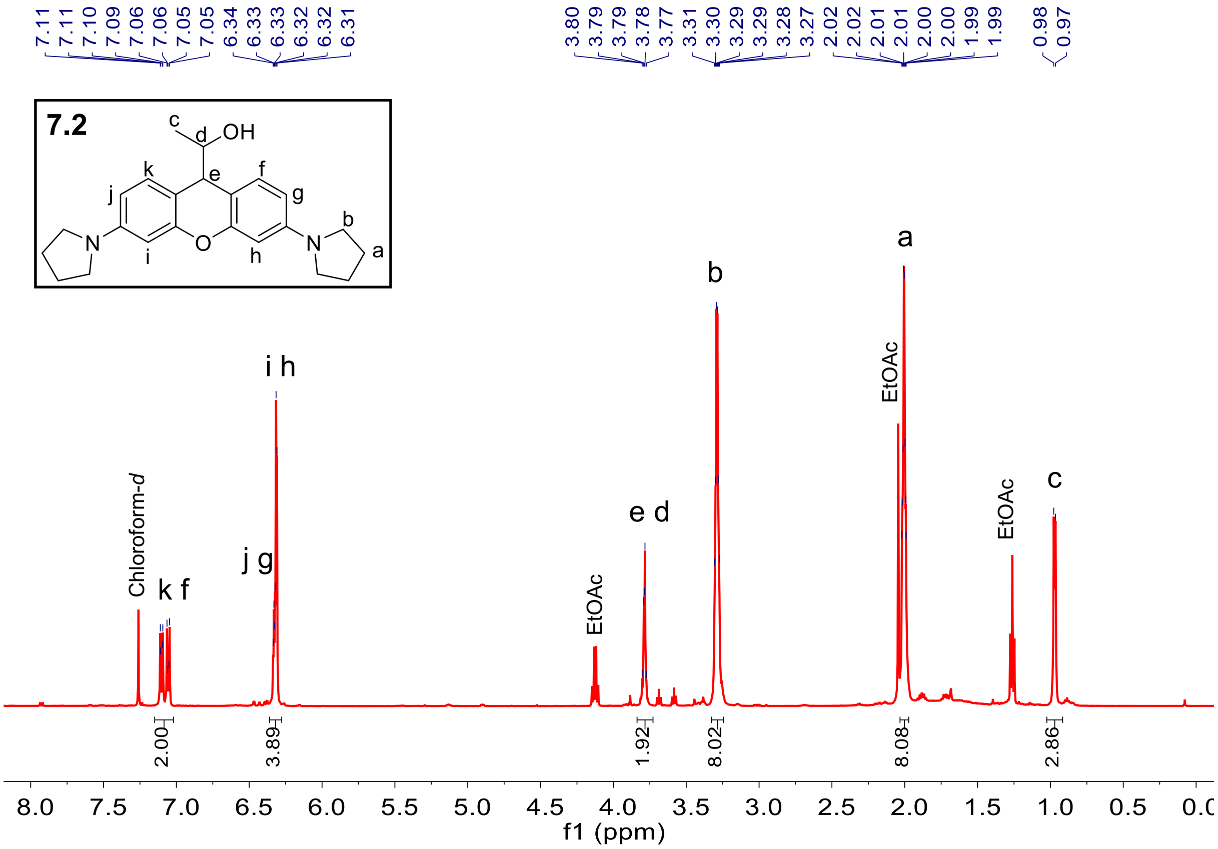
7.11
7.11
7.10
7.09
7.06
7.06
7.05
7.05
6.34
6.33
6.33
6.32
6.32
6.31
3.80
3.79
3.79
3.78
3.77
3.31
3.30
3.29
3.29
3.28
3.27
2.02
2.02
2.01
2.01
2.00
2.00
1.99
1.99
0.98
0.97
7.2
OH
Chloroform-d
k f
j g
i h
EtOAc
e d
b
a
EtOAc
c
2.00
3.89
1.92
8.02
8.08
2.86
f1 (ppm)

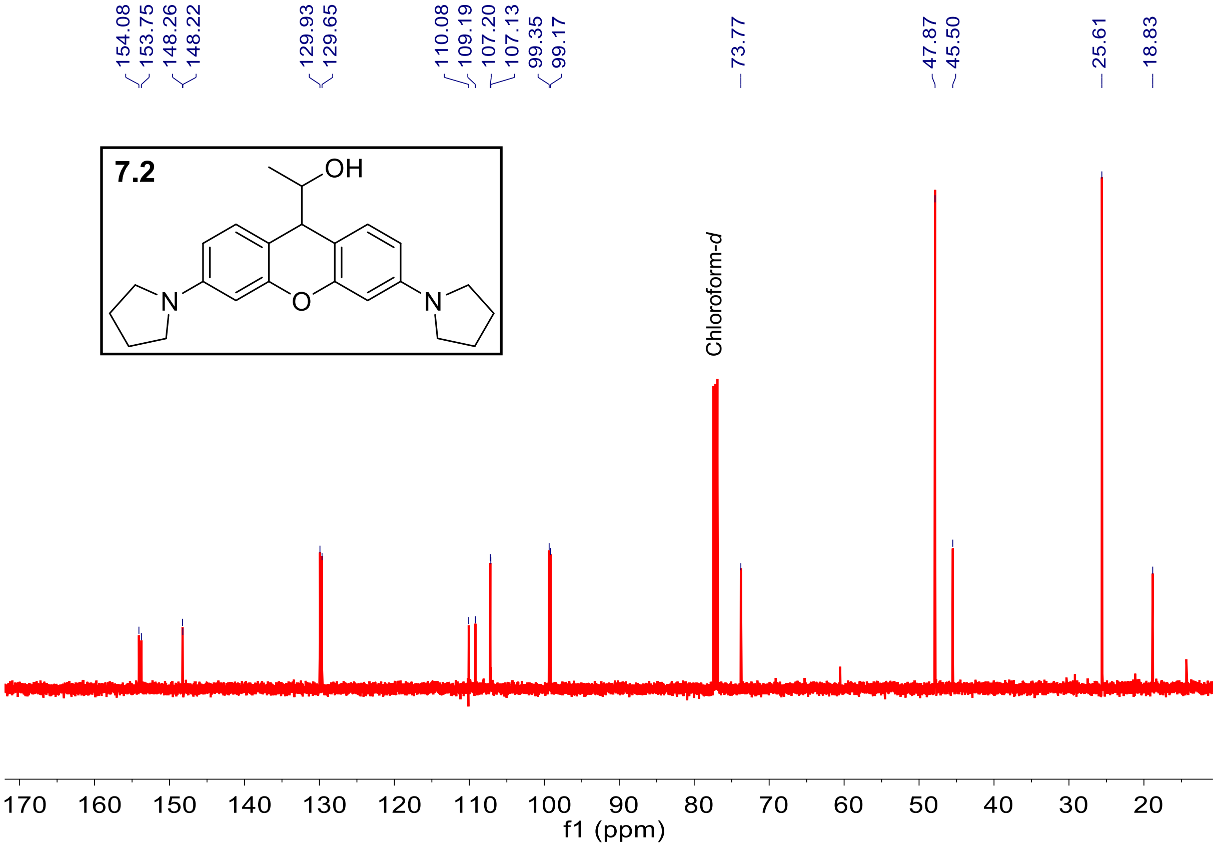
154.08
153.75
148.26
148.22
129.93
129.65
110.08
109.19
107.20
107.13
99.35
99.17
73.77
47.87
45.50
25.61
18.83
7.2
OH
Chloroform-d
f1 (ppm)

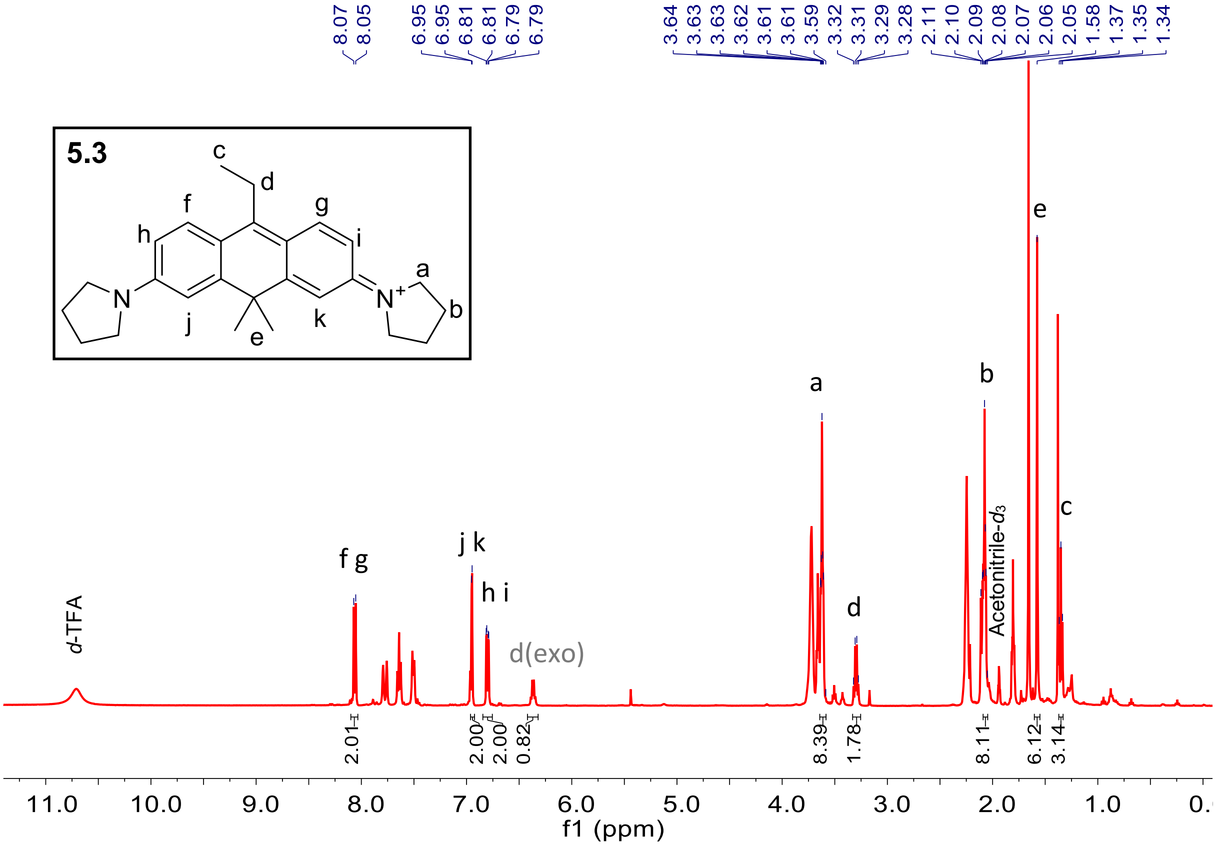
8.07
8.05
6.95
6.95
6.81
6.81
6.79
6.79
3.64
3.63
3.63
3.62
3.61
3.61
3.59
3.32
3.31
3.29
3.28
2.11
2.10
2.09
2.08
2.07
2.06
2.05
1.58
1.37
1.35
1.34
5.3
c
d
f
g
h
i
a
b
j
k
e
N
N+
d-TFA
f g
j k
h i
d(exo)
a
d
b
Acetonitrile-d3
e
c
2.01
2.00
2.00
0.82
8.39
1.78
8.11
6.12
3.14
11.0
10.0
9.0
8.0
7.0
6.0
5.0
4.0
3.0
2.0
1.0
f1 (ppm)

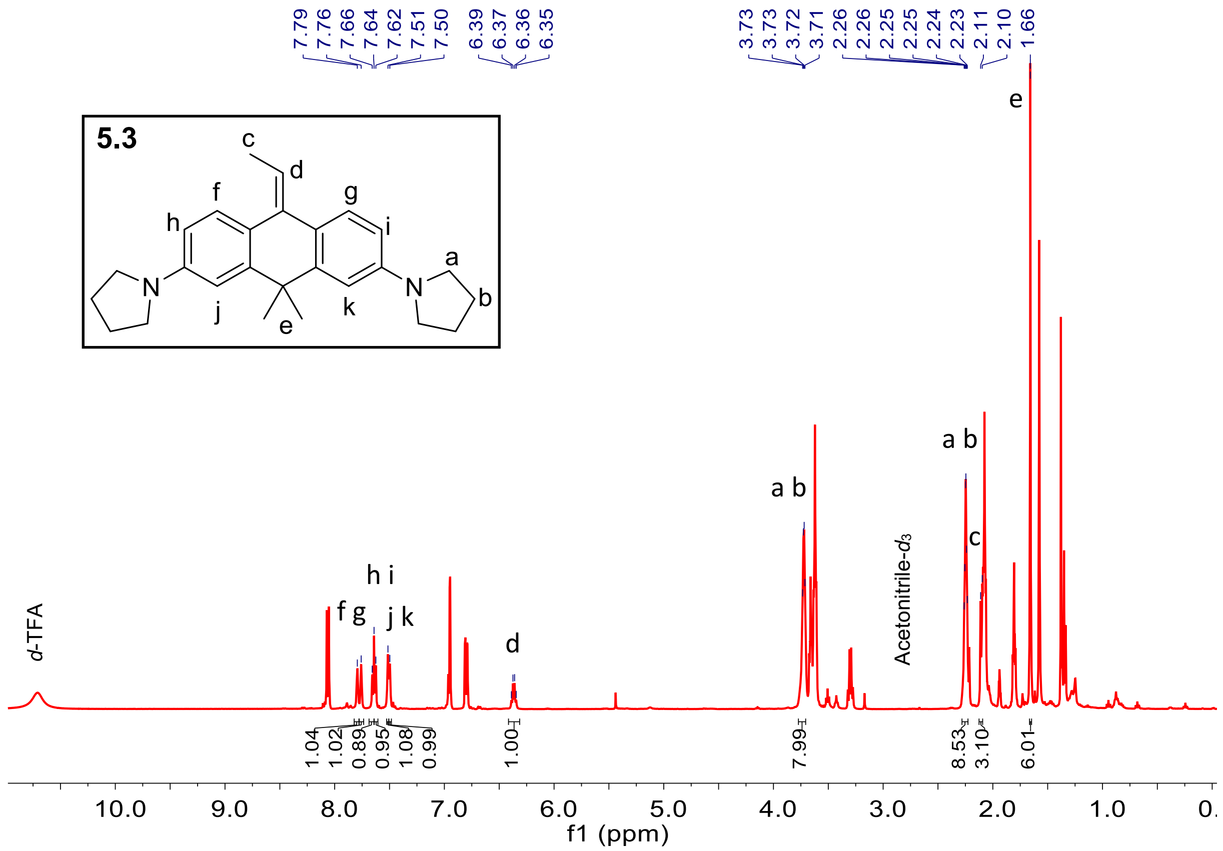
7.79 7.76 7.66 7.64 7.62 7.51 7.50 6.39 6.37 6.36 6.35
3.73 3.73 3.72 3.71 2.26 2.26 2.25 2.25 2.24 2.23 2.11 2.10 1.66
5.3
a b c d e f g h i j k
N
d-TFA
f g
h i
j k
d
a b
Acetonitrile-d_3
c
e
1.04 1.02 0.89 0.95 1.08 0.99 1.00 7.99 8.53 3.10 6.01
10.0 9.0 8.0 7.0 6.0 5.0 4.0 3.0 2.0 1.0 0.
f1 (ppm)

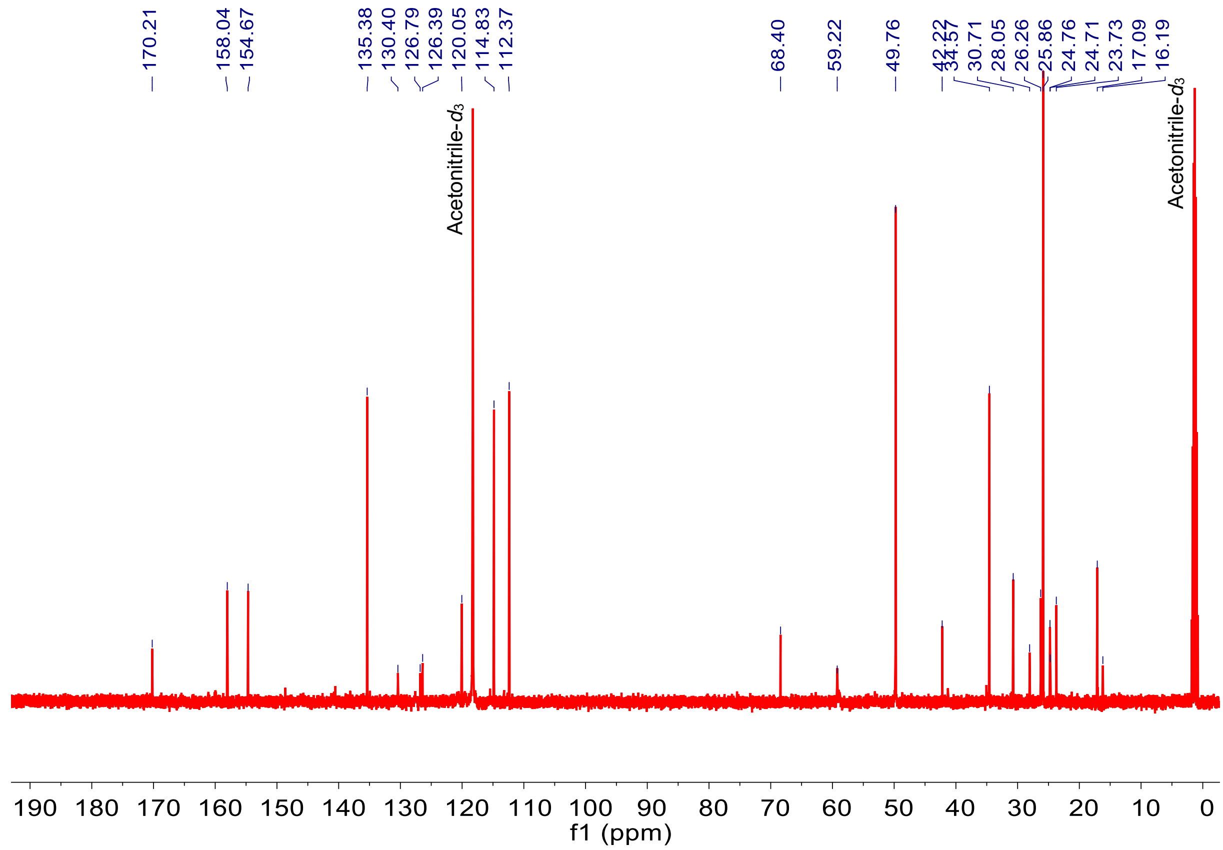
170.21 158.04 154.67 135.38 130.40 126.79 126.39 120.05 114.83 112.37
68.40 59.22 49.76 42.22 34.57 30.71 28.05 26.26 25.86 24.76 24.71 23.73 17.09 16.19
Acetonitrile-d_3
Acetonitrile-d_3
190 180 170 160 150 140 130 120 110 100 90 80 70 60 50 40 30 20 10 0
f1 (ppm)

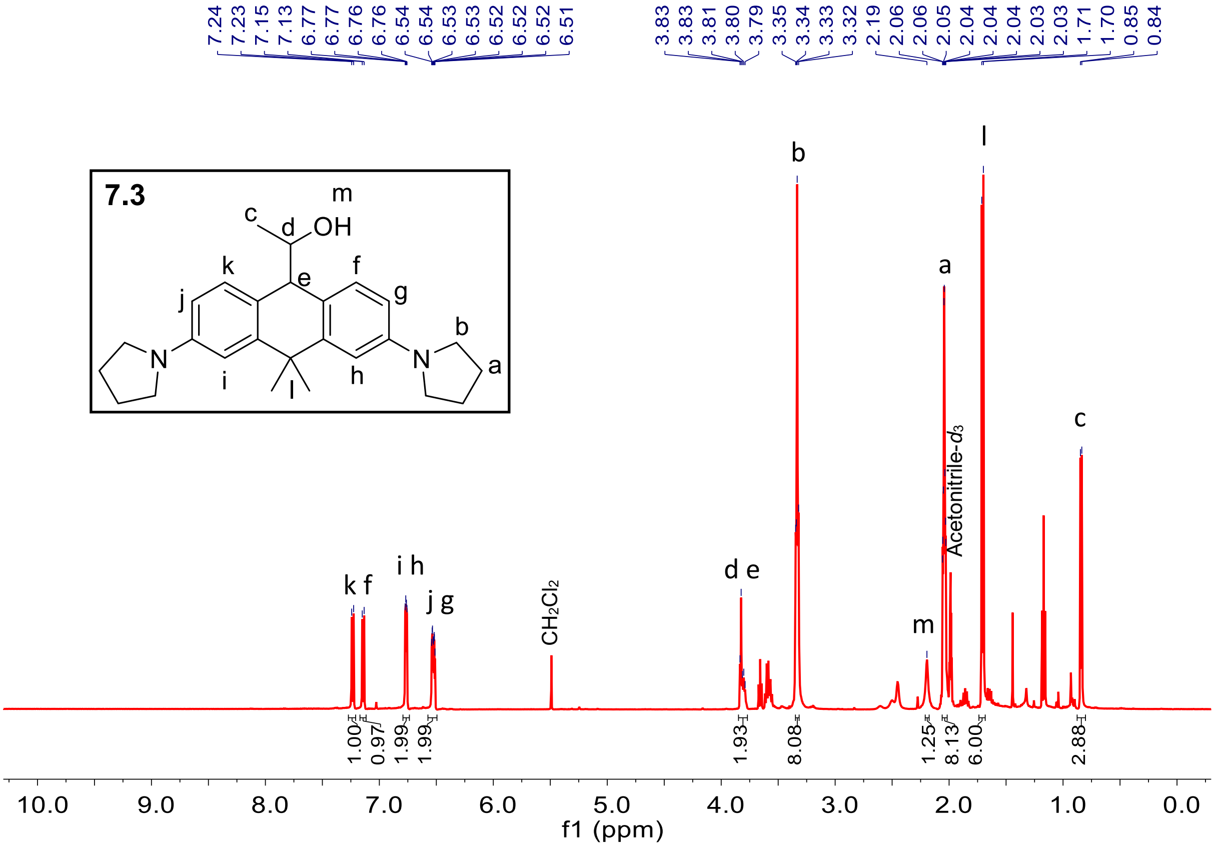
7.24 7.23 7.15 7.13 6.77 6.77 6.76 6.76 6.54 6.54 6.53 6.53 6.52 6.52 6.52 6.51
3.83 3.83 3.81 3.80 3.79 3.35 3.34 3.33 3.32 2.19 2.06 2.06 2.05 2.04 2.04 2.04 2.03 2.03 1.71 1.70 0.85 0.84
7.3
c d m OH
k e f g
j b
N N a
i l h
b
l
a
Acetonitrile-d3
c
k f
i h
j g
CH2Cl2
d e
m
1.00 0.97 1.99 1.99 1.93 8.08 1.25 8.13 6.00 2.88
10.0 9.0 8.0 7.0 6.0 5.0 4.0 3.0 2.0 1.0 0.0
f1 (ppm)

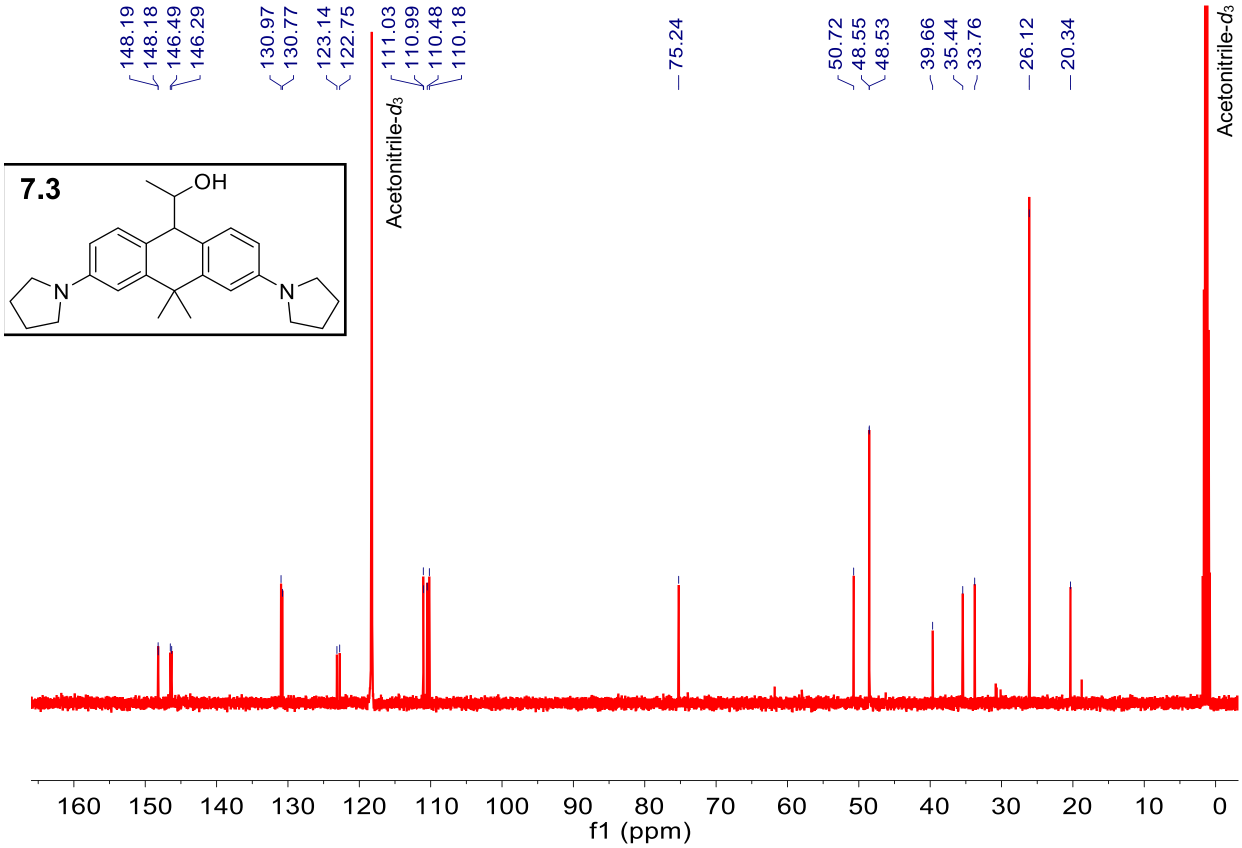
148.19 148.18 146.49 146.29 130.97 130.77 123.14 122.75 111.03 110.99 110.48 110.18 75.24 50.72 48.55 48.53 39.66 35.44 33.76 26.12 20.34
Acetonitrile-d3
7.3
OH
N N
Acetonitrile-d3
160 150 140 130 120 110 100 90 80 70 60 50 40 30 20 10 0
f1 (ppm)

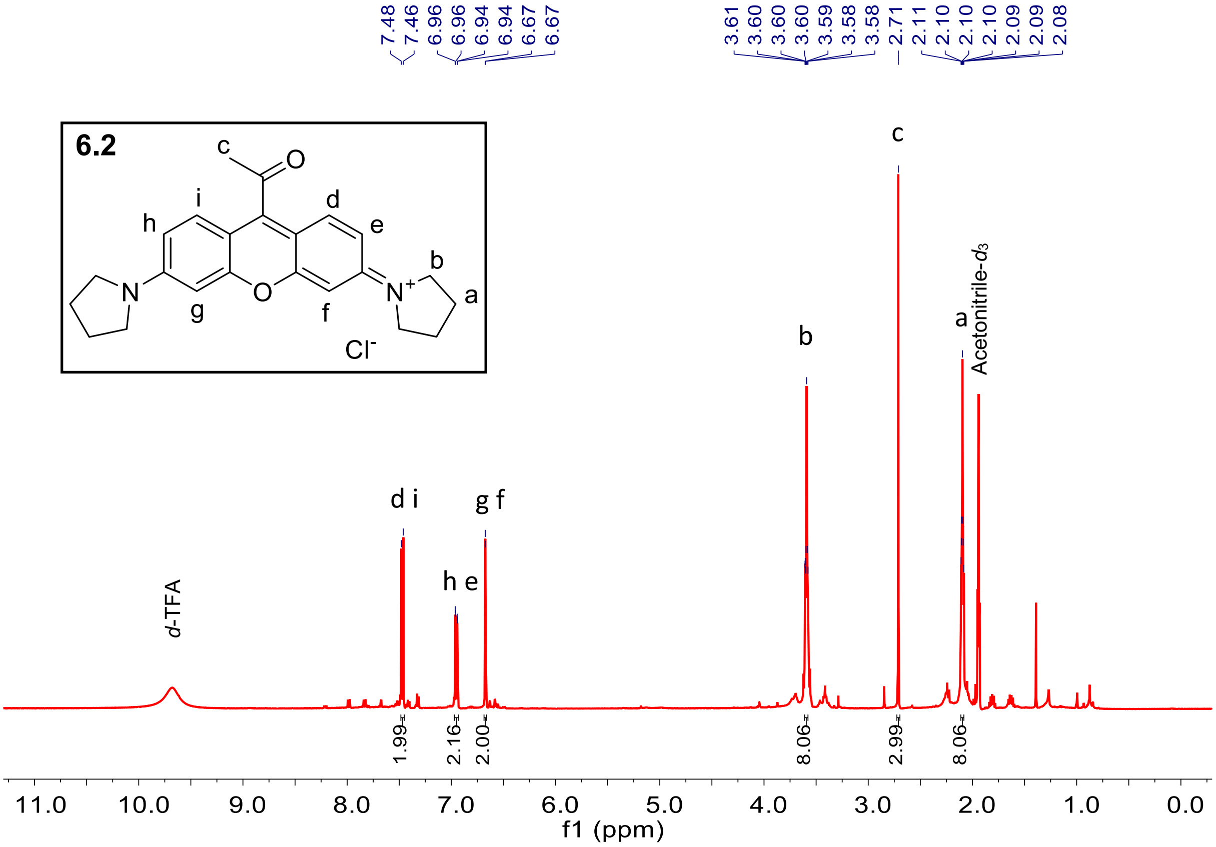
7.48
7.46
6.96
6.96
6.94
6.94
6.67
6.67
3.61
3.60
3.60
3.60
3.59
3.58
3.58
2.71
2.11
2.10
2.10
2.10
2.09
2.09
2.08
6.2
c
i
d
h
e
b
a
N
O
N+
g
f
Cl-
Acetonitrile-d3
d-TFA
d i
h e
g f
1.99
2.16
2.00
8.06
2.99
8.06
11.0
10.0
9.0
8.0
7.0
6.0
5.0
4.0
3.0
2.0
1.0
0.0
f1 (ppm)

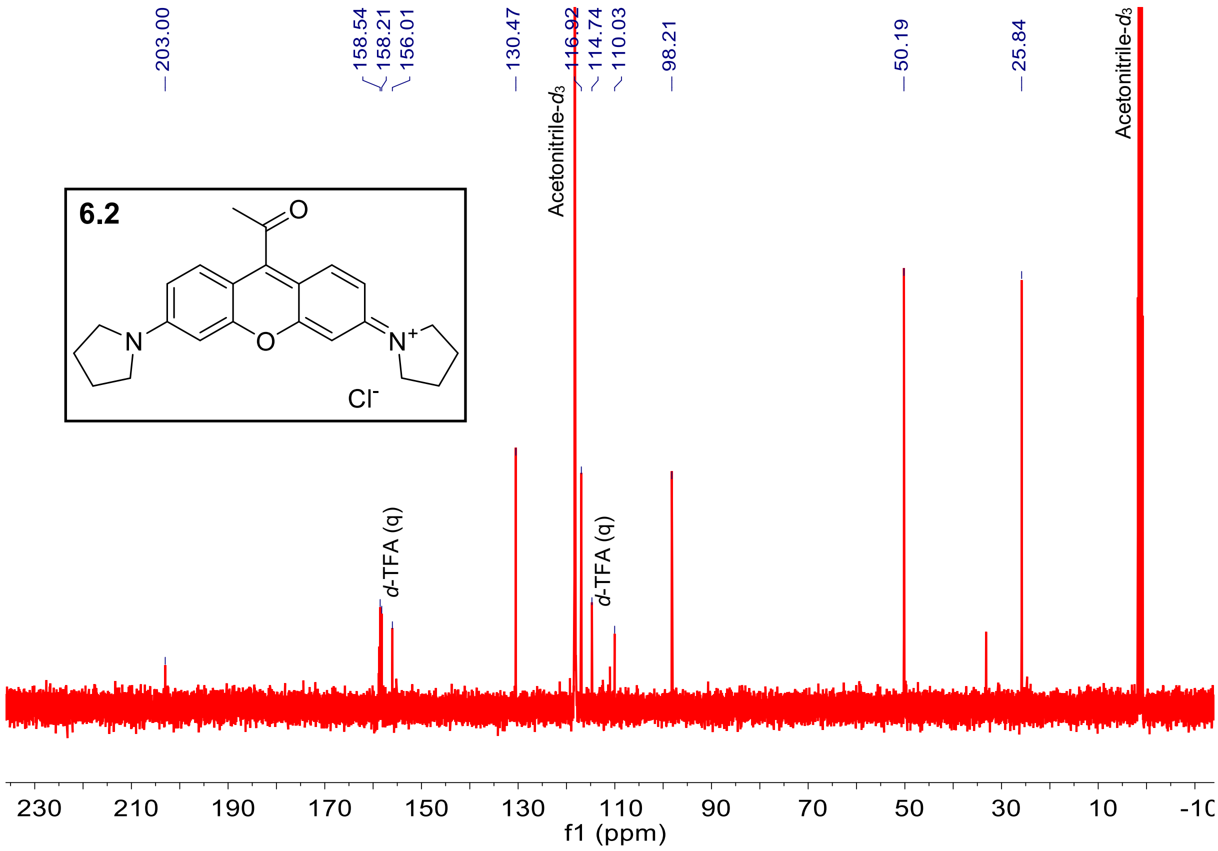
203.00
158.54
158.21
156.01
130.47
116.92
114.74
110.03
98.21
50.19
25.84
Acetonitrile-d3
Acetonitrile-d3
6.2
O
N
O
N+
Cl-
d-TFA (q)
d-TFA (q)
230
210
190
170
150
130
110
90
70
50
30
10
f1 (ppm)

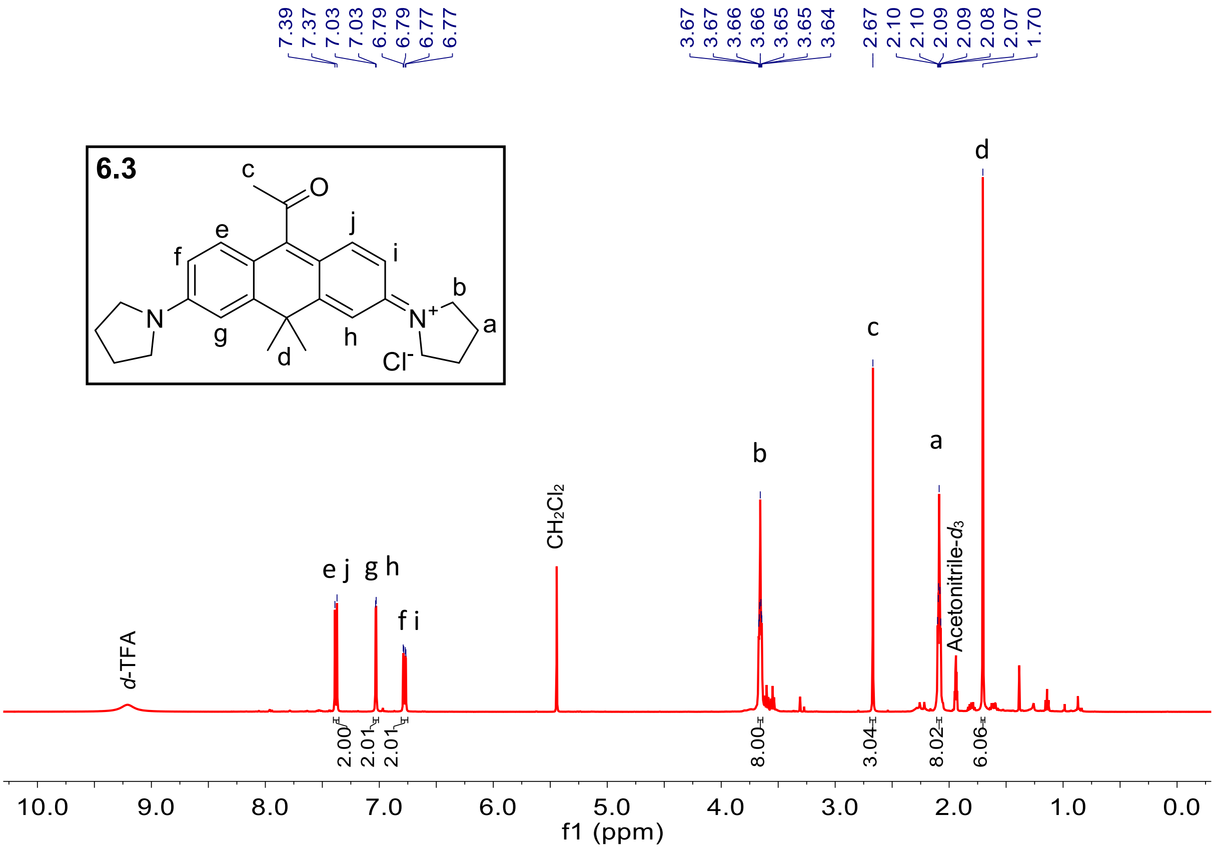
7.39
7.37
7.03
7.03
6.79
6.79
6.77
6.77
3.67
3.67
3.66
3.66
3.65
3.65
3.64
2.67
2.10
2.10
2.09
2.09
2.08
2.07
1.70
6.3
c
O
e
j
i
f
b
N
N+
a
g
d
h
Cl-
d
c
b
a
CH2Cl2
Acetonitrile-d3
e j g h
f i
d-TFA
2.00
2.01
2.01
8.00
3.04
8.02
6.06
10.0 9.0 8.0 7.0 6.0 5.0 4.0 3.0 2.0 1.0 0.0
f1 (ppm)

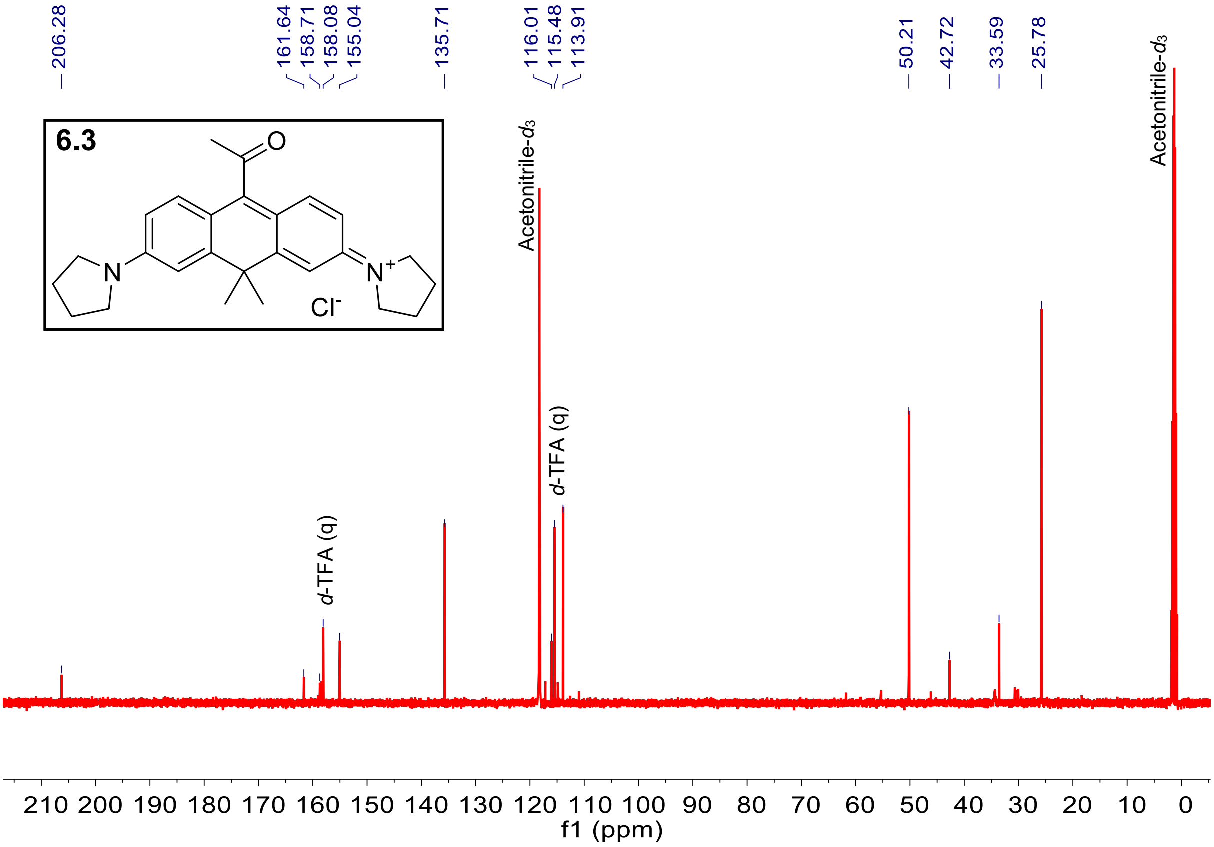
206.28
161.64
158.71
158.08
155.04
135.71
116.01
115.48
113.91
50.21
42.72
33.59
25.78
Acetonitrile-d3
6.3
O
N
N+
Cl-
Acetonitrile-d3
d-TFA (q)
d-TFA (q)
210 200 190 180 170 160 150 140 130 120 110 100 90 80 70 60 50 40 30 20 10 0
f1 (ppm)

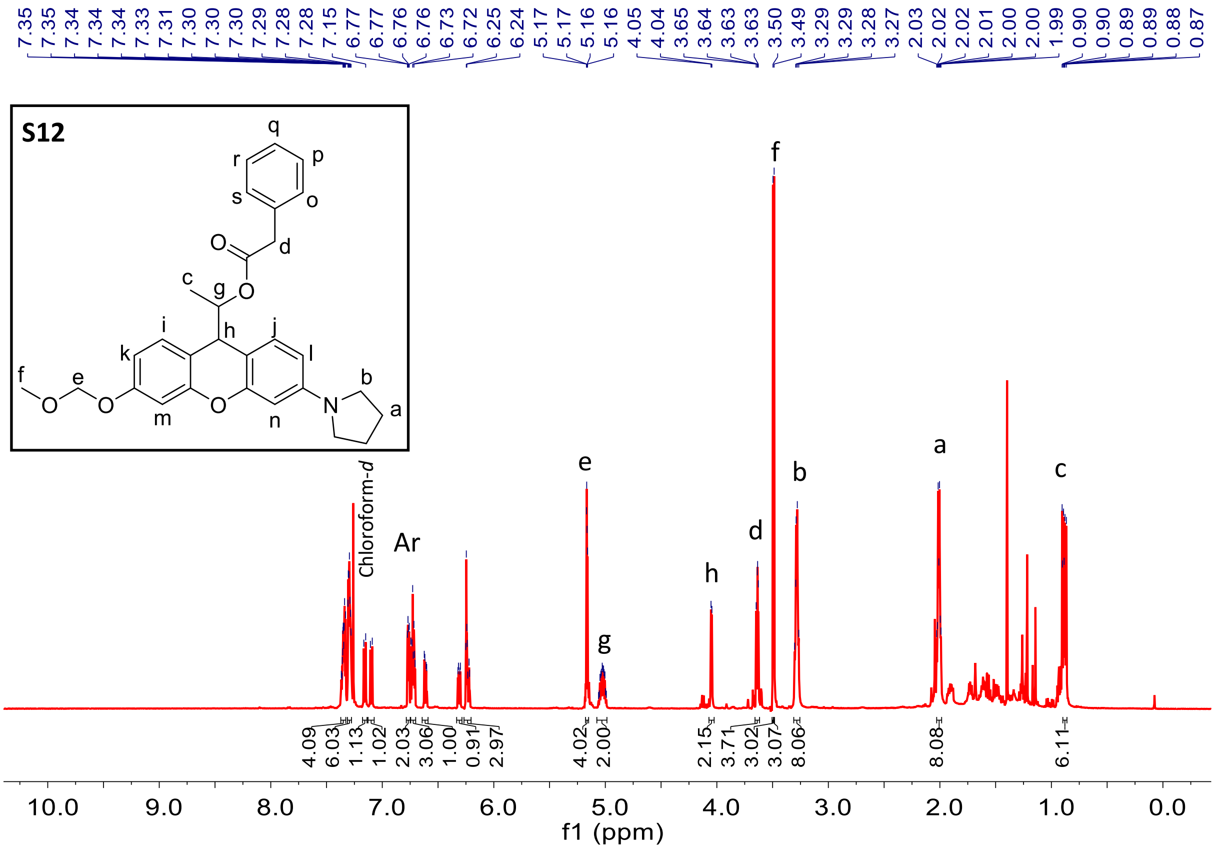

7.35 7.35 7.34 7.34 7.34 7.33 7.31 7.30 7.30 7.30 7.29 7.28 7.28 7.15 6.77 6.77 6.76 6.76 6.73 6.72 6.25 6.24 5.17 5.17 5.16 5.16 4.05 4.04 3.65 3.64 3.63 3.63 3.50 3.49 3.29 3.29 3.28 3.27 2.03 2.02 2.02 2.01 2.00 2.00 1.99 0.90 0.90 0.89 0.89 0.88 0.87
S12
q
r
p
s
o
O
d
c
g
O
i
h
j
k
l
f
e
b
O
O
N
a
m
O
n
Chloroform-d
Ar
e
g
h
d
f
b
a
c
4.09 6.03 1.13 1.02 2.03 3.06 1.00 0.91 2.97
4.02 2.00
2.15 3.71 3.02 3.07 8.06
8.08
6.11
10.0 9.0 8.0 7.0 6.0 5.0 4.0 3.0 2.0 1.0 0.0
f1 (ppm)

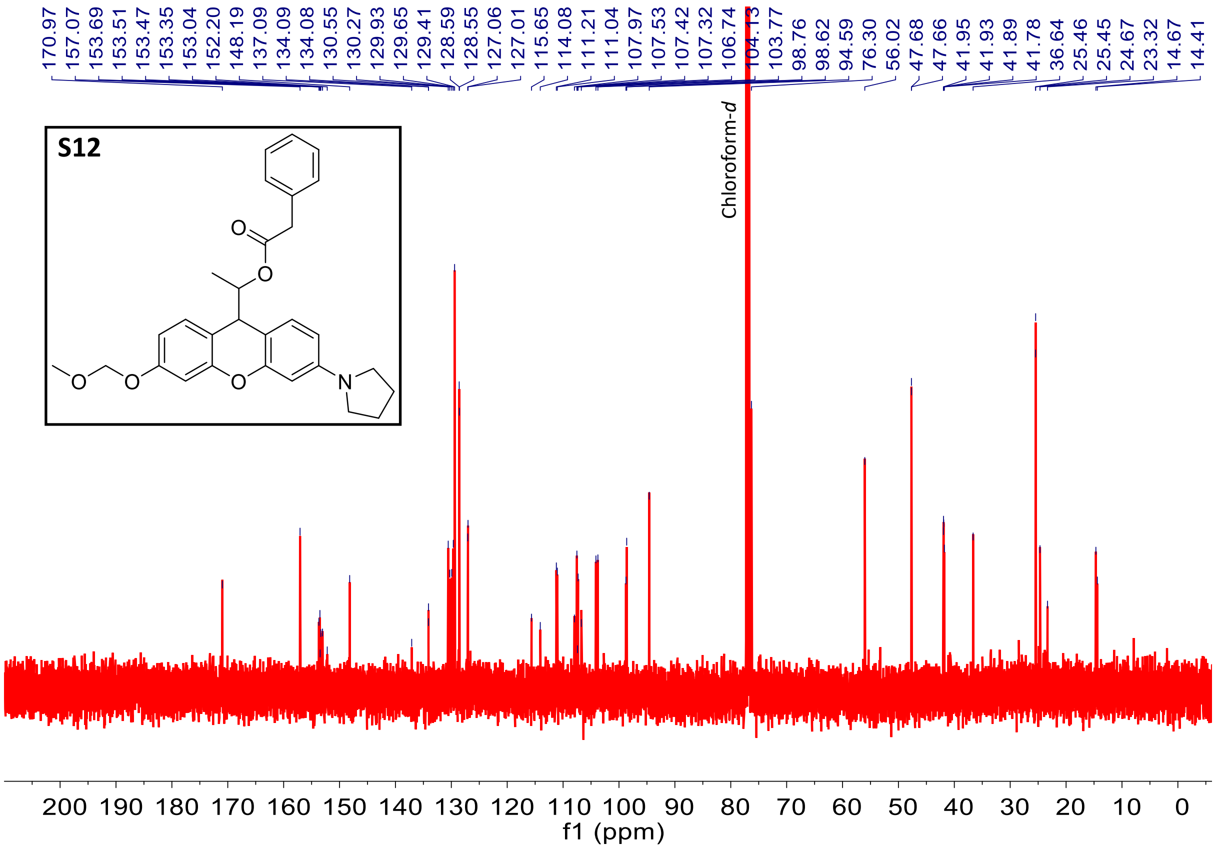

170.97 157.07 153.69 153.51 153.47 153.35 153.04 152.20 148.19 137.09 134.09 134.08 130.55 130.27 129.93 129.65 129.41 128.59 128.55 127.06 127.01 115.65 114.08 111.21 111.04 107.97 107.53 107.42 107.32 106.74 104.13 103.77 98.76 98.62 94.59 76.30 56.02 47.68 47.66 41.95 41.93 41.89 41.78 36.64 25.46 25.45 24.67 23.32 14.67 14.41
S12
O
O
O
O
O
N
Chloroform-d
200 190 180 170 160 150 140 130 120 110 100 90 80 70 60 50 40 30 20 10 0
f1 (ppm)

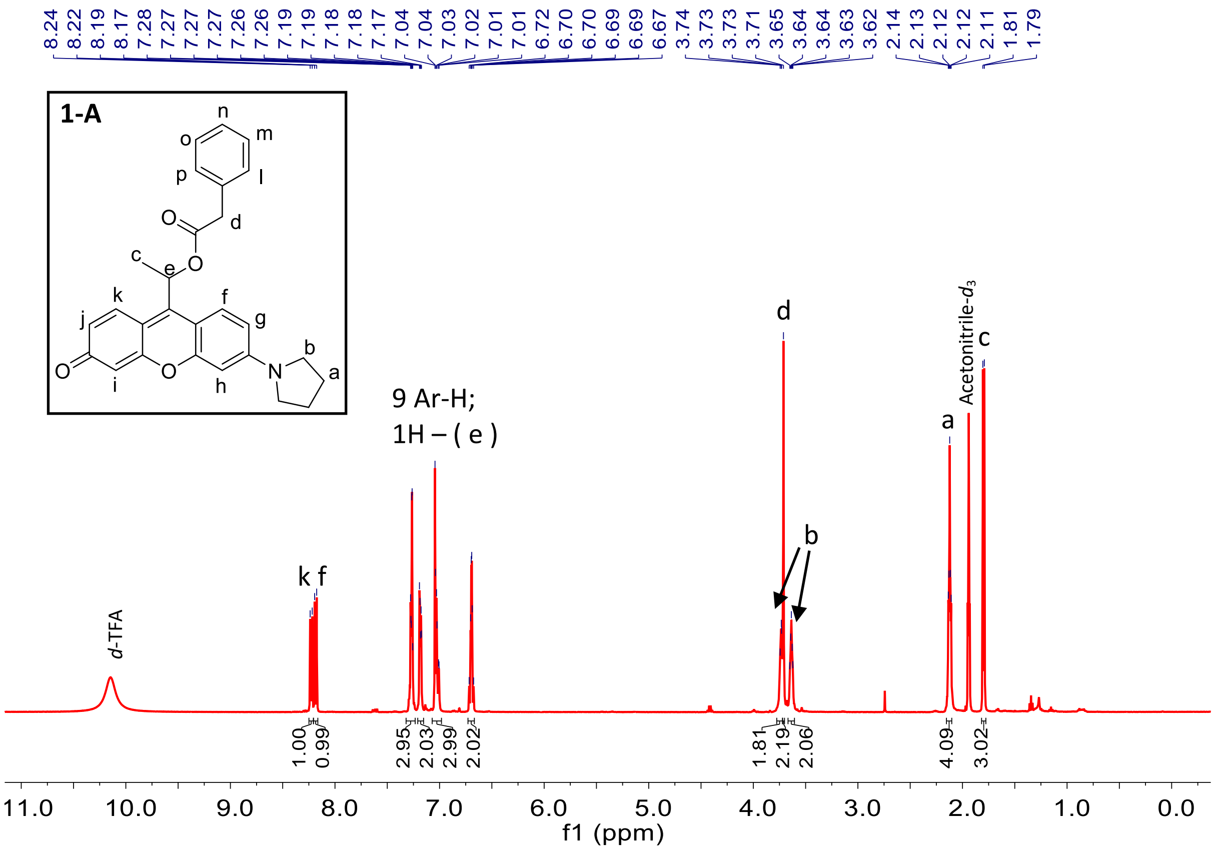
1-A
9 Ar-H;
1H – (e)
k f
d
b
a
c
Acetonitrile-d3
d-TFA
f1 (ppm)

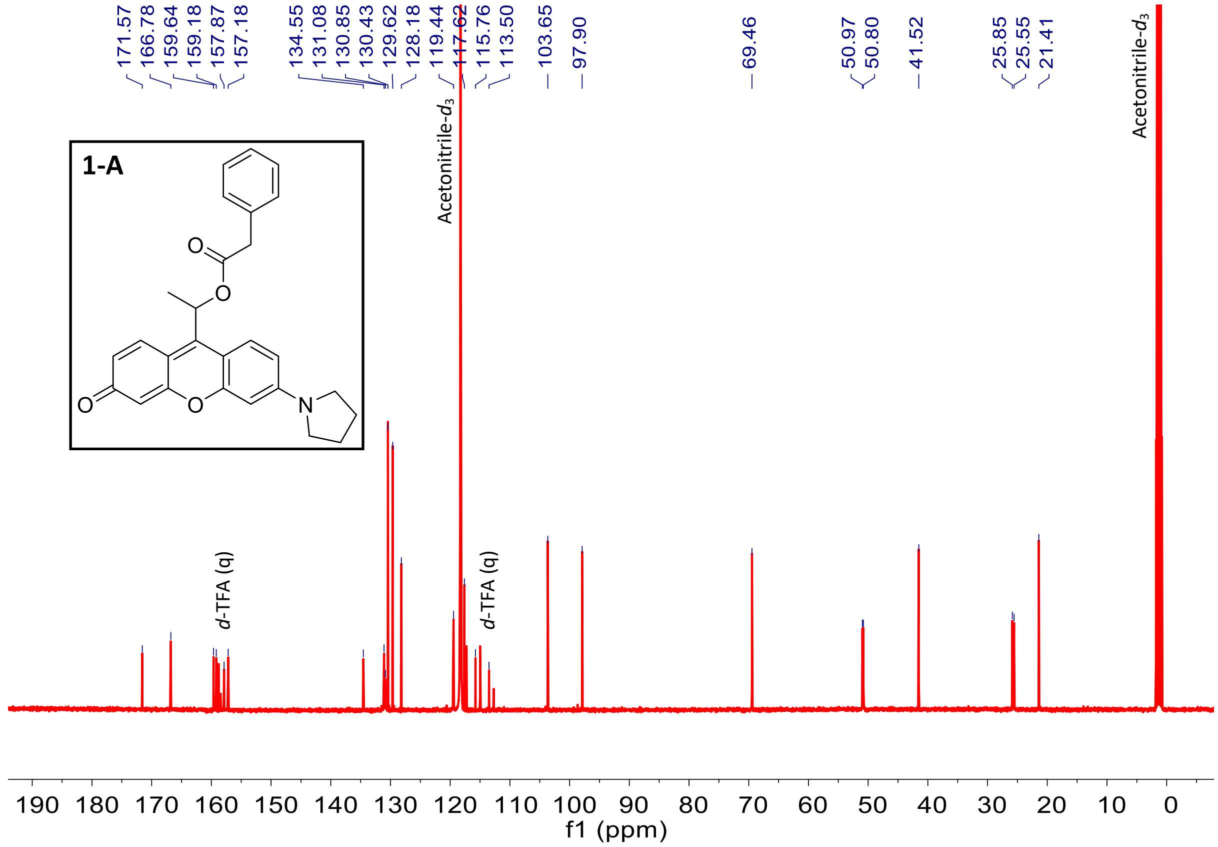
1-A
Acetonitrile-d3
d-TFA (q)
f1 (ppm)

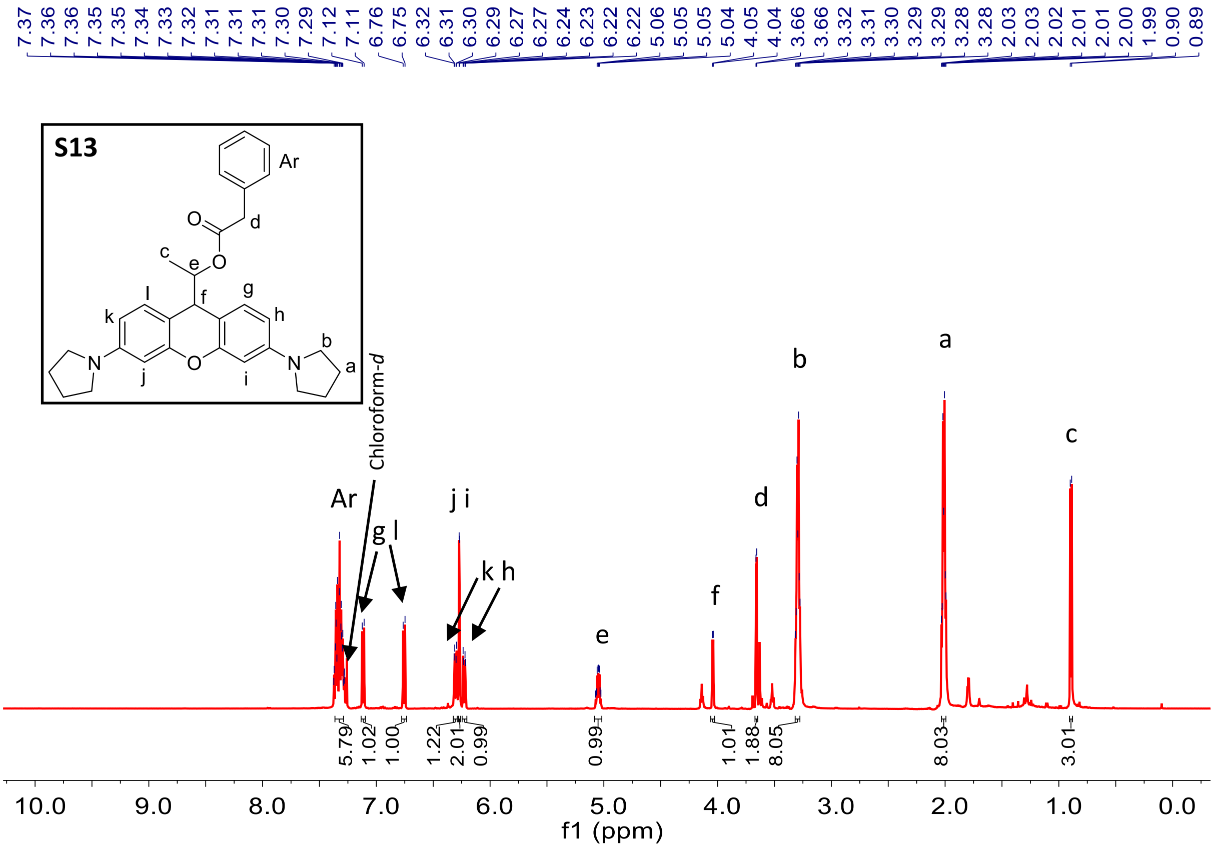
7.37 7.36 7.36 7.35 7.35 7.34 7.33 7.32 7.31 7.31 7.31 7.30 7.29 7.12 7.11 6.76 6.75 6.32 6.31 6.30 6.29 6.27 6.24 6.23 6.22 6.22 5.06 5.05 5.05 5.04 4.05 4.04 3.66 3.66 3.32 3.31 3.30 3.29 3.29 3.28 3.28 2.03 2.03 2.02 2.01 2.01 2.00 1.99 0.90 0.89
S13
Ar
Chloroform-d
g l
j i
k h
e
f
d
b
a
c
5.79 1.02 1.00 1.22 2.01 0.99 0.99 1.01 1.88 8.05 8.03 3.01
10.0 9.0 8.0 7.0 6.0 5.0 4.0 3.0 2.0 1.0 0.0
f1 (ppm)

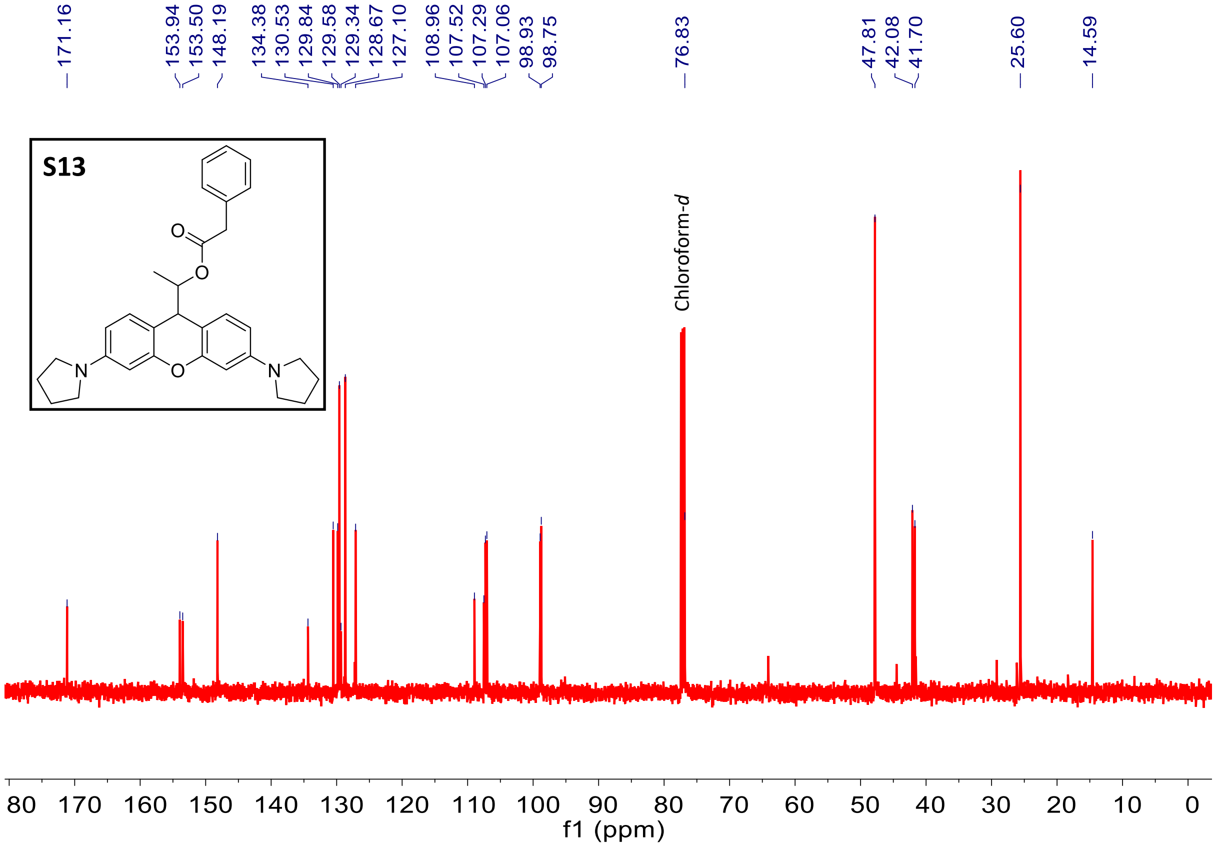
171.16 153.94 153.50 148.19 134.38 130.53 129.84 129.58 129.34 128.67 127.10 108.96 107.52 107.29 107.06 98.93 98.75 76.83 47.81 42.08 41.70 25.60 14.59
S13
Chloroform-d
180 170 160 150 140 130 120 110 100 90 80 70 60 50 40 30 20 10 0
f1 (ppm)

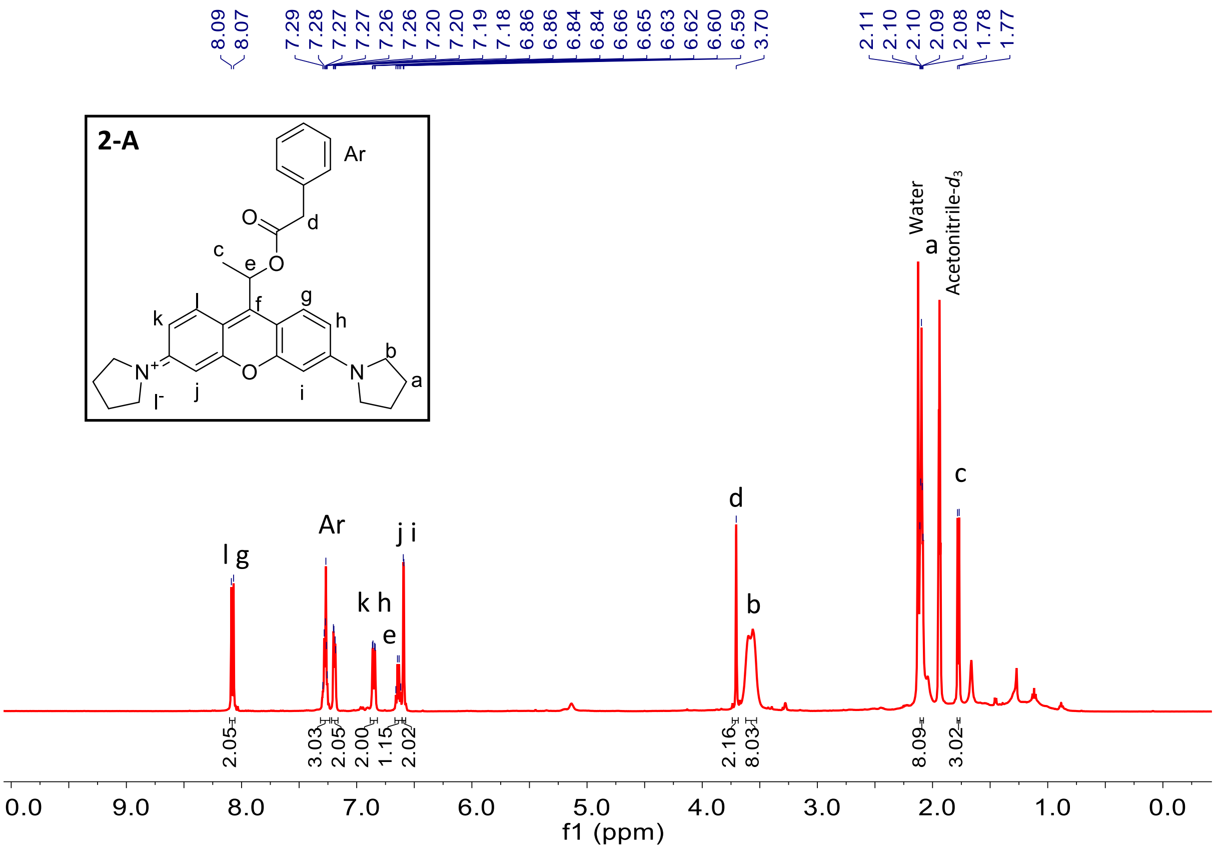
8.09
8.07
7.29
7.28
7.27
7.27
7.26
7.26
7.20
7.20
7.19
7.18
6.86
6.86
6.84
6.84
6.66
6.65
6.63
6.62
6.60
6.59
3.70
2.11
2.10
2.10
2.09
2.08
1.78
1.77
2-A
Ar
O
d
c
e
O
l
f
g
k
h
b
N+
O
N
a
I-
j
i
Water
a
Acetonitrile-d3
c
d
b
l g
Ar
k h
e
j i
2.05
3.03
2.05
2.00
1.15
2.02
2.16
8.03
8.09
3.02
0.0
9.0
8.0
7.0
6.0
5.0
4.0
3.0
2.0
1.0
0.0
f1 (ppm)

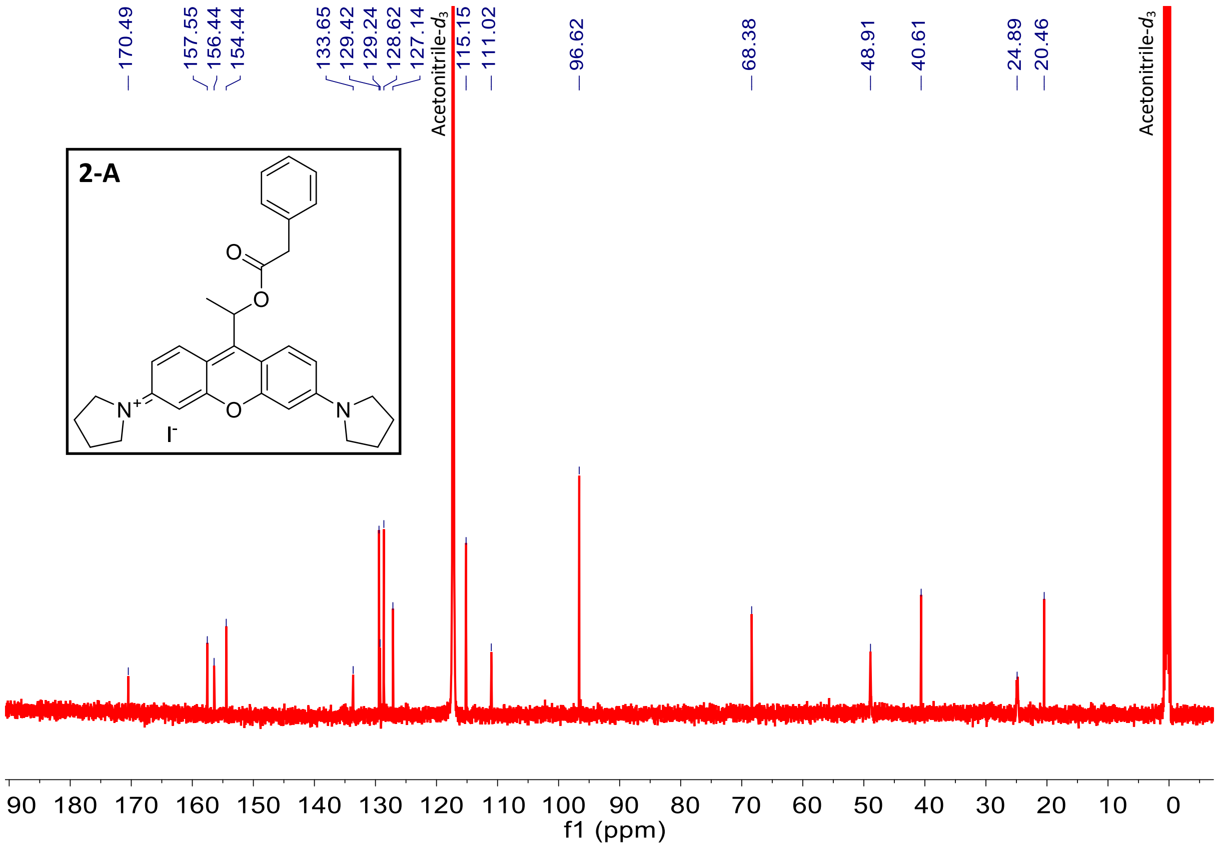
170.49
157.55
156.44
154.44
133.65
129.42
129.24
128.62
127.14
Acetonitrile-d3
115.15
111.02
96.62
68.38
48.91
40.61
24.89
20.46
Acetonitrile-d3
2-A
O
O
N+
O
N
I-
90
180
170
160
150
140
130
120
110
100
90
80
70
60
50
40
30
20
10
0
f1 (ppm)

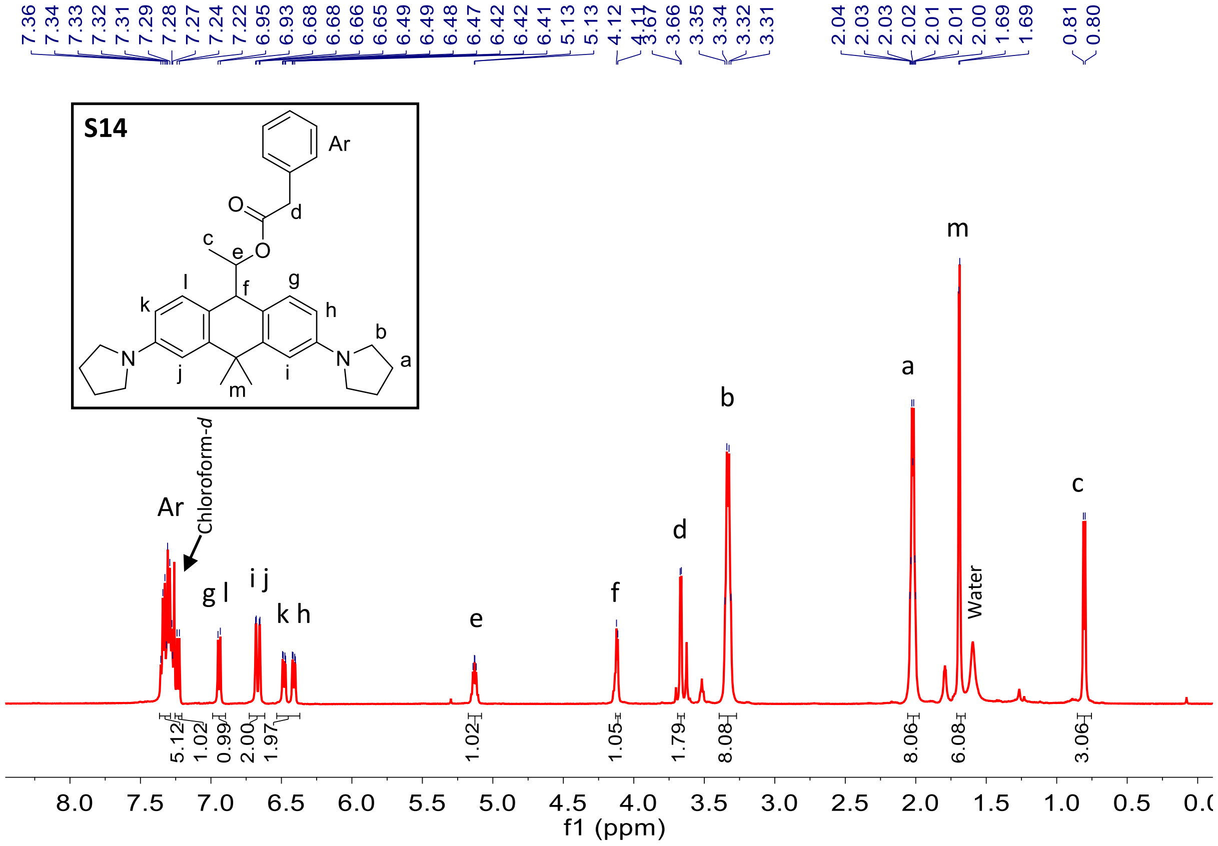
7.36 7.34 7.33 7.32 7.31 7.29 7.28 7.27 7.24 7.22 6.95 6.93 6.68 6.68 6.66 6.65 6.49 6.49 6.48 6.47 6.42 6.42 6.41 5.13 5.13 4.12 4.11 3.67 3.66 3.35 3.34 3.32 3.31 2.04 2.03 2.03 2.02 2.01 2.01 2.00 1.69 1.69 0.81 0.80
S14
Ar
Chloroform-d
Water
5.12 1.02 0.99 2.00 1.97 1.02 1.05 1.79 8.08 8.06 6.08 3.06
f1 (ppm)

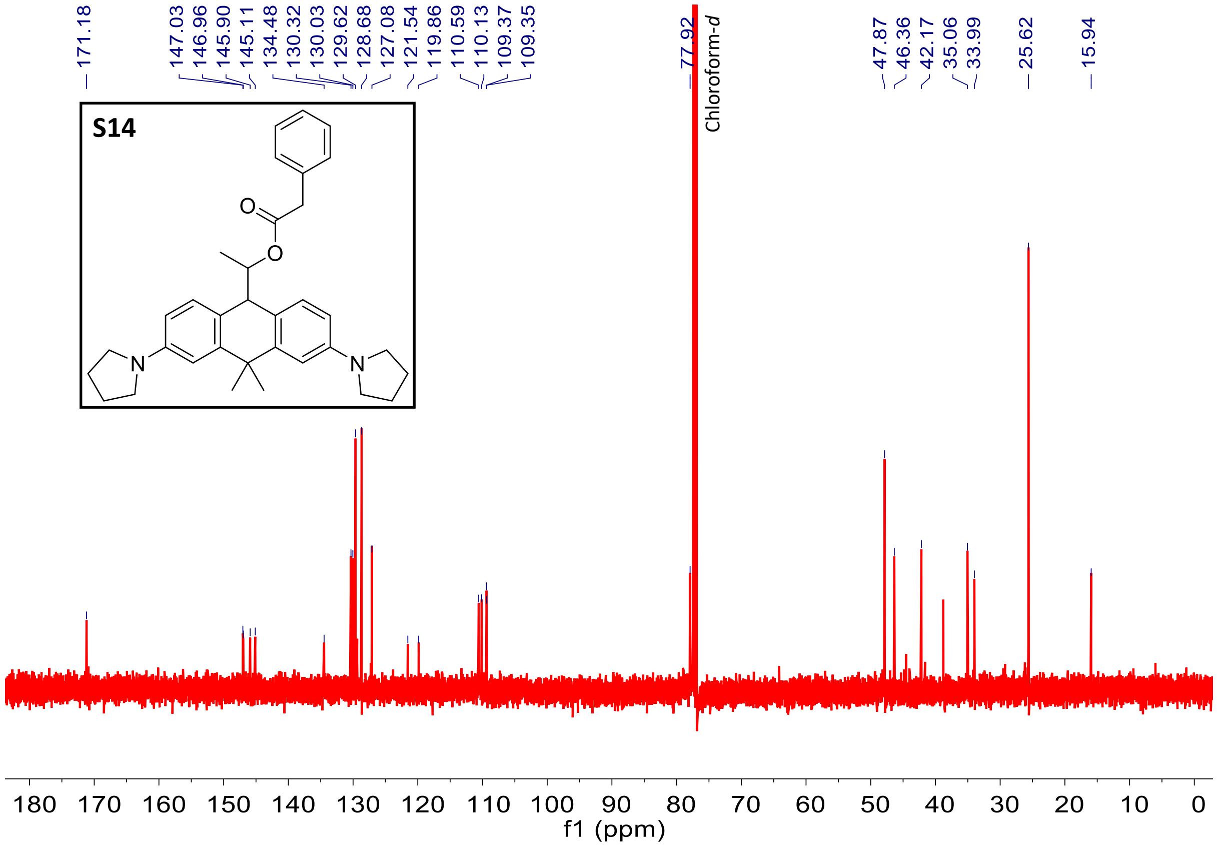
171.18
147.03 146.96 145.90 145.11 134.48 130.32 130.03 129.62 128.68 127.08 121.54 119.86 110.59 110.13 109.37 109.35
77.92
Chloroform-d
47.87 46.36 42.17 35.06 33.99 25.62 15.94
S14
f1 (ppm)

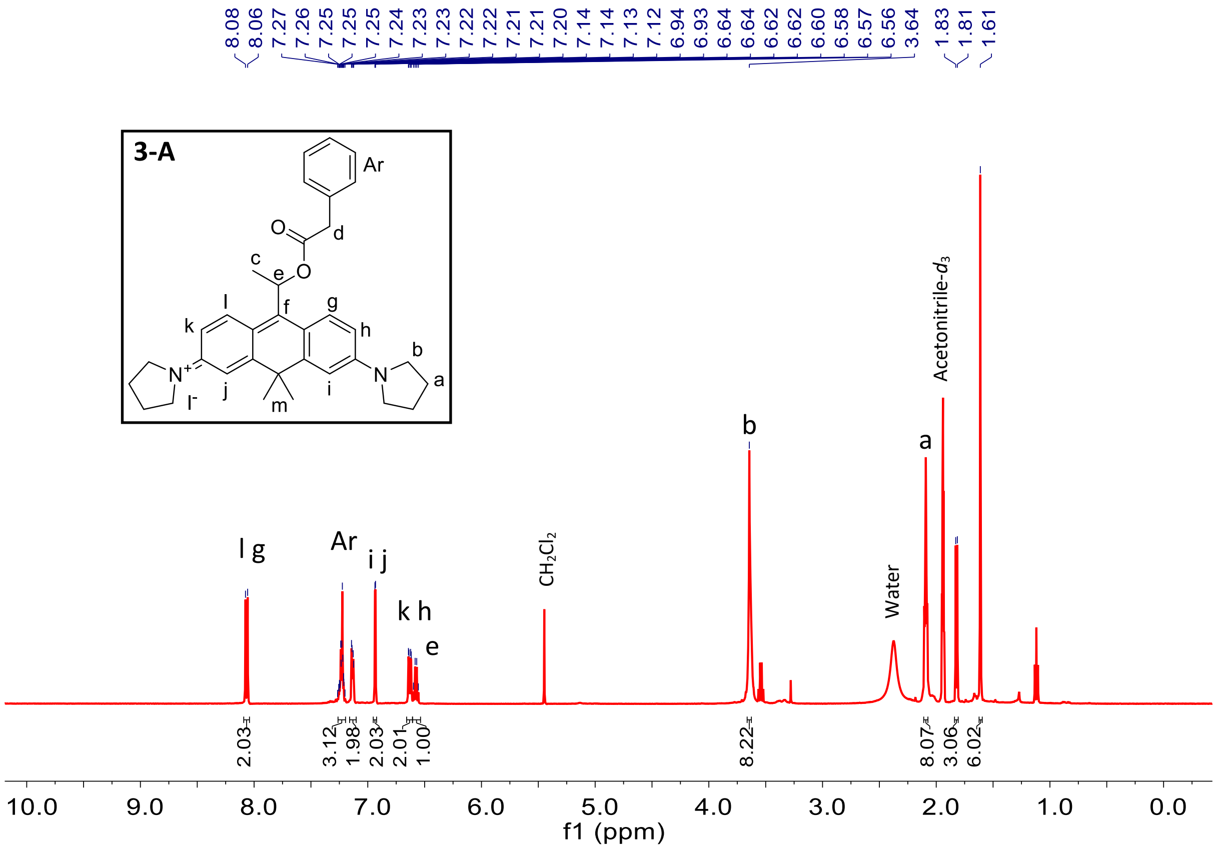
8.08
8.06
7.27
7.26
7.25
7.25
7.25
7.24
7.23
7.23
7.22
7.22
7.21
7.21
7.20
7.14
7.13
7.12
6.94
6.93
6.64
6.64
6.62
6.62
6.60
6.58
6.57
6.56
3.64
1.83
1.81
1.61
3-A
Ar
O
d
c
e
O
l
f
g
k
h
b
N+
j
i
N
a
I-
m
l g
Ar
i j
k h
e
CH2Cl2
b
Water
a
Acetonitrile-d3
2.03
3.12
1.98
2.03
2.01
1.00
8.22
8.07
3.06
6.02
10.0
9.0
8.0
7.0
6.0
5.0
4.0
3.0
2.0
1.0
0.0
f1 (ppm)

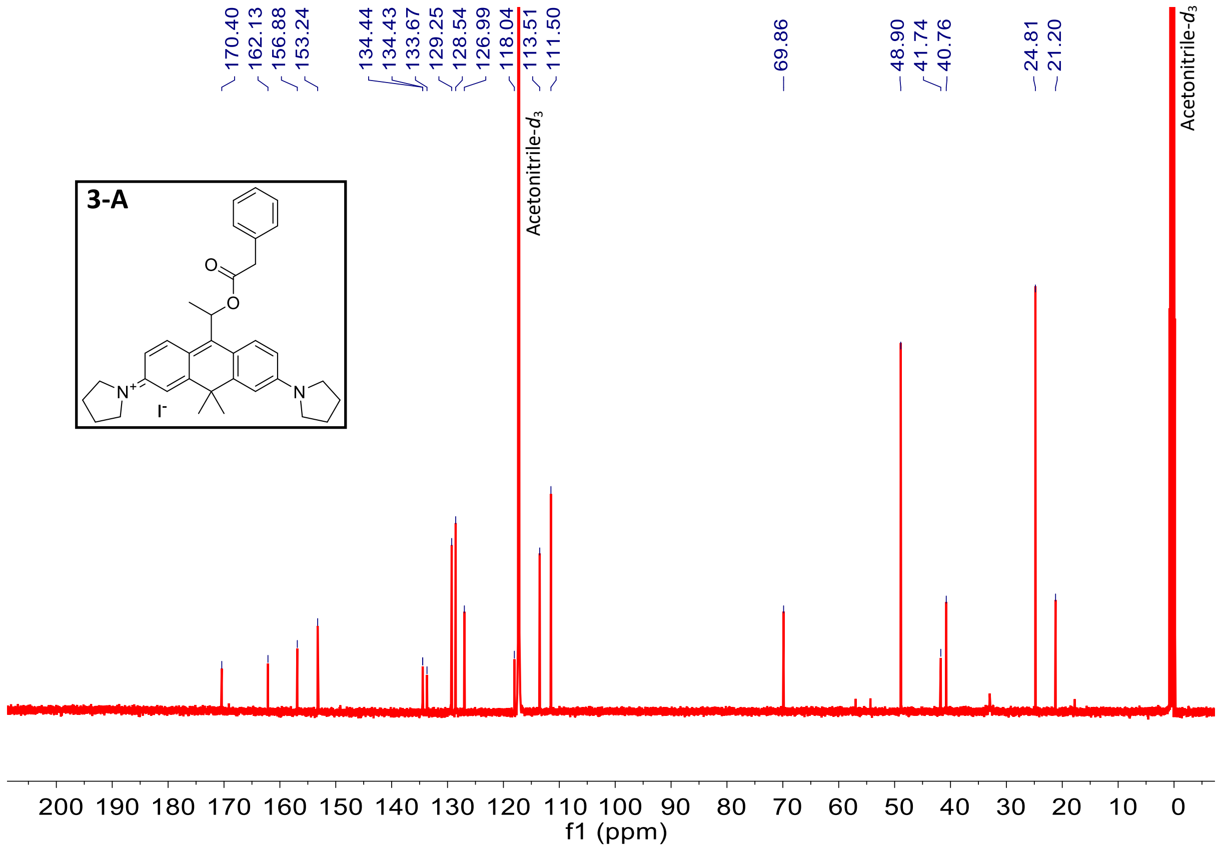
170.40
162.13
156.88
153.24
134.44
134.43
133.67
129.25
128.54
126.99
118.04
113.51
111.50
69.86
48.90
41.74
40.76
24.81
21.20
Acetonitrile-d3
Acetonitrile-d3
3-A
O
O
N+
I-
N
200
190
180
170
160
150
140
130
120
110
100
90
80
70
60
50
40
30
20
10
0
f1 (ppm)

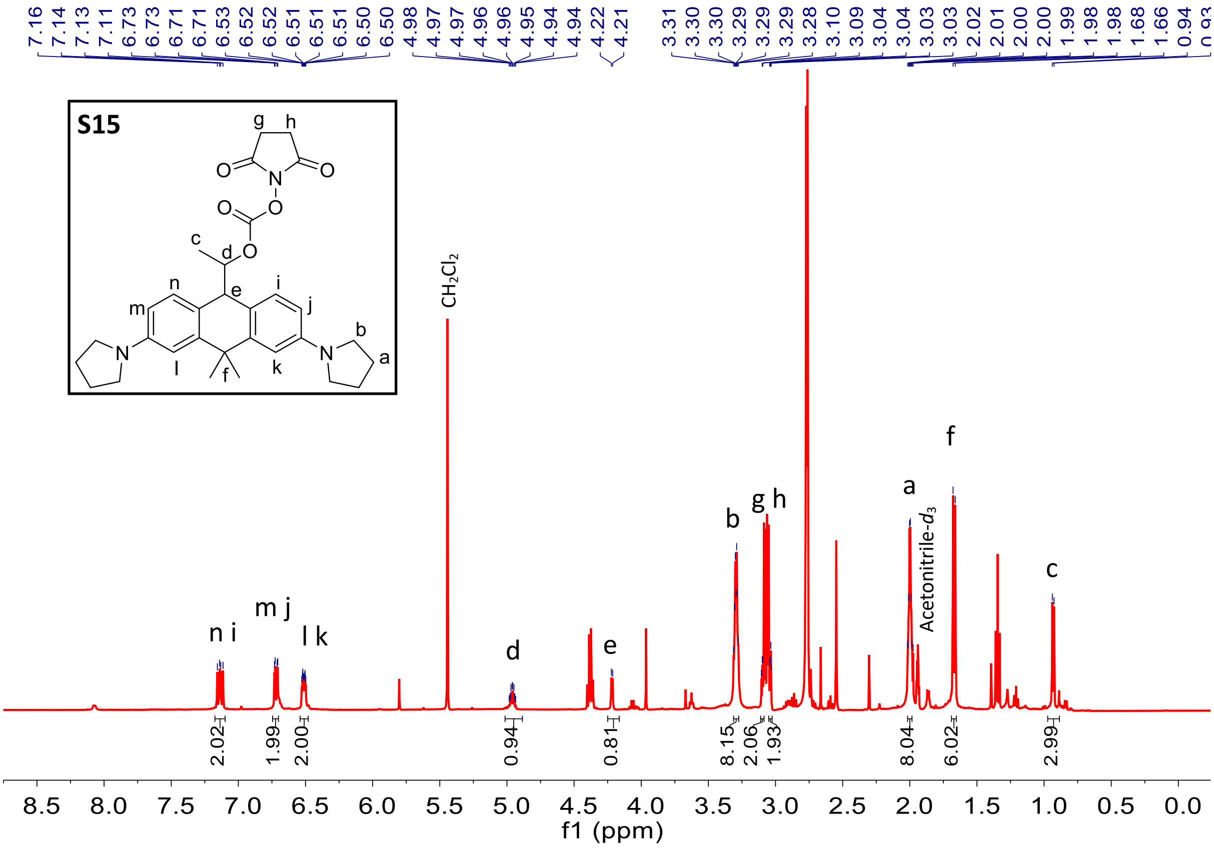
S15
CH2Cl2
Acetonitrile-d3
n i
m j
l k
d
e
b
g h
a
f
c
f1 (ppm)

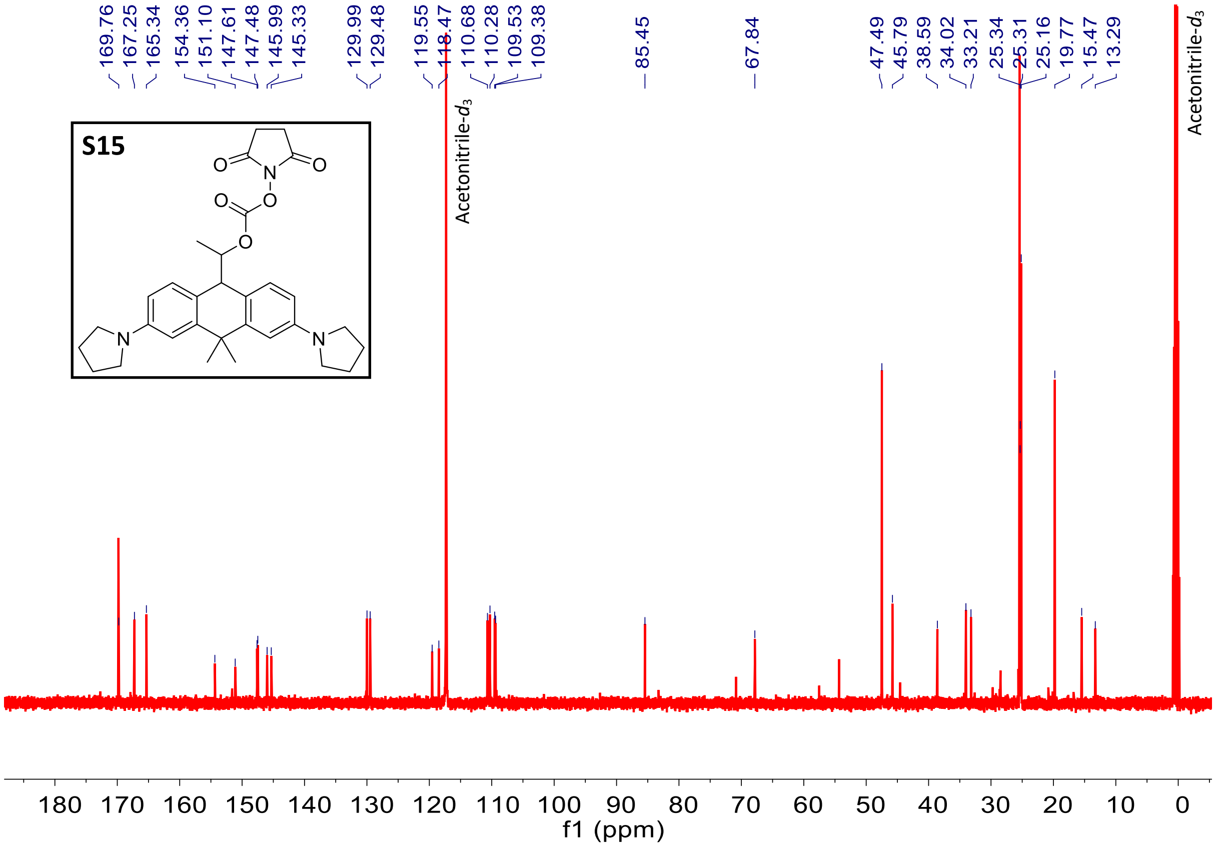
S15
Acetonitrile-d3
Acetonitrile-d3
f1 (ppm)

S15

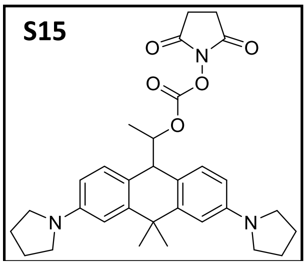

Date Acquired	: 2021.09.22. 8:08:07	Report Format File	: Alap_report.lsr
Sample Name	: EA-5-671-on	Background Data File	: bg_210922.lcd
Tray#	: 1	Batch File	: batch_210922.lcb
Vial#	: 1	Option 1	: EA
Injection Volume	: 1	Option 2	: 103
Data File	: EA-5-671-on.lcd		
Method File	: Alapmodszer_ESI_4min_1ml.lcm		

Chromatogram

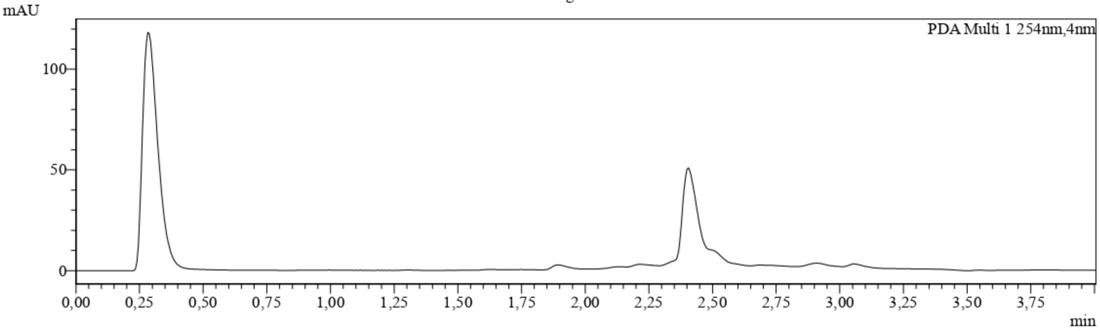

MS Chromatogram

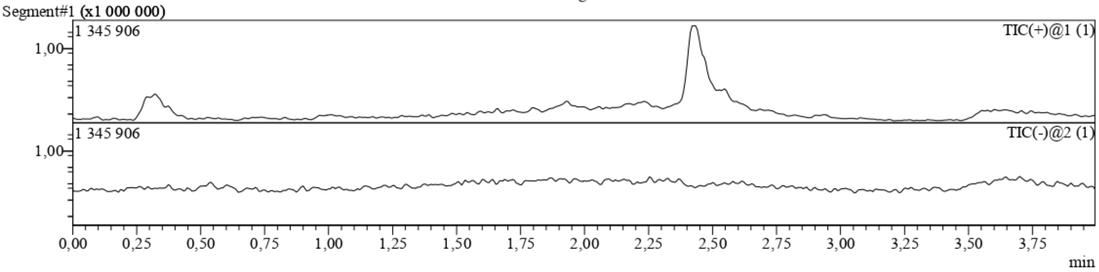

Peak Table

PDA Ch1 254nm

Peak#	Ret. Time	Area	Area%	Height	Height%	Name
Total						

MS Spectrum

Line#:1 R.Time:2,413(Scan#:725)
MassPeaks:1075
Spectrum Mode:Single 2,413(725) Base Peak:287(432548)
BG Mode:None Segment 1 - Event 1

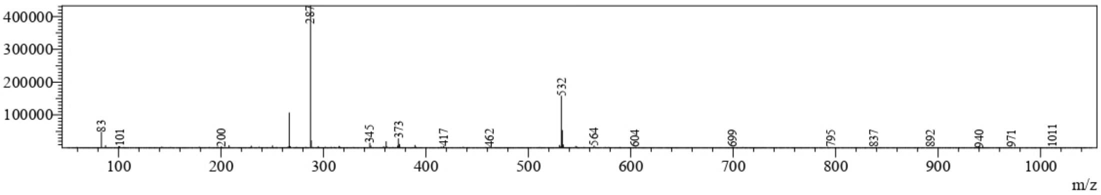

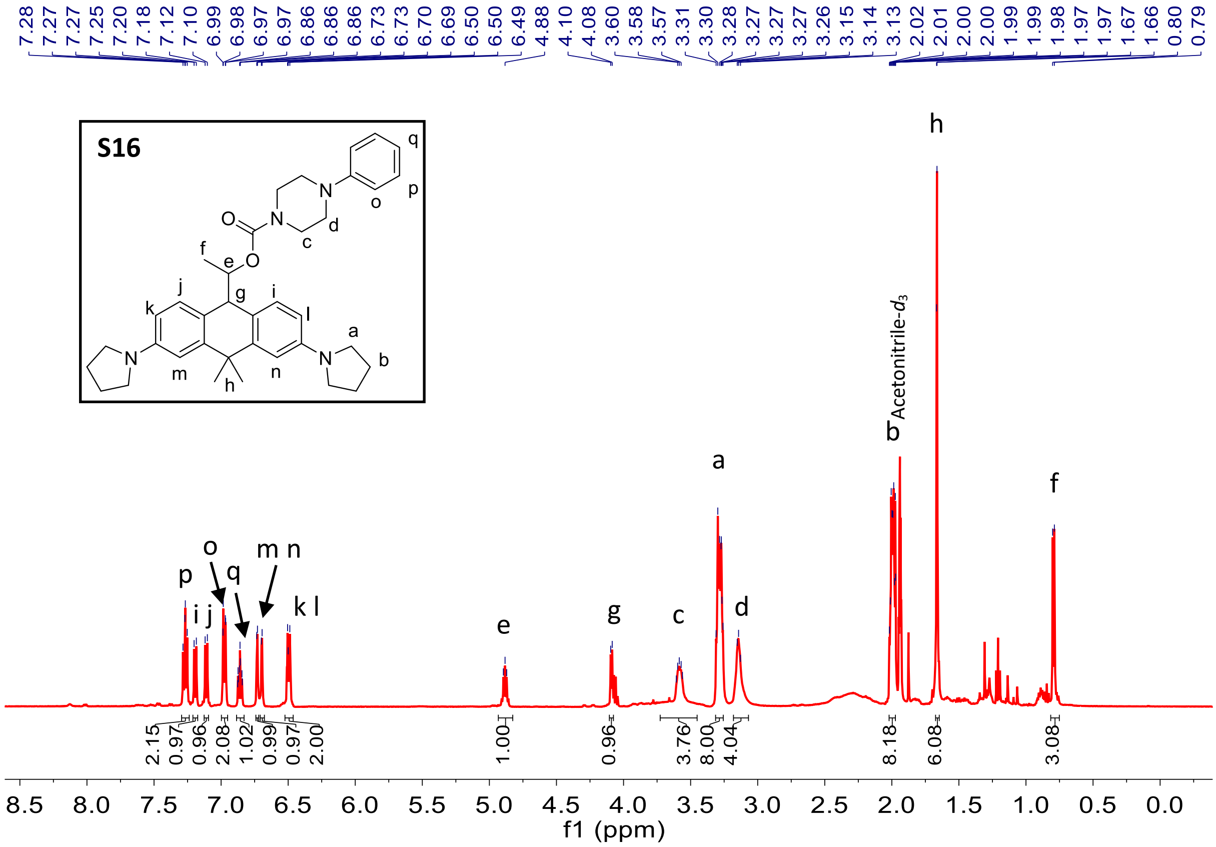
7.28 7.27 7.27 7.25 7.20 7.18 7.12 7.10 6.99 6.98 6.97 6.97 6.86 6.86 6.86 6.73 6.73 6.70 6.69 6.50 6.50 6.49 4.88 4.10 4.08 3.60 3.58 3.57 3.31 3.30 3.28 3.27 3.27 3.27 3.26 3.15 3.14 3.13 2.02 2.01 2.00 2.00 1.99 1.99 1.98 1.97 1.97 1.67 1.66 0.80 0.79
S16
Acetonitrile-d_3
2.15 0.97 0.96 2.08 1.02 0.99 0.97 2.00 1.00 0.96 3.76 8.00 4.04 8.18 6.08 3.08
f1 (ppm)

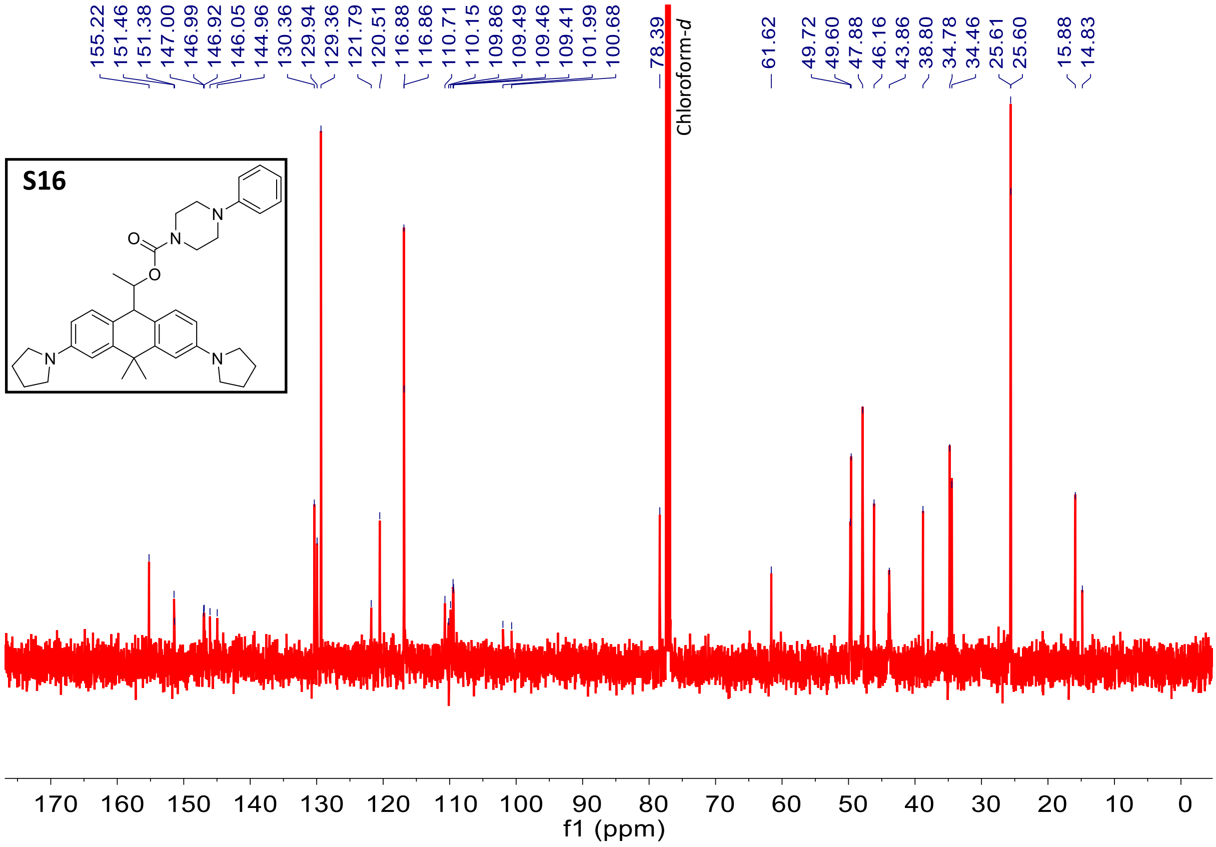
155.22 151.46 151.38 147.00 146.99 146.92 146.05 144.96 130.36 129.94 129.36 121.79 120.51 116.88 116.86 110.71 110.15 109.86 109.49 109.46 109.41 101.99 100.68 78.39 61.62 49.72 49.60 47.88 46.16 43.86 38.80 34.78 34.46 25.61 25.60 15.88 14.83
Chloroform-d
S16
f1 (ppm)

S16

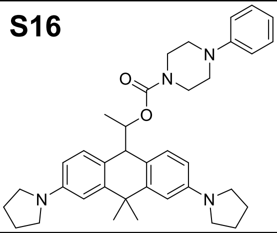

Date Acquired	: 2022.09.05. 11:59:08	Report Format File	: Alap_report.lsr
Sample Name	: EA-6-817-oszlop	Background Data File	: bg_220905.lcd
Tray#	: 1	Batch File	: batch_220905.lcb
Vial#	: 23	Option 1	: EA
Injection Volume	: 3	Option 2	: 103
Data File	: EA-6-817-oszlop.lcd		
Method File	: LowPressure_ESI_5min_0p8ml.lcm		

Chromatogram

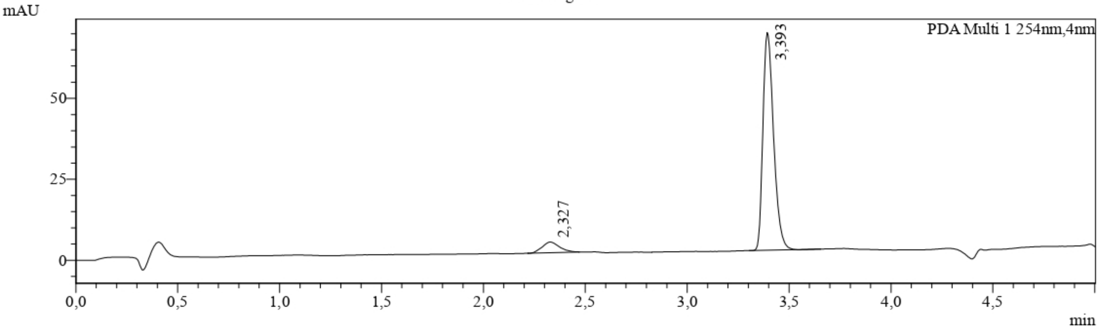

MS Chromatogram

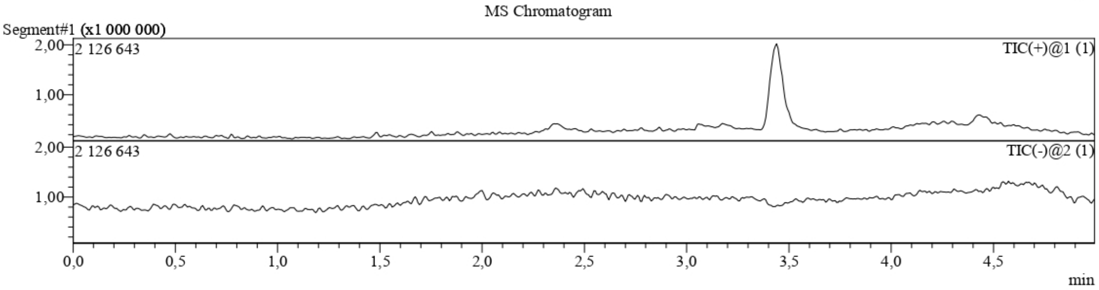

Peak Table

PDA Ch1 254nm

Peak#	Ret. Time	Area	Area%	Height	Height%	Name
1	2,327	19414	7,358	3324	4,708	
2	3,393	244429	92,642	67289	95,292	
Total		263843	100,000	70613	100,000	

MS Spectrum

Line#:1 R.Time:3,433(Scan#:1031)
MassPeaks:1058
Spectrum Mode:Single 3,433(1031) Base Peak:311(318602)
BG Mode:None Segment 1 - Event 1

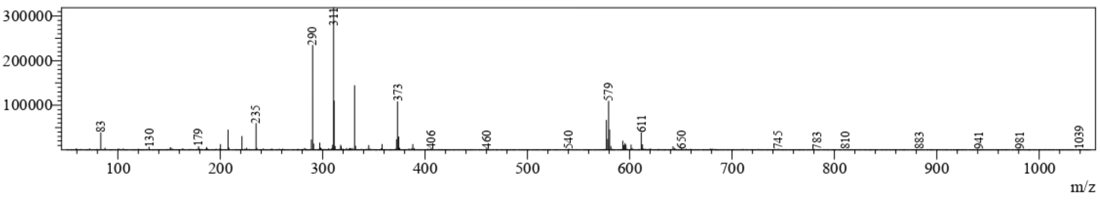

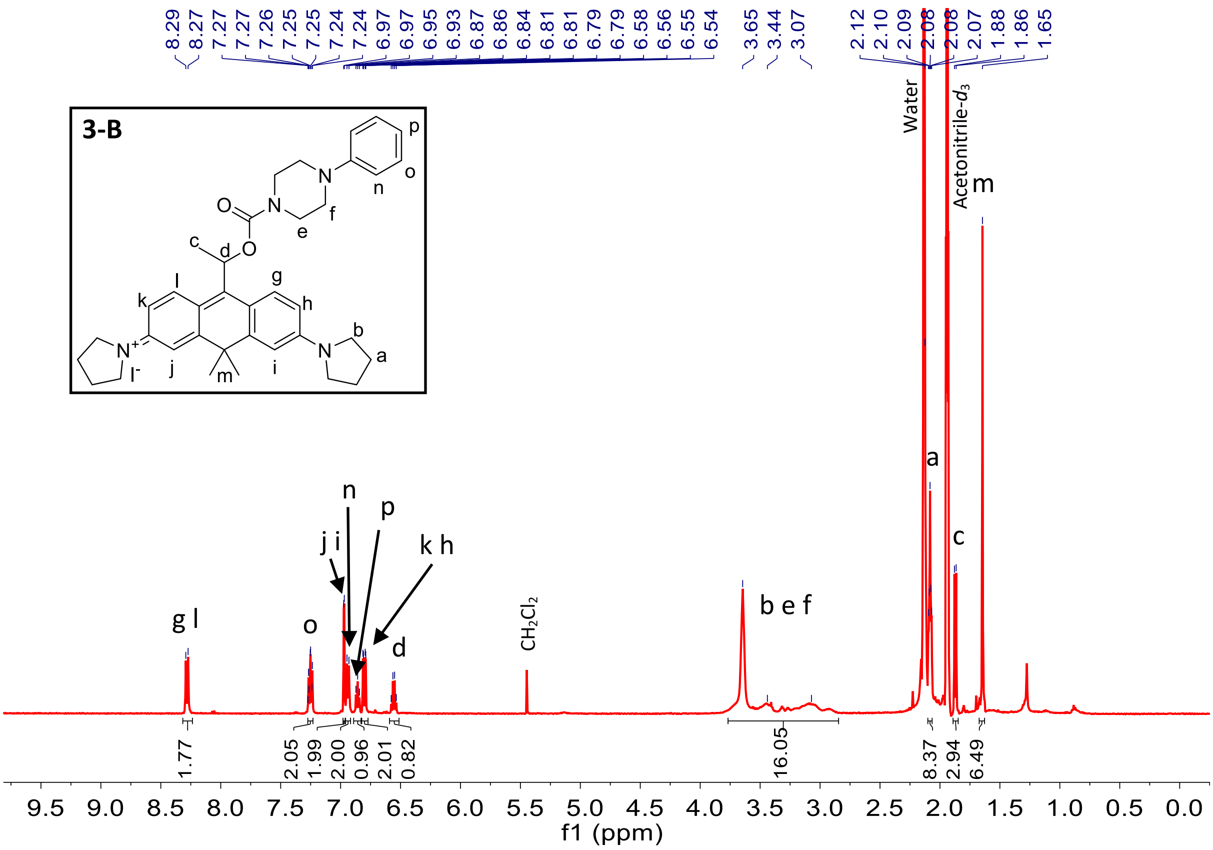

8.29
8.27
7.27
7.27
7.26
7.25
7.25
7.24
7.24
6.97
6.97
6.95
6.93
6.87
6.86
6.84
6.81
6.81
6.79
6.79
6.58
6.56
6.55
6.54
3.65
3.44
3.07
2.12
2.10
2.09
2.08
2.08
2.07
1.88
1.86
1.65
3-B
Water
Acetonitrile-d3
m
a
c
n
p
j i
k h
g l
o
d
CH2Cl2
b e f
1.77
2.05
1.99
2.00
0.96
2.01
0.82
16.05
8.37
2.94
6.49
9.5 9.0 8.5 8.0 7.5 7.0 6.5 6.0 5.5 5.0 4.5 4.0 3.5 3.0 2.5 2.0 1.5 1.0 0.5 0.0
f1 (ppm)

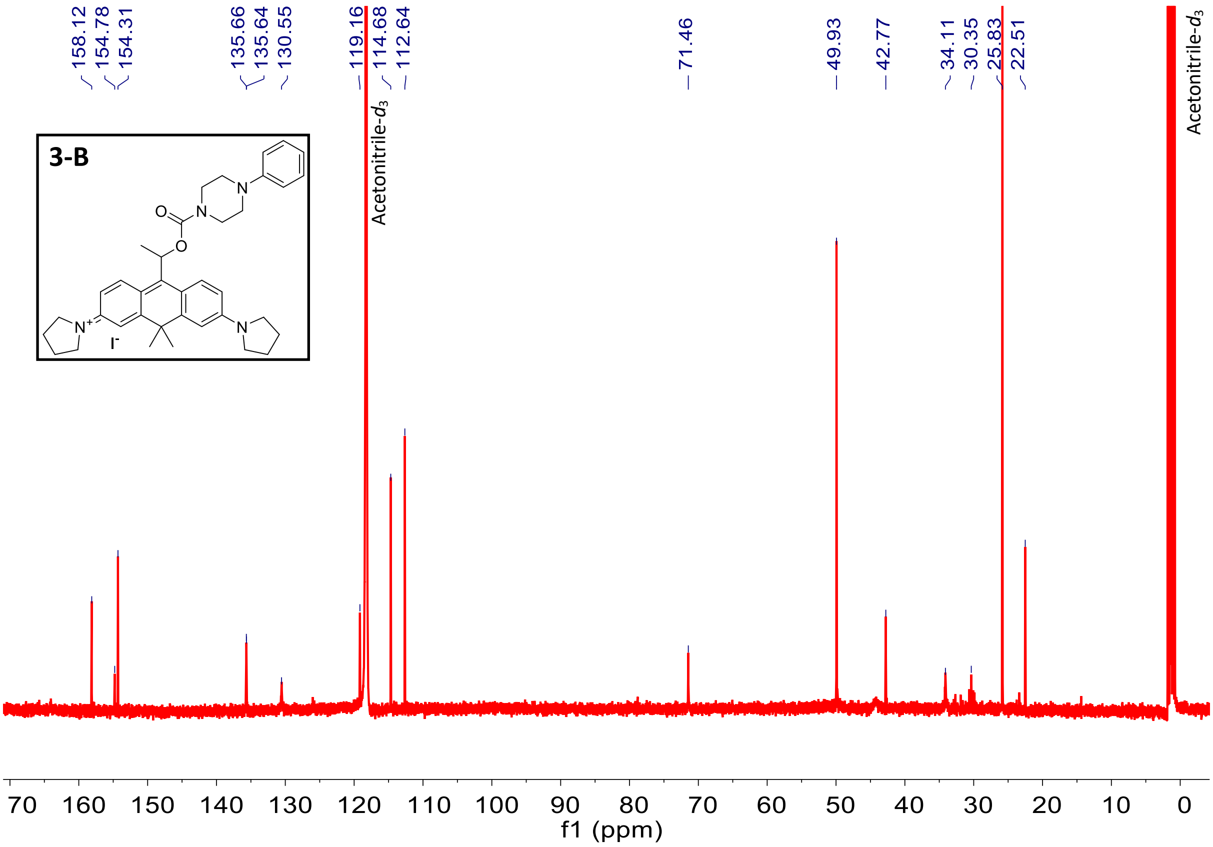

158.12
154.78
154.31
135.66
135.64
130.55
119.16
114.68
112.64
71.46
49.93
42.77
34.11
30.35
25.83
22.51
3-B
Acetonitrile-d3
Acetonitrile-d3
170 160 150 140 130 120 110 100 90 80 70 60 50 40 30 20 10 0
f1 (ppm)

3-B

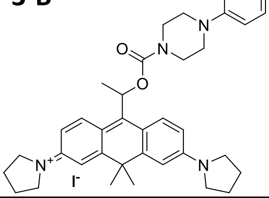

Date Acquired	: 2021.10.27. 14:42:26	Report Format File	: Alap_report.lsr
Sample Name	: EA-5-689-checkTFA	Background Data File	: bg_211027.lcd
Tray#	: 1	Batch File	: batch_211027.lcb
Vial#	: 43	Option 1	: EA
Injection Volume	: 1	Option 2	: 103
Data File	: EA-5-689-checkTFA.lcd		
Method File	: Alapmodszer_ESI_4min_1ml.lcm		

Chromatogram

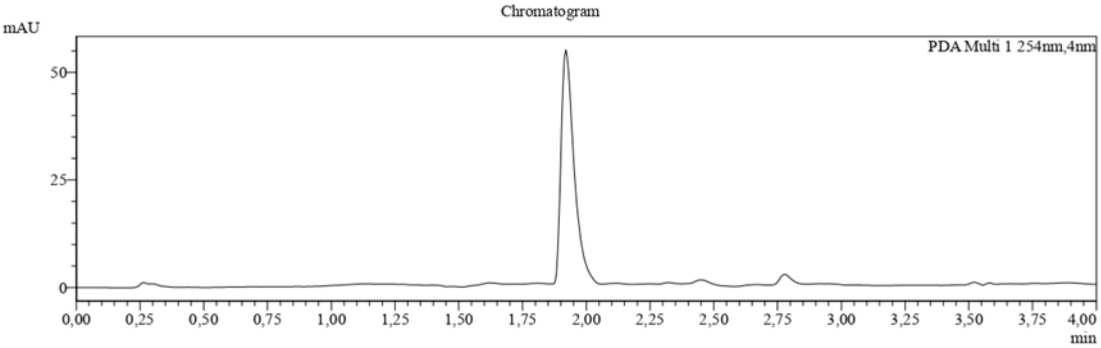

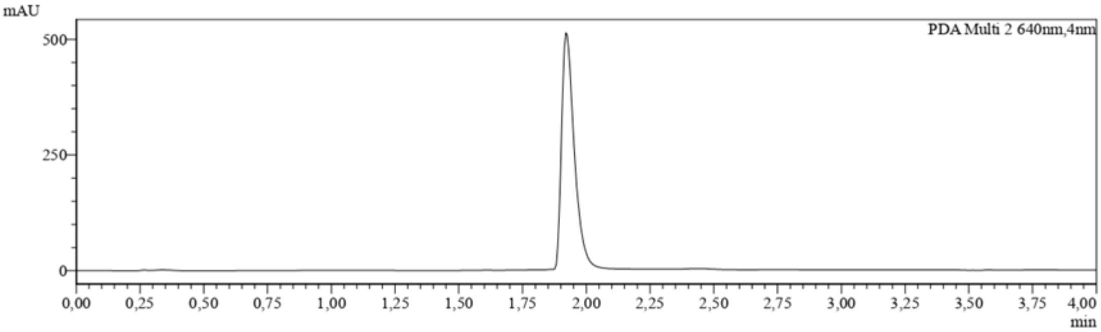

MS Chromatogram

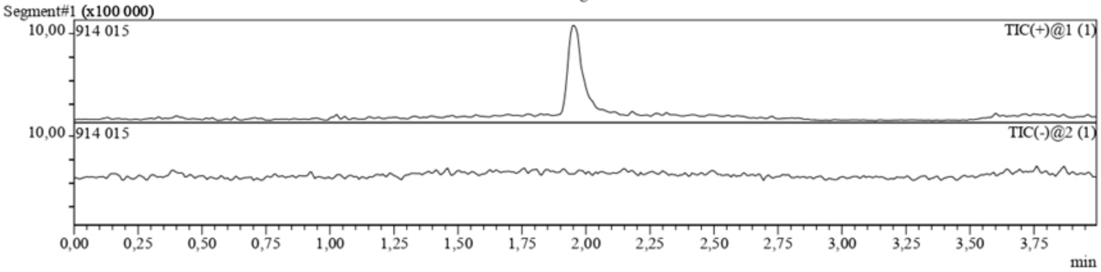

Peak Table

PDA Ch1 254nm

Peak#	Ret. Time	Area	Area%	Height	Height%	Name
Total						

PDA Ch2 640nm

Peak#	Ret. Time	Area	Area%	Height	Height%	Name
Total						

MS Spectrum

Line#:1 R.Time:1,940(Scan#:583)
MassPeaks:1060
Spectrum Mode:Single 1,940(583) Base Peak:577(332203)
BG Mode:None Segment 1 - Event 1

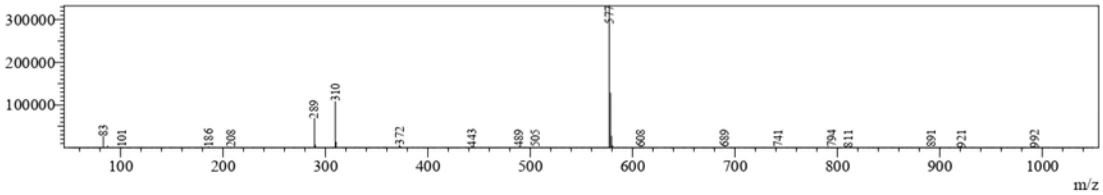

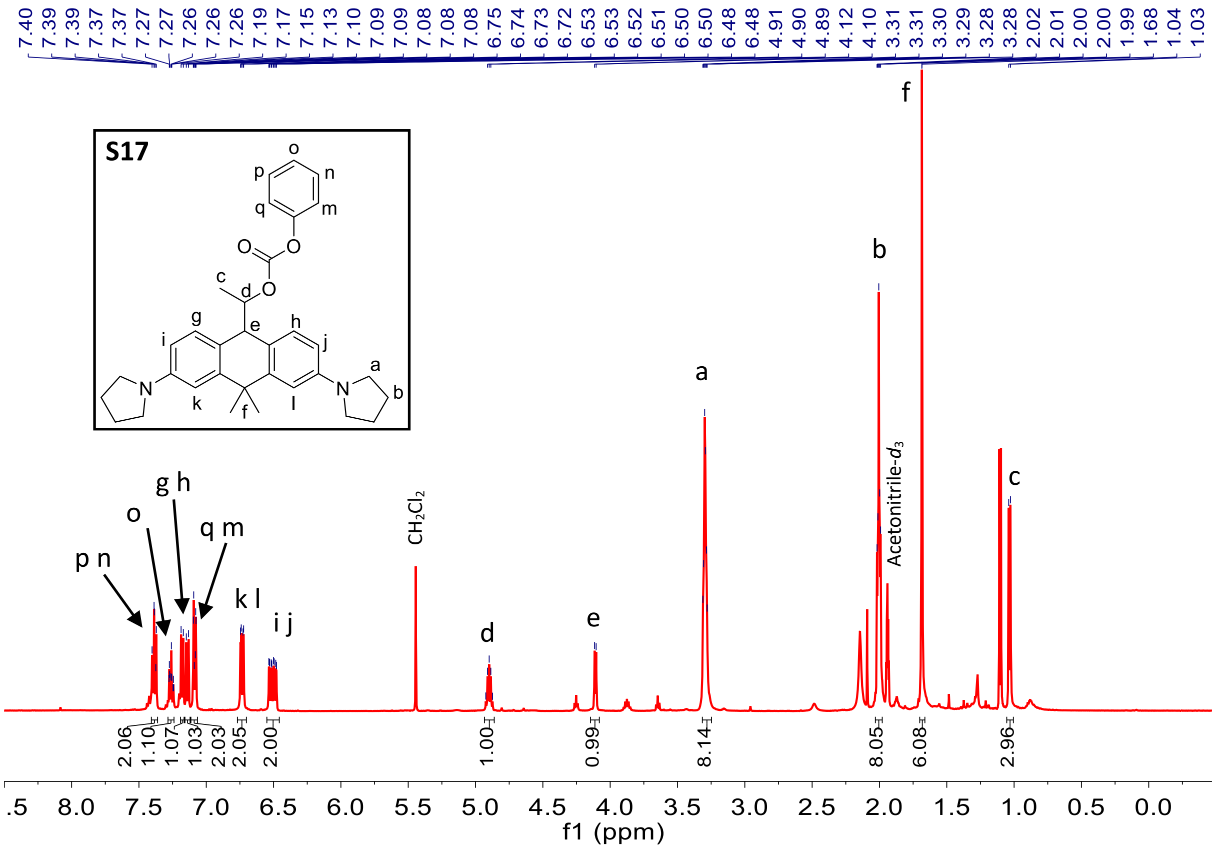
S17
p n
o
g h
q m
k l
i j
CH$_2$Cl$_2$
d
e
a
b
Acetonitrile-d_3
f
c
2.06
1.10
1.07
1.03
2.03
2.05
2.00
1.00
0.99
8.14
8.05
6.08
2.96
f1 (ppm)

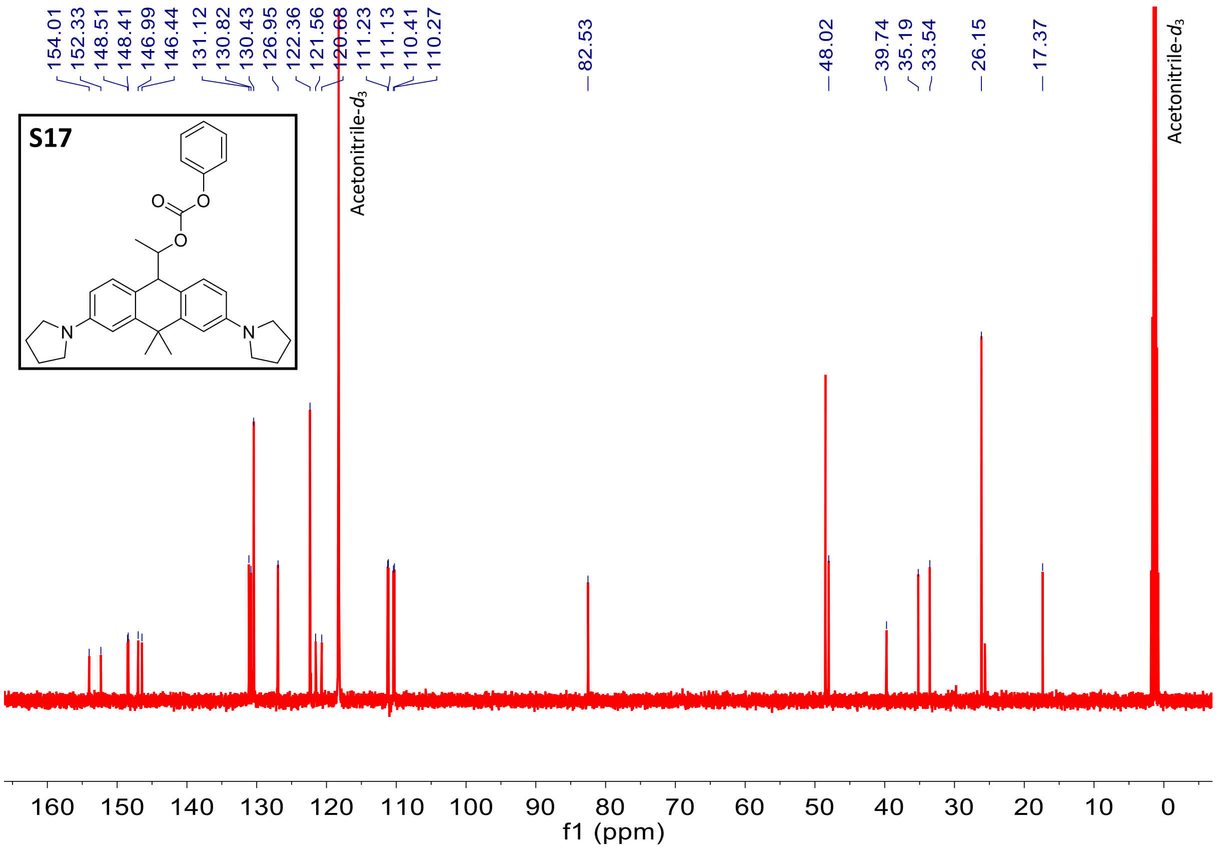
S17
154.01
152.33
148.51
148.41
146.99
146.44
131.12
130.82
130.43
126.95
122.36
121.56
120.68
111.23
111.13
110.41
110.27
82.53
48.02
39.74
35.19
33.54
26.15
17.37
Acetonitrile-d_3
Acetonitrile-d_3
f1 (ppm)

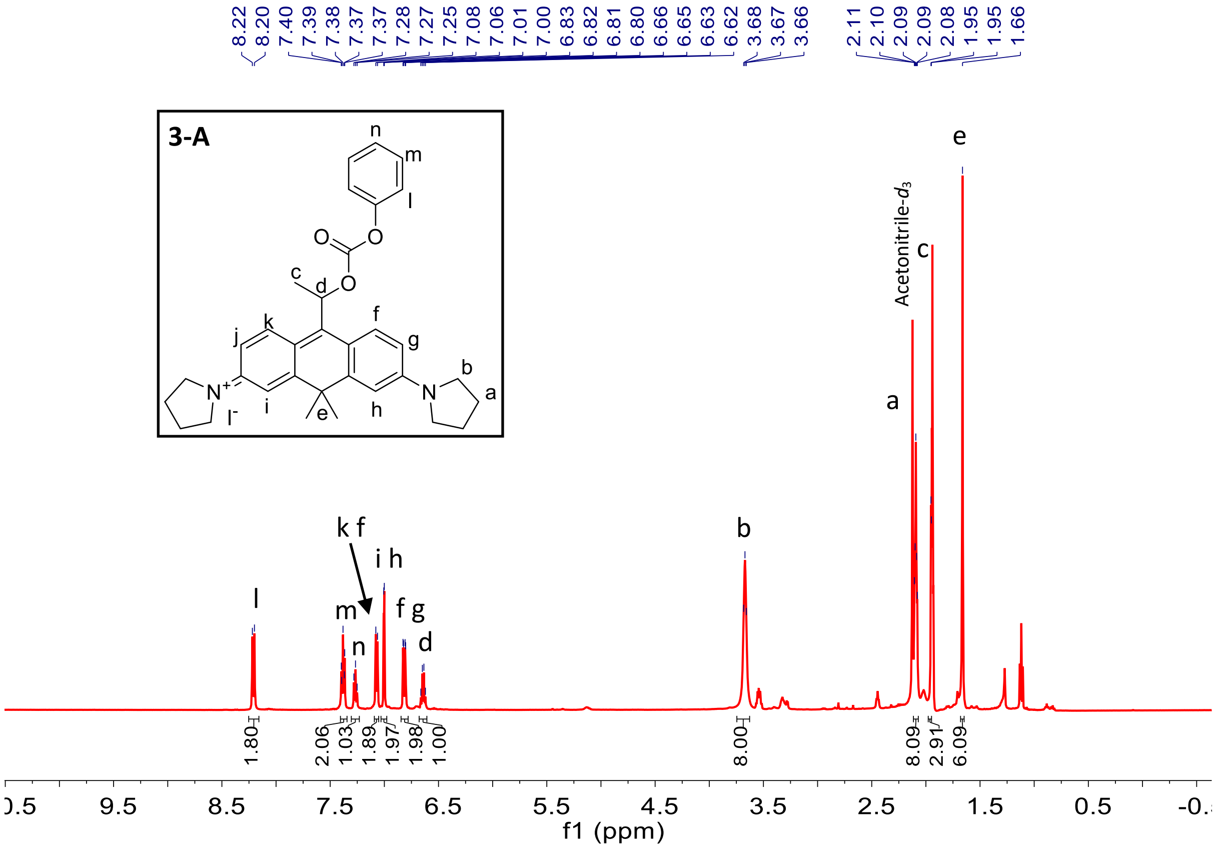
8.22
8.20
7.40
7.39
7.38
7.37
7.37
7.28
7.27
7.25
7.08
7.06
7.01
7.00
6.83
6.82
6.81
6.80
6.66
6.65
6.63
6.62
3.68
3.67
3.66
2.11
2.10
2.09
2.09
2.08
1.95
1.95
1.66
3-A
Acetonitrile-d_3
1.80
2.06
1.03
1.89
1.97
1.98
1.00
8.00
8.09
2.91
6.09
f1 (ppm)

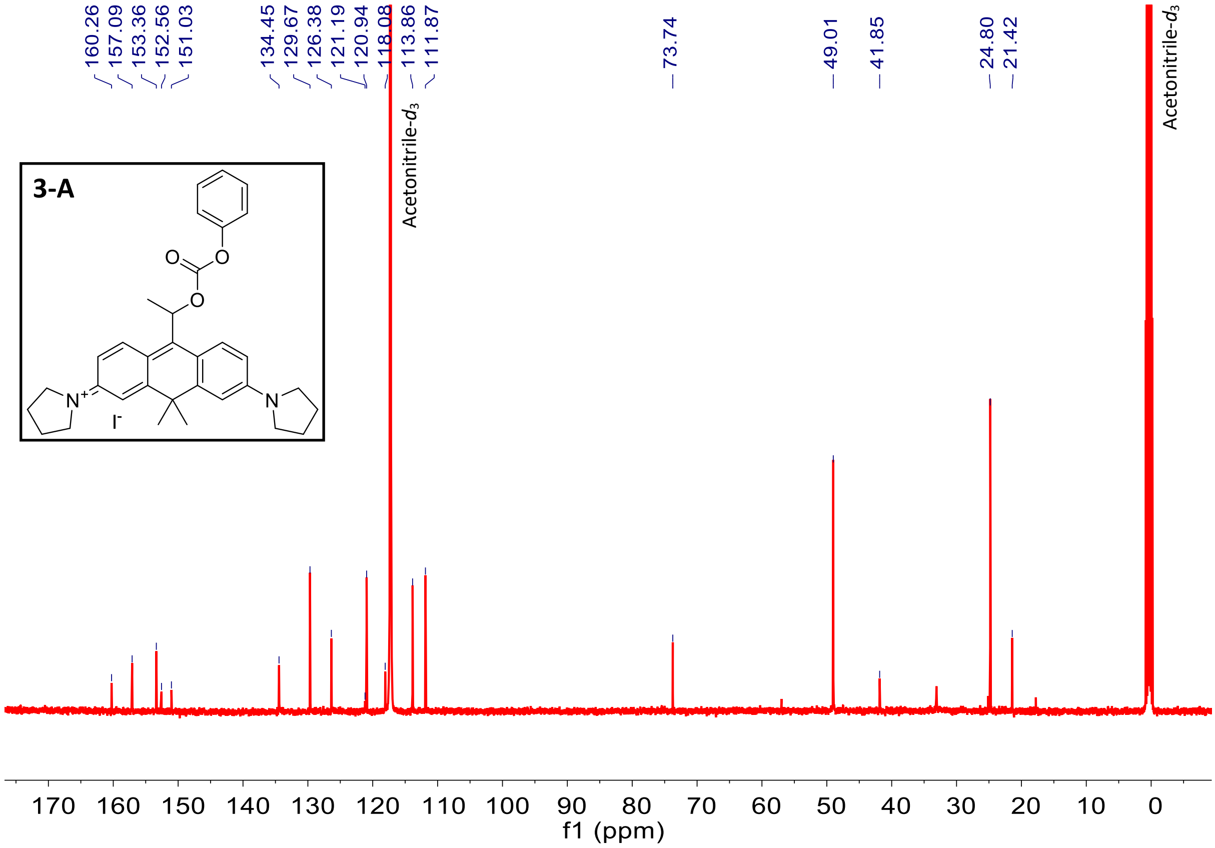
160.26
157.09
153.36
152.56
151.03
134.45
129.67
126.38
121.19
120.94
118.08
113.86
111.87
73.74
49.01
41.85
24.80
21.42
Acetonitrile-d_3
3-A
f1 (ppm)

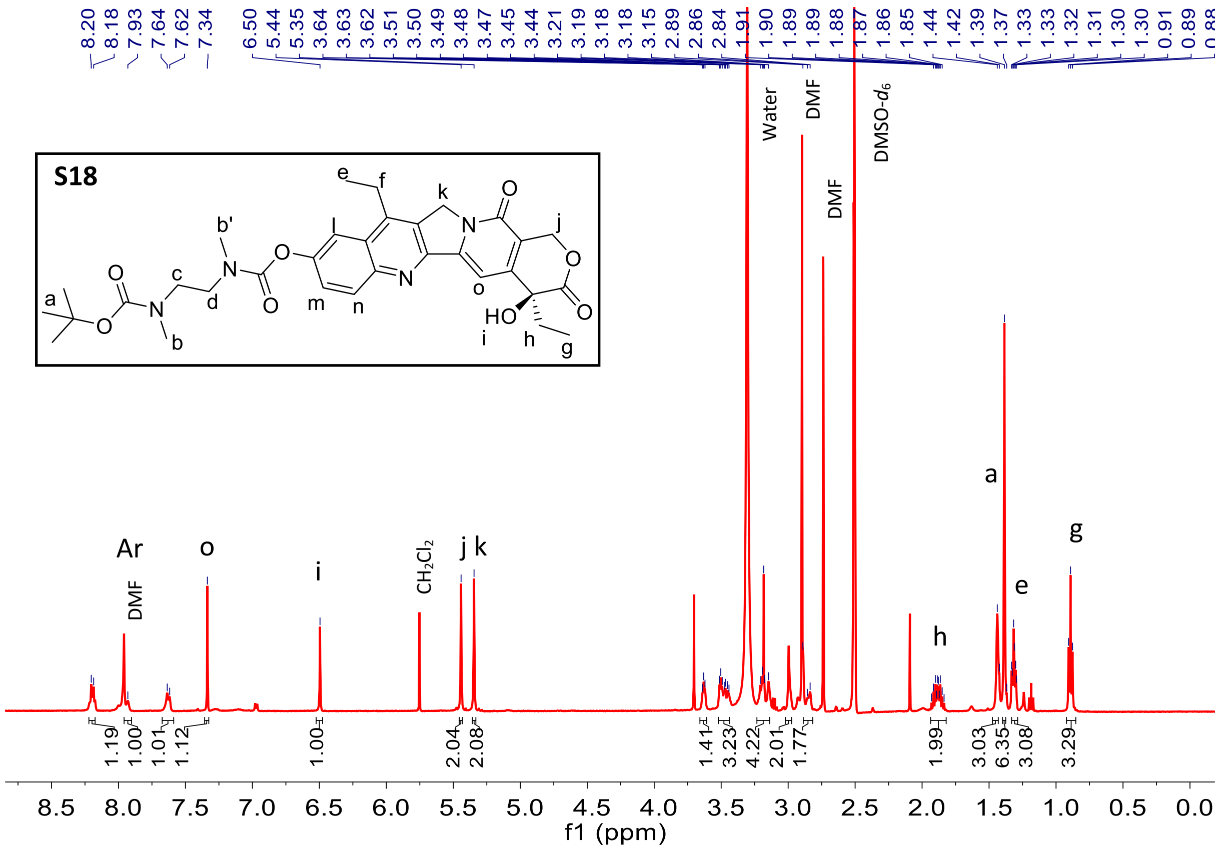
S18
Water
DMF
DMSO-d_6
CH_2Cl_2
f1 (ppm)

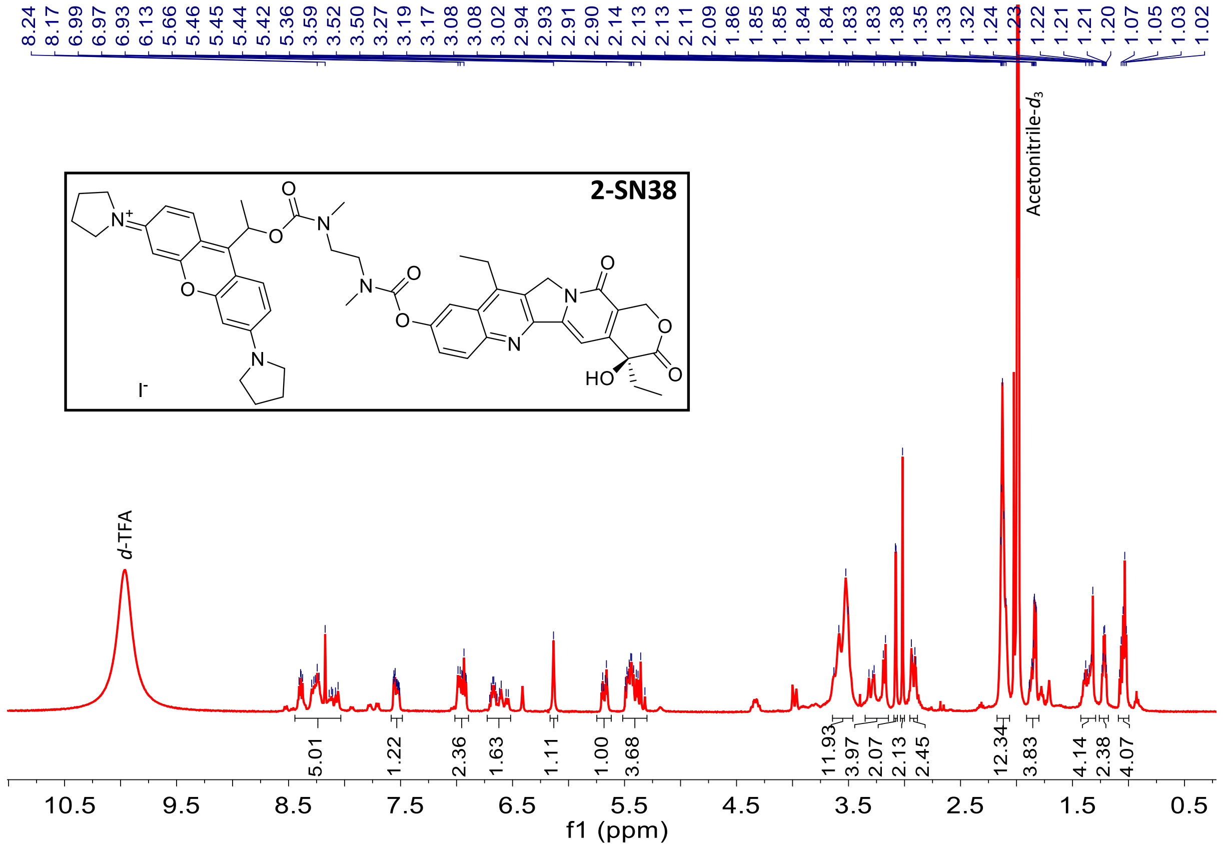
2-SN38
Acetonitrile-d_3
d-TFA
f1 (ppm)

2-SN38

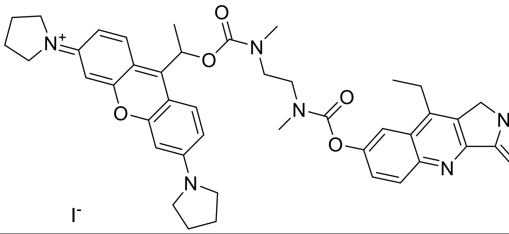

Date Acquired	: 2022.01.31. 9:29:31	Report Format File	: Alap_report.lsr
Sample Name	: EA-6-727-90-10-0min-zöld	Background Data File	: bg_220131_8min.lcd
Tray#	: 1	Batch File	: batch_220131.lcb
Vial#	: 92	Option 1	: EA
Injection Volume	: 6	Option 2	: 103
Data File	: EA-6-727-90-10-0min-zöld.lcd		
Method File	: Alapmodszer_ESI_8min_1ml.lcm		

Chromatogram

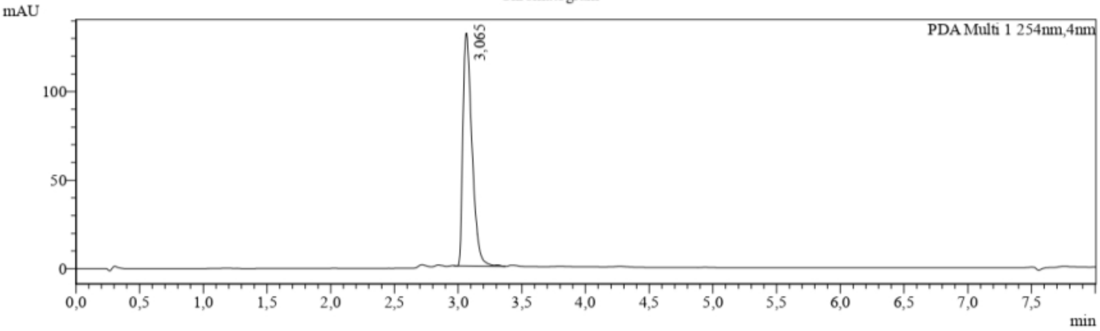

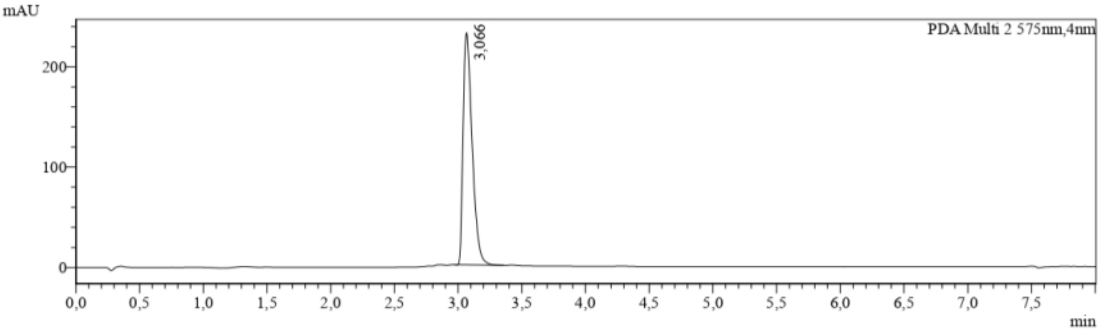

MS Chromatogram

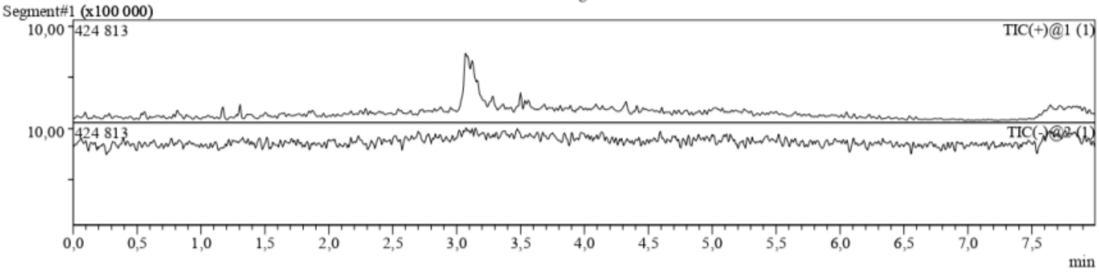

Peak Table

PDA Ch1 254nm

Peak#	Ret. Time	Area	Area%	Height	Height%	Name
1	3,065	653173	100,000	131504	100,000	
Total		653173	100,000	131504	100,000	

PDA Ch2 575nm

Peak#	Ret. Time	Area	Area%	Height	Height%	Name
1	3,066	1140565	100,000	231169	100,000	
Total		1140565	100,000	231169	100,000	

MS Spectrum

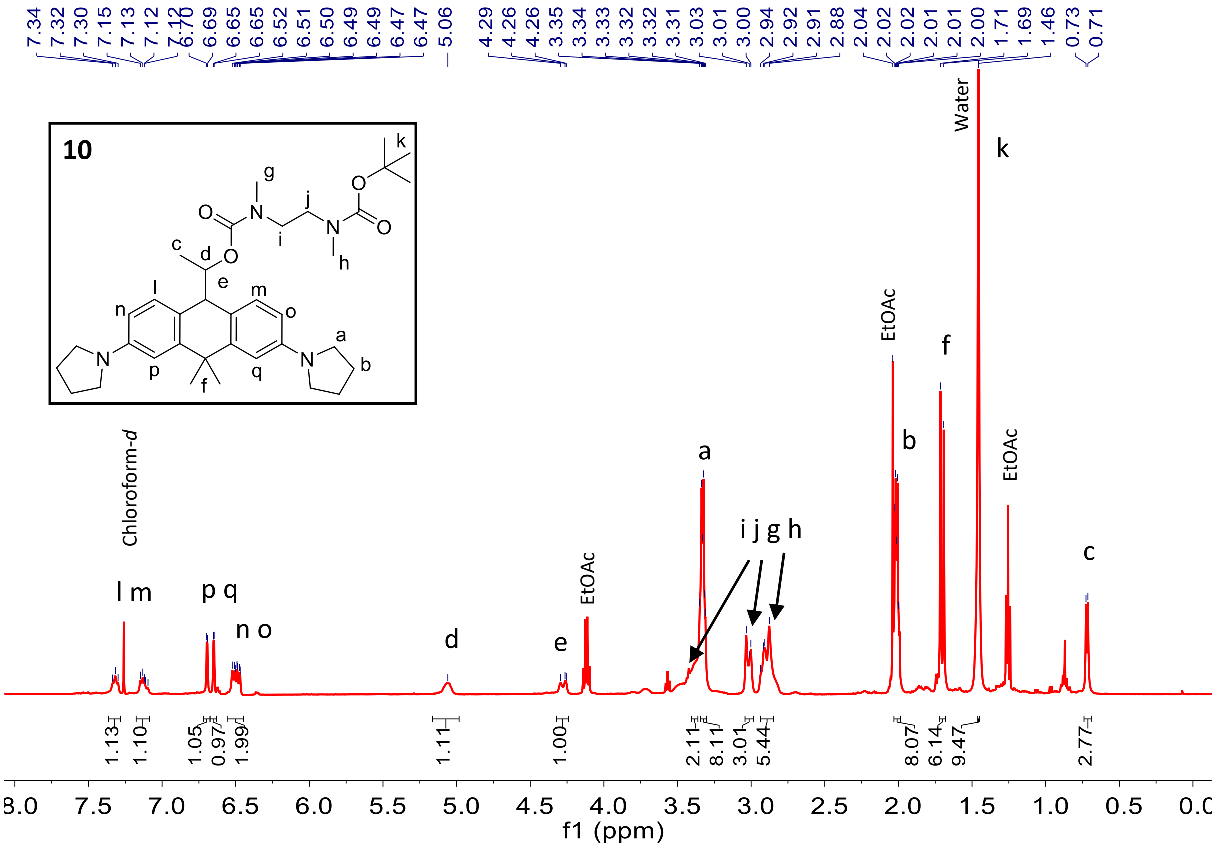
7.34 7.32 7.30 7.15 7.13 7.12 7.12 6.70 6.69 6.65 6.52 6.51 6.50 6.49 6.49 6.47 6.47 5.06 4.29 4.26 4.26 3.35 3.34 3.33 3.32 3.32 3.31 3.03 3.01 3.00 2.94 2.92 2.91 2.88 2.04 2.02 2.02 2.01 2.01 2.00 1.71 1.69 1.46 0.73 0.71
10
Chloroform-d
l m
p q
n o
d
e
EtOAc
a
i j g h
EtOAc
b
f
Water
k
EtOAc
c
1.13 1.10 1.05 0.97 1.99 1.11 1.00 2.11 8.11 3.01 5.44 8.07 6.14 9.47 2.77
f1 (ppm)

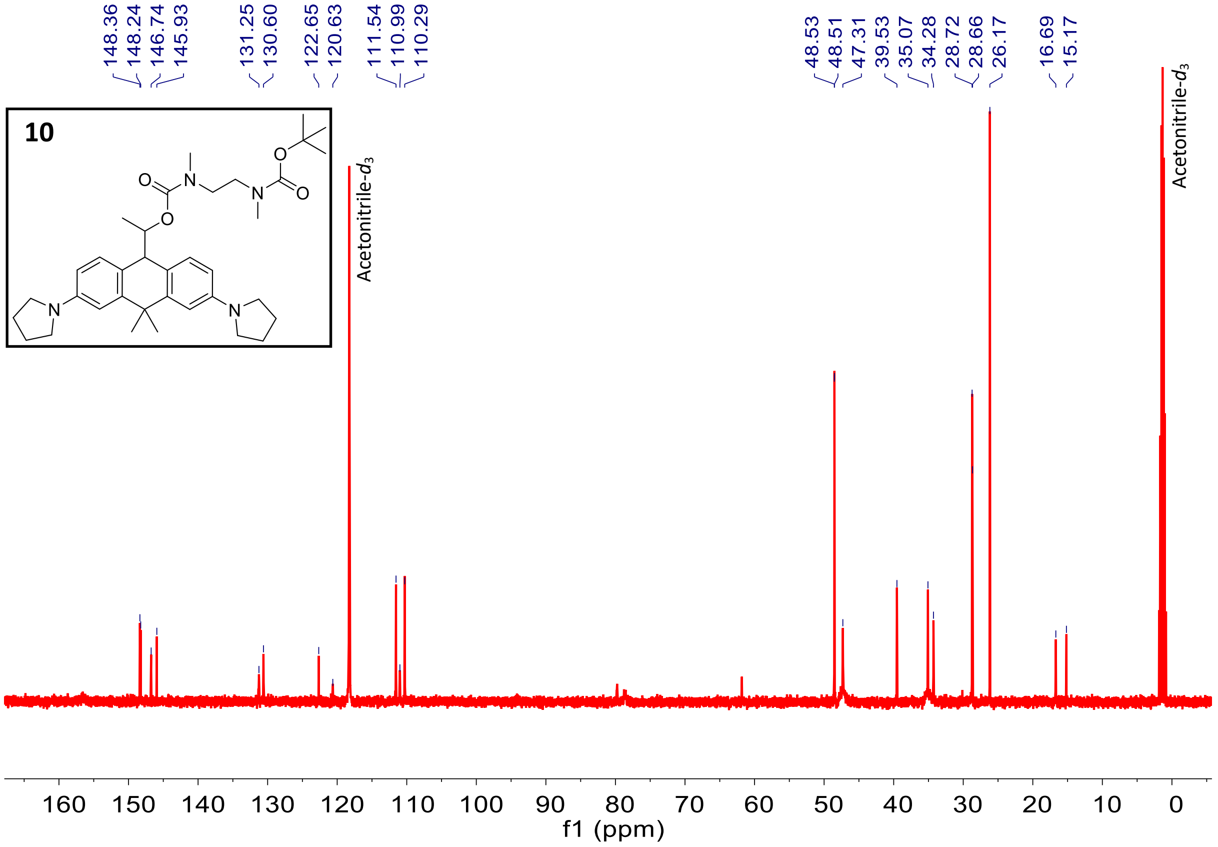
148.36 148.24 146.74 145.93 131.25 130.60 122.65 120.63 111.54 110.99 110.29 48.53 48.51 47.31 39.53 35.07 34.28 28.72 28.66 26.17 16.69 15.17
10
Acetonitrile-d3
Acetonitrile-d3
f1 (ppm)

10

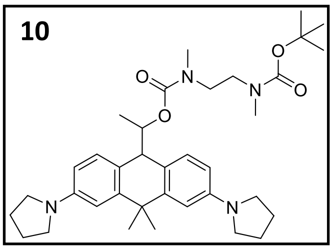

Date Acquired	: 2022.07.15. 15:57:43	Report Format File	: Alap_report.lsr
Sample Name	: EA-6-801-oszlop	Background Data File	: bg_220718.lcd
Tray#	: 1	Batch File	: batch_220715.lcb
Vial#	: 55	Option 1	: EA
Injection Volume	: 2	Option 2	: 103
Data File	: EA-6-801-oszlop.lcd		
Method File	: LowPressure_ESI_5min_0p8ml.lcm		

Chromatogram

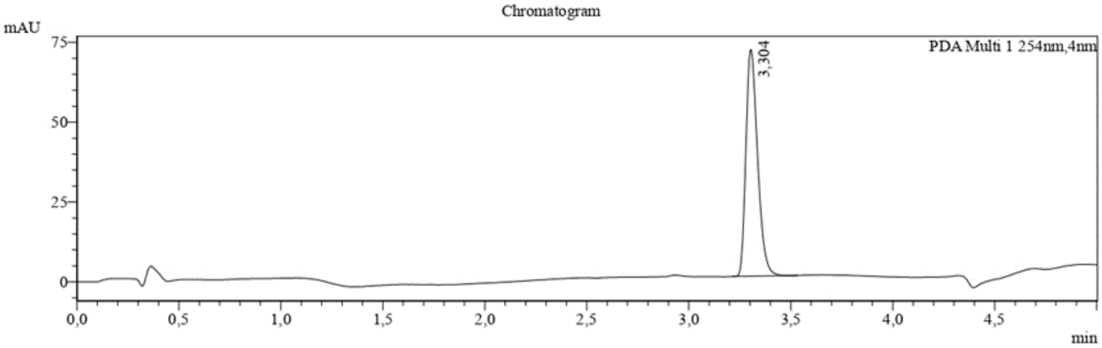

MS Chromatogram

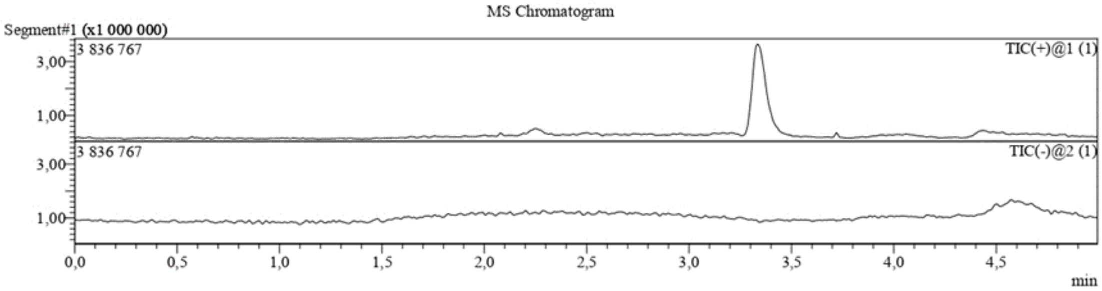

Peak Table

PDA Ch1 254nm

Peak#	Ret. Time	Area	Area%	Height	Height%	Name
1	3,304	282737	100,000	70859	100,000	
Total		282737	100,000	70859	100,000	

MS Spectrum

Line#:1 R.Time:3,347(Scan#:1005)
MassPeaks:1069
Spectrum Mode:Single 3,347(1005) Base Peak:324(841645)
BG Mode:None Segment 1 - Event 1

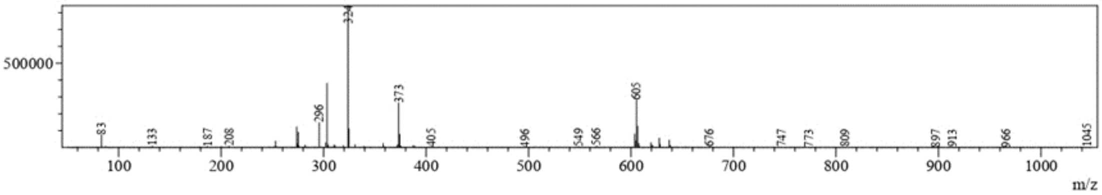

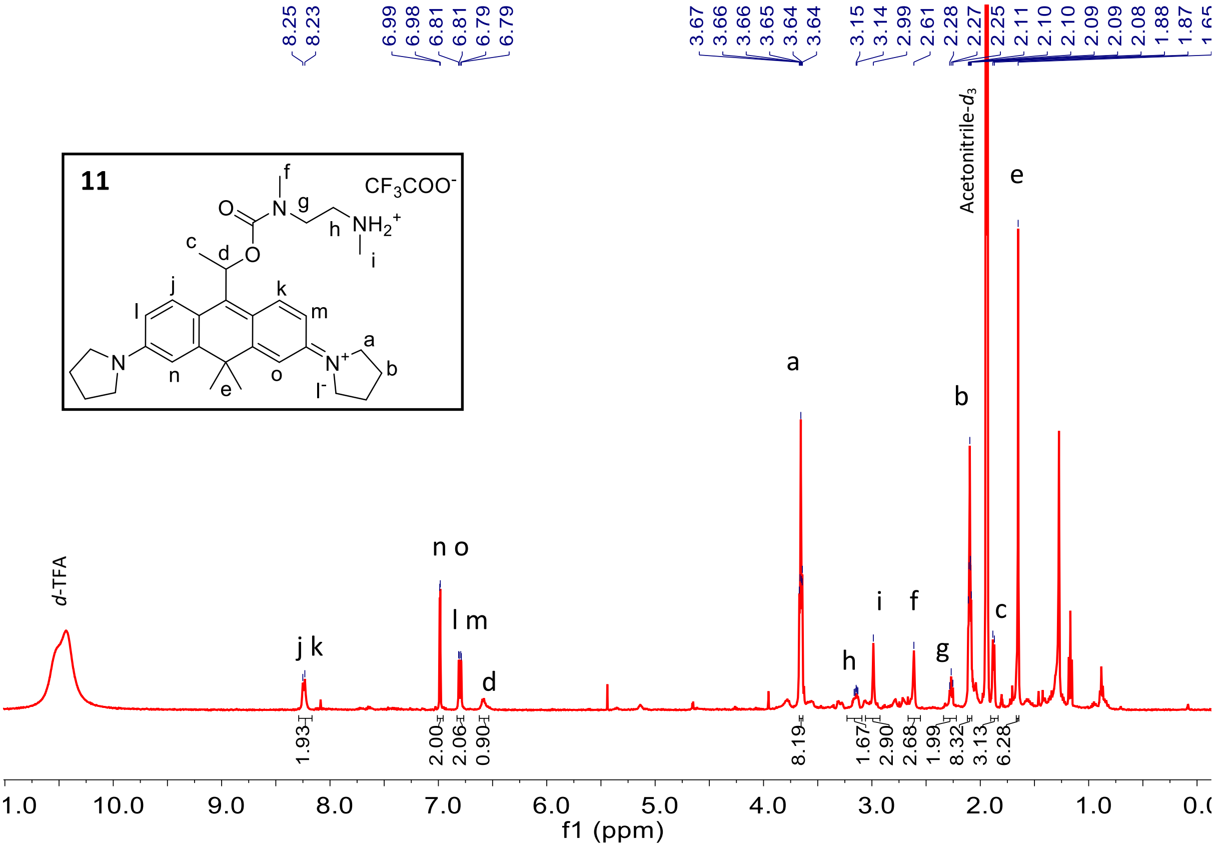
8.25
8.23
6.99
6.98
6.81
6.81
6.79
6.79
3.67
3.66
3.66
3.65
3.64
3.64
3.15
3.14
2.99
2.61
2.28
2.27
2.25
2.11
2.10
2.09
2.09
2.08
1.88
1.87
11
CF3COO-
Acetonitrile-d3
d-TFA
j k
n o
l m
d
a
h
i
f
g
b
c
e
1.93
2.00
2.06
0.90
8.19
1.67
2.90
2.68
1.99
8.32
3.13
6.28
10.0
9.0
8.0
7.0
6.0
5.0
4.0
3.0
2.0
1.0
f1 (ppm)

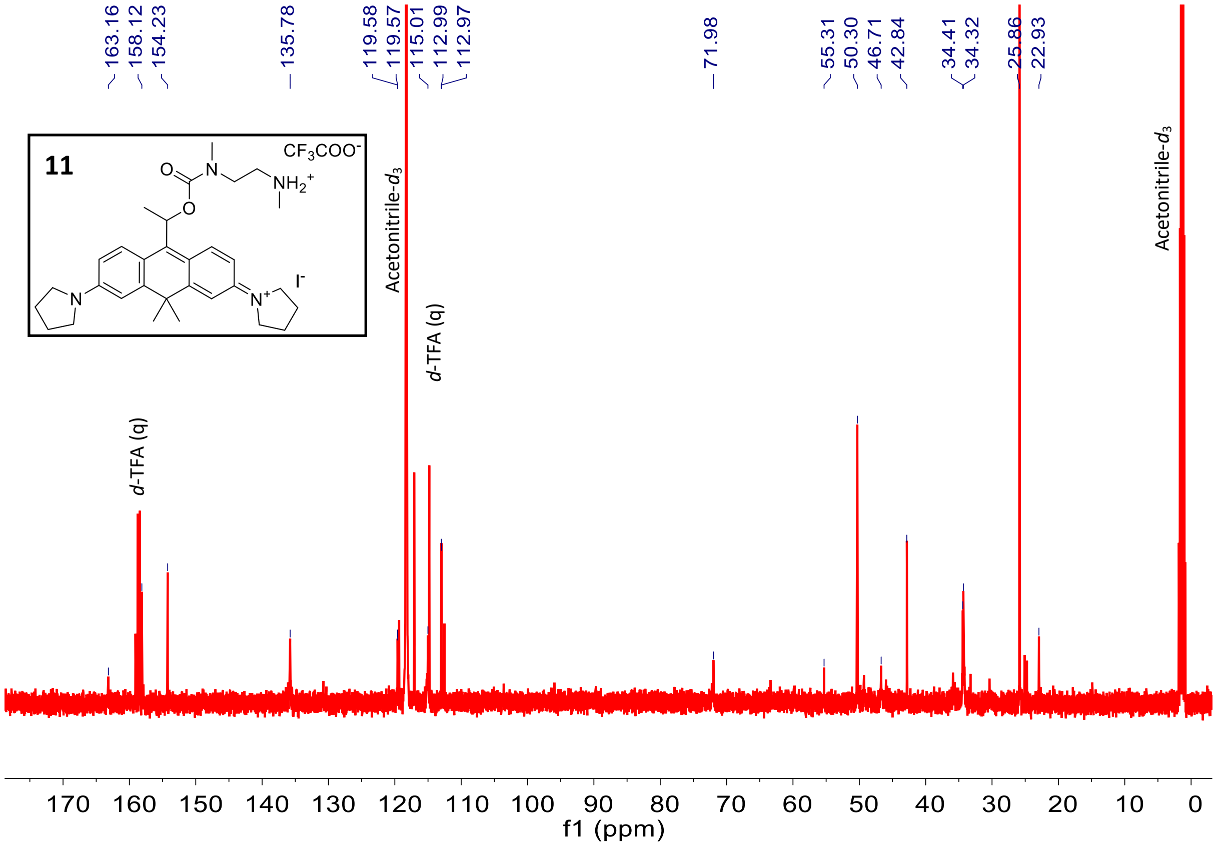
163.16
158.12
154.23
135.78
119.58
119.57
115.01
112.99
112.97
71.98
55.31
50.30
46.71
42.84
34.41
34.32
25.86
22.93
11
CF3COO-
Acetonitrile-d3
d-TFA (q)
d-TFA (q)
Acetonitrile-d3
170
160
150
140
130
120
110
100
90
80
70
60
50
40
30
20
10
0
f1 (ppm)

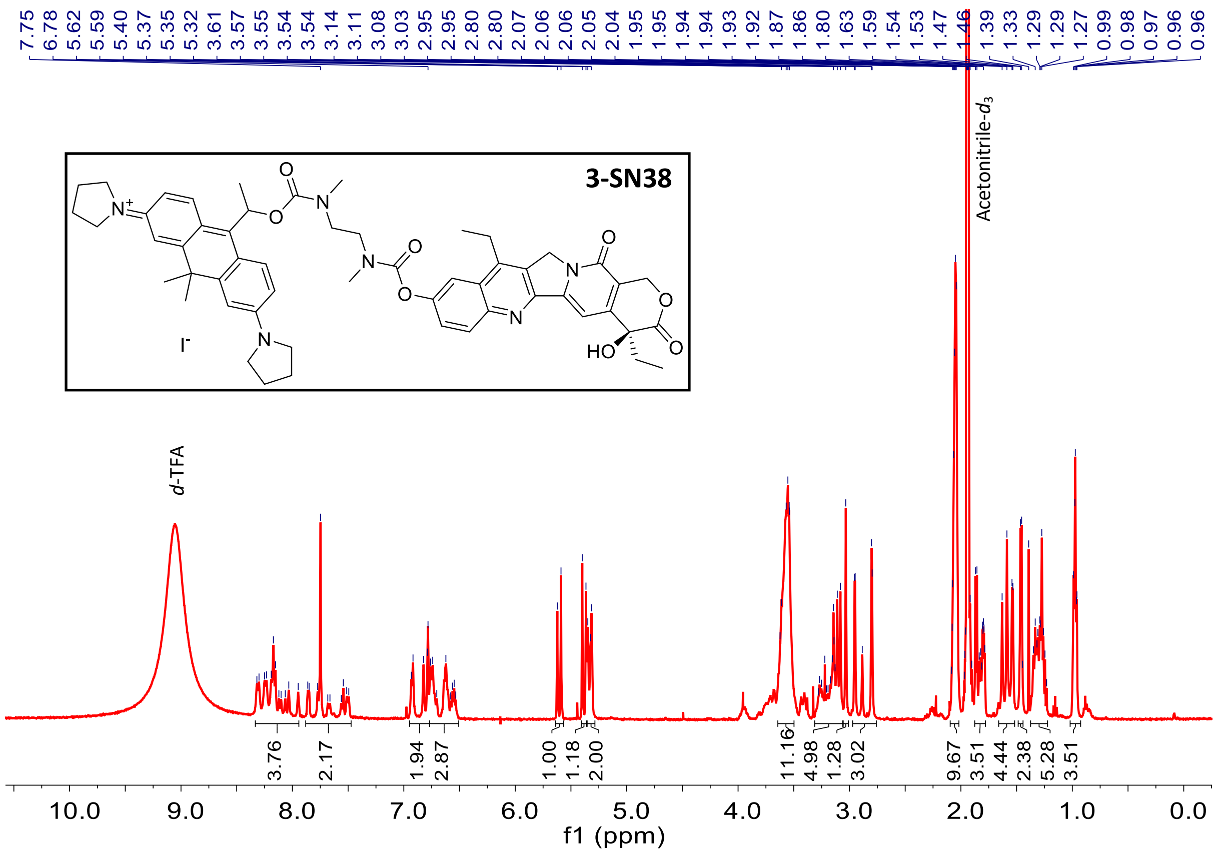

7.75 6.78 5.62 5.59 5.40 5.37 5.35 5.32 3.61 3.57 3.55 3.54 3.54 3.14 3.11 3.08 3.03 2.95 2.95 2.80 2.80 2.07 2.06 2.06 2.05 2.04 1.95 1.95 1.94 1.94 1.93 1.92 1.87 1.86 1.80 1.63 1.59 1.54 1.53 1.47 1.46 1.39 1.33 1.29 1.29 1.27 0.99 0.98 0.97 0.96 0.96
3-SN38
I⁻
HO
Acetonitrile-d3
d-TFA
3.76
2.17
1.94
2.87
1.00
1.18
2.00
11.16
4.98
1.28
3.02
9.67
3.51
4.44
2.38
5.28
3.51
10.0 9.0 8.0 7.0 6.0 5.0 4.0 3.0 2.0 1.0 0.0
f1 (ppm)

3-SN38

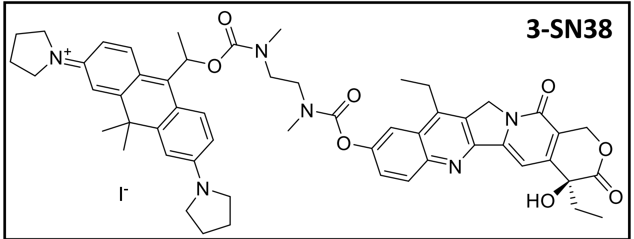

Date Acquired	: 2022.01.20. 8:10:18	Report Format File	: EA-6-730-0min.lsr
Sample Name	: EA-6-730-90-10-sima-0min	Background Data File	: bg_220120-8min.lcd
Tray#	: 1	Batch File	: batch_220120.lcb
Vial#	: 76	Option 1	: EA
Injection Volume	: 6	Option 2	: 103
Data File	: EA-6-730-90-10-sima-0min.lcd		
Method File	: Alapmodszer_ESI_8min_1ml.lcm		

Chromatogram

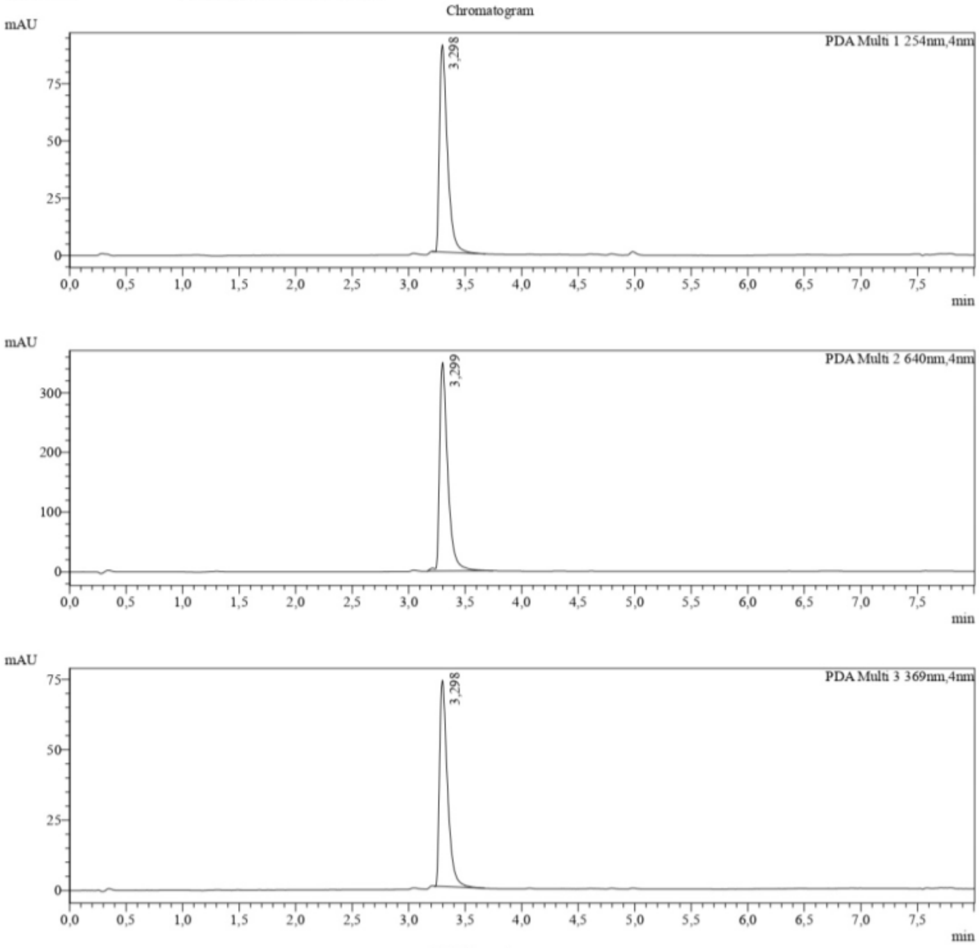

MS Chromatogram

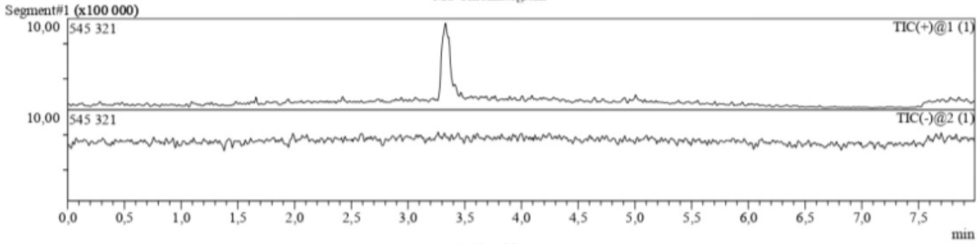

Peak Table

PDA Ch1 254nm

Peak#	Ret. Time	Area	Area%	Height	Height%	Name
1	3,298	438132	100,000	90518	100,000	

Peak#	Ret. Time	Area	Area%	Height	Height%	Name
Total		438132	100,000	90518	100,000	

PDA Ch2 640nm

Peak#	Ret. Time	Area	Area%	Height	Height%	Name
1	3,299	1748934	100,000	349375	100,000	
Total		1748934	100,000	349375	100,000	

PDA Ch3 369nm

Peak#	Ret. Time	Area	Area%	Height	Height%	Name
1	3,298	356631	100,000	73334	100,000	
Total		356631	100,000	73334	100,000	

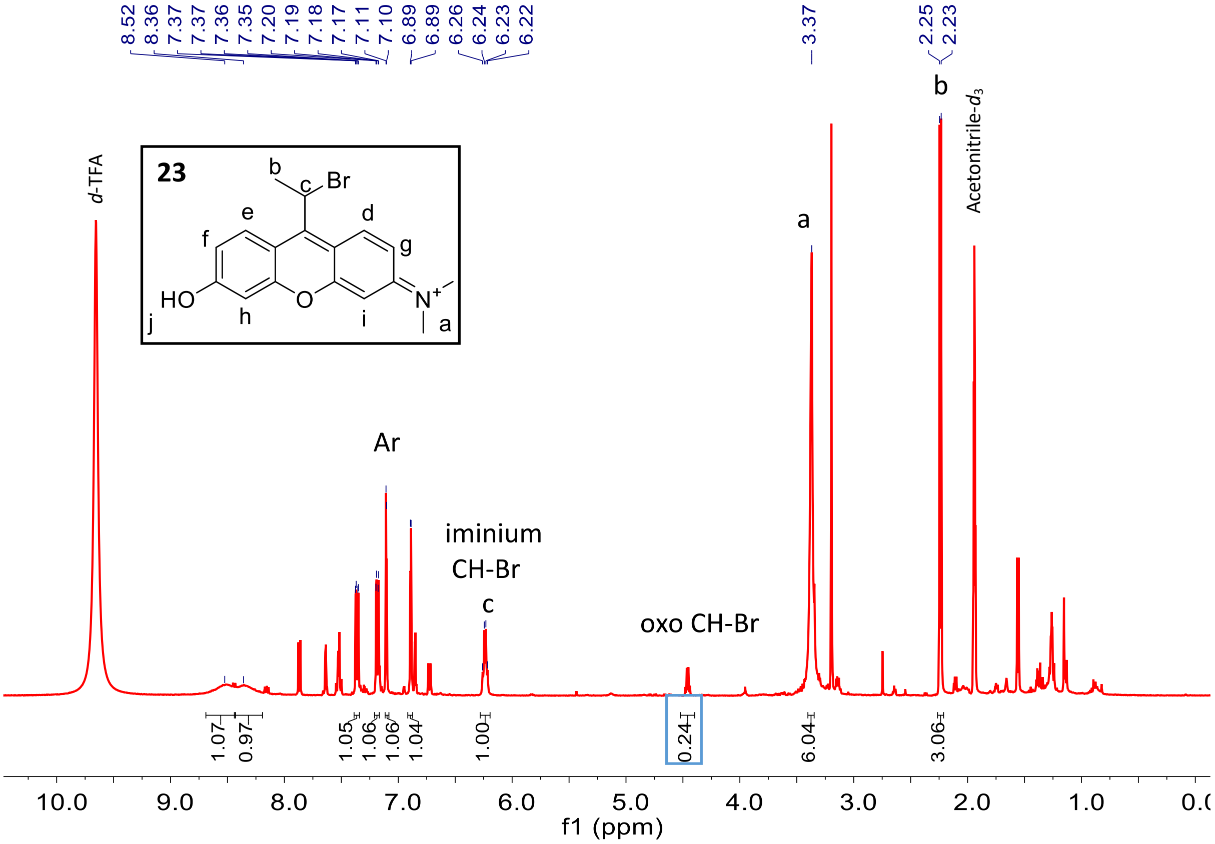

8.52
8.36
7.37
7.37
7.36
7.35
7.20
7.19
7.18
7.17
7.11
7.10
6.89
6.89
6.26
6.24
6.23
6.22
3.37
2.25
2.23
23
b
c
Br
e
d
f
g
HO
O
N+
j
h
i
a
d-TFA
Ar
iminium
CH-Br
c
oxo CH-Br
a
b
Acetonitrile-d_3
1.07
0.97
1.05
1.06
1.06
1.04
1.00
0.24
6.04
3.06
10.0
9.0
8.0
7.0
6.0
5.0
4.0
3.0
2.0
1.0
f1 (ppm)

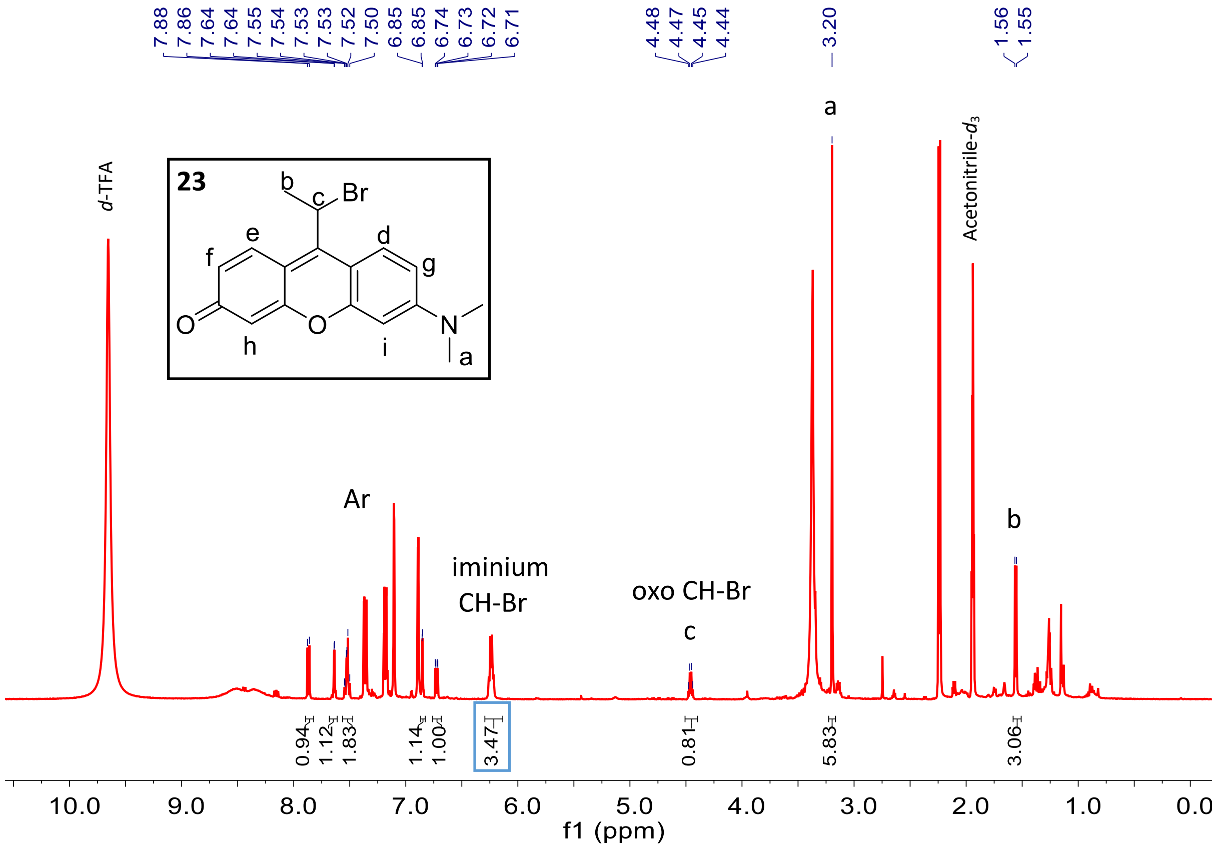

7.88
7.86
7.64
7.55
7.54
7.53
7.53
7.52
7.50
6.85
6.85
6.74
6.73
6.72
6.71
4.48
4.47
4.45
4.44
3.20
1.56
1.55
23
b
c
Br
e
d
f
g
O
O
N
h
i
a
d-TFA
Ar
iminium
CH-Br
oxo CH-Br
c
a
b
Acetonitrile-d_3
0.94
1.12
1.83
1.14
1.00
3.47
0.81
5.83
3.06
10.0
9.0
8.0
7.0
6.0
5.0
4.0
3.0
2.0
1.0
0.0
f1 (ppm)

9. References

[1]: Thiel Z.; Prof. Dr. Rivera-Fuentes, P. Single-Molecule Imaging of Active Mitochondrial Nitroreductases Using a Photo-Crosslinking Fluorescent Sensor. *Angew. Chem. Int. Ed.* **2019**, *58* (33), 11474 – 11478. https://doi.org/10.1002/anie.201904700.

[2]: Grimm, J. B.; J. Sung, A; R. Legant, W.; Hulamm, P.; M. Matlosz, S.; Betzig, E.; D. Lavis, L. Carbofluoresceins and Carborhodamines as Scaffolds for High-Contrast Fluorogenic Probes. *ACS Chem. Biol.* **2013**, *8* (6), 1303–1310. https://doi.org/10.1021/cb4000822.

[3]: Jia S.; M. Ramos-Torres, K.; Kolemen, S.; M. Ackerman, C.; J. Chang, C. Tuning the Color Palette of Fluorescent Copper Sensors through Systematic Heteroatom Substitution at Rhodol Cores. *ACS Chem. Biol.* **2018**, *13* (7), 1844–1852. https://doi.org/10.1021/acschembio.7b00748.

[4]: WO2013/180811, 2013, A1, Location in patent: Paragraph 0305; Wu, L.; Burgess, K. Synthesis and Spectroscopic Properties of Rosamines with Cyclic Amine Substituents. *J. Org. Chem.* **2008**, *73* (22), 8711–8718. https://doi.org/10.1021/jo800902j.; Kraus, G. A.; Guney, T.; Kempema, A.; Hyman, J. M.; Parvin, B. Efficient Synthesis of Fluorescent Rosamines: Multifunctional Platforms for Cellular Imaging. *Tetrahedron Lett.* **2014**, *55* (9), 1549–1551. https://doi.org/10.1016/j.tetlet.2014.01.067.

[5]: Dal Corso, A.; Borlandelli, V.; Corno, C.; Perego, P.; Belvisi, L.; Pignataro, L.; Gennari, C. Fast Cyclization of a Proline-Derived Self-Immolative Spacer Improves the Efficacy of Carbamate Prodrugs. *Angew. Chem. Int. Ed.* **2020**, *59* (10), 4176–4181. https://doi.org/10.1002/anie.201916394.

[6] (Slanina, T.; Shrestha, P.; Palao, E.; Kand, D.; Peterson, J. A.; Dutton, A. S.; Rubinstein, N.; Weinstain, R.; Winter, A. H.; Klán, P. In Search of the Perfect Photocage: Structure–Reactivity Relationships in Meso -Methyl BODIPY Photoremovable Protecting Groups. *J. Am. Chem. Soc.* **2017**, *139* (42), 15168–15175. https://doi.org/10.1021/jacs.7b08532.

[7] Yanai, T.; P. Tew, D.; C. Handy, N. *A new hybrid exchange–correlation functional using the Coulomb-attenuating method (CAM-B3LYP). Chem. Phys. Lett.* **2004**, *393*, 51-57. https://doi.org/10.1016/j.cplett.2004.06.011.

[8] Scalmani, G.; Frisch, M. J.; Mennucci, B.; Tomasi, J.; Cammi, R.; Barone, V. Geometries and properties of excited states in the gas phase and in solution: theory and application of a time-dependent density functional theory polarizable continuum model. *J. Chem. Phys.* **2006**, *124*, 094107 . https://doi.org/10.1063/1.2173258.

[9] Tomasi, J.; Mennucci B.; Cammi, R. Quantum Mechanical Continuum Solvation Models. *Chem. Rev.* **2005,** *105*, 2999–3094. https://doi.org/10.1021/cr9904009.

[10] Gaussian 09, Revision B.01, M. J. Frisch, G. W. Trucks, H. B. Schlegel, G. E. Scuseria, M. A. Robb, J. R. Cheeseman, G. Scalmani, V. Barone, B. Mennucci, G. A. Petersson, H. Nakatsuji, M. Caricato, X. Li, H. P. Hratchian, A. F. Izmaylov, J. Bloino, G. Zheng, J. L. Sonnenberg, M. Hada, M. Ehara, K. Toyota, R. Fukuda, J. Hasegawa, M. Ishida, T. Nakajima, Y. Honda, O. Kitao, H. Nakai, T. Vreven, J. A. Montgomery, Jr., J. E. Peralta, F. Ogliaro, M. Bearpark, J. J. Heyd, E. Brothers, K. N. Kudin, V. N. Staroverov, T. Keith, R. Kobayashi, J. Normand, K. Raghavachari, A. Rendell, J. C. Burant, S. S. Iyengar, J. Tomasi, M. Cossi, N. Rega, J. M. Millam, M. Klene, J. E. Knox, J. B. Cross, V. Bakken, C. Adamo, J. Jaramillo, R. Gomperts, R. E. Stratmann, O. Yazyev, A. J. Austin, R. Cammi, C. Pomelli, J. W. Ochterski, R. L. Martin, K. Morokuma, V. G. Zakrzewski, G. A. Voth, P. Salvador, J. J. Dannenberg, S. Dapprich, A. D. Daniels, O. Farkas, J. B. Foresman, J. V. Ortiz, J. Cioslowski, and D. J. Fox, Gaussian, Inc., Wallingford CT, 2010.

[11] Knizia, G.; Klein, J. E. M. N.Electron flow in reaction mechanisms – revealed from first principles. *Angew. Chem. Int. Ed.* **2015**, *54*, 5518-5522. https://doi.org/10.1002/anie.201410637.
